# DFT insight into asymmetric alkyl–alkyl bond formation *via* nickel-catalysed enantioconvergent reductive coupling of racemic electrophiles with olefins†

**DOI:** 10.1039/d1sc05605k

**Published:** 2022-02-25

**Authors:** Chao-Shen Zhang, Bei-Bei Zhang, Liang Zhong, Xiang-Yu Chen, Zhi-Xiang Wang

**Affiliations:** School of Chemical Sciences, University of Chinese Academy of Sciences Beijing 100049 China chenxiangyu20@ucas.ac.cn zxwang@ucas.ac.cn

## Abstract

A DFT study has been conducted to understand the asymmetric alkyl–alkyl bond formation through nickel-catalysed reductive coupling of racemic alkyl bromide with olefin in the presence of hydrosilane and K_3_PO_4_. The key findings of the study include: (i) under the reductive experimental conditions, the Ni(ii) precursor is easily activated/reduced to Ni(0) species which can serve as an active species to start a Ni(0)/Ni(ii) catalytic cycle. (ii) Alternatively, the reaction may proceed *via* a Ni(i)/Ni(ii)/Ni(iii) catalytic cycle starting with a Ni(i) species such as Ni(i)–Br. The generation of a Ni(i) active species *via* comproportionation of Ni(ii) and Ni(0) species is highly unlikely, because the necessary Ni(0) species is strongly stabilized by olefin. Alternatively, a cage effect enabled generation of a Ni(i) active catalyst from the Ni(ii) species involved in the Ni(0)/Ni(ii) cycle was proposed to be a viable mechanism. (iii) In both catalytic cycles, K_3_PO_4_ greatly facilitates the hydrosilane hydride transfer for reducing olefin to an alkyl coupling partner. The reduction proceeds by converting a Ni–Br bond to a Ni–H bond *via* hydrosilane hydride transfer to a Ni–alkyl bond *via* olefin insertion. On the basis of two catalytic cycles, the origins for enantioconvergence and enantioselectivity control were discussed.

## Abstract 2

The enantioconvergent alkyl–alkyl coupling involves two competitive catalytic cycles with nickel(0) and nickel(i) active catalysts, respectively. K_3_PO_4_ plays a crucial role to enable the hydride transfer from hydrosilane to nickel–bromine species.

## Graphical abstract

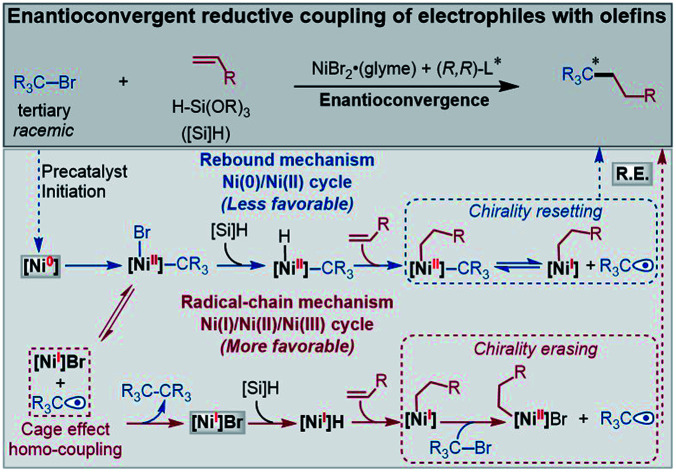

## Introduction

**Scheme 1:**
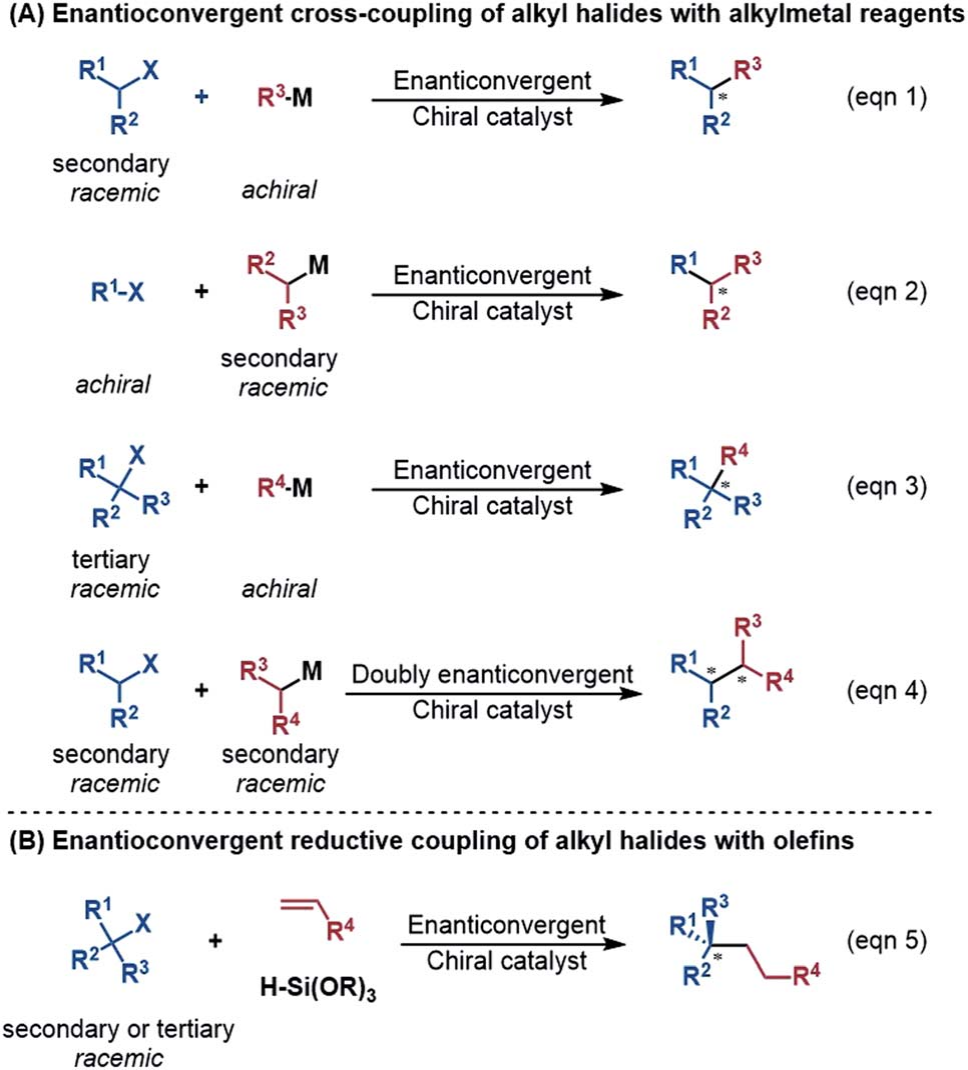
Various nickel-catalysed enantioconvergent alkyl–alkyl bond formations.

An alkyl–alkyl bond is a typical bonding force to build molecules, and the stereochemistry of the carbons greatly affects the structures and properties of molecules. Thus, the development of methods for alkyl–alkyl bond formation with controlled enantioselectivity is of great importance in organic synthesis.^1^ Transition metal-catalysed asymmetric cross-coupling of alkyl electrophiles and alkylmetal nucleophiles is an effective approach to achieve the goal.^2^ Over the few past decades, nickel catalysis has been demonstrated to be particularly effective due to the multiple accessible oxidation states of nickel and the advantage of the catalysis avoiding undesirable β-H elimination.^3^ Fu *et al.*^2*a*,4^ and other groups^5^ reported a series of nickel-catalysed enantioconvergent alkyl–alkyl cross-couplings of secondary racemic alkyl electrophiles/nucleophiles with achiral alkyl partners (eqn (1) and (2) in Scheme 1). Recently, Fu *et al.* further accomplished more challenging cross-couplings, including the enantioconvergent coupling of racemic tertiary alkyl halides (eqn (3))^6^ and the doubly enantioconvergent coupling of racemic alkyl halides and racemic alkylmetal reagents (eqn (4)).^7^ Alternative to nickel-catalysed cross-coupling of alkyl electrophiles and alkylmetal nucleophiles, nickel catalysis also performed well to couple alkyl electrophiles with olefins for alkyl–alkyl bond construction. The use of readily available olefins as nucleophiles overcomes the disadvantages of organometallic reagents such as moisture- and air-sensitivity, harsh reaction conditions, inconvenient operation, and poor tolerance of functional groups. In this context, Fu *et al.* in 2018 made another breakthrough and developed a nickel-catalysed enantioconvergent reductive coupling of racemic alkyl electrophiles with olefins in the presence of hydrosilane (eqn (5) in Scheme 1).^8^ Notably, the reductive coupling also performed well with racemic tertiary alkyl halides which are challenging for the electrophile–nucleophile coupling approach.^9^ Since then, more and more asymmetric reductive couplings of olefins with alkyl electrophiles have been developed by the groups of Fu at Caltech, Zhu, Fu and Lu at China's USTC, Shu, and Hu.^10^

**Scheme 2:**
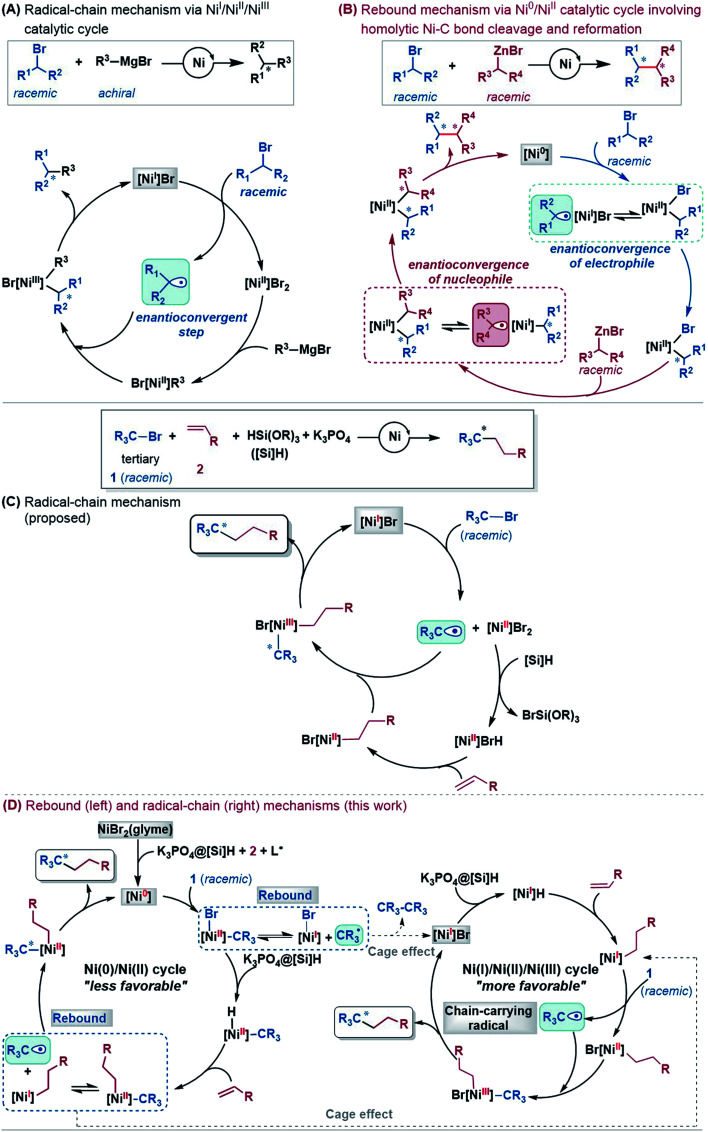
Mechanisms for nickel-catalysed enantioconvergent cross-couplings of alkyl electrophiles.

Mechanistically, depending on the characters of nucleophiles, electrophiles, ligands and additives, diverse pathways have been postulated to account for those enantioconvergent alkyl–alkyl bond forming reactions.^3,5*a*,*b*,11^ Nevertheless, these pathways share a common feature involving a nickel(i)–halogen active species. For example, on the basis of their elegant and extensive mechanistic study, Fu *et al.* proposed a radical-chain mechanism to account for the enantioconvergent Kumada coupling (Scheme 2A). With a nickel(i)–Br species as the chain-carrying radical, the coupling undergoes a Ni(i)/Ni(ii)/Ni(iii) catalytic cycle involving Br-transfer, transmetalation, alkyl radical association, and reductive elimination. The halogen-transfer step converts the alkyl electrophile to an alkyl radical, thus erasing the chirality of the racemic alkyl electrophile to achieve enantioconvergence. The stereospecific additions of the alkyl radical to the Br–Ni(ii)–alkyl species control the enantioselectivity. For the reductive cross-coupling of alkyl electrophiles with alkenes, Ni(ii)–halogen and halogen–Ni(ii)–H species were often considered to be the key species in the catalytic cycle, as exemplified by Scheme 2C for the reaction in eqn (5).

In the past decade, theoretical calculation has been demonstrated to be a powerful tool to gain insight into catalytic mechanisms in greater detail. However, these asymmetric alkyl–alkyl bond formation reactions present challenges for computational study, because of the elusive/undefined active catalysts, multiple possible pathways, multiple spin states of the involved species, and the involvement of a single-electron transfer process. To our knowledge, there has been no systematic computational study to account for the catalytic mechanisms, the enantioconvergence, and enantioselectivity of these reactions. Recently, we carried out a DFT study to investigate the mechanism of the nickel-catalysed doubly enantioconvergent coupling of racemic alkyl nucleophiles with racemic electrophiles.^12^ On the basis of our computed energetic results and the reported experimental mechanistic study results, we proposed a so-called rebound mechanism to account for the double enantioconvergence (Scheme 2B). Under the catalytic conditions, the nickel precatalyst is first activated to a Ni(0) active species. The coupling then undergoes a Ni(0)/Ni(ii) catalytic cycle *via* a sequence of oxidative addition, transmetalation, and reductive elimination. Interestingly, the Ni(ii) intermediates formed from oxidative addition and transmetalation are able to undergo homolytic Ni–C bond cleavage and reformation, thus resetting the chirality of the Ni(ii) intermediates for enantioselective reductive elimination. Notably, Molander, Kozlowski, Gutierrez and coworkers reported that a Ni(iii) intermediate could also undergo Ni–C bond cleavage and reformation to afford an enantioselective product in their cross-coupling reaction enabled by photoredox/nickel dual catalysis.^13^

Continuing our research interest, we attempted to gain insight into the mechanisms of the reductive cross-coupling of alkyl electrophiles with olefins. Specifically, we intended to address the following questions: (i) what is the actual catalyst and how is it generated? (ii) How is olefin transformed into an alkyl coupling partner to form an alkyl–alkyl bond? (iii) How does the nickel catalysis enable the enantioconvergence and how does the chiral ligand control the enantioselectivity? (iv) As the experimental study has shown an indispensable role of K_3_PO_4_, the proposed catalytic cycle (Scheme 2C) does not invoke the base. We unveil the unclear role of the base and how it acts. Expectedly, these insights could aid the rational development of more general enantioconvergent alkyl–alkyl bond forming reactions.

## Computational details

In this study, we used the experimental reaction (eqn (6)) as the representative to compute the reaction pathways. Considering the large size of the system, we adopted the cost-effective M06//B3LYP protocol, which was recommended by Houk *et al.* to study transition metal-catalysed reactions^14^ and was successfully applied to study many nickel-catalysed reactions.^15^ All the structures were optimized at the B3LYP/BSI level in the gas phase, BSI representing a basis set with SDD^16^ for Ni and Br and 6-31G(d,p) for the other atoms. Depending on the nature of a species, the B3LYP calculations could be restricted B3LYP (RB3LYP) for closed-shell singlet species, unrestricted B3LYP (UB3LYP) for doublet and triplet species, or broken-symmetry B3LYP (BSB3LYP) for open-shell singlet species. Particular attention was paid to the singlet species. When the wavefunction of a closed shell singlet species was found to be unstable, the open shell singlet was recalculated with BSB3LYP.^17^ Harmonic frequency analysis calculations at the same level were performed to verify the optimized geometries to be minima (no imaginary frequency) or transition states (TSs, having one unique imaginary frequency). The energies were further improved by M06 (ref. 18)/BSII//B3LYP/BSI single point calculations with solvent effects simulated by the SMD^19^ solvent model with the experimental solvent tetrahydrofuran. BSII denotes a basis set with SDD for Ni and Br and 6-311++G(d,p) for the other atoms. Harmonic vibration frequencies at the B3LYP/BSI level were used to correct the single point energies to free energies at 298.15 K and 1 atm, which are used to discuss the mechanism in the main text. The results related to spin contaminations are given in Tables S1 and S2 in SI1,† which show that the effects of spin contaminations are negligible. The reaction pathways involve nickel species in different spin states. We use left superscripts to specify the spin multiplicities of structures, with 1, 2, and 3 denoting a singlet, doublet and triplet, respectively.

To verify the reliability of the calculation protocol, we computed the energetics of the key processes at other levels of DFT calculations. As compared in the ESI (Fig. S2 and S3 in SI2†), these results agree with those reported in the main text and do not change our conclusions.
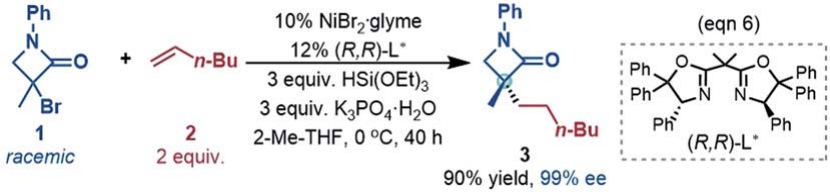


Intrinsic reaction coordinate (IRC) calculations for important transition states were carried out at the B3LYP/BSI level to verify these transition states correctly connecting with their nearby minima.^20^ Natural bond orbital (NBO) analyses were performed at the M06//BSII level to assign partial atomic charges (*Q*).^21^ All DFT calculations were conducted with the Gaussian 09 program.^22^ To analyse the origins of the enantioselectivity, noncovalent interaction (NCI) analyses were carried out. The cubic files from NCI analyses were generated with the Multiwfn program^23^ and visualized with the VMD program.^24^ The displayed structures were drawn with the CYLview.^25^ The SCF energies, free energies, and Cartesian coordinates of all optimized structures are given in SI13.†

## Results and discussion

### Mechanism for precatalyst initiation generating the nickel(0) species

To fully understand a catalytic transformation, it is a starting point to identify the active catalyst. The reaction (eqn (6)) was performed by using a nickel(ii) source (NiBr_2_·glyme) in the presence of K_3_PO_4_ and hydrosilane HSi(OEt)_3_ (denoted as [Si]H hereafter). Mechanistically, a catalytic cycle (Scheme 2C) with a nickel(i) species [Ni^I^]Br as the active catalyst was proposed. Similarly, nickel(i) hydrides were also postulated to be the active catalyst in the nickel-catalysed reductive hydrofunctionalization of alkenes under similar reductive reaction conditions.^10,26^ However, these proposals have not been verified experimentally or computationally. As the characterization of active catalysts sometimes presents great challenges for experimental study due to their elusive natures, quantum mechanics computation has become an effective and convenient approach to attack the problem by providing geometric and energetic information. In the following, guided by the computed results, we analyse how the catalyst precursor was initiated and what species could be generated.

**Fig. 1:**
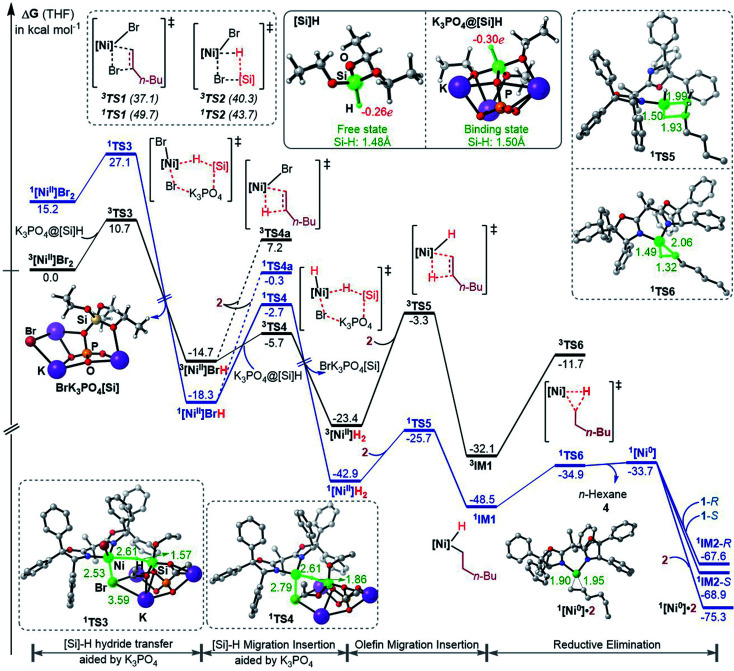
Free energy profiles for the precatalyst initiation to generate the nickel(0) active species. The key bond lengths (Å) and NBO charges in the selected structures are given. The relative free energies of ^1,3^TS3 and ^1,3^TS4 were measured with the complexation energy (22.8 kcal mol^−1^) included.

Fig. 1 shows the energy profiles for the precatalyst initiation. After the ligand (*R*,*R*)-L* undergoes ligand exchange with the precursor ^3^NiBr_2_·glyme (see Fig. S4 in SI3†), the generated ^3^[Ni^II^]Br_2_ (ref. 27) most likely reacts with olefin 1 or [Si]H, described by TS1 and TS2, respectively, but the high TS1 and TS2 (>37.0 kcal mol^−1^) rule out the possibilities.

Keeping in mind that the reaction could not occur in the absence of K_3_PO_4_, we inspected whether the additive K_3_PO_4_ could promote a hydride transfer from [Si]H to [Ni^II^]Br_2_. Since a 1 : 1 ratio of K_3_PO_4_ : [Si]H was applied in the experimental study (eqn (6)), we considered the 1 : 1 complexation of K_3_PO_4_ with [Si]H. The complexation to give a K_3_PO_4_@[Si]H complex is exergonic by 22.8 kcal mol^−1^. Note that the complexation energy of K_3_PO_4_ with [Si]H could be overestimated due to the energy cost to liberate monomeric K_3_PO_4_ from the salt aggregations. In comparison, the complexation of K_3_PO_4_ with 1 or 2, or THF is thermodynamically unfavourable (see Fig. S5 in SI4†). As a simplified model, we hereafter used the K_3_PO_4_@[Si]H complex as a substrate to consider the roles of K_3_PO_4_ and [Si]H. The complexation activates the Si–H bond apparently, as reflected by the elongated Si–H bond length (1.50 Å) and the increased negative charge (−0.30*e*) on the H atom, compared to those (1.48 Å and −0.26*e*) in the isolated [Si]H (Fig. 1). In addition, the hydride transfer concomitantly forms Si–O and K–Br bonds, which benefits the process. The K_3_PO_4_-aided hydride transfer is facile, with a barrier of 10.7 kcal mol^−1^ (^3^TS3 relative to ^3^[Ni^II^]Br_2_ + K_3_PO_4_@[Si]H) and much lower than TS1 and TS2.

Subsequent to the formation of a nickel(ii) species [Ni^II^]BrH, the same hydride transfer further converts [Ni^II^]BrH to [Ni^II^]H_2_ through TS4. The two hydride transfer processes exhibit two-state reactivity,^28^ giving the singlet ^1^[Ni]H_2_ which is 19.5 kcal mol^−1^ lower than its triplet. Then olefin migratory insertion *via*^1^TS5 and reductive elimination *via*^1^TS6 take place sequentially, leading to ^1^[Ni^0^]. The ^1^[Ni^0^] species is less stable than the alkyl nickel(ii) hydride ^1^IM1 and ^1^[Ni^II^]H_2_, but it can be significantly stabilized by the coordination of 1 or 2, forming more stable ^1^[Ni^0^]·1 (denoted as ^1^IM2 hereafter) or ^1^[Ni^0^]·2 complexes. Note that [Ni^II^]BrH and [Ni^II^]H_2_ may undergo reductive elimination to give ^1^[Ni^0^], but the processes are too endergonic (by 61.0 and 32.8 kcal mol^−1^, respectively) to be accessible.

Overall, the initiation is highly exergonic by more than 67.0 kcal mol^−1^, with a rate-determining barrier of 17.2 kcal mol^−1^ for olefin insertion (^1^TS5 relative to ^1^[Ni^II^]H_2_), indicating the facile occurrence of the initiation. Moreover, the initiation mechanism could be applied for similar catalytic systems (see SI5†).

### Coupling pathway with nickel(0) active species

**Fig. 2:**
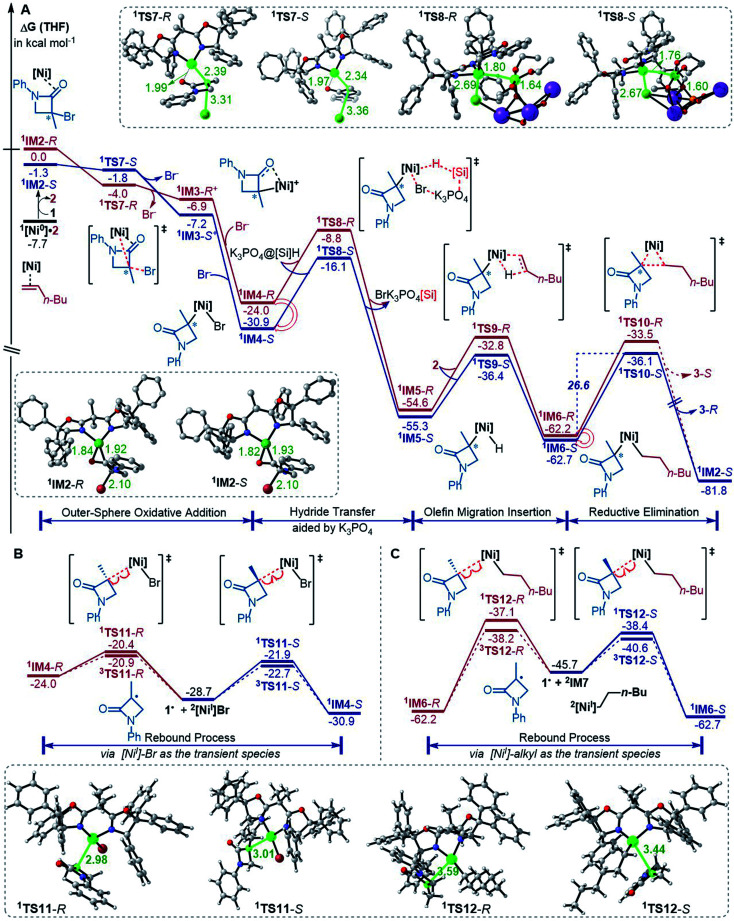
Free energy profiles (in kcal mol^−1^) for the reaction of ^1^IM2 and 2 (A) and the rebound processes (B and C). The key bond lengths in selected structures are given in angstroms.

^1^[Ni^0^]·2 is more stable than ^1^IM2, but ^1^[Ni^0^]·2 cannot react with 1 straightforwardly, because ^1^[Ni^0^]·2 has no vacant site to interact with electrophile 1. To proceed the reaction, ^1^[Ni^0^]·2 first converts to ^1^IM2*via* replacing 2 with 1. We first discuss the *S*-pathway coloured in blue, leading to 3-*R*. The substitution of 1 in ^1^[Ni^0^]·2 with 1-*S* is endergonic by 6.4 kcal mol^−1^, but the process can be driven by subsequent highly exergonic coupling processes. Subsequent to the substitution, ^1^IM2-*S* converts to ^1^IM4-*S via* dissociation–association of the Br^−^ anion. In terms of electron energy, we were able to locate the dissociation transition state (^1^TS7-*S*) in the gas phase. IRC calculations further confirmed ^1^TS7-*S* correctly connecting to its forward and backward intermediates (^1^IM3-*S*^+^ and ^1^IM2-*S*), respectively (see SI6†). In terms of the electronic energies from B3LYP/BI geometric optimizations, ^1^TS7-*S* is 3.6 kcal mol^−1^ higher than ^1^IM2-*S*. However, the solvent effect-corrected free energy makes the low transition state disappear. The disappearance of the barrier is reasonable, because dissociation is an entropically favourable process, and the polarization effect of the solvent favours the polar transition state due to the heterolytic dissociation. Essentially, the process from ^1^IM2-*S* to ^1^IM4-*S* is an outer-sphere oxidative addition *via* an S_N_2-type transition state ^1^TS7-*S*. Previously, others and we reported outer-sphere oxidative additions with low barriers.^12,29^ In addition to the outer-sphere oxidative addition, attempts to locate transition states for ^1^[Ni^0^] to undergo Br-transfer with 1-*S* and the inner-sphere oxidative addition were unsuccessful. We reasoned that the Br-transfer could be less favourable, because (i) ^1^[Ni^0^] is a closed-shell species, which disfavours an abstraction process and (ii) the dissociation of ^1^IM2-*S* into ^1^[Ni^0^] and 1-*S* is highly endergonic by 35.2 kcal mol^−1^ (Fig. 1), while the conversion of ^1^IM2-*S* to ^1^IM4-*S* is barrierless and highly exergonic by 29.6 kcal mol^−1^ (Fig. 2). Proceeding forward, ^1^IM4-*S* undergoes hydride transfer with the K_3_PO_4_@[Si]H complex *via*^1^TS8-*S*, giving the nickel(ii) hydride ^1^IM5-*S*. Recall that similar hydride transfer occurs twice in the initiation stage (Fig. 1). The insertion of alkene 2 into the Ni–H bond converts ^1^IM5-*S* to ^1^IM6-*S via*^1^TS9-*S*. Finally, ^1^IM6-*S* undergoes reductive elimination to form the coupling product 3-*R* and recover the nickel(0) species (^1^IM2-*S*). Overall, the coupling is strongly exergonic by 80.5 kcal mol^−1^ with a rate-determining barrier of 26.6 kcal mol^−1^ at the reductive elimination stage.

The precatalyst initiation converts NiBr_2_·glyme to nickel(0) species, ^1^IM2-*R*, ^1^IM2-*S* and ^1^[Ni^0^]·2. We next explored whether these nickel(0) species could be transformed into the coupling products. Starting with these species, Fig. 2 shows our computed coupling pathways. In the following discussion, we used an appendix-*R* or -*S* to designate the chirality of a species inherited from racemic 1.

The *R*-pathway in red in Fig. 2 describes the coupling of 1-*R* with 2 to afford 3-*S*, which is similar to the (*S*)-pathway except for the energetic differences. If the structures of the two pathways could retain their chiralities inherited from 1-*R* or 1-*S*, the two pathways would be parallel rather than competitive, thus resulting in a mixture of 3-*S* and 3-*R*, in disagreement with the enantioconvergence of the reaction. To afford 3-*R* as the major product, the (*R*)-pathway must be able to merge with the (*S*)-pathway for kinetic competition to reset the chirality. Examining the pathways, the chirality resetting can take place at ^1^IM4, ^1^IM5, and ^1^IM6 individually or combinatorially through Ni–C bond cleavage and reformation. Because the reductive elimination of ^1^IM6 results in a product and is the rate-determining step, the chirality resetting at ^1^IM6 is essential. As shown in Fig. 2C, ^1^IM6-*R* and ^1^IM6-*S* can be converted to each other *via* homolytic Ni–C bond cleavage and reformation. Eqn (7) and (8) indicate the two key factors enabling ^1^IM6 to undergo homolytic Ni–C bond cleavage and reformation for the chirality resetting. Radical 1˙ in eqn (7) is more stable than radical 1a˙, indicating that the electron donation of the radical to the C=O π* orbital (*i.e.* p–π conjugation) of 1˙ favours the homolytic Ni–C bond cleavage. Consistently, the alkyl bromides used in the experiments all featured a carbonyl group. The Ni(ii)–C bond cleavage in eqn (8) is thermodynamically more favourable than Pd(ii)–C bond cleavage by 17.0 kcal mol^−1^, indicating that the nickel(i) oxidation state is more accessible than the palladium(i) oxidation state. Thus, the metal identity (*i.e.* nickel) also plays an important role in allowing the homolytic Ni–C bond cleavage.
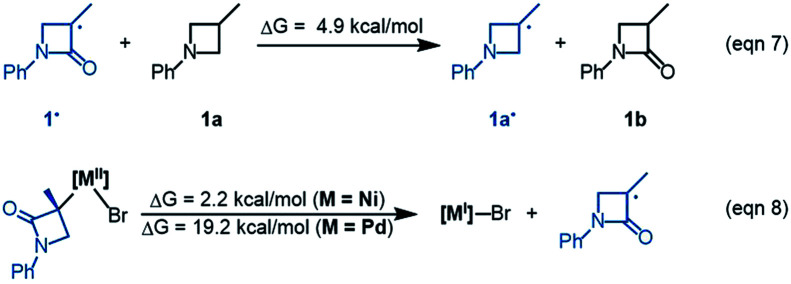


Because ^1^TS12-*R* (Δ*G*^≠^ = −37.1 kcal mol^−1^) is significantly lower than ^1^TS10-*R* (Δ*G*^≠^ = −33.5 kcal mol^−1^), ^1^IM6-*R* would prefer resetting its chirality to convert to ^1^IM6-*S*, rather than undergoing reductive elimination *via*^1^TS10-*R* to give 3-*S*. Fig. 2B exemplifies that the chirality resetting could also take place at ^1^IM4. Note that the slightly lower triplet states ^3^TS11 and ^3^TS12 than ^1^TS11 and ^1^TS12, respectively, would do good rather than harm to the homolytic Ni–C bond cleavage.

In addition to the reductive elimination discussed above, we also examined two alternatives leading ^1^IM6 to the product 3 (see Fig. S12 in SI7†). Specifically, we examined if ^1^IM6 can undergo a two-state reactivity mechanism to afford 3. Because the triplet counterparts of ^1^TS10-*S* and ^1^TS10-*R* are 17.2 and 31.6 kcal mol^−1^ higher than ^1^TS10-*S* and ^1^TS10-*R*, respectively, ^1^IM6 does not possess two-state reactivity. In addition, we considered if ^1^IM6 could first undergo homolytic Ni–C bond cleavage to give the 1˙ radical; then the alkyl–alkyl bond is formed *via* an outer-sphere S_N_2 mechanism. However, the S_N_2 transition states are 20.1 and 13.4 kcal mol^−1^ higher than ^1^TS10-*S* and ^1^TS10-*R*, respectively, excluding the possibility. Moreover, we also examined other conformations of ^1^IM6 and ^1^TS10, but these conformations are higher than those reported in the main text (see Fig. S13 in SI7†).

**Fig. 3:**
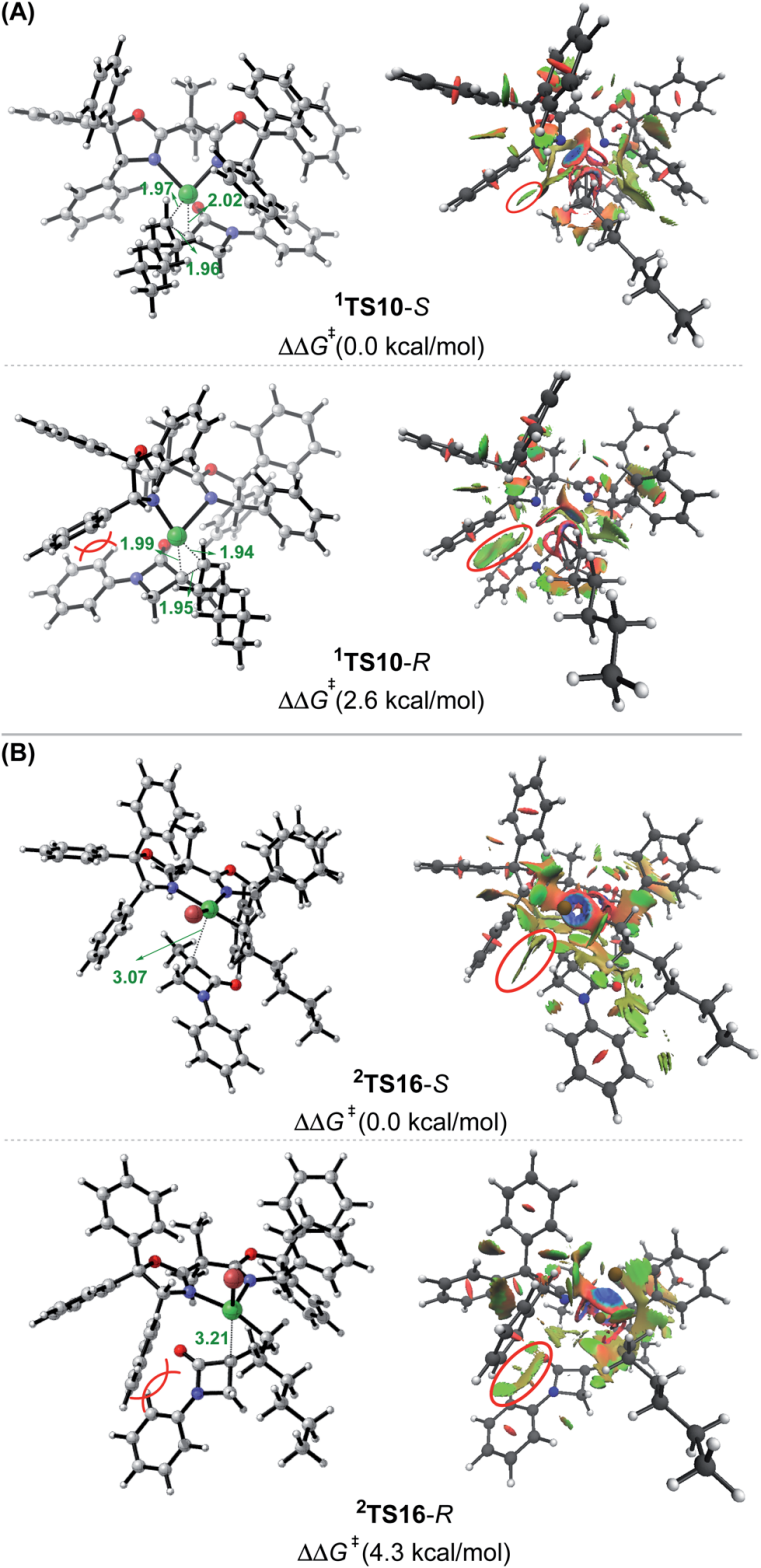
Optimized structures and NCI analysis results for ^1^TS10-*S*, ^1^TS10-*R* (A)*,*^2^TS16-*S* and ^2^TS16-*R* (B) with key bond distances in angstroms and key steric repulsions circled in red.

Merging Fig. 2A with C, the enantioselectivity of the reaction is determined by the energy difference of ^1^TS10-*S* and ^1^TS10-*R*. The energy difference (2.6 kcal mol^−1^), which reasonably agrees with the values at the other two levels (Fig. S2 in SI2†), predicts an ee value of 98 : 2 (3-*R* : 3-*S*), which is in agreement with the experimental value (>99 : 1). To understand how the chiral ligand (*R*,*R*)-L* induces the enantioselective reductive elimination, we performed NCI analyses on ^1^TS10-*S* and ^1^TS10-*R*. As compared in Fig. 3A, obviously, the higher ^1^TS10-*R* than ^1^TS10-*S* can be attributed to the steric repulsion between the phenyl group in alkyl bromide 1 and the phenyl moiety in the chiral (*R*,*R*)-L* ligand.

According to the discussion above, Scheme 2D (bottom left) sketches the catalytic cycle of the coupling reaction with the nickel(0) active species, termed the Ni(0)/Ni(ii) cycle hereafter. After the precatalyst initiation to generate the nickel(0) species, the coupling sequentially proceeds *via* outer-sphere oxidative addition, hydride transfer with the K_3_PO_4_@[Si]H complex, alkene migration insertion, and reductive elimination to form an alkyl–alkyl bond. The enantioconvergence is achieved by resetting the chirality of the reductive elimination precursor *via* homolytic Ni–C bond cleavage and reformation. The enantioselectivity is controlled by the chiral ligand *via* affecting the reductive elimination transition states to favour ^1^TS10-*S* over ^1^TS10-*R*.

### Coupling mechanism with the nickel(i) active species

**Fig. 4:**
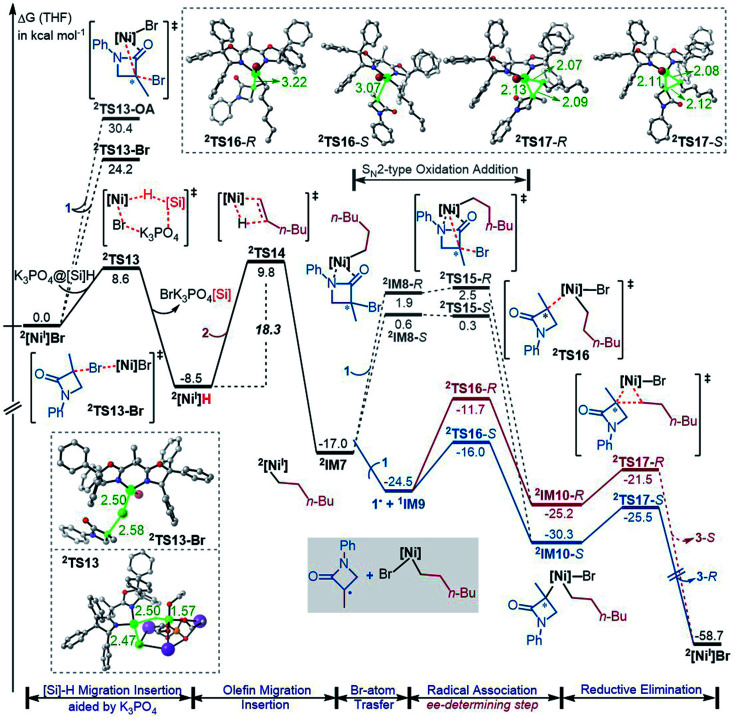
Free energy profiles (in kcal mol^−1^) for the coupling of 1 and 2*via* the Ni(i)/Ni(ii)/Ni(iii) cycle with ^2^[Ni^I^]Br as the active catalyst. The key bond lengths in the key transition states are given in angstroms.

While the Ni(0)/Ni(ii) catalytic cycle well accounts for the enantioselectivity of the reaction, the somewhat high rate-determining barrier (26.6 kcal mol^−1^) raised our concern, because the reaction could occur at 0 °C in spite of the prolonged reaction time (40 h). Regardless of whether the barrier was overestimated or not, we examined the possibility of an alternative catalytic cycle with ^2^[Ni^I^]Br as the active species (Fig. 4). According to the catalytic cycle in Scheme 2C, the first step is to transfer the Br atom of 1 to ^2^[Ni^I^]Br, generating ^3^[Ni^II^]Br_2_ and alkyl radical 1˙ and erasing the chirality of racemic 1 for enantioconvergence. The halogen transfer mechanism was also postulated to account for other nickel-catalysed coupling reactions (*e.g.*Scheme 2A). Two possible reaction modes were examined for the process, including the outer-sphere oxidative addition *via*^2^TS13-OA and the direct Br-transfer *via*^2^TS13-Br. The high barrier of ^2^TS13-OA can be ascribed to the high-valent oxidation state character of nickel(iii) involved in the transition state. Although the barrier height (24.2 kcal mol^−1^) of ^2^TS13-Br is not inaccessible, our finding that K_3_PO_4_@[Si]H can easily undergo hydride transfer with the nickel(ii) species ^1,3^[Ni^II^]Br_2_ or ^1,3^[Ni^II^]BrH species (Fig. 1) encouraged us to inspect if similar hydride transfer could occur between K_3_PO_4_@[Si]H and the nickel(i) species ^2^[Ni^I^]Br. Remarkably, the hydride transfer takes place with a much lower barrier (8.6 kcal mol^−1^, ^2^TS13 relative to ^2^[Ni^I^]Br + K_3_PO_4_@[Si]H), ^2^TS13 being 15.6 kcal mol^−1^ lower than ^2^TS13-Br. Thus, the reaction must proceed *via* hydride transfer, instead of the Br-transfer proposed in Scheme 2C. Note that, because K_3_PO_4_ is highly stabilized by [Si]H by 22.8 kcal mol^−1^, we could exclude the possibility that a separate K_3_PO_4_ can lower ^2^TS13-Br (see Fig. S14 in SI8†).

After ^2^[Ni^I^]H is formed, alkene 2 inserts into the nickel(i) hydride *via*^2^TS14, resulting in ^2^IM7. Subsequently, 1 reacts with ^2^IM7*via* two possible mechanisms. The S_N_2-like outer-sphere oxidative addition mechanism *via*^2^TS15 to give ^2^IM10 has a barrier of about 17.0 kcal mol^−1^. Alternatively, ^2^IM7 extracts the Br atom from 1. Attempts to locate the Br-abstraction transition state failed, but the potential energy surface scan (Fig. S15 in SI9†) indicated that the Br-abstraction could be barrierless. Thus, the nickel(i) species ^2^IM7 prefers extracting the Br atom from 1. Differently, ^1^[Ni^0^] favours outer-sphere oxidative addition to react with 1 (see Fig. 2). We understand the difference as follows. First, ^1^[Ni^0^] is a closed-shell species, while nickel(i) ^2^IM7 is a radical. Intrinsically, ^2^IM7 is a better radical abstractor. Second, the Ni(0)-centre in ^1^[Ni^0^] is more accessible than the Ni(i)-centre in ^2^IM7 for coordination with 1. Consistently, the coordination of 1 to ^2^IM7 is endergonic by more than 17.0 kcal mol^−1^ (see ^2^IM8), while the coordination of 1 to ^1^[Ni^0^] is exergonic by more than 34.0 kcal mol^−1^ (Fig. 1), which is an advantage for outer-sphere oxidative addition. In line with the elucidations, ^2^[Ni^I^]Br also prefers Br-transfer over outer-sphere addition to react with 1, ^2^TS13-Br being 6.2 kcal mol^−1^ lower than ^2^TS13-OA.

The Br-transfer converts 1 to a radical 1˙, which erases the chirality of 1 for enantioconvergence. Afterwards, the radical 1˙ associates with ^1^IM9*via*^2^TS16, resulting in ^2^IM10. Finally, ^2^IM10 undergoes reductive elimination to afford the product 3. Examining the pathways from 1˙ + ^1^IM9 to 3, the enantioselectivity-determining step is the association of 1˙ with ^1^IM9, which is the same as that used in the radical-chain mechanism in Scheme 1A. The energy difference (4.3 kcal mol^−1^) of the two enantiomers of ^2^TS16 reasonably agrees with the experimental ee value (>99 : 1). The NCI analyses indicate that the higher ^2^TS16-*R* than ^2^TS16-*S* is again due to the steric repulsion between the phenyl groups in 1 and a phenyl group in the (*R*,*R*)-L* ligand, as displayed in Fig. 3B.

In their study of cross-coupling enabled by photoredox/nickel dual catalysis, Molander, Kozlowski, Gutierrez and coworkers reported that a nickel(iii) intermediate could also undergo Ni–C bond cleavage and reformation to control enantioselectivity.^13*d*^^2^IM10 in Fig. 4 is also a nickel(iii) species. However, the barriers (^2^TS16) to cleave the Ni–C bond giving 1˙ + ^1^IM9 are substantially higher than the reductive elimination barriers (^2^TS17), excluding ^2^IM10 as a platform to control the enantioselectivity.

Based on the discussion above, we sketch the catalytic cycle with the ^2^[Ni^I^]Br active catalyst in Scheme 2D (right), termed the Ni(i)/Ni(ii)/Ni(iii) cycle hereafter. Compared to the catalytic cycle in Scheme 2C, the reaction sequence in our proposed Ni(i)/Ni(ii)/Ni(iii) cycle is different. This difference is because the K_3_PO_4_@[Si]H complex can reduce ^2^[Ni^I^]Br to ^2^[Ni^I^]H much more easily than the Br-transfer between ^2^[Ni^I^]Br and the electrophile 1. Because of this, there is a difference regarding the species that mediates the transformation of the electrophile 1 into the alkyl radical. In Scheme 2C, the active catalyst ^2^[Ni^I^]Br directly extracts the bromine atom of the electrophile 1, generating the alkyl radical 1˙. In Scheme 2D (right), due to the presence of K_3_PO_4_@[Si]H, ^2^[Ni^I^]Br prefers first reacting with K_3_PO_4_@[Si]H, converting to ^2^[Ni^I^]H, followed by olefin insertion to give ^2^[Ni^I^]-alkyl species. The resultant ^2^[Ni^I^]-alkyl is the mediator to convert the electrophile 1 to the alkyl radical. It should be noted that the process is not only energetically beneficial but also essential for using olefin as an alkyl–alkyl coupling partner, because the processes convert olefin C(sp^2^)=C(sp^2^) to Ni(ii)–C(sp^3^)–C(sp^3^) *via* the sequence from Br–Ni(ii)–alkyl to H–Ni(ii)–alkyl (*via* hydride transfer) to alkyl–Ni(ii)–alkyl (*via* olefin insertion).

In addition to the higher ^2^TS13-Br and ^2^TS13-OA than ^2^TS13, another issue related to the mechanism in Scheme 2C lies in the conversion of the olefin as an alkyl coupling partner. Given that [Ni^II^]Br_2_ could be generated and further converted to [Ni^II^]BrH, the resultant [Ni^II^]BrH would prefer proceeding to the nickel(0) species, because, referring to Fig. 1, the olefin insertion barrier (TS4a) is higher than the K_3_PO_4_-aided [Si]H hydride transfer barrier TS4.

As both the Ni(0)/Ni(ii) and Ni(i)/Ni(ii)/Ni(iii) cycles (Fig. 2 and 4) agree with the observed enantioselectivity, the latter has a rate-determining barrier of 18.3 kcal mol^−1^ lower than that (26.6 kcal mol^−1^) of the former, which is more consistent with the experimental fact that the reaction occurred at 0 °C. Thus, the occurrence of the Ni(i)/Ni(ii)/Ni(iii) cycle relies on whether the ^2^[Ni^I^]Br species could be formed. A common mechanism to generate nickel(i) species is comproportionation of nickel(0) and nickel(ii) species. Referring to Fig. 1, the precatalyst initiation results in nickel(0) species [Ni^0^], thus [Ni^0^] species might undergo comproportionation with nickel(ii) intermediates (*e.g.*[Ni^II^]Br_2_) to give nickel(i) species. However, the comproportionation could be suppressed by the coordination of alkene 2 to the nickel(0) species [Ni^0^], because the coordination is barrierless and highly exergonic by 34.0 kcal mol^−1^. It should be noted that if a nickel(0) species can exist not so stably, comproportionation may occur. Vinyard *et al.* showed that the comproportionation in their catalytic system takes place *via* potential energy surface crossing with low barriers.^30^

**Scheme 3:**
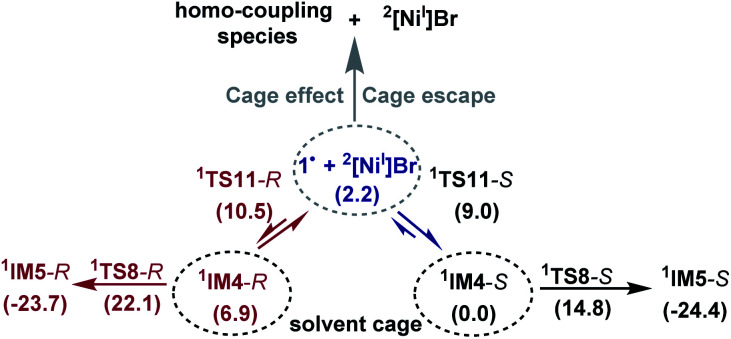
A possible mechanism to form ^2^[Ni^I^]Br active species.

Because the generation of the nickel(i) active catalyst *via* comproportionation could be excluded safely for the present catalytic system, on the basis of the cage effect occurring in free radical polymerization,^31^ we herein proposed a possible alternative to generate ^2^[Ni^I^]Br species. As illustrated in Scheme 3, there is an equilibrium (^1^IM4-*R* ↔ 1˙ + ^2^[Ni^I^]Br ↔ ^1^IM4-*S*) *via* Ni–C bond cleavage and reformation. On the one hand, the equilibrium can shift to ^1^IM5-*S*/^1^IM5-*R* by crossing ^1^TS8-*S*/^1^TS8-*R*. On the other hand, 1˙ + ^2^[Ni^I^]Br may proceed *via* the cage effect, resulting in radical–radical homo-coupling species and ^2^[Ni^I^]Br. Therefore, after forming ^1^IM4, the competition between the hydride transfer *via*^1^TS8 and the cage effect determines the feasibility to generate ^2^[Ni^I^]Br. Note that there is a 5.8 kcal mol^−1^ (the difference between ^1^TS11-*S* and ^1^TS8-*S*) margin for ^1^IM4-*S* to undergo homo-coupling. Experimentally, Fu *et al.* demonstrated that the alkyl radical involved in eqn (9) could escape from the solvent cage to form an out-of-cage cyclized product.^32^ In addition, we studied the experimental control reaction (eqn (10)) in the absence of [Si]H and K_3_PO_4_. The detailed results in SI10† show the possibility.
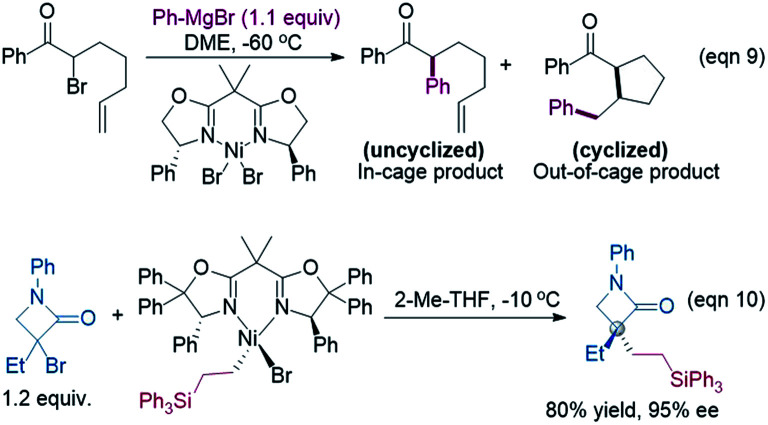


Like ^1^IM4, ^1^IM5 or ^1^IM6 could also follow a similar mechanism to give nickel(i) species (^2^[Ni^I^]H and ^2^[Ni^I^]-alkyl). While it is not certain whether the species could be generated, it is certain that using these species as the active catalysts would not lead to new catalytic cycles, because, as shown in Fig. 4, the species are all involved in the catalytic cycle and are finally converted to ^2^[Ni^I^]Br. In other words, the generations of these nickel(i) species would do more good than harm to the Ni(i)/Ni(ii)/Ni(iii) cycle.

The understanding of the present reaction encouraged us to revisit our previous study of the nickel-catalysed doubly enantioconvergent coupling of racemic alkyl nucleophiles with racemic electrophiles.^12^ Referring to Scheme 2B, the barrier for homolytic Ni–C bond cleavage of the oxidative addition intermediate is 4.1 kcal mol^−1^ lower than the barrier for the attack of the organozinc reagent, thus, it is also possible for the alkyl radical to escape the cage to undergo homo-coupling, giving a nickel(i) species as the active species to start a catalytic cycle similar to that shown in Scheme 2A.

On the basis of our present and previous studies, we proposed that two catalytic cycles (Ni(0)/Ni(ii) and Ni(i)/Ni(ii)/Ni(iii)) with nickel(0) and nickel(i) as the active catalyst, respectively, may operate for the coupling reactions. The preference of a catalytic cycle depends on the competition between the Ni(0)/Ni(ii) cycle and the cage effect to generate a persistent nickel(i) active catalyst to start the Ni(i)/Ni(ii)/Ni(iii) cycle. For the reaction (eqn (4)), because the rate-determining barrier of the Ni(0)/Ni(ii) cycle is low (<16.0 kcal mol^−1^), the Ni(0)/Ni(ii) cycle could operate preferentially. For the present reaction (eqn (6)), the high rate-determining barrier (26.6 kcal mol^−1^) drives the reaction to undergo the Ni(i)/Ni(ii)/Ni(iii) cycle. Notably, both cycles can control the enantioselectivity with similar effects of the chiral ligands.

In our computed model reaction (eqn (6)), the electrophile is a tertiary alkyl bromide 1. Experimentally, secondary alkyl bromides bearing a carbonyl group were also found to be a class of eligible electrophiles.^8^ On the basis of our proposed mechanism, we examined the energetics of the key processes related to ^1^IM4 and ^1^IM6 in the Ni(0)/Ni(ii) cycle and ^2^IM7 in the Ni(i)/Ni(ii)/Ni(iii) cycle, using a secondary alkyl bromide. The detailed results given in the ESI (Fig. S17–S19 in SI11†) show that the secondary alkyl bromide features energetics for these processes comparable with that of 1, explaining why the reaction worked well for the secondary alkyl bromides.

## Conclusions

In summary, we have performed DFT calculations to disclose the mechanisms for the asymmetric alkyl–alkyl bond formation *via* nickel-catalysed reductive enantioconvergent cross-coupling of racemic alkyl bromides with olefins in the presence of hydrosilane and K_3_PO_4_. The study suggests that both nickel(0) and nickel(i)–Br could act as the active catalyst to mediate the reductive coupling. In the case with the nickel(0) active catalyst, the reductive experimental conditions first reduce the nickel(ii) precursor NiBr_2_·glyme to a nickel(0) active species. With the active species, the coupling proceeds *via* a sequence of oxidative addition, K_3_PO_4_-aided hydride transfer, alkene insertion, and reductive elimination. Unlike a conventional two-electron redox catalytic cycle, the nickel(ii) reductive elimination precursor can undergo homolytic Ni–C bond cleavage and reformation to reset the chirality of the coupling carbon to a preferred structure for enantioselective reductive elimination. In the case with the nickel(i)–Br active catalyst, because the K_3_PO_4_-aided hydride transfer from [Si]H to ^2^[Ni^I^]Br is much more favourable than the Br-transfer from alkyl bromide to ^2^[Ni^I^]Br, ^2^[Ni^I^]Br is converted to a ^2^[Ni^I^]H species, followed by olefin insertion giving a ^2^[Ni^I^]-alkyl species which serves as a chain-carrying radical to perform the coupling *via* the radical-chain mechanism. On the basis of the reported experimental and our computed results, we proposed a cage effect enabled pathway for switching the Ni(0)/Ni(ii) cycle to a more favourable Ni(i)/Ni(ii)/Ni(iii) cycle. The pathway proceeds *via* homolytic cleavage of the Ni–C bond of nickel(ii) species (*e.g.* Br–Ni(ii)–alkyl) in the Ni(0)/Ni(ii) cycle, followed by cage effect enabled homo-coupling, leading to a persistent ^2^[Ni^I^]Br species for the Ni(i)/Ni(ii)/Ni(iii) cycle. In both catalytic cycles, the transformation of olefin to an alkyl–alkyl coupling partner is realized by converting a Ni–Br bond to Ni–H *via* hydride transfer, to a Ni–alkyl bond *via* olefin insertion. Expectedly, these insights could offer a guide for developing new enantioconvergent couplings.

## Data availability

Data for this work, including free energies, Cartesian coordinates of all optimized structures and additional results, are presented in the ESI.†

## Author contributions

C.-S. Z. and Z.-X. W. conceived the project. C.-S. Z. performed the calculations with the contributions from B.-B. Z. and L. Z. C.-S. Z., X.-Y. C. and Z.-X. W. analyzed the results and composed the manuscript with input from all the authors.

## Conflicts of interest

The authors declare no competing financial interests.

## Supplementary Material

SC-013-D1SC05605K-s001
