# Supplementary material for: DFT insight into asymmetric alkyl–alkyl bond formation via nickel-catalysed enantioconvergent reductive coupling of racemic electrophiles with olefins
Source: Chem Sci. 2022 Feb 25;13(13):3728–39. doi: 10.1039/d1sc05605k (PMC8966719; doi:10.1039/d1sc05605k)
Supplement: SC-013-D1SC05605K-s001 [file SC-013-D1SC05605K-s001.pdf]

Electronic Supplementary Material (ESI) for Chemical Science.
This journal is © The Royal Society of Chemistry 2022

Supplementary Information

DFT Insight into Asymmetric Alkyl-Alkyl Bond Formation via Nickel-Catalysed Enantioconvergent Reductive Coupling of Racemic Electrophiles with Olefins

Chao-Shen Zhang,[a] Bei-Bei Zhang,[a] Liang Zhong,[a] Xiang-Yu Chen[*,a] Zhi-Xiang Wang[*,a]

[a] School of Chemical Sciences, University of the Chinese Academy of Sciences, Beijing 100049, China

E-mail: chenxiangyu20@ucas.ac.cn;
zxwang@ucas.ac.cn.

Table of Contents

SI1. Results related to spin contaminations 2

SI2. Results for calculation method validation 4

SI3. Energetics for ligand exchange process 7

SI4. Binding energies of K_3PO_4 with [Si]H, solvent and substrates 8

SI5. Experimentally reported reductive hydrofunctionalizations of alkenes and proposed mechanism 9

SI6. IRC results to veify the correctness of important transition states 11

SI7. Additional results for leading ^{1}IM6 to the product 3 14

SI8. Results to consider the effect of K_3PO_4 on ^{2}TS13-Br 16

SI9. Results for potential energy surface (PES) scan 17

SI10. The radical-chain mechanism in the absence of [Si]H and K_3PO_4 18

SI11. Energetic results of the key processes with a secondary alkyl bromide as the electrophile 20

SI12. References 23

SI13. Cartesian coordinates in Å, SCF energies and free energies (in a.u.) 24

SI1. Results related to spin contaminations

Attentions were paid to the spin contaminations of open shell species. **Table S1** lists the $\langle S^2 \rangle$ values of the species. As compared, $\langle S^2 \rangle$ values after annihilation are generally close to the standard values, except for those (highlighted in blue) involved in the rebound processes, are somewhat large. To estimate the energetic influences of the spin contaminations, we used the following formula[1] to correct the energies of the open shell singlet, where S_0 and S_1 are the annihilated spin contamination values of the open-shell singlet and triplet states, respectively. E_0 and E_1 are the energies of calculated open-shell singlet and triplet at the same geometry.

$$E \approx \frac{S_1^2 E_0 - S_0^2 E_1}{S_1^2 - S_0^2}$$

Table S2 shows the energy corrections (**ΔE**) due to spin contaminations for these species (e.g. ^{1}**TS11**-*R*,^{1}**TS11**-*S*, ^{1}**TS12**-*R* and ^{1}**TS12**-*S*) with relatively large spin contaminations. It can be found that the energy corrections are small. Thus, the spin contaminations would not affect our proposed mechanism. In the main text, we used the energies without spin contamination corrections.

Table S1. **$\langle S^2 \rangle$** values of the structures in the main text.

	**Spin contaminant [S*(S+1)]**		**Spin state**
	**Before annihilation**	**After annihilation**	
**$^{1}NiBr_2$·glyme**	0.9814	0.0269	OS
**$^{3}NiBr_2$·glyme**	2.0089	2.0000	Triplet
**$^{1}[Ni^{II}]Br_2$**	0.7054	0.0161	OS
**$^{3}[Ni^{II}]Br_2$**	2.0104	2.0001	Triplet
**^{1}TS1**	1.0010	0.0497	OS
**^{3}TS1**	2.0085	2.0000	Triplet
**^{1}TS2**	0.6266	0.0087	OS
**^{3}TS2**	2.0077	2.0000	Triplet
**^{1}TS3**	0.9954	0.0214	OS
**^{3}TS3**	2.0067	2.0000	Triplet
**$^{1}[Ni^{II}]BrH$**	0.0000	0.0000	CS
**$^{3}[Ni^{II}]BrH$**	2.0111	2.0001	Triplet
**^{1}TS4**	0.6985	0.0182	OS
**^{3}TS4**	2.0124	2.0001	Triplet
**$^{1}[Ni^{II}]H_2$**	0.0000	0.0000	CS
**$^{3}[Ni^{II}]H_2$**	2.0137	2.0001	Triplet
**^{1}TS5**	0.0000	0.0000	CS
**^{3}TS5**	2.0163	2.0002	Triplet
**^{1}IM1**	0.0000	0.0000	CS
**^{3}IM1**	2.0153	2.0001	Triplet
**^{1}TS6**	0.2556	0.0112	OS
**^{3}TS6**	0.0135	0.0001	Triplet
**$^{1}[Ni^{0}]$**	0.0000	0.0000	CS

**^{3}[Ni^{0}]**	2.0145	2.0001	Triplet
**^{1}IM2-*R***	0.0000	0.0000	CS
**^{1}IM2-*S***	0.0000	0.0000	CS
**^{1}TS7-*R***	0.5535	0.0305	OS
**^{1}TS7-*S***	0.5418	0.0291	OS
**^{1}IM3-*R*$^{+}$**	0.1256	0.0005	OS
**^{1}IM3-*S*$^{+}$**	0.0928	0.0003	OS
**^{1}IM4-*R***	0.9519	0.0465	OS
**^{1}IM4-*S***	0.4912	0.0090	OS
**^{1}TS8-*R***	0.7837	0.0176	OS
**^{1}TS8-*S***	0.7589	0.0246	OS
**^{1}IM5-*R***	0.0000	0.0000	CS
**^{1}IM5-*S***	0.0000	0.0000	CS
**^{1}TS9-*R***	0.0000	0.0000	CS
**^{1}TS9-*S***	0.0000	0.0000	CS
**^{1}IM6-*R***	0.0000	0.0000	CS
**^{1}IM6-*S***	0.0000	0.0000	CS
**^{1}TS10-*R***	0.0000	0.0000	CS
**^{1}TS10-*S***	0.0000	0.0000	CS
**^{1}TS11-*R***	0.9712	0.1340	OS
**^{1}TS11-*S***	1.0055	0.1437	OS
**^{2}[Ni^{I}]Br**	0.7643	0.7502	doublet
**^{1}TS12-*R***	1.0141	0.2209	OS
**^{1}TS12-*S***	1.0206	0.2145	OS
**^{2}IM7**	0.7672	0.7502	doublet

Table S2. Spin contamination-corrected energies of the transition states involved in the rebound process.

**TS$_s$**	**E$_0$** (a.u.)	**S$_0$**	**E$_1$**(a.u.)	**S$_1$**	**E**(a.u.)	**ΔE=E-E$_0$** (kcal/mol)
**^{1}TS11-*R***	-2697.89707849	0.1340	-2697.901572	2.0005	-2697.897058	0.01
**^{1}TS11-*S***	-2697.89940638	0.1437	-2697.896423	2.0005	-2697.899422	-0.01
**^{1}TS12-*R***	-2920.79059200	0.2209	-2920.790980	2.0009	-2920.790587	0.00
**^{1}TS12-*S***	-2920.78895000	0.2145	-2920.790517	2.0009	-2920.788932	0.01

SI2. Results for calculation method validation

The species involved in our study are quite large, containing up to 145 atoms. To reduce the computation cost, we computed the reaction pathways at M06(SMD, THF)/BSII//UB3LYP(gas)/BSI level. To validate the reliability of the protocol, we performed the following calibration calculations.

(A) We compared the results of the precatalyst (**^{1,3}[Ni^{II}]Br$_2$**) calculated at M06(SMD, THF)/BSII//UB3LYP(gas)/BSI and M06(SMD, THF)/BSII//UB3LYP-D3BJ(SMD, THF)/BSI levels, respectively. As compared in **Fig. S1**, the results at the two levels of calculations agree with each other well.

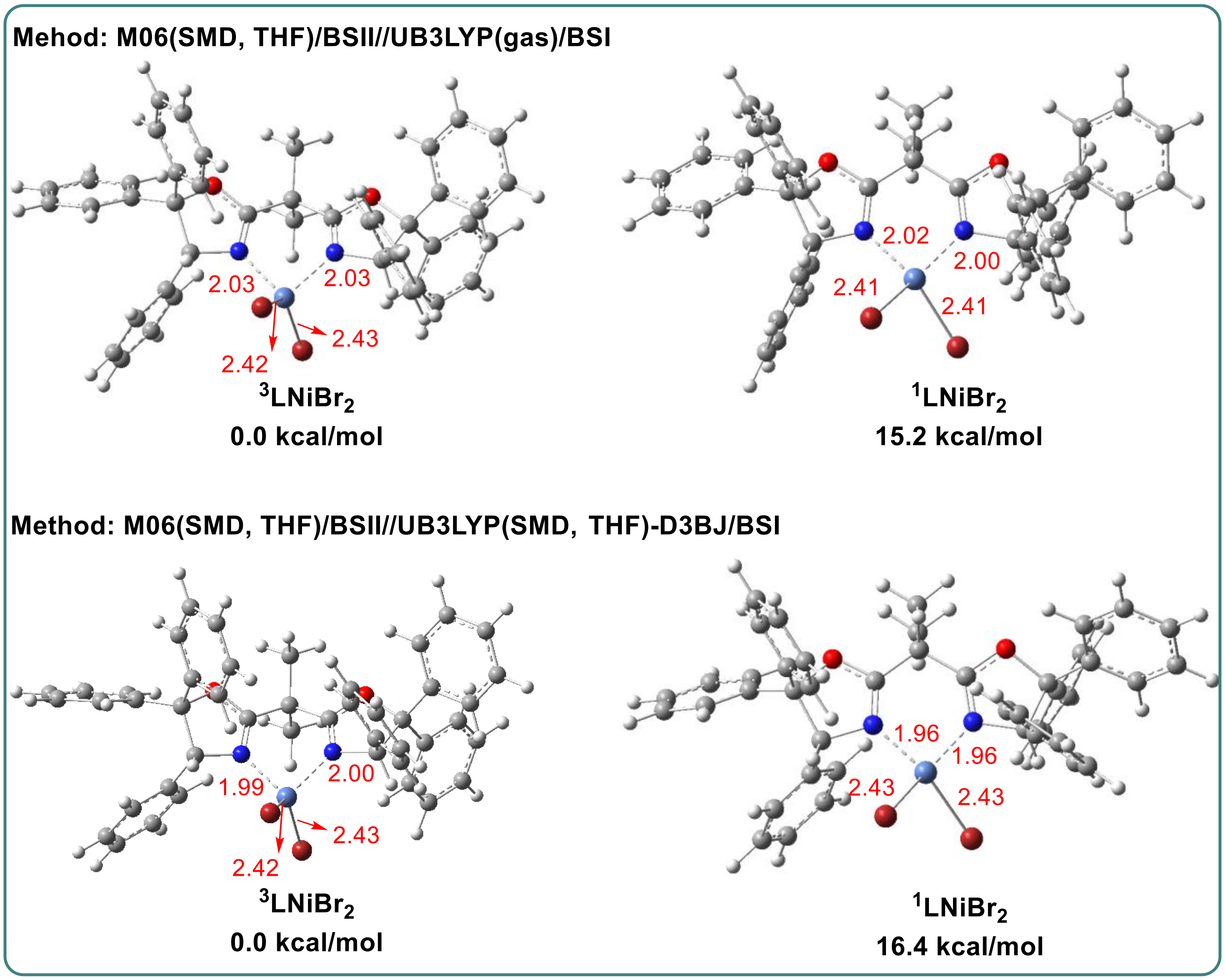

Fig. S1. Comparing the results of precatalyst (**^{1,3}[Ni^{II}]Br$_2$**) calculated at the two levels of calculations. The key bond lengths are given in angstroms.

(B) **^{1}TS10**-*R*/**^{1}TS10**-*S* and **^{1}TS12**-*R*/**^{1}TS12**-*S* are key transition states involved in our proposed mechanism (Fig. 2 in the main text). Using the transition states, we calculated their relative energies at other two levels (M06(SMD, THF)/BSII//UB3LYP-D3BJ(SMD, THF)/BSI (Level 2) and UB3LYP-D3BJ(SMD, THF)/BSII//UB3LYP-D3BJ(SMD, THF)/BSI (Level 3)). Comparing the results in **Fig. S2**, we have the following observations: (i) The optimized structures at UB3LYP-D3BJ(SMD, THF)/BSI level are in good

agreement with those at B3LYP (gas)/BSI level. (ii) Like level 1 used in the main text, Levels 2 and 3 also predicted ^{1}**TS10**-*S* to be lower than ^{1}**TS10**-*R*, which is in agreement with the experimentally observed enantioselectivity. (iii) Comparing to level 1, levels 2 and 3 predicted ^{1}**TS12**-*R*/^{1}**TS12**-*S* to be more significantly lower than ^{1}**TS10**-*R*/^{1}**TS10**-*S*. This indicates that the Ni-C bond cleavage can take place kinetically more favorable, which favors our proposed mechanism.

	M06(SMD, THF)/BSII// B3LYP (gas)/BSI (Level 1)	M06(SMD, THF)/ BSII//B3LYP-D3BJ(SMD, THF)/BSI (Level 2)	B3LYP-D3BJ(SMD, THF)/BSII //B3LYP-D3BJ(SMD, THF)/BSI (Level 3)
^{1}**TS10**-*S*	0.0	0.0	0.0
^{1}**TS10**-*R*	2.6	2.6	3.4
^{1}**TS12**-*R*	-1.0	-3.0	-8.9
^{1}**TS12**-*S*	-2.3	-3.5	-7.8

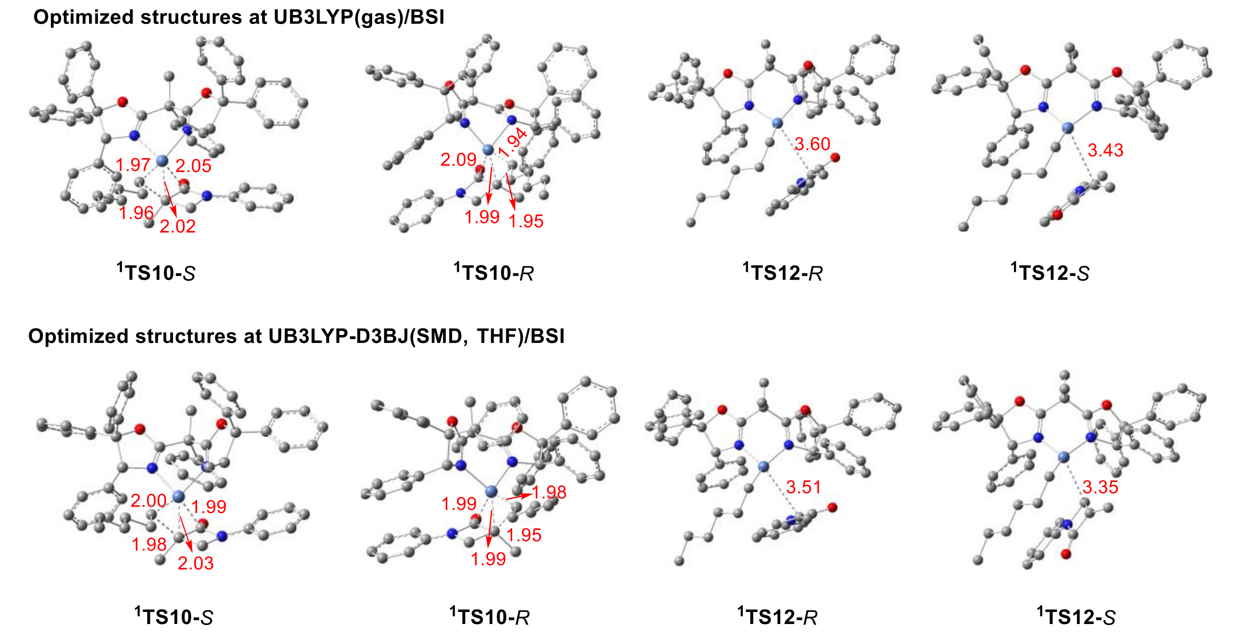

Fig. S2. Comparing the results of ^{1}**TS10**-*R*/^{1}**TS10**-*S* and ^{1}**TS12**-*R*/^{1}**TS12**-*S* at three levels of calculations.

(C) Using ^{1}**TS10**-*R*/^{1}**TS10**-*S* and ^{1}**TS12**-*R*/^{1}**TS12**-*S,* we further calculated the relative energies of the transition states calculated with DFT functionals having different percentage of Hartree-Fock exchange. Compared to B3LYP with 20% HF exchange, the examined *w*B97XD and M11 functionals have 25%, and 43% HF exchange, respectively. Comparing the results in **Fig. S3**, we have the following observations: (i) Like M06(SMD, THF)/BSII// B3LYP (gas)/BSI used in the main text, *w*B97XD(SMD, THF)/BSII//*w*B97XD (gas)/BSI and M11(SMD, THF)/BSII//M11(gas)/BSI also predicted ^{1}**TS10**-*S* to be lower than ^{1}**TS10**-*R*, which is in agreement with the experimentally observed enantioselectivity. (ii) Comparing to M06(SMD, THF)/BSII//B3LYP(gas)/BSI level, *w*B97XD(SMD, THF)/BSII//*w*B97XD (gas)/BSI and M11(SMD, THF)/BSII//M11(gas)/BSI predicted ^{1}**TS12**-*R*/^{1}**TS12**-*S* to be more significantly lower than ^{1}**TS10**-*R*/^{1}**TS10**-*S*. This indicates that the Ni-C bond cleavage can take place more kinetically favorable, which favors our proposed mechanism.

	M06(SMD, THF)/BSII// B3LYP (gas)/BSI	*w*B97XD(SMD, THF)/ BSII// *w*B97XD (gas)/BSI	M11(SMD, THF)/ BSII// M11(gas)/BSI
**^{1}TS10**-*S*	0.0	0.0	0.0
**^{1}TS10**-*R*	2.6	3.2	3.6
**^{1}TS12**-*R*	-1.0	-6.8	-4.2
**^{1}TS12**-*S*	-2.3	-2.7	-5.4

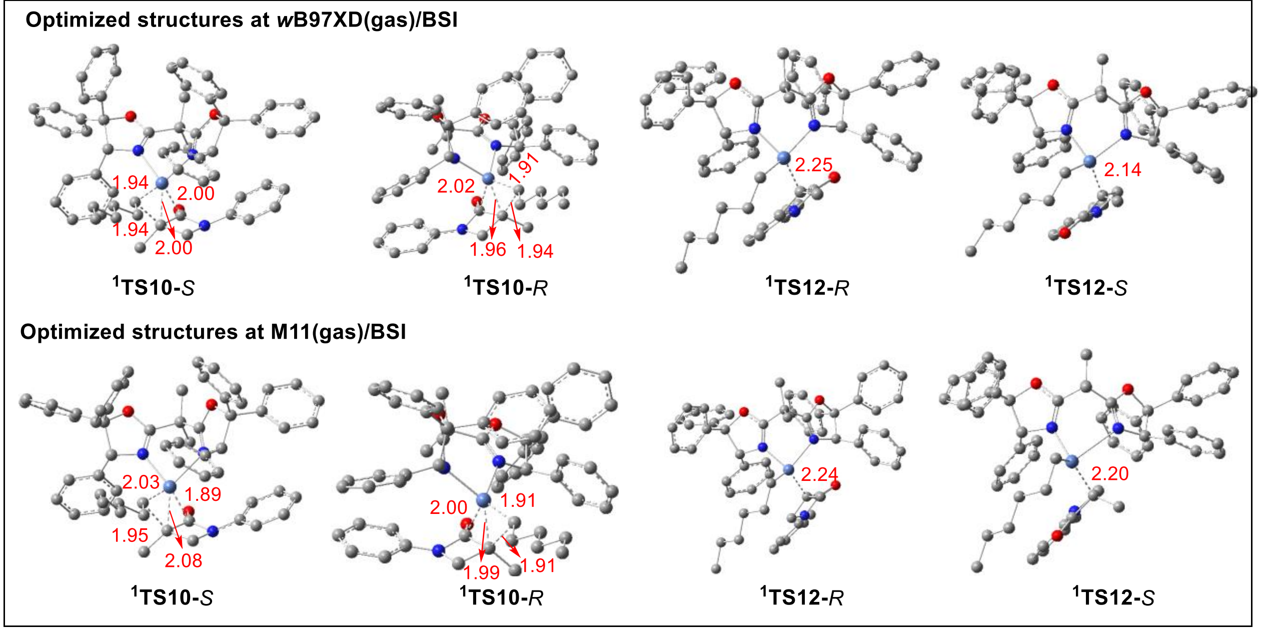

Fig. S3. Comparing the results of **^{1}TS10**-*R*/**^{1}TS10**-*S* and **^{1}TS12**-*R*/**^{1}TS12**-*S* with DFT functionals having different percentage of Hartree-Fock exchange.

SI3. Energetics for ligand exchange process

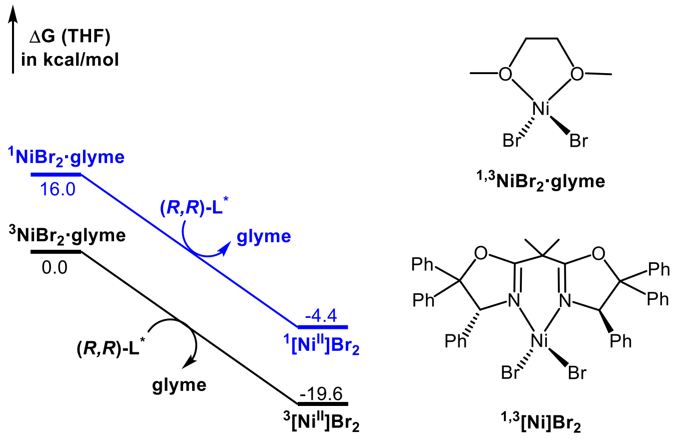

Fig. S4. Energetic results for ligand (*R*,*R*)-L* exchange with $NiBr_2$•glyme precursor.

SI4. Binding energies of K_3PO_4 with [Si]H, solvent and substrates

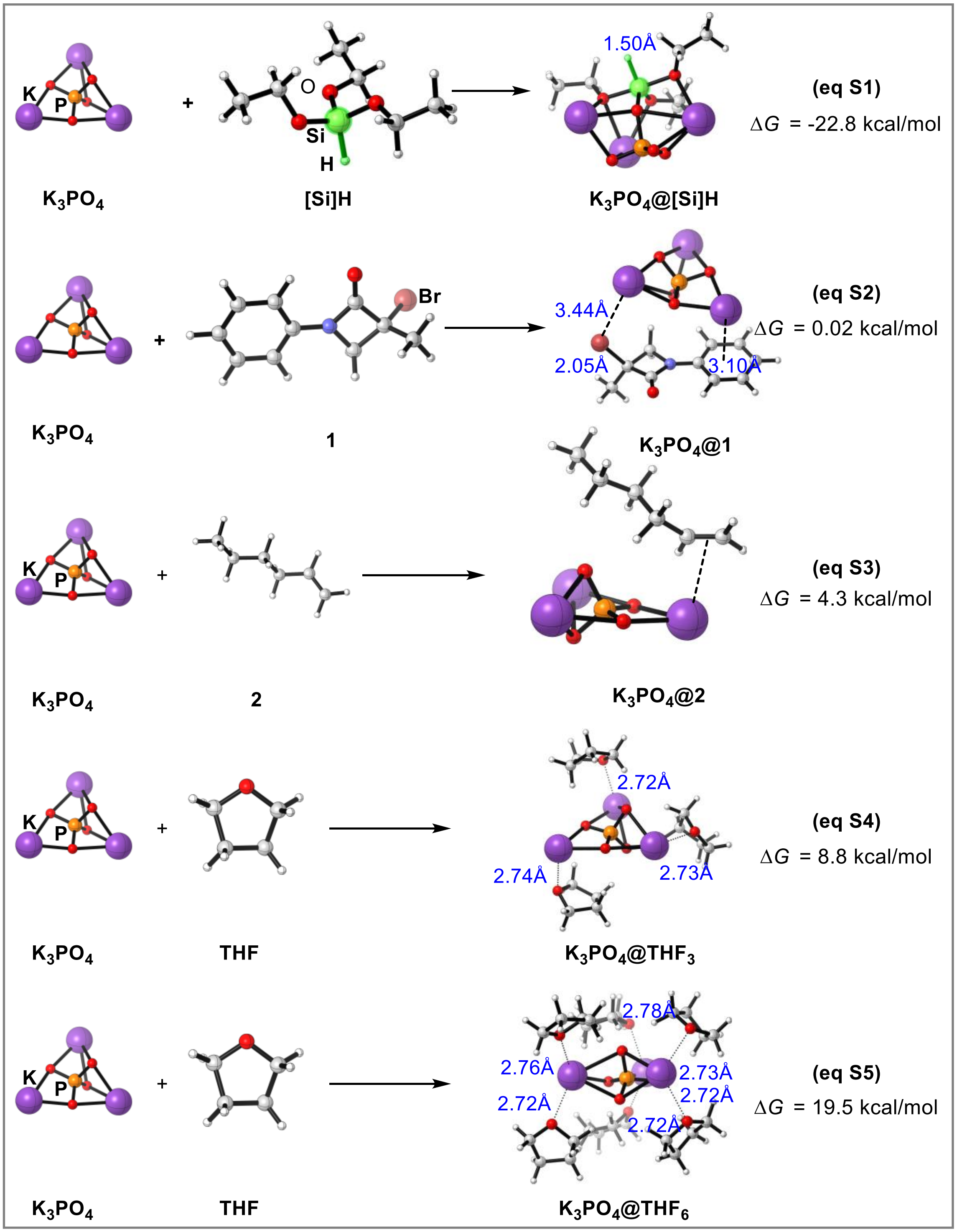

Fig. S5. Binding energies of the complexes between K_3PO_4 with **1** or **2**, or H[Si] or THF. Key bond lengths in the structures are given in angstroms.

SI5. Experimentally reported reductive hydrofunctionalizations of alkenes and proposed mechanism

Over the past several years, several multicomponent catalytic systems involving nickel (II) precursors such as $NiBr_2$·glyme, hydrosilane, and bases such as K_3PO_4 or LiF have been developed to perform reductive hydrofunctionalization of alkenes.[2-5] In those experimental studies, nickel hydrides were postulated to be the active catalysts (see **Fig. S6**). However, these proposals have not been verified experimentally or computationally. To examine whether our proposed precatalyst initiation mechanism applies for those reactions, we calculated the initiation mechanism of eq S9. The energetic results in **Fig. S7** indicates that the nickel (II) precatalyst (**$^3LNiCl_2$**) can also be converted to nickel (0) species.

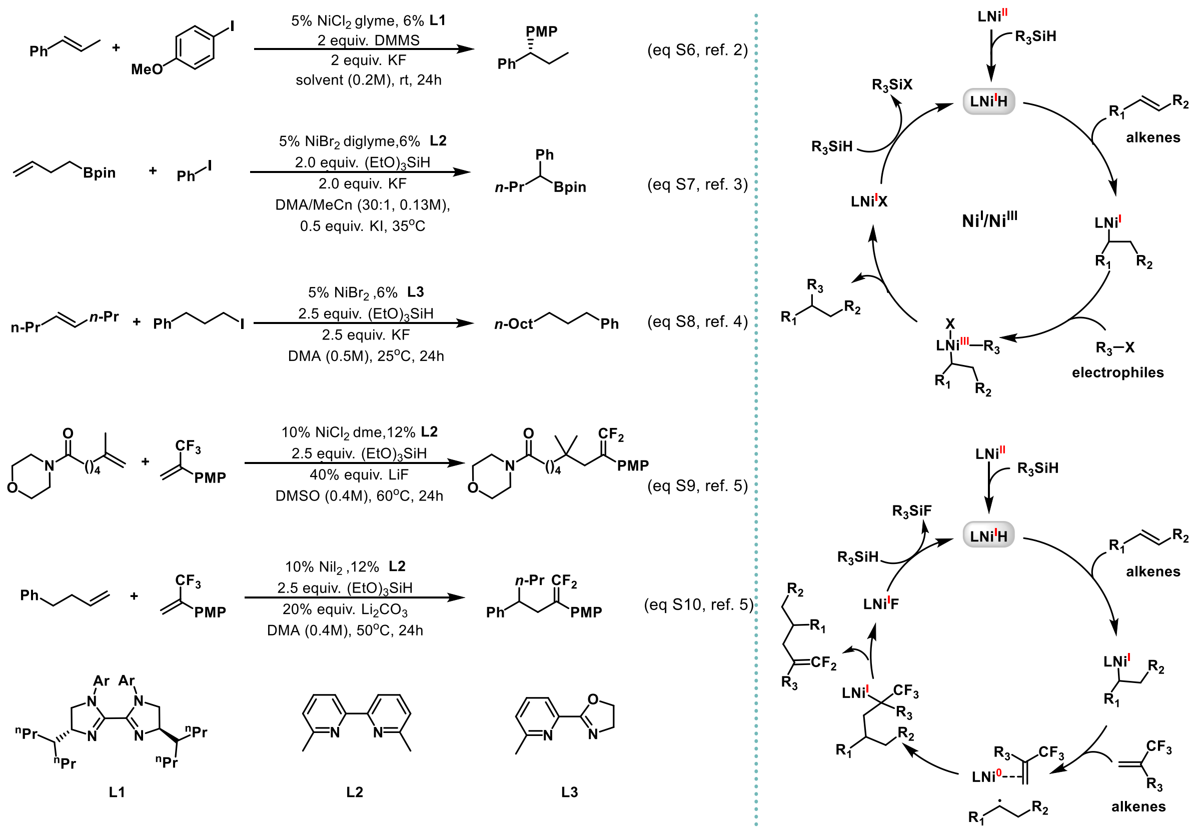

Fig. S6. Reductive hydrofunctionalizations of alkenes and the proposed catalytic cycles with nickel (I) hydrides as the active catalysts.

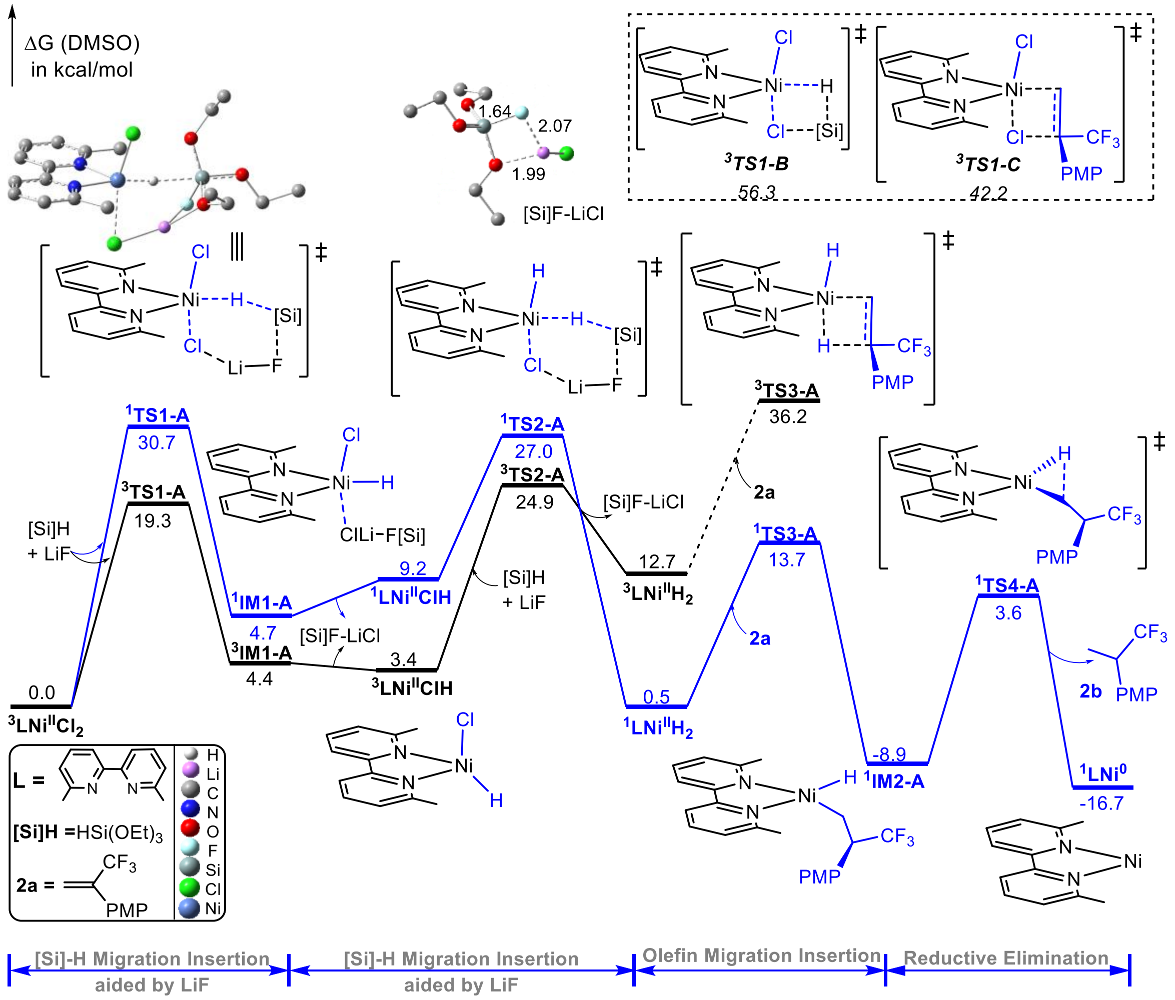

Fig. S7. Free energy profile for precatalyst initiation to give nickel (0) species, using eq S9 in **Fig. S6** as an example.

SI6. IRC results to veify the correctness of important transition states

To verify a transition state correctly connecting to the desired forward and backward intermediates, we performed IRC (intrinsic reaction coordinate) calculations for the important transition states (^{1}**TS7**-*R*/ ^{1}**TS7**-*S*, and ^{1}**TS12**-*S*). The IRC results in **Fig. S8**-**S11** indicate that these transition states correctly connect to their forward and backward intermediates.

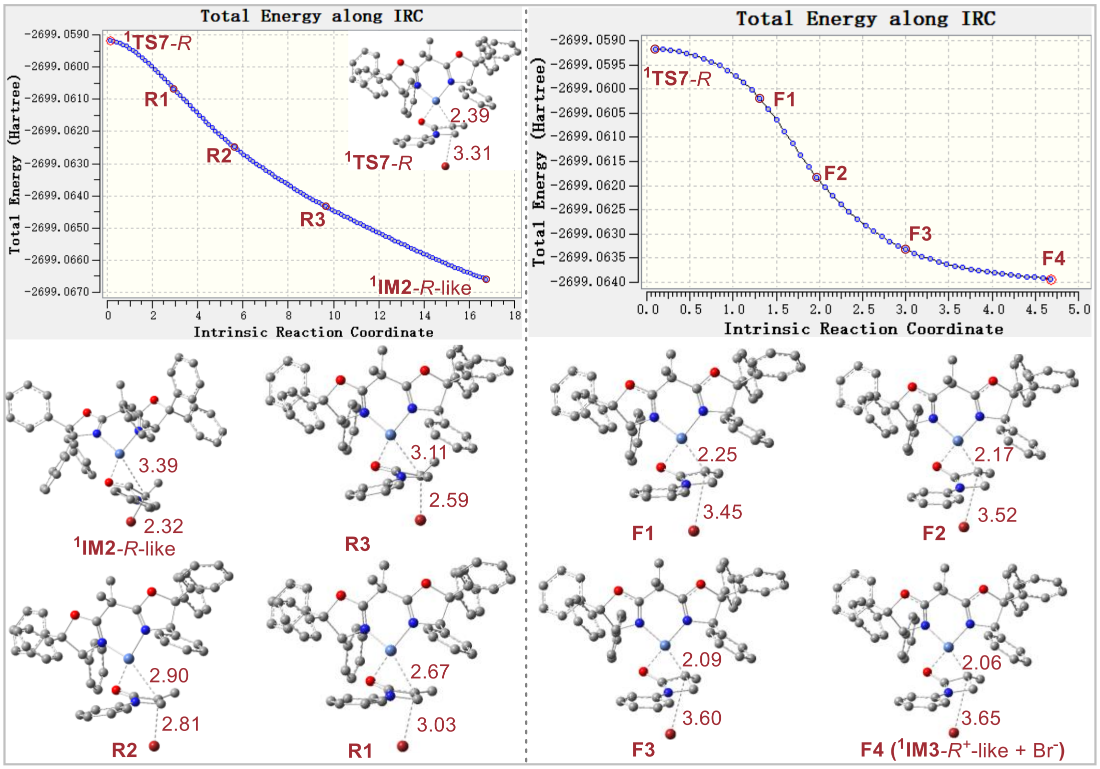

Fig. S8. IRC results showing that ^{1}**TS7**-*R* connects to ^{1}**IM3**-R^{+} in the forward direction and ^{1}**IM2**-*R* in the backward direction. Note that the geometric optimization starting at ^{1}**IM2**-*R*-like structure, obtained after 120 IRC step, converged to ^{1}**IM2**-*R*.

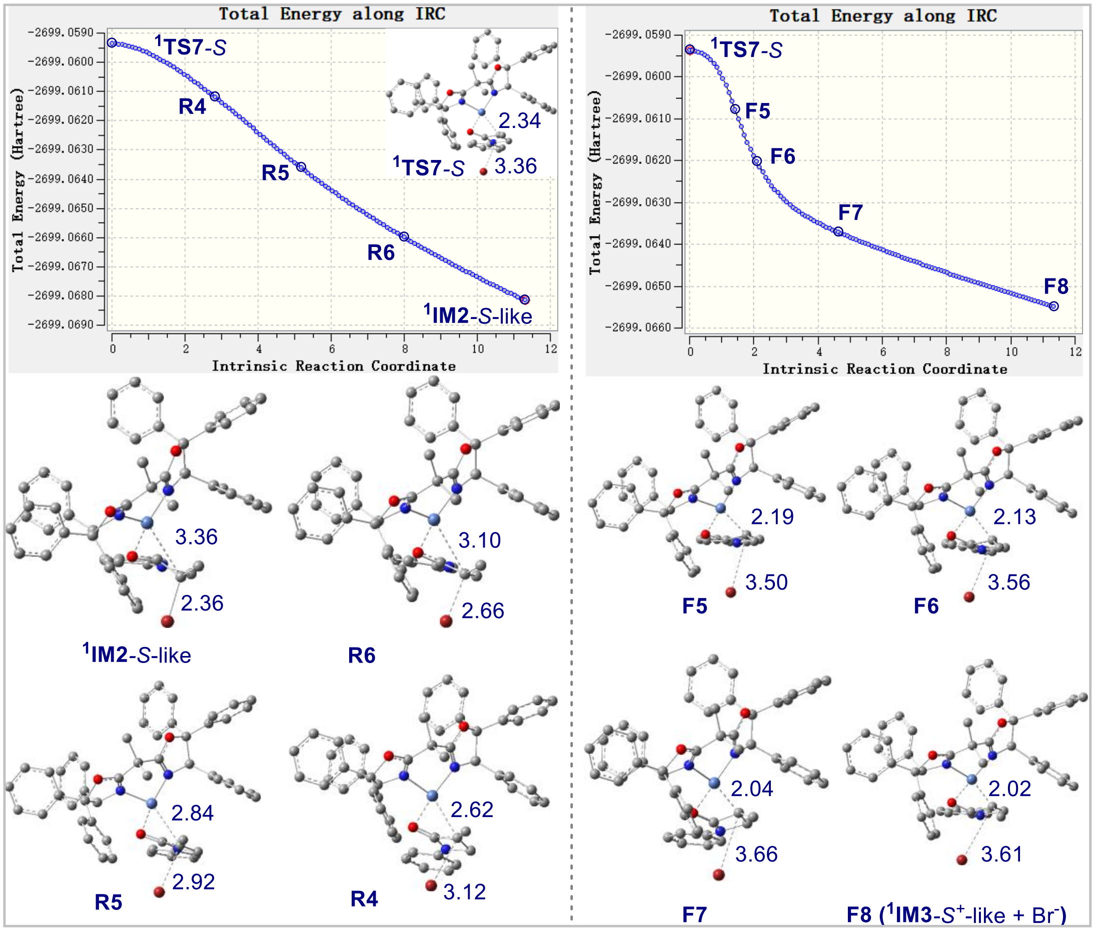

Fig. S9. IRC results showing that **[1]TS7-*S*** connect to **[1]IM3-*S*[+]** in the forward direction and **[1]IM2-*S*** in the backward direction. Note that the geometric optimization starting at **[1]IM2-*S***-like structure, obtained after 120 IRC step, converged to **[1]IM2-*S***.

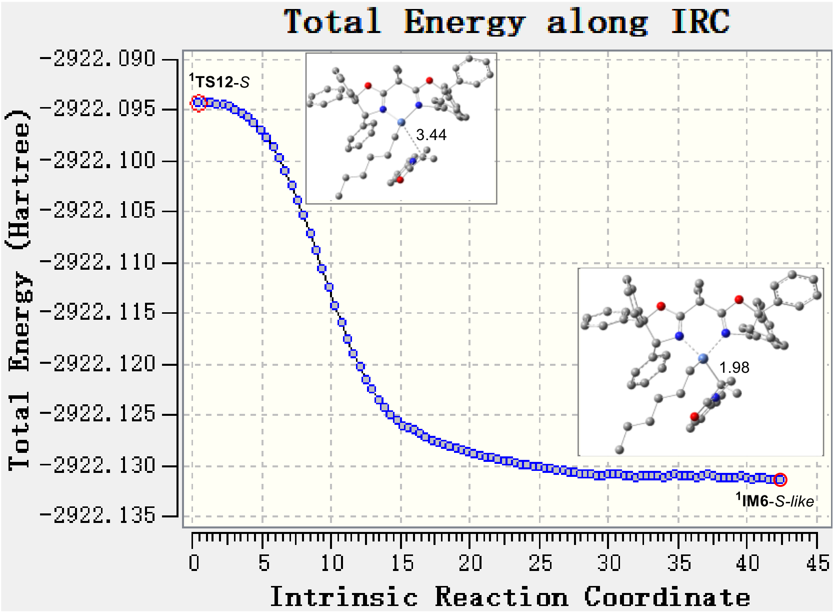

Fig. S10. IRC results showing that [1]**TS12-*S*** connects to [1]**IM6-*S*** in the forward direction. Note that the geometric optimization starting at [1]**IM6-*S***-like structure, obtained after 100 IRC steps, converged to [1]**IM6-*S***.

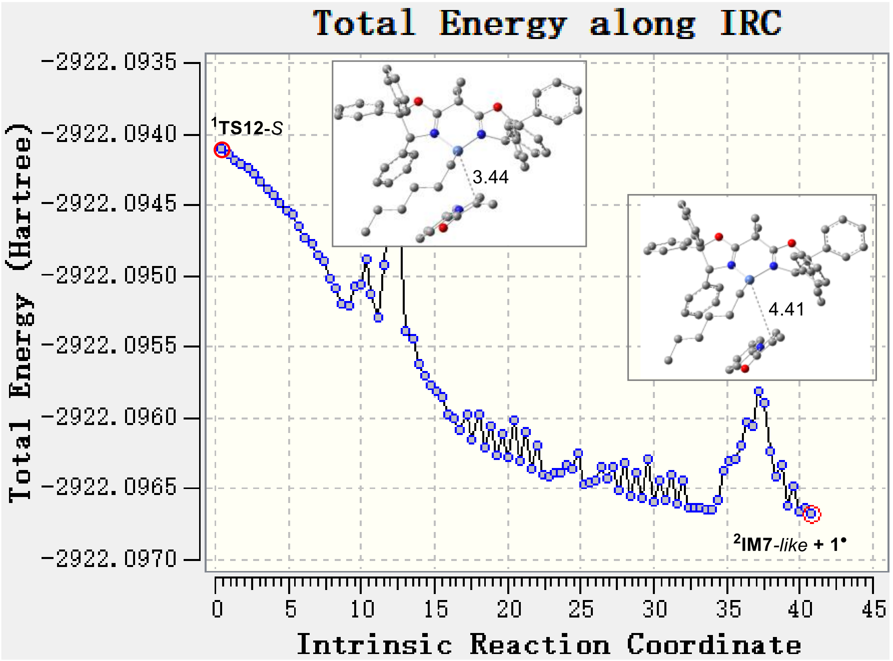

Fig. S11. IRC results showing that [1]**TS12-*S*** connects to [2]**IM7 + 1**$^{\bullet}$ in the backward direction. Note that the geometric optimization starting at [2]**IM7-**like structure, obtained after 100 IRC steps, converged to [2]**IM7**.

SI7. Additional results for leading ¹IM6 to the product 3

In addition to the results for leading **¹IM6** to **3**, reported in the main text, we computed the triplet **³TS10**-*S* and **³TS10**-*R* and the transition states (**³TS10**-*S'* and **³TS10**-*R'*) for S_N2-like outer-sphere mechanism to form alkyl-alkyl bond. As shown in **Fig. S12**, these transition states are much higher than their counterparts (**¹TS10**-*S* and **¹TS10**-*R*), excluding these possibilities.

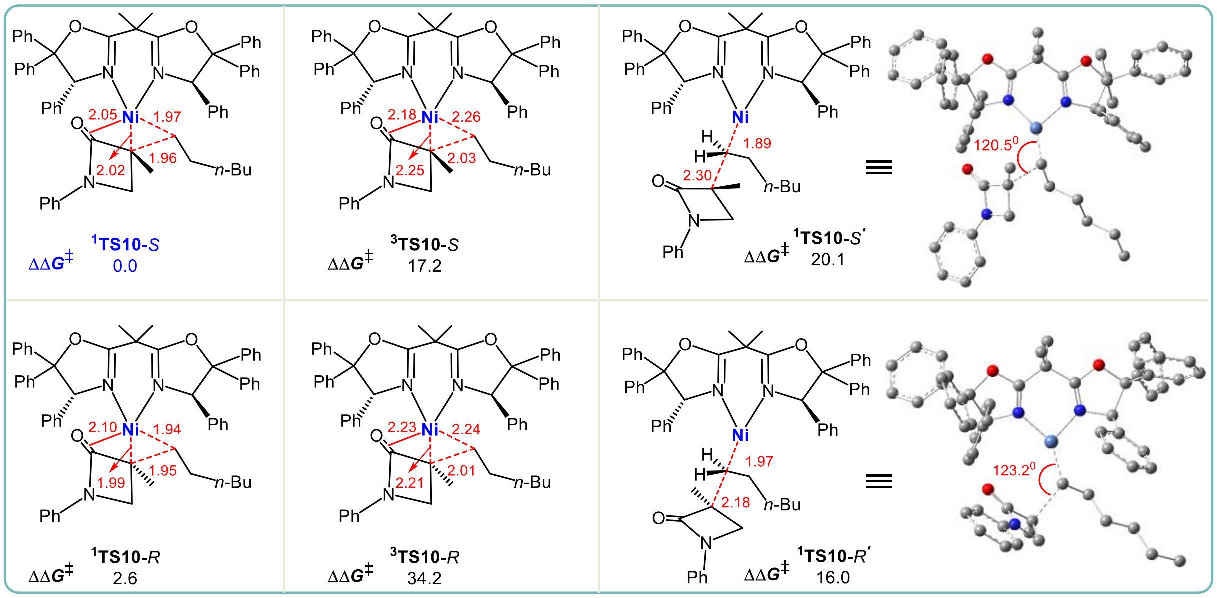

Fig. S12. Transition states alternative to **¹TS10**-*S* and **¹TS10**-*R*. Key bond lengths in red are given in angstroms.

We also considered some conformations of **¹IM6** manually. Examining the structure of **¹IM6-*S***, the LNi moiety is rigid without freedoms. Referring to **¹IM6-*S***, however, the Ph group of the substrate **1** may point out of the paper. We considered this scenario and obtained **¹IM6A**-*S* and **¹IM6B**-*S* which are higher than **¹IM6-*S***. Examining **¹TS10**-*R*/**¹TS10**-*S*, the transition states feature coordination between nickel and carbonyl group, which restricts the conformation of the transition states. We tried to examine other conformations of **¹TS10** manually by removing the coordination, however such transition states could not be located, indicating the importance of coordination of the carbonyl group.

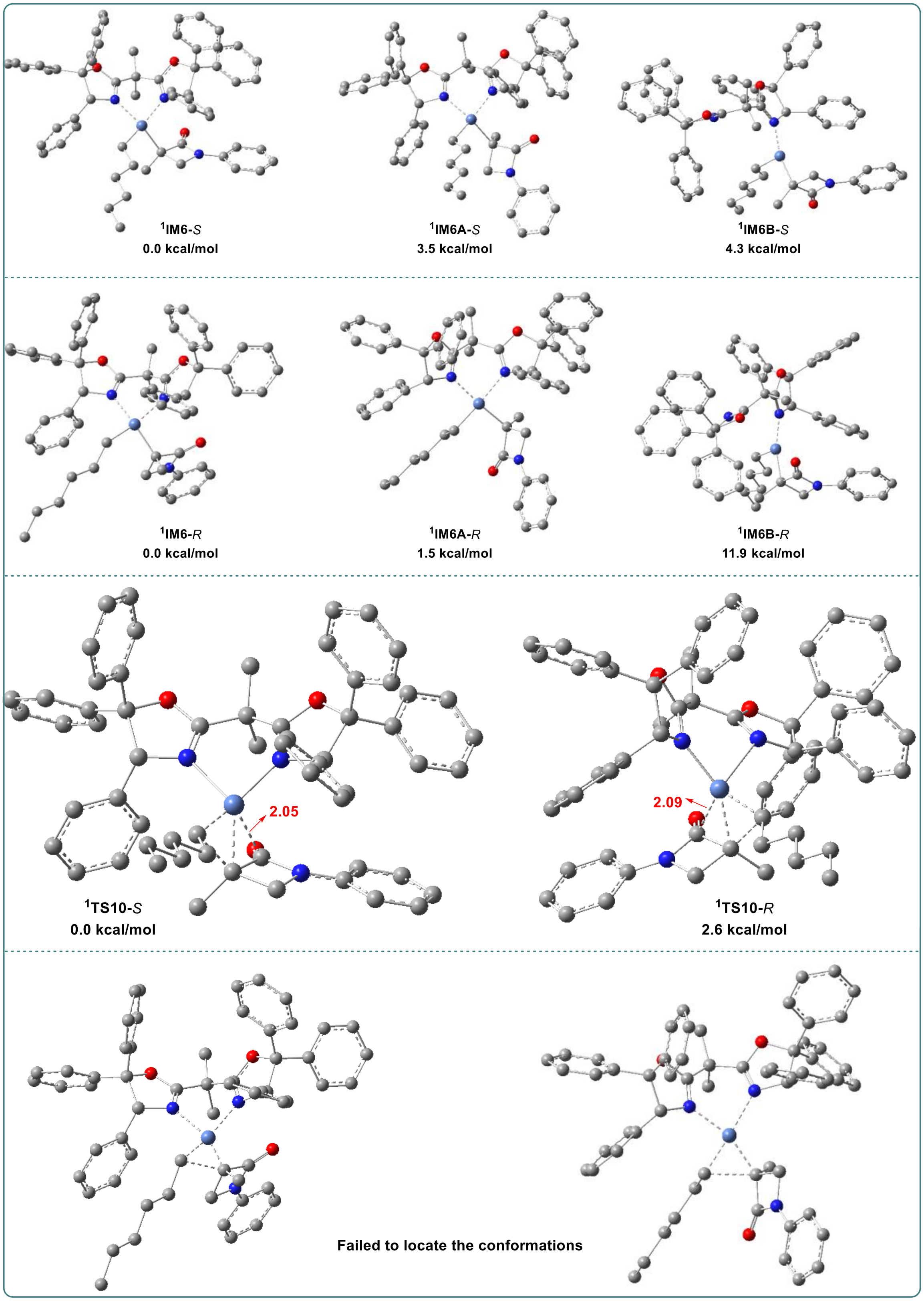

Fig. S13. Other conformations of ^{1}**IM6**-*S*/^{1}**IM6**-*R* intermediates. The initial structures for optimizing the structures of ^{1}**TS10**-*S*/^{1}**TS10**-*R*.

SI8. Results to consider the effect of K_3PO_4 on ^{2}TS13-Br

We considered whether the additive K_3PO_4 could lower the barrier of **^{2}TS13-Br** and located a Br-transfer transition state (i.e. **K-^{2}TS13-Br**) involving K_3PO_4 as a mediator. Relative to separate **^{2}[Ni^I]Br** and K_3PO_4, **K-^{2}TS13-Br** has a free energy of 13.1 kcal/mol, which is lower than **^{2}TS13-Br** ($\Delta G^{\neq}$=24.2 kcal/mol) but still higher than **^{2}TS13** ($\Delta G^{\neq}$=8.6 kcal/mol). Note that K_3PO_4 is highly stabilized by [Si]H by 22.8 kcal/mol (eq S1). If taking the stabilization energy into account and using **^{2}[Ni^I]Br** + K_3PO_4@[Si]H complex as energy reference, **K-^{2}TS13-Br** has a relative energy of 35.9 kcal/mol (13.1 + 22.8), which is much higher **^{2}TS13**. Thus, K_3PO_4 cannot actually facilitate the Br-transfer process.

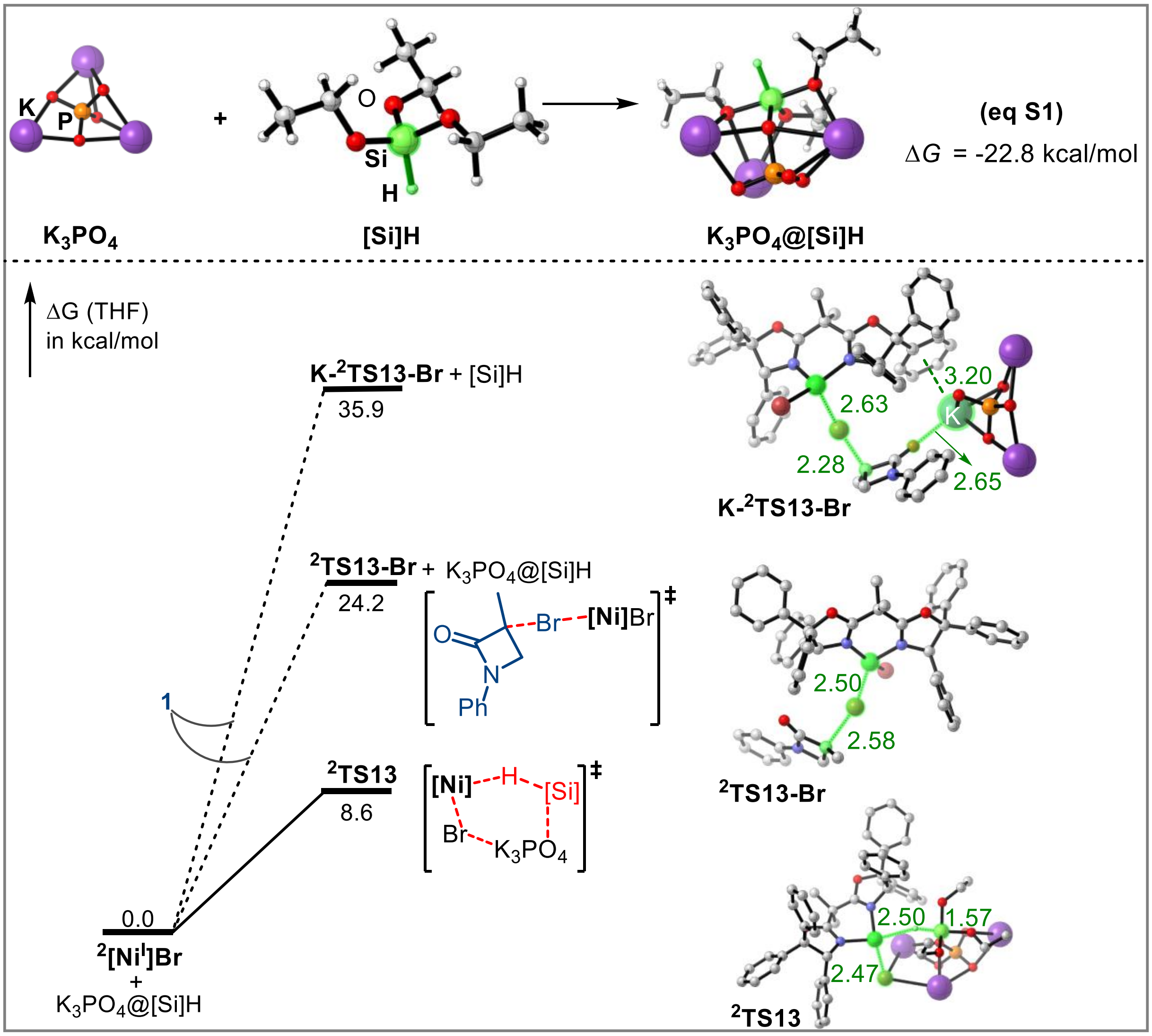

Fig. S14. Comparing the energetics for the effect of K_3PO_4 to **^{2}TS13-Br**.

SI9. Results for potential energy surface (PES) scan

We were not able to locate the transition states for transferring the Br atom of electrophile **1** to ^{2}**IM7**, thus we scanned the potential energy surface (PES) of the process (^{2}**IM7** + **1** to ^{1}**IM9** + **1**$^{\cdot}$). As illustrated in **Fig. S15**, the PES scan started from ^{2}**IM7** + **1** (namely **P1**) and the Ni···Br bond as the reaction coordinate with an interval of 0.05 angstroms. The energy change from **P2** to **P3** indicate that, as Br gets closer to Ni center of ^{2}**IM7**, the Ni–Br bond forms and C(sp^{3})–Br bond breaks without crossing a barrier. In addition, the geometric optimization starting at the point (**P4**) led to separate ^{1}**IM9** and **1**$^{\cdot}$. These results indicate that the Br atom of **1** can easily transfer to Ni center of ^{2}**IM7** without a barrier, giving ^{1}**IM9** + **1**$^{\cdot}$ and explain why the Br-transfer transition state could not be located.

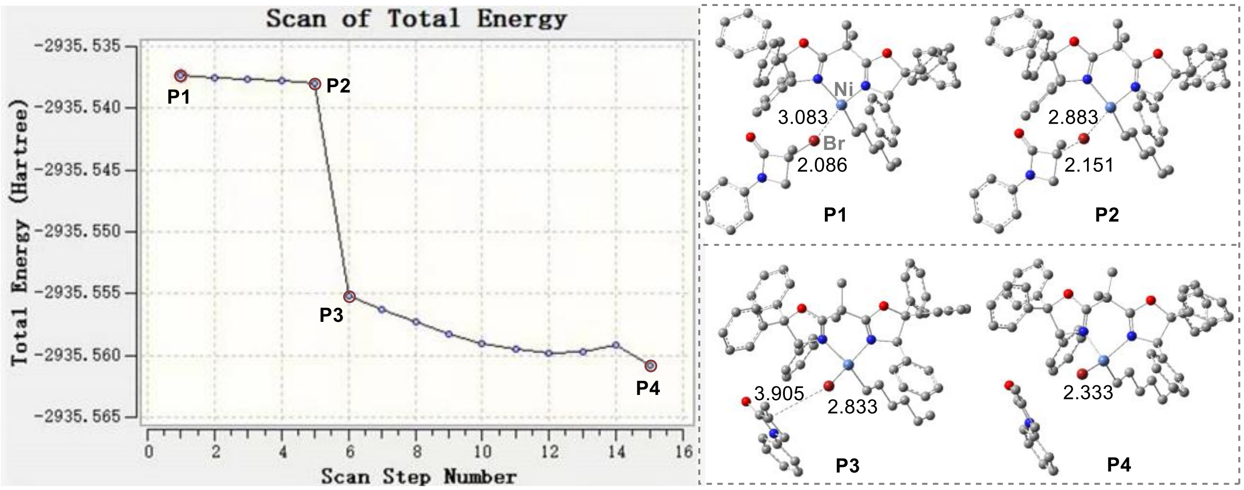

Fig. S15. Results for potential energy surface (PES) scan from ^{2}**IM7** + **1** to ^{1}**IM9** + **1**$^{\cdot}$. Values are key bond lengths in angstroms. Trivial H atoms are omitted for clarity.

SI10. The radical-chain mechanism in the absence of [Si]H and K_3PO_4

To see if our proposed cage-escape enabled mechanism to generate nickel (I) is viable, we studied the stoichiometric reaction (eq S11) reported in reference,[6] using the truncated **B** to model the nickel (II) species **A** for saving computational cost.

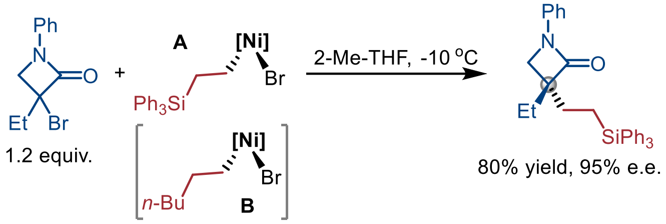

(eq S11)

Experimentally, it was proposed that the reaction takes place catalytically, using an *in-situ* generated nickel (I) species (**^{2}[Ni^I]Br**) as the active catalyst. **^{2}[Ni^I]Br** species is generated by comproportionation between the nickel (II) species **A** and the nicke (0) species generated from reductive elimination of **A**. On the basis of the mechanism, we computed the thermodynamics of the reductive elimination of **B**. The reductive elimination was predicted to be highly endergonic by 59.0 kcal/mol, safely ruling out the comproportionation mechanism (**Fig. S16(A**)). In the following, we proposed two alternatives to generate **^{2}[Ni^I]Br**. (I) as described by **Fig. S16(A**), species **B** first undergoes homolytic Ni-C bond cleavage with a barrier of 12.8 kcal/mol, giving **^{2}[Ni^I]Br** and alkyl radical. Then the cage-escaped alkyl radical couples with another cage-escaped alkyl radical generated from the homolytic Ni-C bond cleavage of **B**, giving the homo-coupling product **5**. The homo-coupling (2***B** → 2*Ni(I)-Br + 2*^{n}BuCH$_2$CH$_2^{\cdot}$ → 2***[Ni^I)Br** + ^{n}BuCH$_2$CH$_2$-CH$_2$CH$_2$^{n}Bu(**5**) is exergonic by 28.6 kcal/mol. The energetics indicates that, as far as the alkyl radical ^{n}BuCH$_2$CH$_2^{\cdot}$ is able to escape the solvent cage, the mechanism could take place, giving **^{2}[Ni^I]Br** species as a persistently existing active catalyst. (II) As described by **Fig. S16(B**), species **B** first undergoes homolytic Ni-C bond cleavage with a barrier of 12.8 kcal/mol, giving **^{2}[Ni^I]Br** and alkyl radical. Then the alkyl radical escapes from the solvent cage to associate with another **B** by crossing a barrier of 25.0 kcal/mol (**^{2}TS19**), followed by reductive elimination via **^{2}TS20** to afford the homo-coupling product **5** and **^{2}[Ni^I]Br**. Although the barrier is somewhat high, we considered it to a viable pathway to generate **^{2}[Ni^I]Br** species. Because the comproportionation mechanism is highly unlikely, we proposed the two alternatives to be viable mechanisms for the generation of **^{2}[Ni^I]Br** species.

Using **^{2}[Ni^I]Br** as the active species, **Fig. S16(C)** shows the energy profile for the reaction of **1** and **B**. First, **1** transfers its Br atom to **^{2}[Ni^I]Br** via a barrier of 24.2 kcal/mol (**^{2}TS13-Br**), giving radical

1$^{\cdot}$ and ^{3}**[Ni^{II}]Br$_2$**. The radical **1**$^{\cdot}$ then associates with **B** by crossing the transition states ^{2}**TS16**, followed by reductive elimination via ^{2}**TS17** to afford **3**. The radical association with **B** is the enantioselectivity-determining step and the energy difference (4.3 kcal/mol) between **TS16**-*R* than **TS16**-*S* are in reasonable agreement with the experimentally observed enantioselectivity.

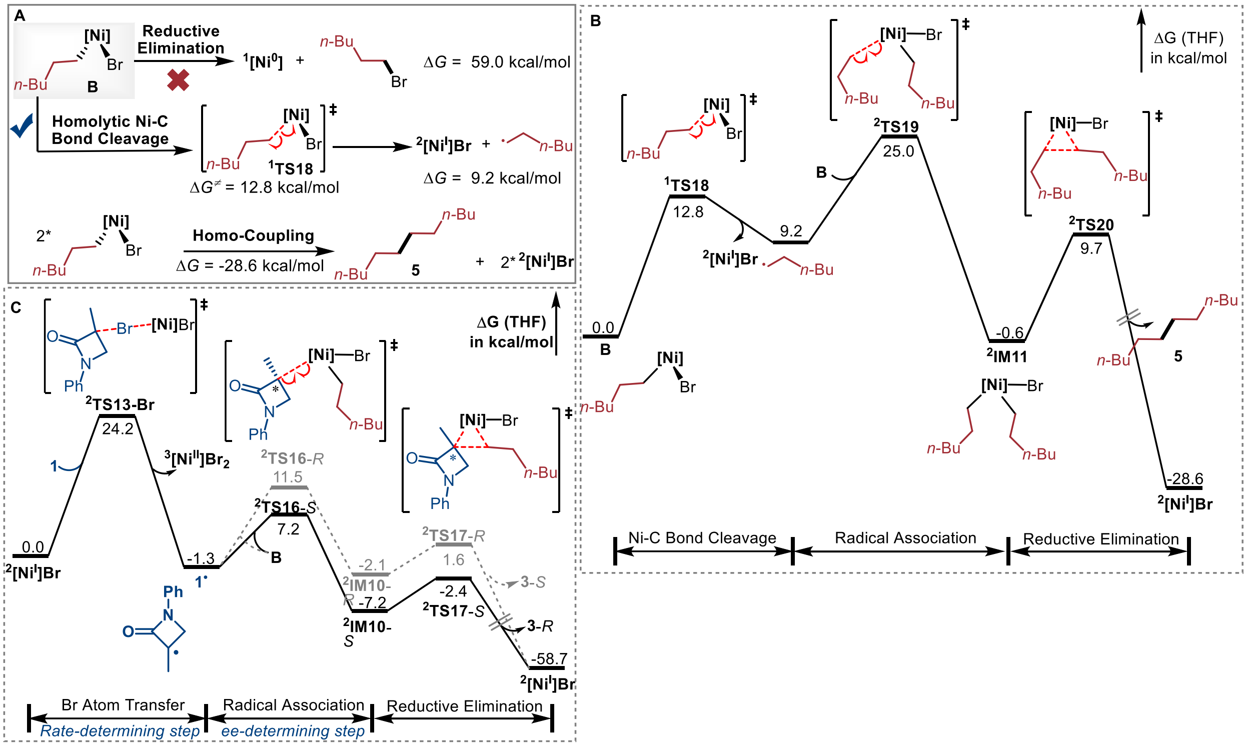

Fig. S16. Energetic results (in kcal/mol) for the two viable mechanisms to generate nickel (I) species ^{2}**[Ni^{I}]Br** (**A** and **B**). (C) Energy profiles for the coupling reaction of eq S11 with ^{2}**[Ni^{I}]Br** as the active catalyst.

SI11. Energetic results of the key processes with a secondary alkyl bromide as the electrophile

In our computed model reaction eq 6 in the main text, the electrophile is a tertiary alkyl bromide **1**. Experimentally, secondary alkyl bromides bearing a carbonyl group were also found to be a class of eligible electrophiles.[6] On the basis of our proposed mechanism, we examined the energetics of the key processes related to **^{1}IM4** and **^{1}IM6** in Ni(0)/Ni(II) cycle and **^{2}IM7** in Ni(I)/Ni(II)/Ni(III) cycle, using a secondary alkyl bromide **1c** in eq S12.

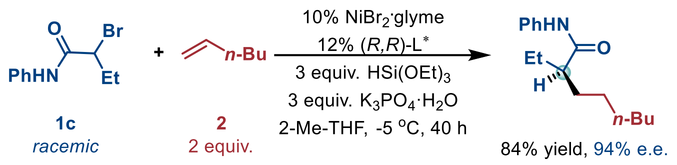

(eq S12)

Because **^{1}IM4** is a key intermediate for generating Ni(I)-Br species via cage effect, we calculated the energetics of the processes starting from **^{1}IM4^{1c}** (Fig. S17). As compared, the energy difference (14.2 kcal/mol) between **^{1}IM4^{1c}** and **^{1}TS8^{1c}** is comparable with that (14.8 kcal/mol) between **^{1}IM4** and **^{1}TS8**. Thus, **^{1}IM4^{1c}** and **^{1}IM4** should behave similarly in terms of the competition between giving Ni(I)-Br via cage effect and undergoing hydride transfer.

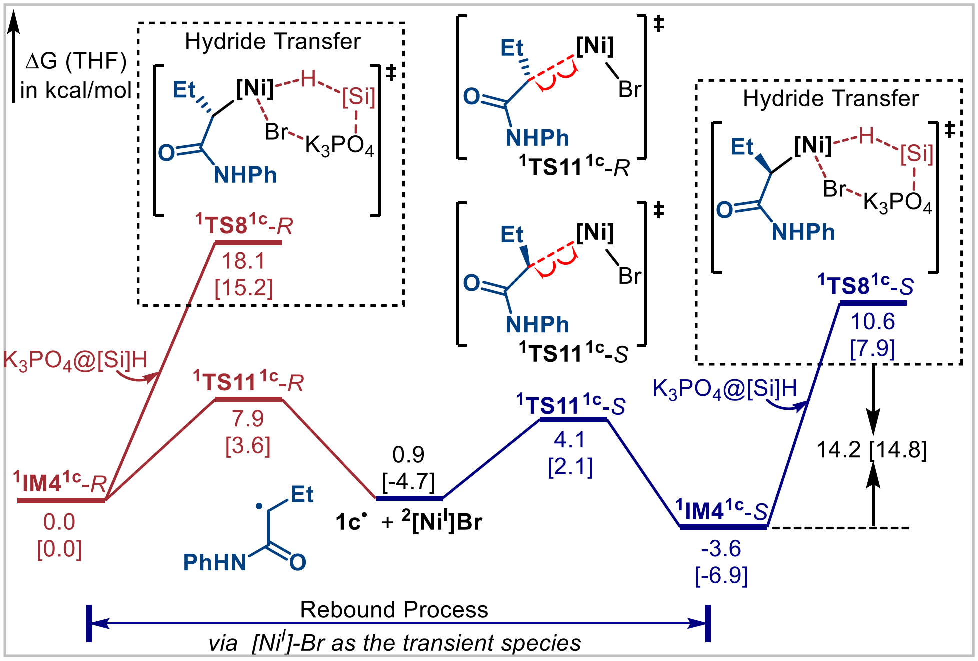

Fig. S17. Comparing the energetic results (in kcal/mol) of the processes of **^{1}IM4^{1c}** and **^{1}IM4**. The values in the square brackets are the corresponding values of the tertiary alkyl bromide (**1**).

We next considered the processes related to the reductive elimination intermediates (e.g. **^{1}IM6**). The results in Fig. S18 indicate that **^{1}IM6^{1c}** is more difficult than **^{1}IM6** to undergo reductive elimination, because the barrier (28.5 kcal/mol) of the former is higher than that (26.6 kcal/mol) of the latter. According to the results, we reason that the Ni(I) cycle is more preferable for secondary alkyl bromide **1c** than for tertiary alkyl bromide **1**.

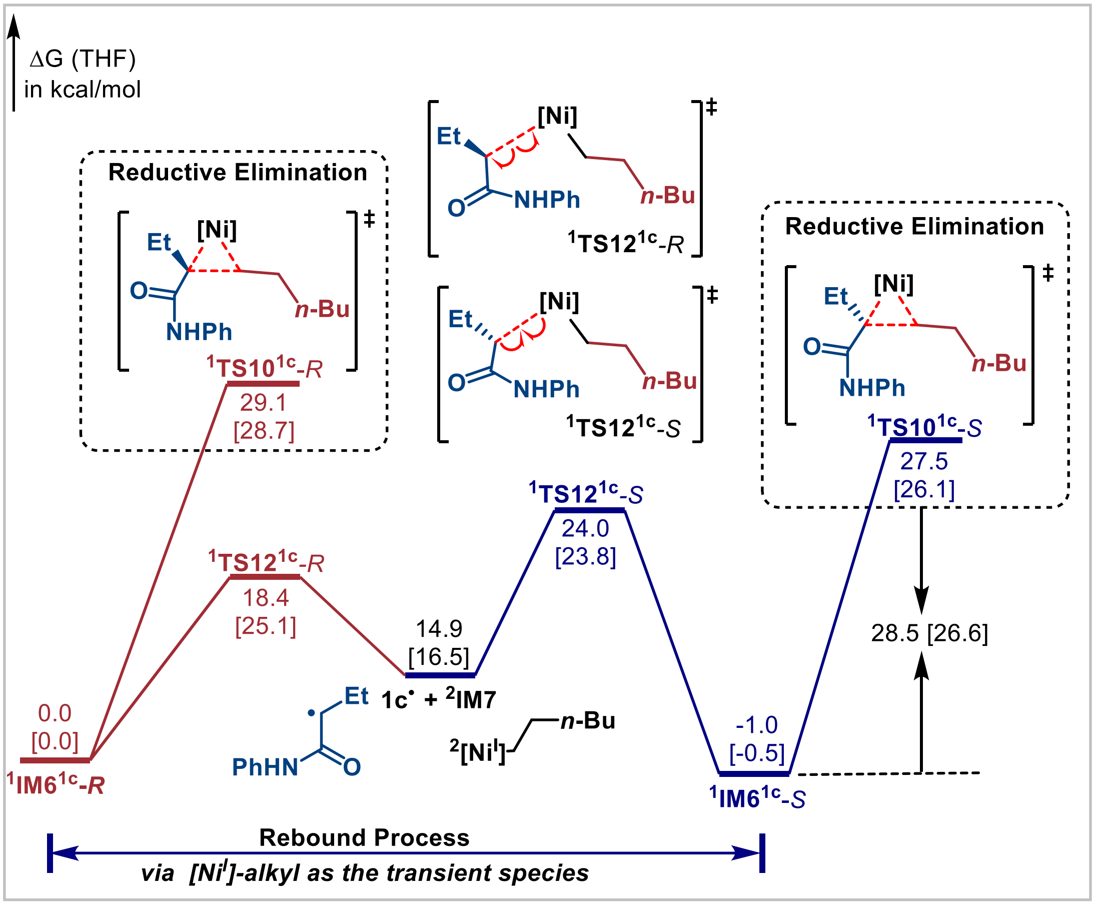

Fig. S18. Comparing the energetic results (in kcal/mol) of the processes of **^{1}IM6^{1c}** and **^{1}IM6**. The values in the square brackets are the corresponding values of the tertiary alkyl bromide (**1**).

Examining the pathway of the Ni(I) cycle in Figure 4 in the main text, the processes from **^{2}[Ni^I]Br** to **^{2}IM7** are not related to alkyl bromide substrate. The radical addition barriers from **1•** + **^{1}IM9** to **^{2}TS16** is significantly higher than the reductive elimination barriers of the Ni(III) intermediates **^{2}IM10** via **^{2}TS17**. Therefore, we calculated the radical addition barriers of the secondary alkyl bromide **1c**. The radical addition barrier (9.3 kcal/mol from **1c•** + **^{1}IM9** to **^{2}TS16^{1c}**-*S*) is comparable to that of tertiary alkyl bromide **1** (8.5 kcal/mol from **1•** + **^{1}IM9** to **^{2}TS16-*S***) (see Fig. S19). Thus, in the case of **1c**, the radical addition also take place easily. In addition, the barrier difference (1.9 kcal/mol) between **^{2}TS16^{1c}**-*R* and **^{2}TS16^{1c}**-*S* also

agrees with the high enantioselectivity of the substrate. The predicted *ee* value 96:4 is in good agreement with the experimental value of 97:3.

The detailed results presented above explain why the secondary alkyl bromides are also eligible electrophiles for the coupling reactions.

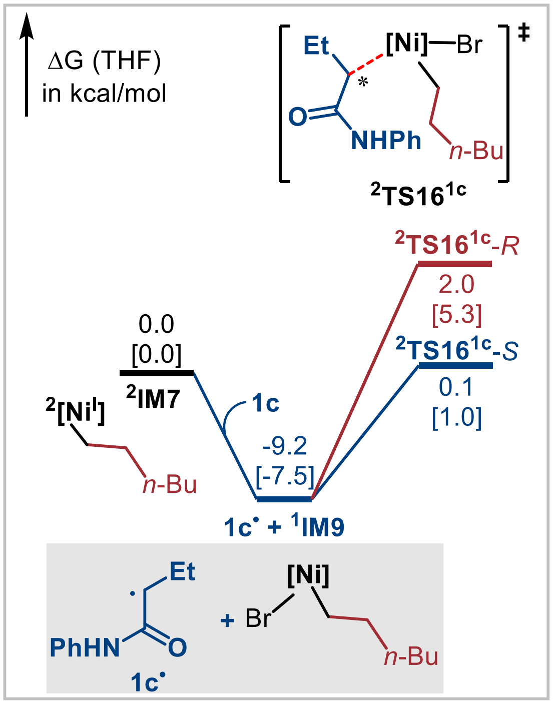

Fig. S19. Comparing the energetic results (in kcal/mol) of the processes starting from ^{2}**IM7.** The values in the square brackets are the corresponding values of the tertiary alkyl bromide (**1**).

SI12. References

[1] (a) K. S. Chan, X. Z. Li, W. I. Dzik, B. de Bruin, *J. Am. Chem. Soc.* 2008, **130**, 2051; (b) Q. Knijnenburg, D. Hetterscheid, T. M. Kooistra, P. H. M. Budzelaar, *Eur. J. Inorg. Chem.* 2004, **2004**, 1204; (c) X. Zhang, Z. Ke, N. J. DeYonker, H. Xu, Z. F. Li, X. Xu, X. Zhang, C. Y. Su, D. L. Phillips, C. Zhao, *J. Org. Chem.* 2013, **78**, 12460.

[2] Y. He, C. Liu, L. Yu, S. Zhu, *Angew. Chem. Int. Ed.* 2020, **59**, 21530.

[3] Y. Zhang, B. Han, S. Zhu, *Angew. Chem., Int. Ed.* 2019, **58**, 13860.

[4] F. Zhou, J. Zhu, Y. Zhang, S. Zhu, *Angew. Chem., Int. Ed.* 2018, **57,** 4058.

[5] F. Chen, X. Xu, Y. He, G. Huang, S. Zhu, *Angew. Chem., Int. Ed.* 2020, **59**, 5398.

[6] Z. Wang, H. Yin, G. C. Fu, *Nature.* 2018, **563**, 379.

SI13. Cartesian coordinates in Å, SCF energies and free energies (in a.u.) at 298.15 K and 1 atm for the optimized structures [BSI= 6-31G(d,p), BSII=6-311++G(d,p)]

$^{1}NiBr_2$·glyme

B3LYP/BSI SCF energy: -506.697496a.u.

M06/BSII SCF energy in solution: -506.539596a.u.

M06/BSII free energy in solution: -506.435723a.u.

C	-1.254345	-1.255342	2.354062
H	-1.013237	-0.347370	2.905937
H	-1.140818	-2.127574	3.006802
H	-2.274225	-1.192601	1.962905
O	-0.304059	-1.344476	1.277321
C	-0.450674	-2.517684	0.466632
H	-0.276883	-3.412104	1.078057
H	-1.462754	-2.553994	0.043285
C	0.588895	-2.427706	-0.631347
H	0.493446	-3.280138	-1.314866
H	1.602078	-2.401334	-0.211160
O	0.332666	-1.210570	-1.350239
C	1.231532	-0.978849	-2.452424
H	1.138574	-1.804131	-3.166040
H	0.913956	-0.047526	-2.920116
H	2.260920	-0.884939	-2.094839
Ni	-0.008391	0.312229	0.005914
Br	2.196311	1.039979	0.486494
Br	-2.222809	1.040797	-0.409173

$^{3}NiBr_2$·glyme

B3LYP/BSI SCF energy: -506.722892a.u.

M06/BSII SCF energy in solution: -506.564481a.u.

M06/BSII free energy in solution: -506.461159a.u.

C	1.224221	1.095228	2.425982
H	0.923102	0.176982	2.929244
H	1.126754	1.943589	3.111347
H	2.252027	1.001759	2.063378
O	0.313833	1.275062	1.323939
C	0.542494	2.472621	0.565452
H	0.415893	3.346704	1.215836
H	1.560062	2.461730	0.154916
C	-0.487821	2.492974	-0.544534
H	-0.332825	3.366684	-1.189253
H	-1.505227	2.513571	-0.133811
O	-0.298638	1.293676	-1.310965
C	-1.222082	1.145144	-2.406558
H	-1.108646	1.995641	-3.086793
H	-0.948776	0.223112	-2.918659
H	-2.248994	1.075243	-2.036177
Ni	-0.004078	-0.301521	-0.001734
Br	-2.221150	-1.041871	0.435028
Br	2.207390	-1.056647	-0.446666

glyme

B3LYP/BSI SCF energy: -308.86377a.u.

M06/BSII SCF energy in solution: -308.750078a.u.

M06/BSII free energy in solution: -308.640059a.u.

C	-1.233960	-2.715758	0.000000
H	-2.276964	-3.041925	0.000000
H	-0.733323	-3.125576	0.891798

H	-0.733323	-3.125576	-0.891798
O	-1.233960	-1.305091	0.000000
C	0.068864	-0.757412	0.000000
H	0.637666	-1.077575	0.888375
H	0.637666	-1.077575	-0.888375
C	-0.068864	0.757412	0.000000
H	-0.637666	1.077575	0.888375
H	-0.637666	1.077575	-0.888375
O	1.233960	1.305091	0.000000
C	1.233960	2.715758	0.000000
H	0.733323	3.125576	0.891798
H	2.276964	3.041925	0.000000
H	0.733323	3.125576	-0.891798

(*R,R*)-L*

B3LYP/BSI SCF energy: -1997.628056a.u.

M06/BSII SCF energy in solution: -1996.663378a.u.

M06/BSII free energy in solution: -1996.028855a.u.

C	1.255352	-1.143031	-0.110156
N	1.306324	-0.471289	0.966668
O	2.395712	-1.284708	-0.844098
C	3.492512	-0.707449	-0.064424
C	2.685031	-0.008247	1.141346
H	3.055076	-0.431663	2.079323
C	2.774725	1.505776	1.262079
C	3.926596	2.097217	1.798535
C	1.709913	2.330208	0.884014
C	4.021695	3.480893	1.937918
H	4.760696	1.473026	2.110070
C	1.800052	3.715979	1.029973
H	0.800345	1.880535	0.501002
C	2.955636	4.296744	1.552431
H	4.923994	3.920812	2.353662
H	0.956013	4.336829	0.743893
H	3.024161	5.375022	1.666839
C	-1.207399	-1.225782	-0.109421
N	-1.944316	-1.666866	0.824847
O	-1.557760	-0.049937	-0.712862
C	-2.842493	0.365126	-0.148089
C	-2.985728	-0.661826	1.074110
H	-2.695589	-0.129150	1.986967
C	-4.340503	-1.300418	1.318502
C	-5.141692	-0.876995	2.384949
C	-4.799353	-2.351758	0.513539
C	-6.385732	-1.464318	2.627081
H	-4.783824	-0.094255	3.049796
C	-6.037962	-2.943085	0.753372
H	-4.175238	-2.710698	-0.297959
C	-6.839380	-2.497441	1.807758
H	-6.991169	-1.121837	3.461629
H	-6.377814	-3.756722	0.118664
H	-7.804338	-2.960158	1.994566
C	0.045397	-1.892979	-0.646451
C	0.128557	-3.344145	-0.117715
H	1.069718	-3.799812	-0.437338
H	0.070336	-3.359063	0.971102
H	-0.702637	-3.934601	-0.512096
C	0.037769	-1.887431	-2.191487
H	-0.827038	-2.449212	-2.557075
H	-0.015641	-0.871661	-2.588994
H	0.942946	-2.362332	-2.573674
C	-2.709234	1.832119	0.260908
C	-1.981347	2.703992	-0.562979

C	-3.350594	2.358005	1.387645
C	-1.889608	4.061370	-0.259308
H	-1.486908	2.311106	-1.444662
C	-3.262733	3.719450	1.690738
H	-3.932762	1.712562	2.036010
C	-2.530828	4.576004	0.870719
H	-1.322559	4.719694	-0.911707
H	-3.767549	4.105383	2.571747
H	-2.461930	5.634037	1.106161
C	-3.914769	0.224529	-1.238024
C	-5.194620	0.763767	-1.039470
C	-3.649081	-0.435938	-2.442712
C	-6.183277	0.632560	-2.011849
H	-5.421826	1.291064	-0.119253
C	-4.639507	-0.563446	-3.420390
H	-2.660811	-0.838254	-2.625168
C	-5.910557	-0.032637	-3.209406
H	-7.168112	1.055232	-1.834405
H	-4.409674	-1.076344	-4.350367
H	-6.680150	-0.130612	-3.969688
C	4.252169	0.243077	-0.991513
C	5.635868	0.429087	-0.884257
C	3.552937	0.973932	-1.963099
C	6.302308	1.328548	-1.718750
H	6.203780	-0.136236	-0.153879
C	4.219058	1.867465	-2.799842
H	2.483662	0.834341	-2.071521
C	5.597822	2.051162	-2.680386
H	7.376656	1.455225	-1.618709
H	3.658111	2.420580	-3.547973
H	6.116717	2.747275	-3.333145
C	4.364009	-1.873874	0.415965
C	4.457378	-3.035620	-0.364154
C	5.120544	-1.801727	1.593139
C	5.268029	-4.099066	0.031098
H	3.884694	-3.102999	-1.282033
C	5.939928	-2.862735	1.985887
H	5.081391	-0.914352	2.217408
C	6.013350	-4.018013	1.208797
H	5.318373	-4.992622	-0.584790
H	6.515270	-2.783786	2.903887
H	6.644535	-4.846388	1.517054

1 (racemic)

B3LYP/BSI SCF energy: -530.441115a.u.

M06/BSII SCF energy in solution: -530.189813a.u.

M06/BSII free energy in solution: -530.049393a.u.

C	0.429134	0.957885	0.278642
C	0.426499	-1.124757	0.651975
H	0.203750	-1.584303	1.622219
H	0.554991	-1.896281	-0.110840
N	-0.513379	-0.056255	0.275584
O	0.363762	2.147694	0.077961
C	-1.899295	-0.083545	0.069056
C	-2.590807	-1.298963	0.168746
C	-2.590828	1.100468	-0.238388
C	-3.969452	-1.328407	-0.038330
H	-2.054626	-2.213682	0.402399
C	-3.967241	1.050016	-0.442119
H	-2.042512	2.031921	-0.312683
C	-4.664275	-0.157628	-0.343969
H	-4.499161	-2.273400	0.039142
H	-4.499703	1.966231	-0.680328

H -5.737464 -0.184860 -0.505172
C 2.232878 0.234503 2.014907
H 2.805744 1.162420 1.957156
H 2.906314 -0.583522 2.282857
H 1.481321 0.343107 2.806474
C 1.533628 -0.035874 0.700514
Br 2.912099 -0.269645 -0.770863

2

B3LYP/BSI SCF energy: -235.861282a.u.

M06/BSII SCF energy in solution: -235.711483a.u.

M06/BSII free energy in solution: -235.577941a.u.

C 3.090082 -0.171647 -0.439429
H 3.976968 -0.775157 -0.269103
H 3.097908 0.468005 -1.319380
C 2.045307 -0.207728 0.388216
H 2.082860 -0.867419 1.257073
C 0.777284 0.584811 0.224896
H 0.863494 1.243062 -0.649045
H 0.644430 1.241259 1.098343
C -0.474686 -0.301846 0.086018
H -0.360092 -0.947735 -0.794707
H -0.539236 -0.976516 0.951535
C -1.776312 0.501067 -0.029453
H -1.709243 1.176549 -0.893188
H -1.881659 1.147353 0.852789
C -3.020046 -0.382626 -0.167607
H -2.958870 -1.016547 -1.059580
H -3.931406 0.218782 -0.249545
H -3.134934 -1.043826 0.698957

H[Si]

B3LYP/BSI SCF energy: -753.572857a.u.

M06/BSII SCF energy in solution: -753.394784a.u.

M06/BSII free energy in solution: -753.217302a.u.

Si -0.016028 -0.035900 -0.782168
O -1.548380 -0.596412 -0.570616
O 0.017197 1.541798 -0.310812
C -2.314172 -0.429536 0.628636
H -2.327471 0.630411 0.910365
H -1.839982 -0.986019 1.448844
C -3.725359 -0.939795 0.388292
H -4.331021 -0.827174 1.293816
H -4.202811 -0.379593 -0.420963
H -3.710738 -1.997778 0.109859
C 1.199914 2.303704 -0.054453
H 1.868482 1.740131 0.607725
H 1.736788 2.480388 -0.997653
C 0.806374 3.628507 0.578916
H 0.281804 3.460745 1.524311
H 1.695932 4.235476 0.778111
H 0.143751 4.191005 -0.085429
O 1.123105 -0.815020 0.134184
C 1.520971 -2.169556 -0.084839
H 0.747737 -2.846057 0.303984
H 1.619198 -2.370083 -1.162105
C 2.845360 -2.422061 0.618084
H 2.752262 -2.226763 1.690495
H 3.160091 -3.461885 0.480335
H 3.624857 -1.767179 0.217336
H 0.271622 -0.243530 -2.218556

K_3PO_4

B3LYP/BSI SCF energy: -2442.201991a.u.

M06/BSII SCF energy in solution: -2442.261433a.u.

M06/BSII free energy in solution: -2442.281698a.u.

O	0.925403	-0.000109	1.253608
P	-0.033792	0.000019	0.000772
O	-0.931601	1.298812	0.001451
O	-0.931675	-1.298726	0.001282
O	0.923337	0.000057	-1.253716
K	-3.047048	0.000091	-0.000585
K	1.539819	-2.284845	-0.000564
K	1.540027	2.284725	-0.000566

THF

B3LYP/BSI SCF energy: -232.456281 a.u.

M06/BSII SCF energy in solution: -232.3549a.u.

M06/BSII free energy in solution: -232.266509 a.u.

C	0.733046	0.996862	0.227024
C	1.165695	-0.430366	-0.131454
C	-1.165132	-0.431138	0.132539
C	-0.734007	0.996026	-0.227512
H	0.795336	1.155249	1.309712
H	1.342957	1.761316	-0.262625
H	1.949926	-0.821002	0.527375
H	1.537363	-0.481135	-1.166625
H	-1.950588	-0.822730	-0.524210
H	-1.534358	-0.481454	1.168653
H	-0.796527	1.153084	-1.310381
H	-1.344536	1.760475	0.261381
O	0.000352	-1.251514	-0.000857

$^1[Ni^{II}]Br_2$

B3LYP/BSI SCF energy: -2195.489146a.u.

M06/BSII SCF energy in solution: -2194.48696825a.u.

M06/BSII free energy in solution: -2193.856965a.u.

C	1.260515	-0.087939	-1.225850
N	1.399783	-0.090208	0.051560
O	2.384416	-0.164194	-1.954536
C	3.483478	-0.528282	-1.012102
C	2.858468	-0.065822	0.353946
H	3.052142	-0.804133	1.129295
C	3.258863	1.294601	0.899191
C	3.871261	1.366294	2.155554
C	3.006920	2.483181	0.199649
C	4.237923	2.598972	2.698430
H	4.038966	0.454642	2.721452
C	3.372110	3.714484	0.740349
H	2.529164	2.451680	-0.775101
C	3.990708	3.775239	1.991634
H	4.703389	2.637448	3.678706
H	3.172455	4.627144	0.186219
H	4.269497	4.735754	2.415081
C	-1.262284	0.088012	-1.231928
N	-1.400883	0.063461	0.045312
O	-2.383327	0.198649	-1.959176
C	-3.475760	0.561150	-1.006482
C	-2.859497	0.052001	0.346571
H	-3.044971	0.770270	1.142597
C	-3.283022	-1.317034	0.850923
C	-3.904029	-1.414841	2.101152
C	-3.046079	-2.488915	0.118988

C	-4.294692	-2.656279	2.605854
H	-4.059727	-0.517426	2.692589
C	-3.434945	-3.729061	0.621583
H	-2.560599	-2.437541	-0.851046
C	-4.062539	-3.815633	1.866800
H	-4.766734	-2.715219	3.581972
H	-3.246257	-4.628590	0.042681
H	-4.359768	-4.783212	2.260614
C	0.000320	-0.004262	-2.060925
C	-0.087730	-1.281343	-2.944712
H	0.811271	-1.362616	-3.557686
H	-0.167197	-2.180855	-2.327733
H	-0.961693	-1.219637	-3.595774
C	0.091814	1.257956	-2.964286
H	-0.804510	1.329402	-3.582164
H	0.168191	2.167633	-2.361827
H	0.968611	1.186656	-3.610182
Ni	0.014750	0.003680	1.489837
C	-4.753762	-0.137956	-1.434399
C	-5.920138	0.087167	-0.688720
C	-4.810319	-0.987143	-2.543297
C	-7.114559	-0.537981	-1.038315
H	-5.893789	0.757478	0.165455
C	-6.011656	-1.607650	-2.896883
H	-3.917778	-1.161671	-3.132529
C	-7.164927	-1.388710	-2.145610
H	-8.007500	-0.358013	-0.447030
H	-6.040808	-2.262961	-3.762735
H	-8.097290	-1.873272	-2.419987
C	-3.591760	2.090029	-1.095612
C	-4.286608	2.649526	-2.180767
C	-2.960519	2.952566	-0.186277

C	-4.357308	4.030343	-2.349828
H	-4.780520	1.997114	-2.893395
C	-3.036802	4.337973	-0.356992
H	-2.406386	2.574618	0.667167
C	-3.733581	4.881645	-1.434681
H	-4.904855	4.440899	-3.193405
H	-2.548128	4.986417	0.364168
H	-3.794099	5.958886	-1.560412
C	4.746574	0.208549	-1.420876
C	5.917732	-0.011710	-0.681279
C	4.784811	1.088085	-2.506660
C	7.098491	0.647959	-1.013533
H	5.905731	-0.705119	0.154598
C	5.972495	1.743515	-2.842930
H	3.888672	1.259153	-3.091416
C	7.130356	1.529225	-2.097388
H	7.995265	0.471098	-0.427140
H	5.987506	2.422163	-3.690994
H	8.052064	2.040834	-2.358348
C	3.630850	-2.051701	-1.139681
C	4.344457	-2.570045	-2.233012
C	3.006711	-2.948291	-0.258772
C	4.440726	-3.944620	-2.437307
H	4.832566	-1.890734	-2.924135
C	3.108594	-4.327112	-0.465119
H	2.437990	-2.601863	0.598641
C	3.824039	-4.830170	-1.550313
H	5.002370	-4.323458	-3.286452
H	2.624689	-5.002623	0.234086
H	3.904216	-5.902536	-1.703798
Br	-1.141534	1.364467	3.103739
Br	1.132667	-1.506327	3.004764

^{3}[Ni^{II}]Br$_2$

B3LYP/BSI SCF energy: -2195.517076a.u.

M06/BSII SCF energy in solution: -2194.509113a.u.

M06/BSII free energy in solution: -2193.881203a.u.

C	1.270381	-0.100976	-1.153597
N	1.420178	-0.111460	0.119983
O	2.386136	-0.153906	-1.894681
C	3.524508	-0.456689	-0.985584
C	2.866188	-0.178065	0.435619
H	3.020950	-1.047721	1.075859
C	3.325493	1.044422	1.207740
C	4.076690	0.866054	2.374747
C	3.007944	2.344831	0.797110
C	4.516222	1.965721	3.113145
H	4.310169	-0.140063	2.714138
C	3.443936	3.444056	1.533599
H	2.409148	2.501907	-0.094700
C	4.201681	3.257585	2.692241
H	5.092355	1.810825	4.020564
H	3.185777	4.447284	1.207655
H	4.535232	4.115624	3.268493
C	-1.268265	0.082608	-1.161043
N	-1.440194	-0.048861	0.103058
O	-2.355875	0.370793	-1.890380
C	-3.449266	0.723958	-0.942786
C	-2.878647	0.135565	0.413835
H	-2.953612	0.889610	1.197797
C	-3.490113	-1.145687	0.950129
C	-4.199091	-1.106213	2.155972
C	-3.348653	-2.370674	0.286003
C	-4.769173	-2.265599	2.684280
H	-4.294782	-0.165039	2.691260
C	-3.914809	-3.529327	0.812587
H	-2.786998	-2.425290	-0.641438
C	-4.628918	-3.479459	2.012295
H	-5.310392	-2.219888	3.624660
H	-3.792023	-4.473852	0.290944
H	-5.063887	-4.385151	2.424701
C	-0.002717	-0.089272	-1.981247
C	-0.100967	-1.463289	-2.709442
H	0.783132	-1.613135	-3.331818
H	-0.164349	-2.281949	-1.987445
H	-0.989682	-1.477779	-3.344537
C	0.091843	1.057555	-3.022176
H	-0.792630	1.051536	-3.659631
H	0.152326	2.032685	-2.530110
H	0.979998	0.918594	-3.640120
Ni	-0.026499	-0.280359	1.535185
C	-4.736383	0.075551	-1.425000
C	-5.917748	0.304092	-0.703900
C	-4.784465	-0.733398	-2.563908
C	-7.117382	-0.276675	-1.107694
H	-5.898684	0.941573	0.174955
C	-5.990347	-1.310667	-2.971428
H	-3.881333	-0.908932	-3.136236
C	-7.158400	-1.087657	-2.244877
H	-8.021589	-0.094037	-0.534594
H	-6.011178	-1.935073	-3.860064
H	-8.094564	-1.538382	-2.560895
C	-3.516443	2.258375	-0.968709
C	-4.293637	2.896953	-1.948412
C	-2.741270	3.050595	-0.107338

C	-4.310941	4.286603	-2.054171
H	-4.892373	2.302798	-2.630053
C	-2.762150	4.443248	-0.214586
H	-2.110111	2.610221	0.657998
C	-3.547241	5.066510	-1.183713
H	-4.925031	4.758797	-2.815705
H	-2.160789	5.034554	0.469625
H	-3.565400	6.149905	-1.260597
C	4.679972	0.467935	-1.334765
C	5.894683	0.320127	-0.648587
C	4.573500	1.459789	-2.313945
C	6.973891	1.154257	-0.929523
H	5.996765	-0.453665	0.106092
C	5.659050	2.292902	-2.598661
H	3.644351	1.580655	-2.857994
C	6.860167	2.145625	-1.907643
H	7.905008	1.028670	-0.384883
H	5.560292	3.056964	-3.364482
H	7.702437	2.794668	-2.128451
C	3.850157	-1.936587	-1.234652
C	4.769632	-2.287457	-2.236157
C	3.172162	-2.963729	-0.559119
C	5.021916	-3.624729	-2.538925
H	5.295727	-1.510341	-2.779233
C	3.427689	-4.302409	-0.863948
H	2.432081	-2.751940	0.206728
C	4.354706	-4.638866	-1.849783
H	5.742956	-3.872068	-3.312827
H	2.895633	-5.078152	-0.321263
H	4.555399	-5.681218	-2.080456
Br	-0.464122	1.685427	2.877072
Br	0.359475	-2.586026	2.175390

$^{1}[Ni^{II}]Br_2$

B3LYP-D3BJ/BSI SCF energy in solution:

-2195.821062a.u.

M06/BSII SCF energy in solution: -2194.483533a.u.

M06/BSII free energy in solution: -2193.855275a.u.

C	1.253309	-0.062664	-1.245607
N	1.366432	-0.238326	0.024910
O	2.391380	-0.023076	-1.941586
C	3.466471	-0.509952	-1.024141
C	2.806300	-0.209693	0.363343
H	3.030624	-1.006865	1.067057
C	3.176618	1.112670	1.000650
C	4.233295	1.146494	1.916867
C	2.519344	2.302964	0.671850
C	4.635654	2.352763	2.487756
H	4.743360	0.224197	2.178422
C	2.916222	3.510196	1.248488
H	1.690355	2.295584	-0.027513
C	3.976943	3.538685	2.154767
H	5.457578	2.365731	3.197249
H	2.395342	4.426990	0.988570
H	4.285533	4.478062	2.603605
C	-1.238144	0.164240	-1.241082
N	-1.359461	0.191064	0.040958
O	-2.370227	0.243325	-1.944509
C	-3.434451	0.665044	-0.983475
C	-2.803360	0.177380	0.362556
H	-3.004670	0.893074	1.154767
C	-3.237512	-1.192689	0.835775
C	-4.311256	-1.283926	1.727913

C	-2.626558	-2.365330	0.379948
C	-4.779626	-2.528231	2.145907
H	-4.783263	-0.375493	2.090320
C	-3.090926	-3.611960	0.802528
H	-1.782938	-2.316360	-0.300034
C	-4.170382	-3.696822	1.682639
H	-5.614667	-2.585366	2.837641
H	-2.607471	-4.515423	0.442945
H	-4.530668	-4.666848	2.011799
C	0.009348	0.084482	-2.078704
C	-0.092580	-1.145618	-3.019416
H	0.806143	-1.204672	-3.635438
H	-0.184796	-2.071318	-2.444915
H	-0.962353	-1.040504	-3.669650
C	0.115466	1.391625	-2.908379
H	-0.781634	1.506746	-3.518342
H	0.208190	2.263086	-2.254370
H	0.986791	1.342766	-3.562974
Ni	-0.005651	-0.136945	1.419615
C	-4.724067	-0.035101	-1.332715
C	-5.923184	0.452487	-0.799232
C	-4.733641	-1.217094	-2.078530
C	-7.117066	-0.235801	-1.008743
H	-5.920671	1.367169	-0.215660
C	-5.931058	-1.900407	-2.293138
H	-3.807628	-1.604074	-2.486087
C	-7.124474	-1.414617	-1.757186
H	-8.040323	0.150007	-0.587254
H	-5.928184	-2.816098	-2.876754
H	-8.054501	-1.950382	-1.921817
C	-3.486519	2.182121	-1.120642
C	-4.121644	2.727840	-2.246417
C	-2.829665	3.041766	-0.230426
C	-4.111749	4.102164	-2.471734
H	-4.624739	2.071114	-2.948293
C	-2.821665	4.420268	-0.458022
H	-2.315959	2.660641	0.645271
C	-3.461899	4.954562	-1.575303
H	-4.611621	4.507574	-3.346294
H	-2.312496	5.072623	0.245078
H	-3.456096	6.026595	-1.748036
C	4.726692	0.279612	-1.275456
C	5.938270	-0.204681	-0.766979
C	4.695883	1.524177	-1.910372
C	7.104155	0.548983	-0.890839
H	5.966908	-1.169700	-0.271507
C	5.866095	2.273415	-2.039984
H	3.760512	1.907524	-2.299707
C	7.071466	1.790691	-1.529208
H	8.037210	0.164749	-0.490057
H	5.832454	3.237161	-2.539153
H	7.980149	2.376678	-1.628852
C	3.585212	-1.997615	-1.333157
C	4.296749	-2.392620	-2.475584
C	2.910169	-2.971131	-0.584157
C	4.346813	-3.732669	-2.852928
H	4.813560	-1.646272	-3.069395
C	2.962005	-4.314161	-0.964522
H	2.337316	-2.704914	0.297253
C	3.680197	-4.699460	-2.096035
H	4.907155	-4.021545	-3.737033
H	2.438356	-5.056549	-0.369584
H	3.721665	-5.744832	-2.387045
Br	-1.064512	1.268546	3.097658

Br 1.039889 -1.799171 2.854370

^{3}[Ni^{II}]Br$_2$

B3LYP-D3BJ/BSI SCF energy in solution:

-2195.844883a.u.

M06/BSII SCF energy in solution: -2194.513428a.u.

M06/BSII free energy in solution: -2193.881352a.u.

C 1.263385 -0.328265 -1.126405
N 1.359201 -0.465921 0.148411
O 2.397146 -0.095812 -1.787788
C 3.519955 -0.304301 -0.826968
C 2.755450 -0.202159 0.549758
H 3.098942 -0.990207 1.219892
C 2.861748 1.128379 1.260717
C 3.644166 1.230660 2.413149
C 2.220148 2.268285 0.763884
C 3.794340 2.458412 3.059659
H 4.137620 0.345190 2.804429
C 2.372819 3.496222 1.404283
H 1.597090 2.202936 -0.122137
C 3.161090 3.594866 2.553816
H 4.402969 2.525679 3.956405
H 1.875394 4.375053 1.005988
H 3.277908 4.551698 3.053964
C -1.212195 -0.094417 -1.189788
N -1.441991 -0.346444 0.051053
O -2.178458 0.545023 -1.850924
C -3.112572 1.079037 -0.810802
C -2.813898 0.089447 0.371255
H -2.815330 0.629991 1.315794
C -3.755333 -1.088756 0.490908
C -4.853856 -0.988006 1.351368
C -3.586200 -2.250441 -0.268737
C -5.779695 -2.026685 1.441329
H -4.987322 -0.088407 1.945231
C -4.508976 -3.292655 -0.175527
H -2.730529 -2.353065 -0.925736
C -5.609048 -3.182733 0.676712
H -6.629219 -1.935190 2.111445
H -4.365286 -4.191615 -0.767730
H -6.326681 -3.994630 0.748331
C 0.021475 -0.476099 -1.981924
C -0.092270 -1.977589 -2.375676
H 0.803291 -2.275831 -2.926226
H -0.191528 -2.609468 -1.490915
H -0.962312 -2.116134 -3.022454
C 0.138678 0.398207 -3.244894
H -0.742918 0.253990 -3.870372
H 0.214480 1.458869 -2.992486
H 1.022165 0.107104 -3.813410
Ni -0.118437 -1.011772 1.378084
C -4.527119 0.998233 -1.326616
C -5.526748 1.722771 -0.665854
C -4.876277 0.144013 -2.375790
C -6.860412 1.589227 -1.047901
H -5.260662 2.386652 0.150344
C -6.211445 0.018442 -2.762099
H -4.107353 -0.421786 -2.887937
C -7.206790 0.736207 -2.098030
H -7.627899 2.152348 -0.525578
H -6.471826 -0.644787 -3.581524
H -8.245541 0.633804 -2.397314
C -2.616845 2.499709 -0.562332

C	-2.924644	3.488354	-1.509171
C	-1.770325	2.824703	0.505248
C	-2.410250	4.776683	-1.383923
H	-3.569303	3.244356	-2.346919
C	-1.258457	4.118770	0.631346
H	-1.491609	2.090230	1.251597
C	-1.576132	5.097504	-0.309356
H	-2.662207	5.530443	-2.123784
H	-0.608085	4.351035	1.468586
H	-1.178621	6.102994	-0.207999
C	4.542093	0.789005	-1.032063
C	5.728831	0.741166	-0.288772
C	4.316101	1.862411	-1.895549
C	6.672678	1.758559	-0.405098
H	5.912634	-0.093174	0.380919
C	5.267019	2.878867	-2.015169
H	3.399909	1.907308	-2.471452
C	6.444481	2.832225	-1.270136
H	7.586741	1.712900	0.179141
H	5.081297	3.708127	-2.691233
H	7.181257	3.624653	-1.361445
C	4.042005	-1.702962	-1.134574
C	5.048050	-1.872455	-2.096076
C	3.456459	-2.840392	-0.560411
C	5.472790	-3.148758	-2.462699
H	5.501579	-1.003816	-2.559976
C	3.881585	-4.116821	-0.930128
H	2.656129	-2.757434	0.165648
C	4.892613	-4.276210	-1.878443
H	6.257125	-3.260165	-3.205401
H	3.418046	-4.985550	-0.472121
H	5.224587	-5.270659	-2.161564
Br	-0.468057	0.373488	3.330791
Br	0.065214	-3.435914	1.341207

K_3PO_4@[Si]H

B3LYP/BSI SCF energy: -3195.848757a.u.

M06/BSII SCF energy in solution: -3195.719331a.u.

M06/BSII free energy in solution: -3195.535384a.u.

Si	0.207563	0.807336	0.741564
H	0.163524	1.453767	2.098988
O	0.203610	1.711472	-0.713158
O	2.010346	0.766770	0.811548
C	1.329399	2.092935	-1.493343
H	1.721012	1.213680	-2.024573
H	2.127833	2.478043	-0.852343
C	0.900794	3.156503	-2.499240
H	1.754847	3.482552	-3.103581
H	0.136180	2.775770	-3.189230
H	0.485712	4.029963	-1.985977
C	2.681276	0.695407	2.041339
H	2.080247	1.148316	2.847160
H	2.843722	-0.359374	2.350192
C	4.034703	1.401959	1.967330
H	3.898411	2.458933	1.717862
H	4.565757	1.334642	2.924125
H	4.673583	0.958635	1.193130
O	-1.650214	0.962458	0.710428
C	-2.198015	2.231982	0.964127
H	-1.595607	2.777173	1.712439
H	-2.188994	2.857783	0.053553
C	-3.634581	2.116683	1.475995
H	-3.666018	1.582238	2.434554

H -4.082532 3.104609 1.633680

H -4.263718 1.571662 0.760692

O 0.525815 -3.216469 -0.132085

P -0.221057 -1.990091 -0.631030

O 0.438198 -1.257453 -1.824171

O -1.763024 -2.039901 -0.727891

O 0.047872 -0.873015 0.679217

K -2.324485 -1.435512 1.662910

K -1.676070 0.266485 -2.078822

K 2.662330 -1.616418 -0.355429

K_3PO_4@1

B3LYP/BSI SCF energy: -2972.678761a.u.

M06/BSII SCF energy in solution: -2972.472436a.u.

M06/BSII free energy in solution: -2972.331062a.u.

Br -3.311682 -1.256294 -0.131350

C -2.325303 1.574330 -0.062236

C -3.946764 0.783864 1.812375

H -3.696681 1.698468 2.363825

H -4.864497 0.962415 1.247344

H -4.119907 -0.018269 2.534602

C -2.796649 0.449634 0.886320

C 0.025112 2.415221 0.419046

C 1.199109 2.083963 1.119870

C -0.008742 3.539095 -0.428469

C 2.320163 2.905413 0.987624

H 1.257892 1.130502 1.649841

C 1.131115 4.334994 -0.556286

H -0.919131 3.768582 -0.970039

C 2.298183 4.033124 0.156945

H 3.228739 2.641279 1.521921

H 1.099047 5.206429 -1.205055

H 3.174462 4.669449 0.068511

N -1.107573 1.616762 0.573116

C -1.359838 0.456102 1.461518

H -1.321249 0.723141 2.524690

H -0.676783 -0.379843 1.262933

O -2.819483 2.217790 -0.964209

O 0.608046 -0.578403 -1.066151

P 1.615785 -1.301889 -0.084665

O 3.084157 -0.791681 -0.356719

O 1.553121 -2.863460 -0.320626

O 1.268037 -1.023424 1.423034

K 3.396511 -2.626085 1.595667

K -0.582113 -2.727619 -1.625076

K 2.316858 1.176763 -1.741436

K_3PO_4@2

B3LYP/BSI SCF energy: -2678.081844a.u.

M06/BSII SCF energy in solution: -2677.983231a.u.

M06/BSII free energy in solution: -2677.852702a.u.

O -2.601115 0.660047 -0.801511

P -1.350751 -0.086040 -0.205892

O -0.561626 -0.853822 -1.334299

O -1.780554 -1.062073 0.952427

O -0.428517 1.061989 0.387633

K -0.594621 -3.030708 -0.044092

K -2.737455 1.258636 1.802612

K -0.604006 1.653560 -2.253277

C 2.784808 -3.050316 0.105841

H 2.785080 -3.846960 -0.635951

H 3.175716 -3.302997 1.090031

C	2.373138	-1.809276	-0.191386
H	1.973596	-1.603097	-1.185551
C	2.347816	-0.631659	0.735470
H	2.737259	-0.912950	1.723721
H	1.306365	-0.289689	0.847298
C	3.128600	0.576690	0.182643
H	4.203197	0.349651	0.124453
H	2.795553	0.761837	-0.849431
C	2.886636	1.843439	1.012257
H	3.227103	1.671392	2.043450
H	1.802550	2.003329	1.054821
C	3.593116	3.080035	0.447564
H	4.679967	2.939556	0.400074
H	3.400804	3.968761	1.059045
H	3.249468	3.300882	-0.571530

K_3PO_4@THF_3

B3LYP/BSI SCF energy: -3139.644008a.u.

M06/BSII SCF energy in solution: -3139.371103a.u.

M06/BSII free energy in solution: -3139.067191a.u.

O	-1.053852	0.537306	-0.965300
P	0.356757	0.110774	-0.403105
O	1.035057	1.323010	0.357740
O	1.289160	-0.380019	-1.577570
O	0.080956	-1.067067	0.608445
K	3.156761	1.246822	-1.044011
K	-0.773373	-1.962170	-1.999411
K	-1.195959	1.100509	1.779167
C	-3.490702	-1.635137	-0.110799
O	-3.147128	-2.774007	-0.932065
C	-3.308539	-3.972839	-0.150527
C	-3.436436	-3.539140	1.328022
C	-3.045277	-2.049626	1.289985
H	-2.939313	-0.769239	-0.496368
H	-4.576032	-1.454471	-0.165298
H	-4.194665	-4.522758	-0.494222
H	-2.429065	-4.604807	-0.325390
H	-4.468664	-3.663010	1.672791
H	-2.792877	-4.128561	1.986793
H	-3.537770	-1.471327	2.081393
H	-1.957108	-1.916403	1.351179
C	3.545308	-0.649181	1.728162
O	4.487908	-0.424002	0.636623
C	4.791476	-1.697370	0.048708
C	3.492034	-2.489446	0.161398
C	2.981817	-2.075566	1.555744
H	4.075987	-0.518275	2.680623
H	2.756022	0.103954	1.634376
H	5.125995	-1.518699	-0.980465
H	5.615517	-2.183915	0.596072
H	2.780337	-2.138118	-0.594475
H	3.641690	-3.569356	0.057394
H	1.887930	-2.051365	1.572881
H	3.368756	-2.746155	2.332044
C	-2.528903	3.325040	-0.480036
O	-2.149088	3.521856	0.917199
C	-1.084993	4.485643	0.974071
C	-0.361696	4.350128	-0.363260
C	-1.545302	4.151718	-1.324610
H	-2.411588	2.260897	-0.709061
H	-3.570622	3.645405	-0.604620
H	-1.497958	5.497465	1.117125
H	-0.456517	4.248218	1.840878

H	0.251660	5.225103	-0.604419
H	0.258829	3.445674	-0.340119
H	-1.979835	5.116561	-1.612788
H	-1.260045	3.611562	-2.230632

K_3PO_4@THF_6

B3LYP/BSI SCF energy: -3837.070032a.u.

M06/BSII SCF energy in solution: -3836.470866a.u.

M06/BSII free energy in solution: -3835.849721a.u.

O	1.491690	0.212673	-0.685019
P	0.228824	-0.113439	0.214450
O	0.365099	-1.574174	0.787450
O	-1.075542	0.028714	-0.661363
O	0.237010	0.950975	1.376102
K	-1.284975	-2.561677	-0.914750
K	-0.087413	2.588048	-0.801627
K	2.620806	-0.345489	1.847053
C	-1.858512	3.269981	2.030723
O	-1.733548	4.038906	0.813862
C	-2.830973	4.954387	0.795308
C	-4.008968	4.246832	1.514642
C	-3.357739	2.974642	2.115362
H	-1.215965	2.384422	1.937307
H	-1.528528	3.887206	2.883042
H	-2.553337	5.881033	1.320874
H	-3.041460	5.204780	-0.249277
H	-4.430472	4.892504	2.290957
H	-4.817749	3.998158	0.821696
H	-3.688653	2.771919	3.138075
H	-3.587826	2.096451	1.504634
C	3.183216	2.640372	-1.888081
O	2.187587	3.688220	-1.843615
C	2.851762	4.954756	-1.704679
C	4.311741	4.656022	-1.287395
C	4.285289	3.149850	-0.963047
H	2.699418	1.712251	-1.558868
H	3.557724	2.530910	-2.918966
H	2.807199	5.507303	-2.653306
H	2.308703	5.534634	-0.948906
H	4.994398	4.853597	-2.120407
H	4.635868	5.270465	-0.442834
H	5.248164	2.659535	-1.134317
H	3.995413	2.982506	0.080857
C	-4.006294	-0.930191	0.254987
O	-3.923878	-2.233304	-0.363565
C	-5.267849	-2.697701	-0.500506
C	-6.135367	-1.445322	-0.782922
C	-5.183957	-0.261774	-0.462607
H	-4.218179	-1.054611	1.329471
H	-3.037153	-0.432946	0.116427
H	-5.287804	-3.438045	-1.306026
H	-5.588416	-3.191443	0.430040
H	-6.474778	-1.415593	-1.822081
H	-7.026169	-1.439135	-0.147901
H	-4.832442	0.212089	-1.384232
H	-5.660839	0.508913	0.149963
C	-0.937013	-2.524356	-4.591606
O	-0.133397	-2.385017	-3.416806
C	0.532338	-1.107442	-3.551265
C	-0.570726	-0.176263	-4.064713
C	-1.466458	-1.107469	-4.925495
H	-0.323755	-2.917227	-5.417403
H	-1.725464	-3.254185	-4.378346

H	0.930805	-0.805488	-2.575754
H	1.350562	-1.210942	-4.282877
H	-1.113673	0.202488	-3.193645
H	-0.171749	0.669392	-4.634008
H	-2.522725	-1.001455	-4.659778
H	-1.376266	-0.901216	-5.996392
C	3.796277	-2.116061	-0.878204
O	4.186281	-2.219460	0.525667
C	3.894000	-3.543662	0.989875
C	2.713745	-4.005009	0.137134
C	3.111779	-3.446938	-1.240118
H	3.103009	-1.270582	-0.966999
H	4.698574	-1.934500	-1.475812
H	4.770146	-4.199376	0.856047
H	3.675140	-3.485893	2.062651
H	2.578815	-5.091718	0.145771
H	1.801200	-3.506193	0.483411
H	3.810040	-4.127520	-1.742393
H	2.253583	-3.292414	-1.900155
C	1.207231	-2.390889	4.364834
O	1.604299	-1.009387	4.334039
C	0.418515	-0.167326	4.466704
C	-0.766960	-1.121964	4.660115
C	-0.266429	-2.397546	3.965093
H	1.846138	-2.947095	3.668823
H	1.361951	-2.800966	5.376002
H	0.571519	0.504521	5.320078
H	0.303812	0.407283	3.538891
H	-0.960686	-1.306901	5.724205
H	-1.679990	-0.724085	4.208570
H	-0.789195	-3.304253	4.287801
H	-0.337904	-2.280487	2.876653

[1]TS1

B3LYP/BSI SCF energy: -2431.309408a.u.

M06/BSII SCF energy in solution: -2430.165294a.u.

M06/BSII free energy in solution: -2429.379947a.u.

C	-1.430286	-1.027830	1.164312
N	-1.563125	-0.299103	0.117871
O	-2.527856	-1.672653	1.599289
C	-3.537859	-1.569684	0.507243
C	-2.996706	-0.315367	-0.272939
C	1.096191	-0.976676	1.270804
N	1.291793	-0.383544	0.149383
O	2.186348	-1.445077	1.905798
C	3.365512	-0.860387	1.217869
C	2.735223	-0.510932	-0.180383
Ni	-0.096207	0.600629	-1.033682
C	-0.198004	-1.202379	2.034610
C	-0.264236	-0.140645	3.173510
H	-1.156881	-0.310696	3.781459
H	-0.300019	0.871171	2.762789
H	0.618176	-0.231486	3.811278
C	-0.186068	-2.624709	2.648403
H	0.686128	-2.741650	3.291956
H	-0.155669	-3.393659	1.871303
H	-1.090079	-2.777752	3.238320
C	4.479456	-1.895302	1.188549
C	5.757304	-1.497822	0.770351
C	4.269250	-3.228806	1.553280
C	6.799819	-2.419837	0.706905
H	5.939179	-0.461163	0.502402
C	5.318751	-4.149218	1.499816

H	3.287525	-3.547133	1.883428
C	6.584673	-3.750361	1.073699
H	7.782561	-2.096495	0.376496
H	5.141297	-5.180370	1.791855
H	7.399002	-4.467640	1.030340
C	3.760863	0.357694	2.067392
C	3.737457	1.679102	1.607083
C	4.143183	0.115005	3.398773
C	4.094881	2.730693	2.459374
H	3.444844	1.923768	0.592047
C	4.488806	1.162792	4.246602
H	4.174058	-0.906681	3.764982
C	4.468313	2.479719	3.776870
H	4.077060	3.747938	2.079273
H	4.782289	0.952315	5.271120
H	4.745042	3.299225	4.433894
C	-3.416444	-2.885504	-0.277620
C	-2.684990	-2.997031	-1.469418
C	-3.994880	-4.044643	0.266245
C	-2.549529	-4.236343	-2.102727
H	-2.211382	-2.137193	-1.931676
C	-3.854221	-5.278334	-0.364497
H	-4.563711	-3.976592	1.187852
C	-3.131594	-5.378306	-1.555944
H	-1.985904	-4.295789	-3.029046
H	-4.314249	-6.160442	0.071784
H	-3.027524	-6.338246	-2.053675
C	-4.915266	-1.402048	1.124730
C	-5.118311	-1.360398	2.507207
C	-6.022935	-1.297048	0.270964
C	-6.406361	-1.206927	3.027763
H	-4.270988	-1.451345	3.176743
C	-7.304853	-1.137175	0.791306
H	-5.881262	-1.345467	-0.804847
C	-7.501817	-1.091447	2.173955
H	-6.548538	-1.179239	4.104396
H	-8.151240	-1.051629	0.116211
H	-8.501957	-0.970418	2.579555
C	-0.169619	2.547782	-0.411868
H	0.100833	2.634377	0.646761
H	-1.251812	2.664427	-0.546868
C	0.521380	3.526896	-1.247216
H	0.065219	3.671434	-2.225560
C	1.040718	4.811533	-0.628973
H	1.680609	5.345833	-1.338027
H	1.652325	4.572645	0.249612
C	-0.129053	5.725326	-0.207153
H	-0.766621	5.926354	-1.079624
H	-0.760776	5.201002	0.520486
C	0.348183	7.056791	0.388327
H	0.978017	7.577667	-0.345071
H	0.993477	6.855001	1.254358
C	-0.805766	7.970676	0.813182
H	-1.449101	8.218329	-0.038847
H	-0.436808	8.911311	1.235447
H	-1.433874	7.488873	1.571315
C	2.946126	-1.513047	-1.304880
C	2.321307	-2.766979	-1.315122
C	3.786751	-1.169141	-2.369749
C	2.542130	-3.661142	-2.361565
H	1.652013	-3.050592	-0.508544
C	4.010092	-2.063136	-3.417698
H	4.253539	-0.188613	-2.388235
C	3.389312	-3.312103	-3.415433

H	2.048441	-4.628714	-2.356093
H	4.658064	-1.775986	-4.240443
H	3.555687	-4.006285	-4.234060
C	-3.642520	1.030216	0.015600
C	-4.226987	1.741002	-1.039167
C	-3.644650	1.598523	1.297097
C	-4.813799	2.988928	-0.818003
H	-4.201678	1.324200	-2.041963
C	-4.229529	2.843836	1.519788
H	-3.193647	1.066208	2.128822
C	-4.817979	3.542312	0.462133
H	-5.258628	3.528750	-1.648824
H	-4.227407	3.269543	2.519228
H	-5.271770	4.513708	0.636285
H	-3.052155	-0.479962	-1.347569
H	3.089424	0.454052	-0.535358
Br	-1.024212	0.292160	-3.275997
Br	2.450369	2.633759	-2.105036

^{3}TS1

B3LYP/BSI SCF energy: -2431.329907a.u.

M06/BSII SCF energy in solution: -2430.184543a.u.

M06/BSII free energy in solution: -2429.400003a.u.

C	-1.430286	-1.027830	1.164312
N	-1.563125	-0.299103	0.117871
O	-2.527856	-1.672653	1.599289
C	-3.537859	-1.569684	0.507243
C	-2.996706	-0.315367	-0.272939
C	1.096191	-0.976676	1.270804
N	1.291793	-0.383544	0.149383
O	2.186348	-1.445077	1.905798
C	3.365512	-0.860387	1.217869
C	2.735223	-0.510932	-0.180383
Ni	-0.096207	0.600629	-1.033682
C	-0.198004	-1.202379	2.034610
C	-0.264236	-0.140645	3.173510
H	-1.156881	-0.310696	3.781459
H	-0.300019	0.871171	2.762789
H	0.618176	-0.231486	3.811278
C	-0.186068	-2.624709	2.648403
H	0.686128	-2.741650	3.291956
H	-0.155669	-3.393659	1.871303
H	-1.090079	-2.777752	3.238320
C	4.479456	-1.895302	1.188549
C	5.757304	-1.497822	0.770351
C	4.269250	-3.228806	1.553280
C	6.799819	-2.419837	0.706905
H	5.939179	-0.461163	0.502402
C	5.318751	-4.149218	1.499816
H	3.287525	-3.547133	1.883428
C	6.584673	-3.750361	1.073699
H	7.782561	-2.096495	0.376496
H	5.141297	-5.180370	1.791855
H	7.399002	-4.467640	1.030340
C	3.760863	0.357694	2.067392
C	3.737457	1.679102	1.607083
C	4.143183	0.115005	3.398773
C	4.094881	2.730693	2.459374
H	3.444844	1.923768	0.592047
C	4.488806	1.162792	4.246602
H	4.174058	-0.906681	3.764982
C	4.468313	2.479719	3.776870
H	4.077060	3.747938	2.079273

H	4.782289	0.952315	5.271120
H	4.745042	3.299225	4.433894
C	-3.416444	-2.885504	-0.277620
C	-2.684990	-2.997031	-1.469418
C	-3.994880	-4.044643	0.266245
C	-2.549529	-4.236343	-2.102727
H	-2.211382	-2.137193	-1.931676
C	-3.854221	-5.278334	-0.364497
H	-4.563711	-3.976592	1.187852
C	-3.131594	-5.378306	-1.555944
H	-1.985904	-4.295789	-3.029046
H	-4.314249	-6.160442	0.071784
H	-3.027524	-6.338246	-2.053675
C	-4.915266	-1.402048	1.124730
C	-5.118311	-1.360398	2.507207
C	-6.022935	-1.297048	0.270964
C	-6.406361	-1.206927	3.027763
H	-4.270988	-1.451345	3.176743
C	-7.304853	-1.137175	0.791306
H	-5.881262	-1.345467	-0.804847
C	-7.501817	-1.091447	2.173955
H	-6.548538	-1.179239	4.104396
H	-8.151240	-1.051629	0.116211
H	-8.501957	-0.970418	2.579555
C	-0.169619	2.547782	-0.411868
H	0.100833	2.634377	0.646761
H	-1.251812	2.664427	-0.546868
C	0.521380	3.526896	-1.247216
H	0.065219	3.671434	-2.225560
C	1.040718	4.811533	-0.628973
H	1.680609	5.345833	-1.338027
H	1.652325	4.572645	0.249612

C	-0.129053	5.725326	-0.207153
H	-0.766621	5.926354	-1.079624
H	-0.760776	5.201002	0.520486
C	0.348183	7.056791	0.388327
H	0.978017	7.577667	-0.345071
H	0.993477	6.855001	1.254358
C	-0.805766	7.970676	0.813182
H	-1.449101	8.218329	-0.038847
H	-0.436808	8.911311	1.235447
H	-1.433874	7.488873	1.571315
C	2.946126	-1.513047	-1.304880
C	2.321307	-2.766979	-1.315122
C	3.786751	-1.169141	-2.369749
C	2.542130	-3.661142	-2.361565
H	1.652013	-3.050592	-0.508544
C	4.010092	-2.063136	-3.417698
H	4.253539	-0.188613	-2.388235
C	3.389312	-3.312103	-3.415433
H	2.048441	-4.628714	-2.356093
H	4.658064	-1.775986	-4.240443
H	3.555687	-4.006285	-4.234060
C	-3.642520	1.030216	0.015600
C	-4.226987	1.741002	-1.039167
C	-3.644650	1.598523	1.297097
C	-4.813799	2.988928	-0.818003
H	-4.201678	1.324200	-2.041963
C	-4.229529	2.843836	1.519788
H	-3.193647	1.066208	2.128822
C	-4.817979	3.542312	0.462133
H	-5.258628	3.528750	-1.648824
H	-4.227407	3.269543	2.519228
H	-5.271770	4.513708	0.636285

H -3.052155 -0.479962 -1.347569
H 3.089424 0.454052 -0.535358
Br -1.024212 0.292160 -3.275997
Br 2.450369 2.633759 -2.105036

[1]TS2

B3LYP/BSI SCF energy: -2949.028683a.u.

M06/BSII SCF energy in solution: -2947.863568a.u.

M06/BSII free energy in solution: -2947.028855a.u.

C	-0.544685	-1.919928	-0.903574
N	-0.954492	-0.926947	-0.199140
O	-1.482466	-2.776197	-1.346533
C	-2.795590	-2.110435	-1.136520
C	-2.418998	-1.093458	0.004536
H	-2.901677	-0.131481	-0.158391
C	-2.717665	-1.523868	1.432927
C	-3.742360	-0.878185	2.134920
C	-2.000367	-2.541118	2.075321
C	-4.054491	-1.247913	3.443858
H	-4.284380	-0.068194	1.657135
C	-2.307052	-2.909806	3.384670
H	-1.191556	-3.048876	1.558902
C	-3.337105	-2.265588	4.072862
H	-4.850403	-0.732753	3.973978
H	-1.738033	-3.698254	3.868799
H	-3.572421	-2.550222	5.094267
C	1.922859	-1.415481	-0.720155
N	1.805177	-0.355989	-0.004723
O	3.172093	-1.836919	-0.987794
C	4.072232	-1.092816	-0.065148
C	3.183116	0.164533	0.244988
H	3.249740	0.435434	1.296049
C	3.455214	1.416336	-0.571988
C	3.854312	2.581134	0.092381
C	3.317505	1.446586	-1.966134
C	4.125584	3.750203	-0.620724
H	3.929369	2.577829	1.175772
C	3.586591	2.612651	-2.680358
H	3.002813	0.556804	-2.502686
C	3.994101	3.768534	-2.008967
H	4.429143	4.646906	-0.088409
H	3.479078	2.619894	-3.761298
H	4.201970	4.677419	-2.566291
C	0.853219	-2.278547	-1.352039
C	0.925582	-2.084767	-2.895622
H	0.166939	-2.704279	-3.377766
H	0.747172	-1.040781	-3.167217
H	1.912308	-2.382332	-3.256955
C	1.126054	-3.765195	-0.994556
H	2.119718	-4.049051	-1.342093
H	1.084911	-3.923948	0.087101
H	0.381018	-4.402114	-1.473386
Ni	0.103252	0.492958	0.742358
Br	-0.121157	2.771653	-0.977507
Si	-1.953017	2.981040	0.521671
H	-1.200212	1.280708	1.115711
O	-2.471702	4.437479	-0.121799
O	-1.760853	3.428355	2.092103
C	-1.878015	5.702769	0.153046
H	-0.846365	5.721486	-0.227522
H	-1.835073	5.865566	1.237266
C	-2.698627	6.793025	-0.520727
H	-2.260363	7.777546	-0.323820

H	-2.729919	6.639466	-1.604024
H	-3.726726	6.791788	-0.144741
C	-1.898425	2.702763	3.315955
H	-1.795545	1.628704	3.136833
H	-1.067839	3.001065	3.960376
C	-3.237716	3.027086	3.966717
H	-4.072424	2.696482	3.339272
H	-3.315960	2.519173	4.934662
H	-3.341290	4.103830	4.133554
O	-3.240219	1.997597	0.110651
C	-4.580939	2.512839	-0.026084
H	-5.246407	1.724833	0.349481
H	-4.711632	3.392803	0.611296
C	-4.927011	2.855817	-1.467234
H	-4.807703	1.985256	-2.118412
H	-5.969655	3.189375	-1.528879
H	-4.282095	3.660858	-1.825605
C	5.380703	-0.801162	-0.778767
C	6.390475	-0.126984	-0.076906
C	5.619539	-1.194606	-2.098577
C	7.607016	0.161628	-0.690378
H	6.225103	0.168319	0.955183
C	6.844031	-0.911462	-2.710092
H	4.850615	-1.725065	-2.647813
C	7.839195	-0.230565	-2.011278
H	8.375989	0.690284	-0.134743
H	7.015350	-1.226176	-3.735640
H	8.789486	-0.008772	-2.487994
C	4.275524	-2.029033	1.136908
C	5.165313	-3.108044	1.002953
C	3.547394	-1.903266	2.329117
C	5.330346	-4.031578	2.032378
H	5.736288	-3.219879	0.086945
C	3.719314	-2.829781	3.362538
H	2.842559	-1.092107	2.481523
C	4.608411	-3.893440	3.220519
H	6.027405	-4.855597	1.908662
H	3.152035	-2.707078	4.280556
H	4.741689	-4.608036	4.027852
C	-3.825291	-3.156015	-0.746654
C	-5.167475	-2.766153	-0.631276
C	-3.481730	-4.486684	-0.491083
C	-6.142956	-3.687069	-0.255909
H	-5.449860	-1.739167	-0.844487
C	-4.462798	-5.411324	-0.123604
H	-2.448866	-4.801273	-0.582277
C	-5.793824	-5.015756	-0.001768
H	-7.177275	-3.367779	-0.167373
H	-4.180795	-6.442809	0.067451
H	-6.554863	-5.735235	0.285668
C	-3.114638	-1.456665	-2.488959
C	-3.572855	-2.276720	-3.534191
C	-2.863628	-0.104477	-2.759734
C	-3.781193	-1.760101	-4.810698
H	-3.769528	-3.326608	-3.342640
C	-3.074800	0.412478	-4.042216
H	-2.512301	0.569228	-1.987865
C	-3.533010	-0.409817	-5.069848
H	-4.140073	-2.411790	-5.602257
H	-2.877832	1.464330	-4.226468
H	-3.698062	-0.003866	-6.063760
Br	1.210177	1.225928	2.808997

[3]TS2

B3LYP/BSI SCF energy: -2949.037895a.u.

M06/BSII SCF energy in solution: -2947.865192a.u.

M06/BSII free energy in solution: -2947.034301a.u.

C	-0.546056	-1.687206	-1.153159
N	-0.925223	-0.964754	-0.164500
O	-1.479333	-2.462038	-1.732195
C	-2.800012	-2.037025	-1.200729
C	-2.360375	-1.229870	0.092329
H	-2.872273	-0.268759	0.132028
C	-2.568945	-1.906054	1.436027
C	-3.553339	-1.411135	2.298175
C	-1.803992	-3.004955	1.843718
C	-3.783136	-2.010329	3.537240
H	-4.133464	-0.541610	2.000699
C	-2.029226	-3.604276	3.081374
H	-1.017032	-3.388241	1.201572
C	-3.021725	-3.110822	3.930693
H	-4.547274	-1.610169	4.197449
H	-1.422839	-4.451723	3.387297
H	-3.191710	-3.575128	4.897773
C	1.924221	-1.118532	-1.031154
N	1.844519	-0.254073	-0.087762
O	3.155943	-1.496989	-1.411539
C	4.113612	-0.975231	-0.401058
C	3.221892	0.112627	0.328720
H	3.282005	-0.026544	1.408191
C	3.499420	1.577247	0.039826
C	3.841560	2.424586	1.100222
C	3.397355	2.115088	-1.250336
C	4.091253	3.780571	0.876346
H	3.901956	2.022082	2.107890
C	3.649079	3.466605	-1.476613
H	3.125675	1.475916	-2.084614
C	3.998591	4.303451	-0.413121
H	4.353118	4.424964	1.710555
H	3.571279	3.868119	-2.482757
H	4.192591	5.357308	-0.590775
C	0.807140	-1.755965	-1.838294
C	0.686180	-0.975141	-3.181858
H	-0.105489	-1.415402	-3.791919
H	0.447575	0.076248	-3.001423
H	1.630567	-1.039253	-3.728131
C	1.155628	-3.239760	-2.121502
H	2.100105	-3.300834	-2.662407
H	1.259078	-3.806219	-1.191506
H	0.367469	-3.693060	-2.723653
Ni	0.135121	0.437349	0.907257
Br	-0.471440	2.867757	-1.084234
Si	-2.004452	3.317274	0.609682
H	-1.049775	1.356738	1.362390
O	-2.721722	4.682080	-0.009988
O	-1.369405	3.874261	2.005763
C	-2.125711	5.979564	-0.015395
H	-1.237621	5.974096	-0.663187
H	-1.798052	6.239948	0.998418
C	-3.141472	6.988891	-0.528082
H	-2.705862	7.993783	-0.541828
H	-3.459155	6.736477	-1.544489
H	-4.027435	7.005576	0.114265
C	-0.812452	3.300034	3.196202
H	-0.075171	2.537809	2.936780
H	-0.301365	4.125223	3.704140
C	-1.891942	2.701337	4.083407

H	-2.364626	1.851251	3.583967
H	-1.446249	2.338035	5.015053
H	-2.656152	3.447169	4.327711
O	-3.249119	2.224885	0.589211
C	-4.600279	2.623774	0.905572
H	-5.024513	1.809964	1.505213
H	-4.595479	3.525355	1.527574
C	-5.428354	2.857434	-0.348387
H	-5.443441	1.964598	-0.980075
H	-6.461023	3.102475	-0.074009
H	-5.012765	3.689115	-0.921955
C	5.332956	-0.426288	-1.124301
C	6.372491	0.132253	-0.365987
C	5.463116	-0.478909	-2.515121
C	7.509105	0.641524	-0.988850
H	6.291899	0.166695	0.716393
C	6.606600	0.028549	-3.138892
H	4.672612	-0.919753	-3.111026
C	7.630611	0.592286	-2.380207
H	8.301805	1.076032	-0.386907
H	6.692669	-0.021058	-4.220689
H	8.518067	0.987780	-2.865527
C	4.458765	-2.185171	0.481630
C	5.501864	-3.043732	0.098598
C	3.695190	-2.520619	1.610577
C	5.785996	-4.195545	0.830307
H	6.099600	-2.808349	-0.775152
C	3.984155	-3.674948	2.342736
H	2.870583	-1.899533	1.947826
C	5.029353	-4.514322	1.959426
H	6.601888	-4.841382	0.518182
H	3.385064	-3.906881	3.218472
H	5.254117	-5.408101	2.534637
C	-3.634907	-3.277573	-0.923380
C	-4.982019	-3.119058	-0.567262
C	-3.104424	-4.568469	-1.004375
C	-5.776702	-4.228199	-0.287282
H	-5.411750	-2.123039	-0.515343
C	-3.904927	-5.680771	-0.731466
H	-2.066571	-4.705506	-1.284313
C	-5.240758	-5.515687	-0.369674
H	-6.816748	-4.086641	-0.008217
H	-3.478176	-6.677197	-0.802847
H	-5.861440	-6.380829	-0.155669
C	-3.404514	-1.180116	-2.322866
C	-3.921290	-1.837699	-3.453060
C	-3.369499	0.220035	-2.322714
C	-4.398174	-1.118082	-4.545469
H	-3.951233	-2.922207	-3.472223
C	-3.852527	0.941548	-3.419991
H	-2.977610	0.777550	-1.480910
C	-4.367509	0.278761	-4.531924
H	-4.796328	-1.648274	-5.405971
H	-3.820440	2.026500	-3.393686
H	-4.742889	0.843058	-5.380810
Br	0.960411	-0.258488	3.163117

^{1}TS3

B3LYP/BSI SCF energy: -5391.345679 a.u.

M06/BSII SCF energy in solution: -5390.217748 a.u.

M06/BSII free energy in solution: -5389.37335 a.u.

C	-1.020361	1.775899	-1.276901
N	-0.378678	1.202661	-0.325504

O	-0.518470	2.943915	-1.718398
C	0.816764	3.098562	-1.098150
C	0.677829	2.149140	0.148062
H	1.589991	1.582401	0.333530
C	0.244147	2.795795	1.456300
C	0.994205	2.544546	2.610207
C	-0.895260	3.608538	1.545670
C	0.622162	3.114340	3.830131
H	1.849814	1.878641	2.544134
C	-1.265833	4.175049	2.763412
H	-1.492157	3.817698	0.662982
C	-0.504588	3.931424	3.910207
H	1.209806	2.907291	4.719884
H	-2.146087	4.809773	2.815991
H	-0.793444	4.373710	4.859673
C	-3.128802	0.432713	-1.072787
N	-2.720042	-0.403262	-0.189942
O	-4.454023	0.532447	-1.247803
C	-5.102476	-0.195521	-0.119919
C	-3.900127	-1.087218	0.398125
H	-3.821782	-1.001708	1.481196
C	-3.913577	-2.566552	0.057784
C	-3.936849	-3.498647	1.101477
C	-3.889403	-3.030144	-1.263556
C	-3.949586	-4.868986	0.832580
H	-3.932705	-3.149301	2.130527
C	-3.905474	-4.396171	-1.534899
H	-3.856282	-2.324406	-2.087188
C	-3.936950	-5.320274	-0.487044
H	-3.964866	-5.579310	1.654163
H	-3.888473	-4.740675	-2.564756
H	-3.947745	-6.385404	-0.700351

C	-2.284077	1.310545	-1.988105
C	-1.880802	0.473451	-3.237365
H	-1.239184	1.074832	-3.888293
H	-1.360452	-0.441706	-2.944426
H	-2.779560	0.201338	-3.798232
C	-3.103144	2.544746	-2.436599
H	-4.004785	2.217598	-2.954789
H	-3.405691	3.159140	-1.584900
H	-2.505900	3.156340	-3.113526
Ni	-0.784697	-0.640120	0.461731
Br	-0.148067	-2.397824	-1.236612
Si	3.296874	-1.061530	1.113105
H	1.775162	-0.697687	0.964335
O	4.870575	-1.362694	1.855863
O	2.764563	-2.658282	1.660063
C	5.269344	-2.471860	2.632256
H	5.653822	-3.272400	1.976190
H	4.422130	-2.903061	3.170051
C	6.337048	-2.061280	3.650567
H	6.645604	-2.925395	4.250688
H	7.244719	-1.648790	3.185437
H	5.949860	-1.292065	4.326329
C	1.521004	-2.986772	2.234872
H	0.749806	-2.267293	1.934568
H	1.199664	-3.967863	1.843216
C	1.575574	-3.051636	3.759493
H	1.847748	-2.075650	4.174529
H	0.595408	-3.325920	4.164612
H	2.311464	-3.789593	4.098929
O	3.453113	0.646411	1.500308
C	4.559709	1.334527	2.062260
H	4.160977	2.181984	2.641620

H	5.094708	0.682765	2.757962
C	5.533985	1.864863	1.005140
H	5.098617	2.689499	0.429888
H	6.447843	2.247163	1.479677
H	5.793565	1.080285	0.286457
C	-6.289583	-0.972846	-0.662572
C	-7.067170	-1.724826	0.230487
C	-6.640082	-0.954597	-2.015687
C	-8.162224	-2.454547	-0.225244
H	-6.815046	-1.736891	1.286706
C	-7.742949	-1.682798	-2.470700
H	-6.052827	-0.370103	-2.714139
C	-8.504838	-2.436502	-1.579851
H	-8.749858	-3.036448	0.478684
H	-8.003264	-1.656864	-3.525042
H	-9.360401	-3.003679	-1.934541
C	-5.521945	0.904150	0.867598
C	-6.764169	1.539038	0.707241
C	-4.653985	1.373300	1.866423
C	-7.137053	2.599882	1.530944
H	-7.446079	1.199044	-0.064433
C	-5.030322	2.437258	2.689913
H	-3.677479	0.928190	2.027960
C	-6.271630	3.051658	2.529005
H	-8.106116	3.070972	1.392749
H	-4.340584	2.778442	3.456008
H	-6.563709	3.874866	3.174919
C	1.049921	4.576408	-0.809061
C	2.177452	4.956316	-0.067787
C	0.200783	5.568190	-1.310728
C	2.441621	6.302073	0.176035
H	2.848277	4.200256	0.327600
C	0.469758	6.917192	-1.066556
H	-0.673787	5.289123	-1.886536
C	1.588539	7.289140	-0.323149
H	3.315334	6.578797	0.758610
H	-0.200593	7.675799	-1.460689
H	1.796125	8.338133	-0.133415
C	1.848379	2.616992	-2.132855
C	1.604716	2.830344	-3.500602
C	3.071946	2.052044	-1.745903
C	2.561583	2.478988	-4.455187
H	0.670019	3.281639	-3.815716
C	4.035978	1.700666	-2.700991
H	3.281906	1.832676	-0.704667
C	3.779815	1.915717	-4.058001
H	2.356041	2.651586	-5.508481
H	4.936498	1.176606	-2.380385
H	4.520118	1.630975	-4.800128
O	4.095899	-2.308580	-2.739371
P	4.895508	-1.867277	-1.497767
O	5.467528	-3.055768	-0.688221
O	5.883175	-0.699552	-1.647492
O	3.611396	-1.264651	-0.538597
K	2.130790	-0.735020	-2.773939
K	7.232274	-1.498435	0.380596
K	2.989482	-4.041453	-0.679114
Br	-1.329786	-0.509429	2.821968

^{3}TS3

B3LYP/BSI SCF energy: -5391.370895 a.u.

M06/BSII SCF energy in solution: -5390.24287 a.u.

M06/BSII free energy in solution: -5389.399574 a.u.

C	-1.014711	1.772281	-1.280020
N	-0.369857	1.200064	-0.328787
O	-0.518650	2.943883	-1.715587
C	0.814997	3.104738	-1.092019
C	0.675439	2.156328	0.153227
H	1.590249	1.596776	0.347786
C	0.223543	2.800837	1.456743
C	0.955050	2.543478	2.621131
C	-0.914866	3.616648	1.531889
C	0.567010	3.110897	3.837123
H	1.807996	1.873505	2.566197
C	-1.301477	4.180803	2.745734
H	-1.498085	3.830861	0.641405
C	-0.557843	3.931756	3.902868
H	1.140395	2.898622	4.734868
H	-2.180434	4.818091	2.787049
H	-0.859062	4.372537	4.849185
C	-3.121914	0.427669	-1.080105
N	-2.710203	-0.410590	-0.201811
O	-4.447817	0.532825	-1.249658
C	-5.092471	-0.196718	-0.120590
C	-3.889379	-1.091973	0.389715
H	-3.805715	-1.009746	1.472753
C	-3.907731	-2.570637	0.045973
C	-3.931277	-3.505333	1.087367
C	-3.886875	-3.031134	-1.276526
C	-3.947563	-4.874991	0.815092
H	-3.924332	-3.158496	2.117252
C	-3.906443	-4.396464	-1.551246
H	-3.853551	-2.323412	-2.098473
C	-3.938191	-5.323100	-0.505639
H	-3.962972	-5.587294	1.634968

H	-3.891985	-4.738477	-2.581987
H	-3.951660	-6.387680	-0.721553
C	-2.275724	1.304411	-1.995214
C	-1.866911	0.465989	-3.241696
H	-1.223352	1.066805	-3.891345
H	-1.347766	-0.448885	-2.945816
H	-2.763541	0.192840	-3.805446
C	-3.094092	2.536995	-2.449138
H	-3.994917	2.207107	-2.967021
H	-3.398274	3.154337	-1.600222
H	-2.496727	3.146435	-3.127922
Ni	-0.749607	-0.641911	0.457832
Br	-0.131927	-2.409411	-1.250120
Si	3.276172	-1.070710	1.112174
H	1.747597	-0.719227	0.946675
O	4.856774	-1.363793	1.847961
O	2.758991	-2.670264	1.652766
C	5.262776	-2.469019	2.625598
H	5.658065	-3.265718	1.970956
H	4.417741	-2.909621	3.159157
C	6.321810	-2.050091	3.649835
H	6.637727	-2.912973	4.247948
H	7.226514	-1.625159	3.190131
H	5.923372	-1.288068	4.327199
C	1.520177	-3.016807	2.228626
H	0.738490	-2.309090	1.929467
H	1.214394	-4.002872	1.837271
C	1.577155	-3.080721	3.753063
H	1.835099	-2.100689	4.167481
H	0.601220	-3.368676	4.158855
H	2.323640	-3.808141	4.092017
O	3.407793	0.630463	1.529003

C	4.505133	1.319663	2.108742
H	4.095051	2.160074	2.690182
H	5.035839	0.665013	2.804709
C	5.488027	1.863897	1.067149
H	5.053845	2.689926	0.492952
H	6.393605	2.248525	1.555298
H	5.761582	1.086040	0.346581
C	-6.283666	-0.970305	-0.659725
C	-7.059521	-1.721938	0.235122
C	-6.639244	-0.949422	-2.011475
C	-8.157831	-2.448682	-0.217544
H	-6.803437	-1.736041	1.290364
C	-7.745360	-1.674596	-2.463420
H	-6.053273	-0.365173	-2.711216
C	-8.505525	-2.427965	-1.570814
H	-8.744046	-3.030355	0.487764
H	-8.009564	-1.646585	-3.516751
H	-9.363643	-2.992790	-1.923096
C	-5.505375	0.901125	0.871736
C	-6.745197	1.541773	0.715402
C	-4.634324	1.362631	1.871378
C	-7.112913	2.600669	1.543839
H	-7.429311	1.207619	-0.056908
C	-5.005592	2.424609	2.699808
H	-3.659267	0.913222	2.029933
C	-6.244576	3.044647	2.542933
H	-8.080210	3.076277	1.408684
H	-4.313460	2.759717	3.466373
H	-6.532678	3.866219	3.192728
C	1.041556	4.583740	-0.804225
C	2.160328	4.968018	-0.052136
C	0.196572	5.572233	-1.319288
C	2.419754	6.315110	0.189517
H	2.827950	4.214396	0.353248
C	0.460714	6.922518	-1.077095
H	-0.671067	5.289688	-1.903869
C	1.570637	7.298911	-0.322693
H	3.286592	6.595422	0.780569
H	-0.206380	7.678638	-1.481368
H	1.774457	8.348915	-0.134522
C	1.850572	2.627188	-2.124510
C	1.611237	2.845218	-3.492346
C	3.073366	2.062276	-1.735537
C	2.571888	2.498651	-4.444846
H	0.677114	3.296558	-3.809154
C	4.041289	1.715694	-2.688383
H	3.279907	1.839388	-0.694573
C	3.789517	1.935612	-4.045406
H	2.369790	2.674883	-5.498204
H	4.940885	1.190931	-2.366079
H	4.532749	1.654542	-4.785994
O	4.100928	-2.280458	-2.746024
P	4.891715	-1.846580	-1.496223
O	5.463134	-3.040080	-0.693908
O	5.877232	-0.675202	-1.631830
O	3.600117	-1.254166	-0.540038
K	2.129427	-0.718723	-2.783717
K	7.227107	-1.490533	0.386506
K	2.991644	-4.036428	-0.700406
Br	-1.309245	-0.533541	2.825455

^{1}[Ni^{II}]BrH

B3LYP/BSI SCF energy: -2182.645637a.u.

M06/BSII SCF energy in solution: -2181.670491a.u.

M06/BSII free energy in solution: -2181.030793a.u.

C	1.329783	0.055528	0.972772
N	1.476659	-0.377814	-0.228160
O	2.408753	0.637371	1.535416
C	3.376380	0.884360	0.451371
C	2.894350	-0.168887	-0.621392
C	-1.160974	-0.061761	1.039638
N	-1.280916	-0.332300	-0.206920
O	-2.286013	0.221187	1.722664
C	-3.416541	-0.155710	0.824851
C	-2.699690	-0.113250	-0.576409
Ni	0.164938	-1.166398	-1.356382
C	0.110696	-0.077129	1.866349
C	0.074936	1.070632	2.901836
H	0.960846	1.026075	3.537218
H	0.054466	2.048973	2.413660
H	-0.814401	0.972751	3.525459
C	0.184976	-1.448167	2.602372
H	-0.696623	-1.568538	3.236965
H	0.218483	-2.273070	1.886806
H	1.077791	-1.482591	3.233412
C	-3.825009	-1.568012	1.268717
C	-4.575997	-1.706943	2.448318
C	-3.410173	-2.727067	0.596293
C	-4.912186	-2.966538	2.938113
H	-4.902963	-0.819519	2.980799
C	-3.753607	-3.990176	1.088447
H	-2.819307	-2.676457	-0.312610
C	-4.503741	-4.115657	2.255982
H	-5.497822	-3.050670	3.849269
H	-3.428107	-4.873613	0.547165

H	-4.771136	-5.099033	2.632328
C	-4.539979	0.850367	1.002188
C	-5.753986	0.626093	0.336652
C	-4.404054	1.992837	1.796406
C	-6.804747	1.532770	0.455417
H	-5.878778	-0.265903	-0.270338
C	-5.462238	2.896897	1.921485
H	-3.472737	2.172745	2.320123
C	-6.663251	2.672886	1.250474
H	-7.736751	1.345965	-0.070046
H	-5.342573	3.777426	2.546435
H	-7.484446	3.377099	1.347016
C	4.783238	0.688776	1.005362
C	5.886957	0.988746	0.195143
C	5.005749	0.227899	2.307147
C	7.184575	0.820333	0.672973
H	5.734241	1.365733	-0.811256
C	6.307566	0.068842	2.787971
H	4.162627	-0.002329	2.947361
C	7.400677	0.360963	1.974051
H	8.027510	1.054012	0.029243
H	6.462683	-0.285712	3.802997
H	8.412215	0.235377	2.348760
C	3.172280	2.344065	0.008569
C	2.917848	3.313414	0.991996
C	3.297637	2.761187	-1.321708
C	2.771193	4.655950	0.650538
H	2.835969	3.009634	2.029954
C	3.155813	4.109155	-1.663501
H	3.507779	2.049692	-2.113100
C	2.888103	5.060163	-0.681463
H	2.570510	5.388367	1.427286

H	3.250367	4.407942	-2.703222
H	2.774227	6.106461	-0.949054
C	-2.848405	1.158457	-1.397192
C	-3.577841	1.114924	-2.590833
C	-2.269080	2.372428	-1.005741
C	-3.739620	2.261574	-3.369988
H	-4.004824	0.172528	-2.921283
C	-2.428044	3.519296	-1.782464
H	-1.684698	2.427361	-0.092211
C	-3.166529	3.467672	-2.966771
H	-4.303791	2.207179	-4.296510
H	-1.972474	4.452753	-1.464098
H	-3.286866	4.360276	-3.574124
H	-3.021884	-0.944665	-1.200842
H	2.895506	0.270769	-1.615808
C	3.664354	-1.475492	-0.687416
C	4.459434	-1.740058	-1.807399
C	3.612608	-2.422903	0.342239
C	5.193335	-2.923199	-1.899261
H	4.491268	-1.023014	-2.623863
C	4.342609	-3.606892	0.252771
H	2.992532	-2.245392	1.215292
C	5.136655	-3.860284	-0.867780
H	5.797871	-3.115808	-2.780684
H	4.287927	-4.335234	1.056771
H	5.700463	-4.785817	-0.938777
H	1.213556	-1.763189	-2.136277
Br	-1.277011	-2.324161	-2.825239

^{3}[Ni^{II}]BrH

B3LYP/BSI SCF energy: -2182.645627a.u.

M06/BSII SCF energy in solution: -2181.659343a.u.

M06/BSII free energy in solution: -2181.025069a.u.

C	1.204308	0.195670	0.922345
N	1.436948	-0.224781	-0.262416
O	2.196596	0.863301	1.537696
C	3.305666	1.036299	0.583741
C	2.786152	0.198102	-0.675819
C	-1.304402	0.007871	0.891207
N	-1.410955	-0.265775	-0.355516
O	-2.436690	0.279439	1.559421
C	-3.578246	-0.017608	0.655117
C	-2.831985	-0.171184	-0.745758
Ni	0.075931	-1.068959	-1.555561
C	-0.050132	-0.027214	1.754514
C	-0.150563	1.037202	2.870504
H	0.721558	0.973729	3.522626
H	-0.195396	2.049503	2.459233
H	-1.048578	0.860876	3.463073
C	0.031208	-1.450421	2.386972
H	-0.862984	-1.630791	2.989253
H	0.098680	-2.220682	1.614566
H	0.907156	-1.512085	3.039511
C	-4.173479	-1.334118	1.174833
C	-5.212854	-1.313003	2.118039
C	-3.628001	-2.575069	0.808265
C	-5.709727	-2.497407	2.661081
H	-5.640060	-0.365734	2.426995
C	-4.126886	-3.759345	1.353699
H	-2.800980	-2.650230	0.108878
C	-5.171558	-3.726599	2.277185
H	-6.519480	-2.456812	3.384129
H	-3.689213	-4.705381	1.049256

H -5.561532 -4.649868 2.696221
C -4.557097 1.144427 0.725661
C -5.747947 1.077170 -0.013351
C -4.311182 2.277708 1.506533
C -6.664305 2.125330 0.020370
H -5.960799 0.198430 -0.614322
C -5.233429 3.327359 1.542749
H -3.400950 2.338063 2.091021
C -6.410030 3.257076 0.799439
H -7.578662 2.057365 -0.561750
H -5.027535 4.199161 2.157343
H -7.125804 4.073446 0.827255
C 4.576026 0.502369 1.249655
C 5.833551 0.812668 0.714189
C 4.512535 -0.314279 2.384562
C 6.996645 0.307190 1.292096
H 5.909281 1.457448 -0.154740
C 5.678804 -0.812904 2.968238
H 3.550827 -0.553808 2.822004
C 6.924962 -0.507456 2.423425
H 7.961033 0.556673 0.859300
H 5.608064 -1.441321 3.851474
H 7.831957 -0.896690 2.876509
C 3.415791 2.540221 0.308451
C 3.156918 3.447257 1.347050
C 3.830875 3.043325 -0.930535
C 3.291153 4.819369 1.144103
H 2.845215 3.073532 2.315966
C 3.974436 4.418660 -1.131140
H 4.050197 2.371991 -1.754287
C 3.700828 5.311777 -0.096983
H 3.078124 5.505189 1.959136
H 4.295022 4.786392 -2.101409
H 3.805987 6.381073 -0.254754
C -3.049652 0.910164 -1.788399
C -3.793333 0.613433 -2.935383
C -2.514257 2.195669 -1.642705
C -4.011481 1.584086 -3.914683
H -4.195938 -0.387635 -3.069371
C -2.728722 3.166212 -2.619103
H -1.920293 2.438065 -0.766878
C -3.480248 2.863922 -3.757410
H -4.586523 1.335585 -4.801954
H -2.304773 4.158478 -2.494358
H -3.643027 3.620197 -4.519906
H -3.121094 -1.123313 -1.194299
H 2.648293 0.873813 -1.521614
C 3.638153 -0.965069 -1.146354
C 4.472834 -0.796843 -2.257886
C 3.608916 -2.208516 -0.503802
C 5.280326 -1.841637 -2.707960
H 4.482051 0.153245 -2.786969
C 4.410050 -3.256329 -0.956262
H 2.940337 -2.369488 0.335099
C 5.251732 -3.074558 -2.055074
H 5.918692 -1.695919 -3.574570
H 4.367110 -4.218709 -0.455404
H 5.871483 -3.893486 -2.408491
H 0.248263 -0.090318 -2.747041
Br -0.314202 -3.480086 -1.199855

BrK_3PO_4[Si]

B3LYP/BSI SCF energy: -3208.752259a.u.

M06/BSII SCF energy in solution: -3208.585194a.u.

M06/BSII free energy in solution: -3208.414987a.u.

Br	-4.175565	0.537597	0.381420
O	1.412013	-2.130324	-1.182153
P	0.140546	-1.666137	-0.479825
O	-1.215664	-1.849619	-1.171699
O	0.103004	-2.031967	1.024723
O	0.266908	0.070197	-0.483491
K	2.668914	-2.444028	1.023684
K	-2.537379	-2.077066	1.132977
K	-2.040880	0.444287	-1.931650
Si	1.488364	1.083197	-0.109875
O	2.735919	0.296042	0.692599
O	0.978601	2.267443	0.909248
C	3.762302	1.094653	1.305377
H	3.304691	1.797302	2.010541
H	4.281309	1.681461	0.536543
C	4.750619	0.189156	2.025078
H	5.547137	0.782194	2.485516
H	4.256025	-0.374121	2.825913
H	5.223184	-0.512629	1.326853
C	-0.329403	2.850428	0.916577
H	-0.463848	3.455961	0.007831
H	-1.092477	2.062298	0.920495
C	-0.477600	3.728457	2.147805
H	0.272579	4.525220	2.148484
H	-1.473269	4.182886	2.167611
H	-0.351956	3.134091	3.057937
O	2.088778	1.838325	-1.449591
C	2.899195	1.237567	-2.465590
H	3.384132	2.065908	-2.994572
H	3.691687	0.634372	-2.000739
C	2.098051	0.376488	-3.435442
H	1.282092	0.961934	-3.876196
H	2.745909	0.039986	-4.253492
H	1.694387	-0.507395	-2.931924

HBr

B3LYP/BSI SCF energy: -13.97872a.u.

M06/BSII SCF energy in solution: -13.953162a.u.

M06/BSII free energy in solution: -13.966665a.u.

Br	0.000000	0.000000	0.039674
H	0.000000	0.000000	-1.388590

^{1}TS4

B3LYP/BSI SCF energy: -5378.498223 a.u.

M06/BSII SCF energy in solution: -5377.392703 a.u.

M06/BSII free energy in solution: -5376.541218 a.u.

C	-0.931146	1.988218	-1.182150
N	-0.285981	1.412477	-0.246109
O	-0.470449	3.208937	-1.588554
C	0.829590	3.391571	-0.914387
C	0.670278	2.408381	0.297521
H	1.601191	1.893836	0.529337
C	0.088208	2.958143	1.598476
C	0.474295	2.349480	2.799453
C	-0.869788	3.981809	1.636614
C	-0.048123	2.788515	4.017806
H	1.153286	1.504059	2.762912
C	-1.387202	4.423066	2.854468
H	-1.205638	4.453581	0.720256
C	-0.969872	3.835641	4.051228

H	0.264331	2.306352	4.940043
H	-2.115992	5.228600	2.866401
H	-1.365539	4.188480	4.999991
C	-2.912563	0.480838	-0.945666
N	-2.406306	-0.450471	-0.222308
O	-4.257590	0.598420	-0.934471
C	-4.768159	-0.250234	0.163326
C	-3.510603	-1.175844	0.448484
H	-3.296432	-1.169717	1.516787
C	-3.604467	-2.630083	0.024968
C	-3.684765	-3.616303	1.015565
C	-3.630607	-3.020435	-1.318543
C	-3.803577	-4.965022	0.675667
H	-3.651341	-3.325143	2.062741
C	-3.750625	-4.365651	-1.662056
H	-3.539244	-2.276446	-2.101890
C	-3.840105	-5.342083	-0.667205
H	-3.864676	-5.716544	1.457941
H	-3.764013	-4.652121	-2.709522
H	-3.932800	-6.390148	-0.938270
C	-2.202654	1.491183	-1.854672
C	-1.864485	0.809835	-3.209329
H	-1.321748	1.513697	-3.848176
H	-1.270932	-0.095767	-3.061956
H	-2.791533	0.533424	-3.720913
C	-3.126858	2.707445	-2.111890
H	-4.044826	2.378905	-2.600221
H	-3.398851	3.211452	-1.181290
H	-2.616227	3.421298	-2.759972
Ni	-0.464212	-0.788322	0.322108
Br	-0.132998	-2.358193	-1.959647
Si	2.761776	-1.558814	1.339943
H	1.066482	-1.045250	0.779887
O	4.259328	-2.045677	2.101958
O	2.033398	-3.076285	1.535722
C	4.572145	-3.326474	2.611901
H	5.103777	-3.907170	1.839118
H	3.663002	-3.887666	2.844921
C	5.423926	-3.215818	3.878445
H	5.684537	-4.212738	4.252518
H	6.365524	-2.671596	3.714341
H	4.881592	-2.682767	4.665625
C	0.743094	-3.442334	2.012620
H	-0.033106	-2.920612	1.439995
H	0.639169	-4.519863	1.824769
C	0.566776	-3.144487	3.496245
H	0.621445	-2.065843	3.670789
H	-0.414978	-3.493467	3.836942
H	1.336220	-3.641839	4.097307
O	2.563331	-0.162249	2.273871
C	3.554754	0.412385	3.125772
H	3.018397	0.970027	3.904926
H	4.139325	-0.371069	3.613287
C	4.476936	1.359157	2.359303
H	3.917405	2.222111	1.980220
H	5.267589	1.743575	3.016527
H	4.934663	0.855114	1.500982
C	-6.007446	-0.983017	-0.325373
C	-6.715892	-1.797784	0.570589
C	-6.466075	-0.871051	-1.641189
C	-7.849193	-2.492331	0.155057
H	-6.379804	-1.885589	1.599307
C	-7.606475	-1.564292	-2.056166
H	-5.932530	-0.238621	-2.340637

C	-8.300219	-2.378070	-1.162498
H	-8.381599	-3.122508	0.861602
H	-7.949811	-1.463803	-3.081976
H	-9.185577	-2.917688	-1.486034
C	-5.071919	0.720071	1.317036
C	-6.338227	1.318323	1.412568
C	-4.079564	1.109070	2.231473
C	-6.614941	2.256907	2.405736
H	-7.115653	1.043590	0.708337
C	-4.358706	2.049130	3.225908
H	-3.075004	0.700273	2.180329
C	-5.626510	2.622140	3.320782
H	-7.605259	2.699971	2.463743
H	-3.572309	2.334673	3.917094
H	-5.842299	3.349359	4.098630
C	1.031337	4.874287	-0.624832
C	2.017228	5.277990	0.285430
C	0.303380	5.852604	-1.312513
C	2.262571	6.631172	0.510640
H	2.589887	4.535158	0.831967
C	0.549062	7.208161	-1.083446
H	-0.463395	5.554388	-2.018293
C	1.528934	7.602937	-0.173132
H	3.025580	6.925237	1.225395
H	-0.028828	7.954713	-1.620962
H	1.719774	8.657355	0.003632
C	1.936385	2.941301	-1.888076
C	1.747960	3.077693	-3.272858
C	3.172667	2.464176	-1.425577
C	2.766680	2.733921	-4.166507
H	0.803270	3.455284	-3.648564
C	4.188665	2.100723	-2.316327
H	3.357136	2.330249	-0.365935
C	3.987261	2.241160	-3.692481
H	2.601746	2.846850	-5.234803
H	5.088755	1.631881	-1.927144
H	4.767474	1.947301	-4.388036
O	4.093401	-1.738360	-2.545771
P	4.683746	-1.650817	-1.132947
O	5.148388	-3.018351	-0.581038
O	5.635682	-0.491066	-0.811190
O	3.235269	-1.306739	-0.236856
K	2.081509	-0.239708	-2.581900
K	6.811217	-1.825137	1.005659
K	2.651485	-3.912543	-1.217164
H	-0.848086	-0.750783	1.776602

^{3}TS4

B3LYP/BSI SCF energy: -5378.508531 a.u.

M06/BSII SCF energy in solution: -5377.397761 a.u.

M06/BSII free energy in solution: -5376.546104 a.u.

C	-0.909219	1.922772	-1.188908
N	-0.280245	1.373880	-0.216922
O	-0.435437	3.119446	-1.607540
C	0.865736	3.312257	-0.925968
C	0.679836	2.379967	0.318201
H	1.597178	1.857566	0.584085
C	0.085582	2.988274	1.585735
C	0.511598	2.485943	2.821393
C	-0.916984	3.969071	1.562171
C	-0.025042	2.980805	4.011544
H	1.234864	1.677604	2.835088
C	-1.451151	4.464168	2.751703

H	-1.274742	4.367099	0.619040
C	-1.000934	3.977353	3.981645
H	0.316840	2.579605	4.961470
H	-2.215850	5.234954	2.715928
H	-1.411287	4.370993	4.907728
C	-2.927084	0.473219	-0.981080
N	-2.451774	-0.430526	-0.207069
O	-4.261422	0.633880	-1.000501
C	-4.833312	-0.192536	0.089502
C	-3.568073	-1.064395	0.525443
H	-3.379500	-0.901181	1.587208
C	-3.644629	-2.565116	0.323288
C	-3.868991	-3.381080	1.440337
C	-3.509618	-3.161933	-0.934643
C	-3.980908	-4.765116	1.303067
H	-3.955035	-2.928193	2.425443
C	-3.617723	-4.545137	-1.074007
H	-3.287858	-2.554329	-1.804135
C	-3.859393	-5.349804	0.041281
H	-4.155860	-5.383336	2.179195
H	-3.498835	-4.993379	-2.055704
H	-3.942438	-6.427283	-0.071224
C	-2.147092	1.404404	-1.909335
C	-1.722159	0.612376	-3.177476
H	-1.100976	1.247320	-3.817304
H	-1.183840	-0.303396	-2.916683
H	-2.614650	0.330716	-3.744246
C	-3.025765	2.608938	-2.323164
H	-3.923218	2.251965	-2.829362
H	-3.336930	3.200530	-1.458845
H	-2.467164	3.251824	-3.004857
Ni	-0.477036	-0.544892	0.526584
Br	-0.087261	-2.649083	-2.024821
Si	2.746017	-1.410187	1.317638
H	1.198689	-0.877514	0.784651
O	4.201779	-1.937301	2.148801
O	1.987198	-2.926363	1.571931
C	4.471924	-3.212589	2.693138
H	4.999953	-3.827067	1.944152
H	3.544044	-3.740497	2.929075
C	5.307735	-3.093523	3.970154
H	5.523126	-4.086980	4.380628
H	6.273483	-2.592717	3.806949
H	4.772983	-2.514297	4.729517
C	0.658516	-3.250909	1.925436
H	-0.063335	-2.674780	1.325092
H	0.501798	-4.305153	1.658097
C	0.376412	-3.034427	3.407055
H	0.461828	-1.973187	3.658546
H	-0.642165	-3.357922	3.649421
H	1.077965	-3.605377	4.025075
O	2.686992	0.076701	2.159877
C	3.709953	0.621989	2.989795
H	3.214767	1.262926	3.732482
H	4.228686	-0.174546	3.527666
C	4.710337	1.452405	2.185561
H	4.235694	2.353658	1.779994
H	5.540820	1.782416	2.823558
H	5.105924	0.880432	1.338739
C	-5.991817	-0.997008	-0.480640
C	-6.780530	-1.772808	0.382571
C	-6.288332	-0.999876	-1.847083
C	-7.835255	-2.536310	-0.112018
H	-6.569417	-1.777312	1.447255

C	-7.348604	-1.763629	-2.342263
H	-5.691807	-0.401607	-2.525288
C	-8.124362	-2.534765	-1.478829
H	-8.431716	-3.133456	0.571585
H	-7.564665	-1.751860	-3.406817
H	-8.947518	-3.129268	-1.864354
C	-5.260955	0.817645	1.165145
C	-6.572425	1.315291	1.195277
C	-4.324429	1.351040	2.066206
C	-6.945930	2.294875	2.115424
H	-7.308712	0.932617	0.497840
C	-4.698682	2.333473	2.984246
H	-3.290645	1.018688	2.063959
C	-6.011249	2.804903	3.017072
H	-7.969696	2.658258	2.124718
H	-3.952637	2.729409	3.666222
H	-6.302565	3.565382	3.736177
C	1.072840	4.800081	-0.674387
C	2.076213	5.215786	0.210976
C	0.333902	5.767168	-1.365317
C	2.327141	6.571883	0.409707
H	2.659718	4.480290	0.756101
C	0.586412	7.125894	-1.163291
H	-0.446084	5.460029	-2.052561
C	1.582582	7.533453	-0.276874
H	3.103865	6.876324	1.104969
H	0.000483	7.864603	-1.702812
H	1.778176	8.590243	-0.120974
C	1.964371	2.820104	-1.887443
C	1.794143	2.980048	-3.272909
C	3.175867	2.291384	-1.418559
C	2.808836	2.611659	-4.160345
H	0.869340	3.398487	-3.655106
C	4.190874	1.909518	-2.303840
H	3.343067	2.130714	-0.360178
C	4.008310	2.075045	-3.679407
H	2.658649	2.743359	-5.228701
H	5.070397	1.405554	-1.907818
H	4.786877	1.766186	-4.370379
O	4.187178	-1.815367	-2.526677
P	4.744700	-1.674348	-1.104681
O	5.195423	-3.018483	-0.485023
O	5.700950	-0.508942	-0.807166
O	3.283304	-1.288433	-0.262991
K	2.083353	-0.434978	-2.614918
K	6.783364	-1.763147	1.126164
K	2.747408	-3.972699	-1.104796
H	-0.936467	-0.374695	2.015333

^{1}TS4a

B3LYP/BSI SCF energy: -2418.491324a.u.

M06/BSII SCF energy in solution: -2417.376192a.u.

M06/BSII free energy in solution: -2416.58007a.u.

C	-1.318081	-1.335794	0.452540
N	-1.440433	-0.256875	-0.227607
O	-2.451877	-1.903220	0.904260
C	-3.607742	-1.148413	0.376197
C	-2.875698	0.070784	-0.357254
C	1.208232	-1.344695	0.634752
N	1.463575	-0.395910	-0.177681
O	2.213237	-1.757684	1.448545
C	3.311302	-0.794283	1.320824
C	2.865861	0.007386	0.007166

Ni	-0.095462	0.744255	-1.296509
C	-0.072115	-2.155110	0.759276
C	-0.187469	-2.755394	2.181229
H	-1.098757	-3.348935	2.257085
H	-0.212853	-1.973958	2.946658
H	0.670698	-3.398435	2.379722
C	-0.013646	-3.307030	-0.290806
H	0.867676	-3.926314	-0.099856
H	0.032637	-2.901912	-1.304661
H	-0.905509	-3.931989	-0.197043
C	4.604660	-1.601399	1.212678
C	5.807099	-1.142927	1.764326
C	4.605678	-2.825703	0.528024
C	6.983049	-1.883161	1.626440
H	5.831064	-0.209287	2.314803
C	5.777810	-3.567660	0.397053
H	3.683793	-3.203533	0.101618
C	6.973671	-3.098402	0.943828
H	7.904482	-1.508817	2.063323
H	5.755497	-4.514527	-0.134754
H	7.887126	-3.676975	0.840943
C	3.276055	0.094631	2.571290
C	3.777348	1.403684	2.560019
C	2.785413	-0.419194	3.780140
C	3.778272	2.181759	3.720009
H	4.182024	1.830051	1.647077
C	2.778448	0.359768	4.937083
H	2.405611	-1.433815	3.809761
C	3.273954	1.664503	4.912637
H	4.171330	3.193740	3.685523
H	2.387421	-0.057010	5.861005
H	3.268634	2.270640	5.813772
C	-4.311608	-2.104131	-0.598631
C	-3.764415	-2.346138	-1.869560
C	-5.444899	-2.832972	-0.208576
C	-4.354607	-3.270094	-2.732362
H	-2.865813	-1.835434	-2.206688
C	-6.033889	-3.755344	-1.074509
H	-5.875140	-2.681243	0.774462
C	-5.494439	-3.974073	-2.342124
H	-3.913996	-3.434422	-3.711341
H	-6.915418	-4.303003	-0.753154
H	-5.955127	-4.689629	-3.017409
C	-4.481331	-0.737754	1.555315
C	-4.124984	-1.026389	2.876317
C	-5.679192	-0.045556	1.317095
C	-4.941123	-0.623426	3.937365
H	-3.210912	-1.572024	3.076597
C	-6.490485	0.357962	2.374586
H	-5.978474	0.177861	0.298128
C	-6.123667	0.071335	3.692121
H	-4.647925	-0.859063	4.956588
H	-7.410990	0.896524	2.168914
H	-6.757045	0.385043	4.516819
C	0.969336	2.021734	-2.409011
H	0.810317	1.656461	-3.416064
H	2.006822	2.090734	-2.085329
C	-0.004982	2.798375	-1.758659
H	-0.201933	1.793609	-0.325436
H	-0.964858	2.924266	-2.260459
C	0.346458	3.942046	-0.817731
H	1.257838	3.692730	-0.259020
H	-0.451238	4.088888	-0.079855
C	0.565814	5.253230	-1.593756

H	1.369649	5.107977	-2.327989
H	-0.338465	5.487791	-2.173071
C	0.907332	6.441073	-0.685067
H	1.810156	6.204383	-0.105171
H	0.102262	6.577825	0.049932
C	1.123171	7.747316	-1.455996
H	1.945662	7.652632	-2.174070
H	1.364171	8.575729	-0.781542
H	0.225603	8.027180	-2.019221
C	3.705693	-0.189010	-1.245743
C	3.282196	-1.001057	-2.302938
C	4.932416	0.481734	-1.355959
C	4.079348	-1.150574	-3.440360
H	2.309093	-1.477170	-2.264050
C	5.731179	0.326818	-2.487466
H	5.272014	1.126773	-0.548705
C	5.306502	-0.494849	-3.534490
H	3.727183	-1.772976	-4.257757
H	6.680315	0.851253	-2.554000
H	5.923764	-0.612277	-4.420672
C	-3.189377	1.474964	0.122864
C	-3.980027	2.308592	-0.676289
C	-2.706062	1.968807	1.341628
C	-4.299536	3.603714	-0.261962
H	-4.345771	1.942067	-1.632532
C	-3.020615	3.261789	1.756944
H	-2.078980	1.340514	1.966292
C	-3.821780	4.082770	0.957894
H	-4.915185	4.236422	-0.894965
H	-2.641088	3.629422	2.706142
H	-4.066826	5.089677	1.283586
H	-3.112998	0.029629	-1.421976
H	2.868787	1.073715	0.242229
Br	-0.512099	-0.787962	-3.277934

^{3}TS4a

B3LYP/BSI SCF energy: -2418.48256a.u.

M06/BSII SCF energy in solution: -2417.359458a.u.

M06/BSII free energy in solution: -2416.56805a.u.

C	-1.565868	-1.139274	0.555993
N	-1.567989	-0.145807	-0.244801
O	-2.748653	-1.541541	1.058499
C	-3.817314	-0.734147	0.425733
C	-2.957404	0.307697	-0.437328
C	0.945190	-1.311698	0.768260
N	1.296492	-0.441536	-0.107829
O	1.908855	-1.742453	1.610191
C	3.057469	-0.836711	1.461299
C	2.754405	-0.226349	0.028335
Ni	0.048417	0.529554	-1.465643
C	-0.391303	-2.018583	0.945747
C	-0.554835	-2.494593	2.408128
H	-1.510869	-3.007094	2.517908
H	-0.526134	-1.655031	3.108760
H	0.248505	-3.184915	2.667953
C	-0.408747	-3.250452	-0.012253
H	0.424185	-3.917294	0.228509
H	-0.338522	-2.933311	-1.055990
H	-1.344604	-3.797898	0.125549
C	4.325191	-1.677801	1.588510
C	5.516648	-1.116586	2.065254
C	4.323800	-3.025511	1.203370
C	6.681313	-1.881316	2.145247

H	5.538568	-0.081310	2.387448
C	5.485466	-3.790995	1.292635
H	3.409794	-3.477662	0.837612
C	6.670466	-3.221886	1.761466
H	7.594820	-1.426951	2.517958
H	5.462498	-4.834828	0.993250
H	7.575127	-3.818931	1.830045
C	2.944045	0.207922	2.582375
C	3.415740	1.518626	2.436353
C	2.412583	-0.175193	3.823035
C	3.341862	2.428135	3.494639
H	3.853860	1.849375	1.500113
C	2.332820	0.733359	4.877135
H	2.059710	-1.191220	3.958387
C	2.795608	2.040978	4.717000
H	3.709732	3.440423	3.355587
H	1.912160	0.416613	5.827259
H	2.733431	2.749243	5.537878
C	-4.618615	-1.722944	-0.433802
C	-4.126830	-2.151203	-1.677848
C	-5.801273	-2.301678	0.051417
C	-4.816431	-3.109055	-2.422507
H	-3.198743	-1.761230	-2.087682
C	-6.489065	-3.258408	-0.695723
H	-6.192361	-2.003761	1.017290
C	-6.002265	-3.662690	-1.938929
H	-4.417060	-3.417389	-3.384294
H	-7.406377	-3.686825	-0.301591
H	-6.539596	-4.404510	-2.523092
C	-4.645991	-0.095895	1.533980
C	-4.326627	-0.258936	2.885484
C	-5.764061	0.682344	1.195372
C	-5.100967	0.349786	3.877485
H	-3.475346	-0.868350	3.164013
C	-6.533750	1.290467	2.183824
H	-6.033307	0.812376	0.152141
C	-6.204343	1.127382	3.531988
H	-4.838127	0.209356	4.922281
H	-7.392129	1.892489	1.900260
H	-6.805317	1.600532	4.303153
C	1.566933	1.567213	-2.858605
H	1.161156	1.208332	-3.795125
H	2.568751	1.245352	-2.594413
C	0.925330	2.566819	-2.120895
H	0.003750	2.010192	-0.847448
H	0.047778	3.020550	-2.583202
C	1.704731	3.526654	-1.233570
H	2.466914	2.969214	-0.673708
H	1.038701	3.980081	-0.490554
C	2.390102	4.632333	-2.056555
H	3.065504	4.173747	-2.789325
H	1.630124	5.172942	-2.638505
C	3.166660	5.630923	-1.188660
H	3.925753	5.089527	-0.606291
H	2.484991	6.080379	-0.453543
C	3.843718	6.738710	-2.002273
H	4.553855	6.321439	-2.725143
H	4.393655	7.432055	-1.357064
H	3.106474	7.322297	-2.565234
C	3.545480	-0.813852	-1.131739
C	3.031564	-1.814229	-1.962954
C	4.837214	-0.326925	-1.377597
C	3.799235	-2.325037	-3.011788
H	2.015413	-2.165614	-1.827631

C	5.606577	-0.841043	-2.419657
H	5.247157	0.459054	-0.747911
C	5.088716	-1.845560	-3.240408
H	3.377038	-3.088860	-3.657951
H	6.605558	-0.452239	-2.595152
H	5.682439	-2.241532	-4.059199
C	-3.123733	1.785555	-0.137260
C	-3.815837	2.591027	-1.048672
C	-2.594742	2.374310	1.018417
C	-3.993176	3.955031	-0.806019
H	-4.213476	2.148432	-1.958887
C	-2.769243	3.734832	1.263085
H	-2.038832	1.767628	1.725980
C	-3.471594	4.529622	0.352841
H	-4.531577	4.565610	-1.525294
H	-2.353553	4.177301	2.163976
H	-3.605039	5.590591	0.544428
H	-3.194392	0.164923	-1.493431
H	2.934090	0.847923	0.047962
Br	-0.837201	-0.945498	-3.354210

^{1}[Ni^{II}]H$_2$

B3LYP/BSI SCF energy: -2169.788588a.u.

M06/BSII SCF energy in solution: -2168.838011a.u.

M06/BSII free energy in solution: -2168.190296a.u.

C	-1.256777	0.109592	-0.665313
N	-1.388406	0.023037	0.608388
O	-2.385992	0.307671	-1.386713
C	-3.441119	0.674636	-0.413004
C	-2.839473	0.069690	0.913455
C	1.256726	-0.109313	-0.665333
N	1.388418	-0.022673	0.608355
O	2.385856	-0.307656	-1.386777
C	3.441027	-0.674647	-0.413128
C	2.839480	-0.069725	0.913408
Ni	0.000017	-0.000034	1.992912
C	-0.000009	0.000376	-1.503193
C	0.120761	1.265798	-2.397841
H	-0.766439	1.358764	-3.026490
H	0.205874	2.169160	-1.787080
H	1.004281	1.185663	-3.034684
C	-0.120729	-1.264429	-2.398694
H	0.766463	-1.356928	-3.027420
H	-0.205775	-2.168221	-1.788557
H	-1.004267	-1.183912	-3.035458
C	3.480371	-2.212292	-0.413343
C	4.210424	-2.876395	-1.412130
C	2.734707	-2.983865	0.491598
C	4.209139	-4.267343	-1.497042
H	4.788111	-2.297973	-2.125312
C	2.736070	-4.378536	0.406611
H	2.136618	-2.515976	1.266091
C	3.473701	-5.025481	-0.583786
H	4.786612	-4.758293	-2.275271
H	2.156540	-4.954662	1.122094
H	3.476733	-6.110080	-0.644120
C	4.759083	-0.066095	-0.859091
C	5.913560	-0.330077	-0.107298
C	4.861741	0.744123	-1.993647
C	7.140753	0.215038	-0.477031
H	5.852192	-0.970603	0.767747
C	6.094971	1.285038	-2.367519
H	3.977898	0.948066	-2.586236

C	7.236547	1.025600	-1.611115
H	8.023710	0.004232	0.119338
H	6.158394	1.910392	-3.253590
H	8.194092	1.447878	-1.901647
C	-4.759210	0.066095	-0.858855
C	-5.913674	0.330385	-0.107151
C	-4.861898	-0.744465	-1.993162
C	-7.140902	-0.214749	-0.476740
H	-5.852265	0.971190	0.767687
C	-6.095166	-1.285390	-2.366896
H	-3.978055	-0.948655	-2.585666
C	-7.236735	-1.025634	-1.610591
H	-8.023857	-0.003697	0.119544
H	-6.158625	-1.911002	-3.252782
H	-8.194309	-1.447922	-1.901016
C	-3.480493	2.212280	-0.413244
C	-4.210023	2.876249	-1.412508
C	-2.735465	2.983973	0.492103
C	-4.208837	4.267185	-1.497504
H	-4.787226	2.297708	-2.125990
C	-2.736936	4.378645	0.407041
H	-2.137780	2.516197	1.266970
C	-3.474034	5.025453	-0.583836
H	-4.785891	4.758034	-2.276108
H	-2.157894	4.954876	1.122834
H	-3.477142	6.110048	-0.644233
C	3.348771	1.293698	1.348727
C	4.080753	1.402816	2.535752
C	3.100651	2.452672	0.602261
C	4.565338	2.638757	2.966024
H	4.257780	0.515485	3.137488
C	3.582236	3.689100	1.029199
H	2.524686	2.393375	-0.315945
C	4.318174	3.786024	2.212392
H	5.125551	2.704626	3.894265
H	3.380734	4.578664	0.438931
H	4.689603	4.750536	2.546674
H	2.993051	-0.757528	1.742785
H	-2.993241	0.757304	1.742948
C	-3.348510	-1.293918	1.348511
C	-4.080972	-1.403296	2.535215
C	-3.099764	-2.452791	0.602095
C	-4.565406	-2.639387	2.965223
H	-4.258512	-0.516041	3.136913
C	-3.581196	-3.689371	1.028771
H	-2.523415	-2.393307	-0.315856
C	-4.317609	-3.786552	2.211644
H	-5.125998	-2.705449	3.893221
H	-3.379198	-4.578854	0.438551
H	-4.688909	-4.751184	2.545727
H	0.928663	0.011015	3.118253
H	-0.928518	-0.011460	3.118365

^{3}[Ni^{II}]H$_2$

B3LYP/BSI SCF energy: -2169.763357a.u.

M06/BSII SCF energy in solution: -2168.802976a.u.

M06/BSII free energy in solution: -2168.159336a.u.

C	1.258629	0.108101	-0.701238
N	1.452289	0.345597	0.542946
O	2.307413	-0.360729	-1.407841
C	3.355561	-0.734295	-0.424212
C	2.870943	0.067428	0.852441
C	-1.258849	0.289317	-0.673249

N	-1.373911	0.119818	0.590833
O	-2.401530	0.443016	-1.378140
C	-3.479457	0.665952	-0.379095
C	-2.815720	0.044649	0.911157
Ni	0.026204	0.500776	2.087940
C	0.001098	0.357933	-1.517651
C	-0.113433	-0.693910	-2.650810
H	0.754441	-0.631038	-3.308493
H	-0.157236	-1.708436	-2.244712
H	-1.016868	-0.506981	-3.233521
C	0.105501	1.783554	-2.132867
H	-0.777532	1.988333	-2.741578
H	0.173501	2.543305	-1.349902
H	0.994440	1.847295	-2.766359
C	-3.650754	2.191394	-0.304670
C	-4.395683	2.835820	-1.306171
C	-3.016646	2.977601	0.669406
C	-4.516204	4.223510	-1.328606
H	-4.888586	2.243241	-2.069932
C	-3.142667	4.369654	0.646330
H	-2.411815	2.531616	1.453750
C	-3.891809	4.997175	-0.347532
H	-5.102074	4.699588	-2.109842
H	-2.648757	4.957954	1.414258
H	-3.989982	6.079028	-0.358940
C	-4.741769	-0.033411	-0.852680
C	-5.919018	0.121640	-0.106056
C	-4.770079	-0.827878	-2.002619
C	-7.095322	-0.513895	-0.496634
H	-5.915506	0.749134	0.780483
C	-5.952811	-1.458746	-2.397644
H	-3.868643	-0.949620	-2.591286

C	-7.117168	-1.306938	-1.646763
H	-7.996774	-0.386099	0.095603
H	-5.958977	-2.069595	-3.295994
H	-8.035461	-1.798793	-1.954055
C	4.709048	-0.311639	-0.969159
C	5.858905	-0.580402	-0.211684
C	4.847676	0.332679	-2.202176
C	7.116802	-0.199148	-0.672339
H	5.768844	-1.095264	0.740317
C	6.110989	0.710431	-2.665625
H	3.967862	0.534768	-2.801450
C	7.247930	0.449492	-1.903080
H	7.995786	-0.410668	-0.070462
H	6.201525	1.208225	-3.626993
H	8.229061	0.744657	-2.263418
C	3.228940	-2.256654	-0.255627
C	3.937743	-3.112574	-1.112930
C	2.339091	-2.821709	0.673404
C	3.778404	-4.495469	-1.035170
H	4.622038	-2.694703	-1.843246
C	2.183736	-4.207668	0.749030
H	1.748424	-2.202505	1.344581
C	2.902652	-5.049045	-0.099732
H	4.342056	-5.139082	-1.704710
H	1.495166	-4.622845	1.479189
H	2.782069	-6.126738	-0.033855
C	-3.216903	-1.371175	1.290374
C	-3.963905	-1.578163	2.455214
C	-2.854999	-2.481066	0.516351
C	-4.354331	-2.863236	2.834828
H	-4.230266	-0.727777	3.077278
C	-3.242374	-3.765896	0.893044

H	-2.261945	-2.346062	-0.382891
C	-3.995840	-3.961000	2.052804
H	-4.928606	-3.005053	3.745803
H	-2.952669	-4.616375	0.282295
H	-4.293885	-4.963056	2.347829
H	-3.002729	0.680591	1.775491
H	2.902606	-0.586734	1.724251
C	3.601215	1.349615	1.212065
C	4.305990	1.420898	2.419105
C	3.567596	2.479250	0.383380
C	4.974585	2.589844	2.788150
H	4.322930	0.558101	3.080124
C	4.234383	3.647102	0.748551
H	3.018211	2.447324	-0.552474
C	4.941537	3.705578	1.952283
H	5.512005	2.628648	3.731197
H	4.200222	4.514051	0.094863
H	5.457218	4.617870	2.237869
H	-0.869046	1.354573	3.077838
H	0.450249	-0.846980	2.798771

H_2

B3LYP/BSI SCF energy: -1.178539a.u.

M06/BSII SCF energy in solution: -1.169882a.u.

M06/BSII free energy in solution: -1.171187a.u.

H	0.000000	0.000000	0.371265
H	0.000000	0.000000	-0.371265

^{1}TS5

B3LYP/BSI SCF energy: -2405.633269a.u.

M06/BSII SCF energy in solution: -2404.544146a.u.

M06/BSII free energy in solution: -2403.740883a.u.

C	-1.108744	-0.974610	0.871887
N	-1.456128	-0.043713	0.062713
O	-1.961776	-2.011054	1.005621
C	-3.095408	-1.817609	0.101267
C	-2.737709	-0.416818	-0.575213
C	1.352335	-0.756759	0.883129
N	1.556678	-1.284257	-0.253165
O	2.321059	0.034465	1.421772
C	3.456605	0.024678	0.498723
C	2.837155	-0.770940	-0.753291
Ni	-0.599371	1.550456	-0.589125
C	0.132775	-1.026230	1.753384
C	-0.024559	0.014471	2.885566
H	-0.892410	-0.244131	3.500316
H	-0.162670	1.016984	2.475604
H	0.862292	0.010483	3.523148
C	0.314170	-2.439905	2.360819
H	1.228557	-2.458915	2.960561
H	0.395980	-3.194164	1.576213
H	-0.527240	-2.693627	3.009820
C	4.630695	-0.667840	1.205011
C	5.906959	-0.667820	0.622876
C	4.462237	-1.304791	2.439900
C	6.977696	-1.300904	1.249879
H	6.068437	-0.170331	-0.327005
C	5.537080	-1.935896	3.071433
H	3.490581	-1.292096	2.918077
C	6.798272	-1.939694	2.478942
H	7.955853	-1.291280	0.777568
H	5.383478	-2.420635	4.031728

H	7.634434	-2.429707	2.969443
C	3.810972	1.479899	0.190285
C	4.371188	1.858726	-1.035898
C	3.634768	2.459706	1.178101
C	4.741014	3.184995	-1.273483
H	4.527384	1.121371	-1.816398
C	3.997382	3.784701	0.938578
H	3.203127	2.179904	2.132292
C	4.553012	4.153738	-0.288682
H	5.171791	3.457283	-2.232810
H	3.846852	4.530039	1.714568
H	4.835768	5.185764	-0.474380
C	-3.083644	-2.982619	-0.894309
C	-3.581329	-2.845729	-2.195976
C	-2.620490	-4.239854	-0.482115
C	-3.611586	-3.936811	-3.067329
H	-3.950397	-1.886709	-2.545561
C	-2.641662	-5.326311	-1.354954
H	-2.233066	-4.361681	0.523001
C	-3.138876	-5.180410	-2.651419
H	-3.998684	-3.807300	-4.073871
H	-2.267767	-6.289721	-1.020199
H	-3.154393	-6.027329	-3.331102
C	-4.373645	-1.840728	0.948309
C	-4.320428	-1.768744	2.345222
C	-5.628648	-1.928633	0.329684
C	-5.492746	-1.775866	3.104576
H	-3.359888	-1.717877	2.843693
C	-6.798098	-1.928306	1.087119
H	-5.696856	-2.003186	-0.749954
C	-6.736298	-1.852136	2.479920
H	-5.428082	-1.724081	4.187885
H	-7.759701	-1.993497	0.586214
H	-7.647998	-1.857382	3.070213
C	0.189196	2.937993	-1.786374
H	-0.522905	3.203233	-2.563525
H	1.152082	2.587717	-2.144965
C	0.066421	3.490391	-0.501114
H	-0.557707	2.181384	0.770196
H	0.967668	3.545825	0.104992
C	-0.979557	4.546333	-0.186360
H	-1.207908	4.540381	0.885833
H	-1.916150	4.305312	-0.703882
C	-0.514302	5.954965	-0.600231
H	0.437522	6.184563	-0.099546
H	-0.300465	5.964031	-1.677557
C	-1.536493	7.050913	-0.272634
H	-1.750655	7.035904	0.804923
H	-2.486593	6.818859	-0.773158
C	-1.072363	8.452640	-0.681759
H	-0.142680	8.726906	-0.169812
H	-1.822579	9.212247	-0.437281
H	-0.882371	8.507712	-1.759874
C	3.675064	-1.861895	-1.393387
C	3.844397	-3.107795	-0.774809
C	4.265716	-1.651077	-2.644579
C	4.604018	-4.105793	-1.381774
H	3.372012	-3.295053	0.183800
C	5.031259	-2.647214	-3.255132
H	4.108922	-0.706215	-3.159665
C	5.205540	-3.877519	-2.622322
H	4.725151	-5.065697	-0.887452
H	5.478878	-2.464019	-4.227971
H	5.796565	-4.656919	-3.094959

C	-3.769905	0.691652	-0.490216
C	-4.505986	1.036380	-1.629548
C	-4.008698	1.387200	0.702451
C	-5.475401	2.040151	-1.578973
H	-4.308011	0.528545	-2.570340
C	-4.972761	2.392276	0.755356
H	-3.429893	1.147887	1.588134
C	-5.712813	2.719171	-0.383921
H	-6.035256	2.295737	-2.474149
H	-5.145123	2.923562	1.687036
H	-6.463707	3.502980	-0.340737
H	-2.509743	-0.581403	-1.629464
H	2.598833	-0.033657	-1.527329
H	-0.599639	0.945387	-1.962469

^{3}TS5

B3LYP/BSI SCF energy: -2405.597883a.u.

M06/BSII SCF energy in solution: -2404.502099a.u.

M06/BSII free energy in solution: -2403.70517a.u.

C	-0.998236	-0.488679	0.831854
N	-1.510599	0.133303	-0.167066
O	-1.567621	-1.659507	1.180474
C	-2.522846	-2.030101	0.128427
C	-2.662870	-0.655939	-0.655026
C	1.417933	0.006478	0.859985
N	1.646949	-0.754632	-0.129788
O	2.390470	0.866043	1.267160
C	3.522449	0.714474	0.350323
C	2.979601	-0.426370	-0.647021
Ni	-0.597876	1.390661	-1.638210
C	0.158137	-0.022796	1.712798
C	-0.168282	1.374640	2.284369
H	-1.080858	1.324405	2.886419
H	-0.310013	2.098447	1.481693
H	0.649900	1.713057	2.923788
C	0.402430	-1.015032	2.876418
H	1.245195	-0.663637	3.477582
H	0.627305	-2.015510	2.503318
H	-0.476611	-1.074061	3.523593
C	4.753134	0.335582	1.182385
C	6.029315	0.339861	0.600789
C	4.636996	-0.022058	2.530635
C	7.153134	-0.017680	1.342478
H	6.150743	0.629361	-0.437240
C	5.764582	-0.374118	3.276318
H	3.662551	-0.010952	3.003322
C	7.026980	-0.376707	2.686074
H	8.130904	-0.009983	0.869264
H	5.650712	-0.643501	4.322842
H	7.904094	-0.650406	3.265374
C	3.738374	2.065171	-0.339390
C	4.257901	2.159336	-1.636329
C	3.472476	3.249980	0.361705
C	4.498380	3.404306	-2.222760
H	4.480049	1.262386	-2.205493
C	3.704555	4.492615	-0.225608
H	3.073204	3.193127	1.367879
C	4.219630	4.576130	-1.521204
H	4.898191	3.452457	-3.231598
H	3.484101	5.398502	0.332255
H	4.401027	5.544680	-1.978048
C	-1.868161	-3.145197	-0.700505
C	-2.078812	-3.283406	-2.076816

C	-1.087954	-4.107195	-0.042338
C	-1.515381	-4.350504	-2.781129
H	-2.676135	-2.561363	-2.623326
C	-0.519367	-5.166295	-0.745568
H	-0.921205	-4.017248	1.025393
C	-0.731154	-5.293305	-2.120152
H	-1.685527	-4.432744	-3.850627
H	0.091473	-5.893684	-0.218593
H	-0.285840	-6.117521	-2.669576
C	-3.793218	-2.544794	0.805688
C	-3.998977	-2.402100	2.182509
C	-4.784765	-3.172986	0.039225
C	-5.171076	-2.872913	2.778497
H	-3.236938	-1.930972	2.791649
C	-5.958019	-3.633904	0.633048
H	-4.635182	-3.315206	-1.026053
C	-6.156533	-3.487147	2.007402
H	-5.309170	-2.758578	3.850081
H	-6.714765	-4.115792	0.020660
H	-7.067966	-3.852803	2.471367
C	-2.059918	2.817770	-2.293886
H	-3.033768	2.423796	-2.021373
H	-1.862973	2.934495	-3.353784
C	-1.291494	3.516502	-1.341545
H	-0.074724	2.585290	-0.686586
H	-0.529754	4.190128	-1.733228
C	-1.902356	3.973401	-0.028168
H	-1.111973	4.185507	0.702415
H	-2.515953	3.165038	0.385521
C	-2.772954	5.229921	-0.210643
H	-2.166499	6.034375	-0.651217
H	-3.568827	5.014866	-0.934641
C	-3.392607	5.723412	1.103149
H	-2.593110	5.928864	1.828428
H	-3.998574	4.917107	1.538815
C	-4.257774	6.975738	0.929525
H	-3.671186	7.809929	0.527830
H	-4.688560	7.301319	1.882339
H	-5.085713	6.791588	0.235349
C	3.837690	-1.664672	-0.831810
C	3.900396	-2.663171	0.148506
C	4.568477	-1.838622	-2.012318
C	4.688149	-3.797072	-0.040790
H	3.321512	-2.552721	1.059578
C	5.361300	-2.971823	-2.205506
H	4.504890	-1.089811	-2.798395
C	5.425216	-3.953921	-1.217399
H	4.725360	-4.562032	0.729918
H	5.918606	-3.089739	-3.130618
H	6.037956	-4.838821	-1.364642
C	-3.970231	0.098249	-0.483176
C	-4.896772	0.118459	-1.532167
C	-4.282444	0.779096	0.700831
C	-6.111499	0.794639	-1.402278
H	-4.661397	-0.390312	-2.463625
C	-5.493954	1.457273	0.833947
H	-3.571852	0.785545	1.521449
C	-6.413555	1.466092	-0.217339
H	-6.815751	0.802293	-2.229280
H	-5.720485	1.979251	1.759476
H	-7.355966	1.996179	-0.114149
H	-2.500208	-0.817696	-1.719933
H	2.832453	0.025531	-1.631744
H	-0.595612	0.332371	-2.783131

^{3}TS5

B3LYP/BSI SCF energy: -2405.597883a.u.

M06/BSII SCF energy in solution: -2404.502099a.u.

M06/BSII free energy in solution: -2403.70517a.u.

C	-0.998236	-0.488679	0.831854
N	-1.510599	0.133303	-0.167066
O	-1.567621	-1.659507	1.180474
C	-2.522846	-2.030101	0.128427
C	-2.662870	-0.655939	-0.655026
C	1.417933	0.006478	0.859985
N	1.646949	-0.754632	-0.129788
O	2.390470	0.866043	1.267160
C	3.522449	0.714474	0.350323
C	2.979601	-0.426370	-0.647021
Ni	-0.597876	1.390661	-1.638210
C	0.158137	-0.022796	1.712798
C	-0.168282	1.374640	2.284369
H	-1.080858	1.324405	2.886419
H	-0.310013	2.098447	1.481693
H	0.649900	1.713057	2.923788
C	0.402430	-1.015032	2.876418
H	1.245195	-0.663637	3.477582
H	0.627305	-2.015510	2.503318
H	-0.476611	-1.074061	3.523593
C	4.753134	0.335582	1.182385
C	6.029315	0.339861	0.600789
C	4.636996	-0.022058	2.530635
C	7.153134	-0.017680	1.342478
H	6.150743	0.629361	-0.437240
C	5.764582	-0.374118	3.276318
H	3.662551	-0.010952	3.003322
C	7.026980	-0.376707	2.686074
H	8.130904	-0.009983	0.869264
H	5.650712	-0.643501	4.322842
H	7.904094	-0.650406	3.265374
C	3.738374	2.065171	-0.339390
C	4.257901	2.159336	-1.636329
C	3.472476	3.249980	0.361705
C	4.498380	3.404306	-2.222760
H	4.480049	1.262386	-2.205493
C	3.704555	4.492615	-0.225608
H	3.073204	3.193127	1.367879
C	4.219630	4.576130	-1.521204
H	4.898191	3.452457	-3.231598
H	3.484101	5.398502	0.332255
H	4.401027	5.544680	-1.978048
C	-1.868161	-3.145197	-0.700505
C	-2.078812	-3.283406	-2.076816
C	-1.087954	-4.107195	-0.042338
C	-1.515381	-4.350504	-2.781129
H	-2.676135	-2.561363	-2.623326
C	-0.519367	-5.166295	-0.745568
H	-0.921205	-4.017248	1.025393
C	-0.731154	-5.293305	-2.120152
H	-1.685527	-4.432744	-3.850627
H	0.091473	-5.893684	-0.218593
H	-0.285840	-6.117521	-2.669576
C	-3.793218	-2.544794	0.805688
C	-3.998977	-2.402100	2.182509
C	-4.784765	-3.172986	0.039225
C	-5.171076	-2.872913	2.778497
H	-3.236938	-1.930972	2.791649

C	-5.958019	-3.633904	0.633048
H	-4.635182	-3.315206	-1.026053
C	-6.156533	-3.487147	2.007402
H	-5.309170	-2.758578	3.850081
H	-6.714765	-4.115792	0.020660
H	-7.067966	-3.852803	2.471367
C	-2.059918	2.817770	-2.293886
H	-3.033768	2.423796	-2.021373
H	-1.862973	2.934495	-3.353784
C	-1.291494	3.516502	-1.341545
H	-0.074724	2.585290	-0.686586
H	-0.529754	4.190128	-1.733228
C	-1.902356	3.973401	-0.028168
H	-1.111973	4.185507	0.702415
H	-2.515953	3.165038	0.385521
C	-2.772954	5.229921	-0.210643
H	-2.166499	6.034375	-0.651217
H	-3.568827	5.014866	-0.934641
C	-3.392607	5.723412	1.103149
H	-2.593110	5.928864	1.828428
H	-3.998574	4.917107	1.538815
C	-4.257774	6.975738	0.929525
H	-3.671186	7.809929	0.527830
H	-4.688560	7.301319	1.882339
H	-5.085713	6.791588	0.235349
C	3.837690	-1.664672	-0.831810
C	3.900396	-2.663171	0.148506
C	4.568477	-1.838622	-2.012318
C	4.688149	-3.797072	-0.040790
H	3.321512	-2.552721	1.059578
C	5.361300	-2.971823	-2.205506
H	4.504890	-1.089811	-2.798395
C	5.425216	-3.953921	-1.217399
H	4.725360	-4.562032	0.729918
H	5.918606	-3.089739	-3.130618
H	6.037956	-4.838821	-1.364642
C	-3.970231	0.098249	-0.483176
C	-4.896772	0.118459	-1.532167
C	-4.282444	0.779096	0.700831
C	-6.111499	0.794639	-1.402278
H	-4.661397	-0.390312	-2.463625
C	-5.493954	1.457273	0.833947
H	-3.571852	0.785545	1.521449
C	-6.413555	1.466092	-0.217339
H	-6.815751	0.802293	-2.229280
H	-5.720485	1.979251	1.759476
H	-7.355966	1.996179	-0.114149
H	-2.500208	-0.817696	-1.719933
H	2.832453	0.025531	-1.631744
H	-0.595612	0.332371	-2.783131

[1]IM1

B3LYP/BSI SCF energy: -2405.677161a.u.

M06/BSII SCF energy in solution: -2404.584368a.u.

M06/BSII free energy in solution: -2403.77716a.u.

C	0.593446	-1.560887	-0.479594
N	1.090619	-0.390390	-0.650738
O	1.379874	-2.480352	0.129089
C	2.489625	-1.724568	0.737797
C	2.498123	-0.445503	-0.191011
C	-1.807953	-0.969643	-0.618801
N	-1.598462	0.291834	-0.525286
O	-3.082748	-1.398509	-0.495782

C	-3.932467	-0.182113	-0.563642
C	-2.882779	0.929813	-0.166242
Ni	0.030410	1.303146	-1.023219
C	-0.777769	-2.039204	-0.929270
C	-0.757699	-2.240497	-2.473035
H	-0.046446	-3.029705	-2.735526
H	-0.471446	-1.315319	-2.978212
H	-1.751055	-2.540209	-2.817467
C	-1.159295	-3.363987	-0.234916
H	-2.144534	-3.685566	-0.575504
H	-1.186154	-3.255121	0.852308
H	-0.428982	-4.137125	-0.480430
C	-5.094089	-0.338968	0.401493
C	-6.150578	0.582148	0.350834
C	-5.128754	-1.355604	1.361123
C	-7.214459	0.491812	1.246087
H	-6.144090	1.367003	-0.399760
C	-6.199880	-1.449749	2.252740
H	-4.320255	-2.075547	1.405107
C	-7.243843	-0.527045	2.201091
H	-8.023147	1.215075	1.193982
H	-6.214699	-2.248735	2.988652
H	-8.075408	-0.601217	2.895834
C	-4.385202	-0.092787	-2.028925
C	-3.726871	0.704174	-2.977776
C	-5.437342	-0.916575	-2.462411
C	-4.127925	0.690662	-4.316791
H	-2.889337	1.334736	-2.697807
C	-5.833441	-0.930994	-3.797899
H	-5.951432	-1.547308	-1.744525
C	-5.181663	-0.121765	-4.731464
H	-3.607783	1.320481	-5.032666
H	-6.653660	-1.572104	-4.108344
H	-5.492993	-0.126529	-5.772178
C	2.061419	-1.419654	2.183797
C	2.367022	-0.219078	2.835101
C	1.380578	-2.415563	2.902434
C	1.990143	-0.012079	4.165462
H	2.898287	0.574348	2.320885
C	1.000154	-2.207852	4.226476
H	1.150263	-3.356813	2.415229
C	1.303337	-1.002309	4.864833
H	2.232013	0.930696	4.646935
H	0.471993	-2.991280	4.762714
H	1.010760	-0.840393	5.898230
C	3.739676	-2.594682	0.700660
C	3.844661	-3.676825	-0.181797
C	4.821339	-2.303972	1.543018
C	5.005987	-4.449387	-0.220376
H	3.013578	-3.918190	-0.833234
C	5.985407	-3.070219	1.495613
H	4.751842	-1.483187	2.249545
C	6.082021	-4.148106	0.614507
H	5.067199	-5.288897	-0.907087
H	6.813337	-2.828117	2.155723
H	6.985564	-4.749973	0.582517
C	1.485936	2.432998	-1.586702
H	1.180879	3.029683	-2.456808
H	2.311916	1.791135	-1.927872
C	2.001662	3.385403	-0.496228
H	1.188599	4.048555	-0.171938
H	2.292831	2.822223	0.406914
C	3.204723	4.242296	-0.931381
H	2.922229	4.823858	-1.820939

H	4.022531	3.580184	-1.252893
C	3.721250	5.196603	0.153858
H	2.903055	5.857775	0.474810
H	4.004410	4.616266	1.045317
C	4.916045	6.051899	-0.287177
H	4.633519	6.635608	-1.174023
H	5.734094	5.392538	-0.609259
C	5.424420	6.999027	0.805119
H	4.637638	7.692423	1.124363
H	6.274011	7.597653	0.458578
H	5.750147	6.442716	1.692111
C	-2.880231	1.393771	1.281688
C	-2.278116	0.649662	2.303354
C	-3.491928	2.610095	1.608025
C	-2.296344	1.106377	3.621201
H	-1.770924	-0.281076	2.071250
C	-3.516496	3.067605	2.925529
H	-3.942779	3.210063	0.821813
C	-2.918884	2.315174	3.937511
H	-1.811356	0.520563	4.396782
H	-3.991778	4.016316	3.158043
H	-2.929141	2.673171	4.963120
C	3.488750	-0.486218	-1.345102
C	4.748476	0.102759	-1.175707
C	3.198822	-1.112434	-2.562123
C	5.700266	0.060445	-2.193411
H	4.983913	0.606798	-0.241950
C	4.148066	-1.152587	-3.584476
H	2.219850	-1.549579	-2.725201
C	5.402458	-0.569309	-3.403158
H	6.669612	0.527306	-2.044518
H	3.902984	-1.634903	-4.526611
H	6.139099	-0.597051	-4.200844
H	2.701005	0.451017	0.391229
H	-3.019114	1.810986	-0.789967
H	-0.753044	2.522101	-1.210207

^{3}IM1

B3LYP/BSI SCF energy: -2405.658046a.u.

M06/BSII SCF energy in solution: -2404.553367a.u.

M06/BSII free energy in solution: -2403.751156a.u.

C	1.151177	-1.324762	0.703209
N	1.326574	-0.273525	-0.007767
O	2.261211	-1.947848	1.162055
C	3.392474	-1.019519	0.929492
C	2.783176	-0.090169	-0.194850
C	-1.371217	-1.336575	0.541360
N	-1.503828	-0.128715	0.138156
O	-2.465888	-2.126355	0.497360
C	-3.489671	-1.398680	-0.292047
C	-2.915299	0.078588	-0.245304
Ni	-0.037491	1.240323	-0.543009
C	-0.140165	-1.995930	1.137203
C	-0.084101	-3.486982	0.714050
H	0.792355	-3.962060	1.157572
H	-0.029037	-3.586720	-0.373695
H	-0.981609	-4.003920	1.055580
C	-0.248098	-1.896133	2.686883
H	-1.159304	-2.397179	3.024341
H	-0.278211	-0.852068	3.009748
H	0.614242	-2.379362	3.150548
C	-4.841194	-1.577760	0.378159
C	-5.975154	-1.003366	-0.215709

C	-4.993801	-2.296520	1.567503
C	-7.229493	-1.134706	0.375324
H	-5.876061	-0.456254	-1.148533
C	-6.253726	-2.432175	2.157087
H	-4.127576	-2.755033	2.029411
C	-7.374109	-1.850683	1.566437
H	-8.095403	-0.679292	-0.096345
H	-6.354609	-2.996664	3.079809
H	-8.352652	-1.955400	2.025883
C	-3.430837	-2.015461	-1.698406
C	-2.517071	-1.555787	-2.661758
C	-4.229480	-3.127408	-2.007288
C	-2.426933	-2.185127	-3.905315
H	-1.857556	-0.712820	-2.464843
C	-4.135328	-3.753179	-3.249795
H	-4.933050	-3.504561	-1.273105
C	-3.235271	-3.281093	-4.206458
H	-1.719046	-1.807906	-4.637775
H	-4.768437	-4.608393	-3.468991
H	-3.164887	-3.763971	-5.177143
C	3.606228	-0.304950	2.273949
C	3.039496	0.944041	2.567279
C	4.313350	-0.971401	3.288438
C	3.192826	1.515134	3.833746
H	2.467447	1.489834	1.825461
C	4.462006	-0.402970	4.551655
H	4.754066	-1.941309	3.083116
C	3.904377	0.847282	4.828605
H	2.752014	2.487303	4.034394
H	5.017661	-0.935568	5.318248
H	4.025210	1.295587	5.810626
C	4.611421	-1.821155	0.505895
C	4.553852	-3.197420	0.267279
C	5.835145	-1.155207	0.343612
C	5.697815	-3.894784	-0.129973
H	3.616144	-3.724533	0.396855
C	6.973033	-1.850146	-0.059523
H	5.898796	-0.089126	0.541425
C	6.908809	-3.225325	-0.297506
H	5.637200	-4.964800	-0.307593
H	7.911371	-1.317599	-0.184239
H	7.796731	-3.768362	-0.607797
C	1.024277	2.942971	-0.414007
H	1.899226	2.834064	-1.074877
H	1.426068	3.103010	0.604380
C	0.245264	4.200510	-0.831132
H	-0.179829	4.049939	-1.833974
H	-0.615841	4.350459	-0.161047
C	1.074322	5.498267	-0.843191
H	1.923284	5.371088	-1.531171
H	1.513707	5.654513	0.153634
C	0.277800	6.746635	-1.245428
H	-0.169483	6.587469	-2.237661
H	-0.566596	6.876816	-0.552342
C	1.110216	8.035244	-1.270197
H	1.948744	7.909766	-1.969043
H	1.562979	8.192247	-0.281236
C	0.300504	9.275841	-1.662387
H	-0.137219	9.162965	-2.661147
H	0.921522	10.178364	-1.671998
H	-0.524263	9.447317	-0.960749
C	-3.584977	1.076287	0.684248
C	-3.501972	0.963429	2.078702
C	-4.287031	2.156831	0.137451

C -4.117831 1.902120 2.904676
H -2.951602 0.139896 2.523154
C -4.905279 3.098584 0.962135
H -4.341756 2.267017 -0.942610
C -4.823484 2.972082 2.348600
H -4.045330 1.800309 3.983806
H -5.441292 3.933371 0.519948
H -5.299787 3.705149 2.993234
C 3.190461 -0.377191 -1.630716
C 4.024808 0.526897 -2.297199
C 2.751148 -1.517994 -2.313497
C 4.422406 0.294584 -3.614668
H 4.355555 1.427183 -1.786072
C 3.145856 -1.752894 -3.629428
H 2.088783 -2.223248 -1.821501
C 3.984629 -0.848000 -4.283934
H 5.064510 1.011272 -4.118451
H 2.794150 -2.641219 -4.146428
H 4.287291 -1.029371 -5.311260
H 3.029638 0.950630 0.011320
H -2.929087 0.502947 -1.250086
H -0.463362 1.045226 -2.064133

[1]TS6

B3LYP/BSI SCF energy: -2405.649612a.u.

M06/BSII SCF energy in solution: -2404.559907a.u.

M06/BSII free energy in solution: -2403.755555a.u.

C 0.555717 -1.398481 -0.829035
N 1.076618 -0.238472 -0.671743
O 1.303293 -2.474930 -0.457601
C 2.432664 -1.955104 0.318233
C 2.441031 -0.430315 -0.147560
C -1.832116 -0.721325 -0.871771
N -1.636576 0.504043 -0.539355
O -3.105196 -1.194302 -0.773767
C -3.972283 -0.034651 -0.541973
C -2.915340 1.030530 -0.015954
Ni -0.017548 1.526550 -0.761279
C -0.808279 -1.695506 -1.433776
C -0.730327 -1.467207 -2.972358
H -0.019622 -2.169870 -3.419611
H -0.412943 -0.444094 -3.187188
H -1.713012 -1.634168 -3.422950
C -1.232074 -3.150761 -1.143953
H -2.204578 -3.350667 -1.595851
H -1.305943 -3.341879 -0.070409
H -0.498419 -3.842088 -1.562614
C -5.057564 -0.448845 0.448188
C -6.253916 0.275789 0.537085
C -4.870001 -1.541799 1.304322
C -7.233131 -0.077053 1.465457
H -6.430875 1.112833 -0.130045
C -5.854357 -1.899773 2.225791
H -3.955227 -2.118351 1.239558
C -7.038904 -1.168030 2.313308
H -8.153001 0.498463 1.518098
H -5.692266 -2.754670 2.876357
H -7.804545 -1.447392 3.031339
C -4.572506 0.348617 -1.904967
C -4.873765 1.669681 -2.258018
C -4.894080 -0.672662 -2.812462
C -5.466674 1.964601 -3.488889
H -4.652625 2.488351 -1.581164

C	-5.480061	-0.379258	-4.042272
H	-4.679293	-1.702055	-2.547544
C	-5.768455	0.943073	-4.387282
H	-5.685346	2.997939	-3.742100
H	-5.715456	-1.185992	-4.730840
H	-6.224430	1.173149	-5.345786
C	2.060641	-2.110457	1.802323
C	2.521792	-1.232818	2.791948
C	1.275875	-3.205074	2.196606
C	2.196130	-1.433376	4.136095
H	3.142671	-0.381643	2.531417
C	0.945335	-3.402641	3.536221
H	0.923187	-3.900713	1.443600
C	1.402802	-2.515676	4.513341
H	2.562190	-0.736230	4.884180
H	0.331765	-4.253957	3.817371
H	1.146552	-2.669701	5.557495
C	3.669756	-2.772431	-0.043278
C	3.742010	-3.460996	-1.261386
C	4.770320	-2.825444	0.822638
C	4.885385	-4.183420	-1.603391
H	2.895744	-3.439683	-1.937402
C	5.916579	-3.540519	0.475817
H	4.731497	-2.317947	1.780558
C	5.979199	-4.224702	-0.738465
H	4.918288	-4.715719	-2.549887
H	6.757886	-3.568378	1.162477
H	6.868987	-4.787353	-1.005690
C	1.196695	3.156844	-1.073796
H	0.983590	4.073767	-1.641684
H	1.767351	2.523654	-1.768374
C	2.026510	3.531396	0.163092

H	1.399954	4.100890	0.863578
H	2.324108	2.624380	0.705702
C	3.283289	4.350175	-0.170601
H	2.985851	5.258795	-0.714413
H	3.915181	3.774314	-0.861304
C	4.108899	4.745386	1.060711
H	3.476051	5.314289	1.757400
H	4.410662	3.836504	1.602709
C	5.359284	5.569329	0.727774
H	5.058841	6.480201	0.191996
H	5.989596	5.002627	0.028685
C	6.181752	5.952022	1.962674
H	5.586877	6.546851	2.665517
H	7.064463	6.541757	1.692894
H	6.528919	5.061189	2.499341
C	-2.861146	1.249799	1.486777
C	-2.102490	0.430553	2.331552
C	-3.595264	2.299552	2.051679
C	-2.086061	0.651312	3.709035
H	-1.502433	-0.369323	1.911139
C	-3.583344	2.521625	3.428859
H	-4.177233	2.954333	1.407560
C	-2.828275	1.695801	4.263003
H	-1.483000	0.010077	4.345817
H	-4.156951	3.343679	3.847745
H	-2.812136	1.870246	5.335167
C	3.501929	-0.033143	-1.160753
C	4.722337	0.484950	-0.709290
C	3.300429	-0.171713	-2.539035
C	5.724902	0.844207	-1.610264
H	4.890064	0.611763	0.357504
C	4.299607	0.190910	-3.443397

H 2.350309 -0.543887 -2.906732
C 5.515871 0.696996 -2.982621
H 6.664441 1.244995 -1.240642
H 4.124156 0.082787 -4.510100
H 6.291997 0.981723 -3.687154
H 2.557968 0.212479 0.724779
H -3.091182 1.993983 -0.489435
H -0.090301 3.011360 -0.802505

^{3}TS6

B3LYP/BSI SCF energy: -2405.624042a.u.

M06/BSII SCF energy in solution: -2404.519758a.u.

M06/BSII free energy in solution: -2403.718582a.u.

C 1.158441 -1.562096 -0.625883
N 1.433089 -0.299574 -0.794652
O 2.070757 -2.278405 0.091083
C 2.965810 -1.312520 0.726821
C 2.759200 -0.044731 -0.212927
C -1.250539 -1.459555 -0.776938
N -1.380571 -0.157413 -0.923310
O -2.354833 -2.105149 -0.279898
C -3.471375 -1.179992 -0.407592
C -2.714376 0.215589 -0.419462
Ni 0.062084 1.089812 -1.343613
C -0.054523 -2.285334 -1.177058
C 0.040696 -2.353221 -2.733141
H 0.923993 -2.928712 -3.031539
H 0.112768 -1.348273 -3.157705
H -0.851110 -2.841255 -3.137993
C -0.151742 -3.713926 -0.609362
H -1.030444 -4.214668 -1.019836
H -0.236971 -3.710755 0.479606
H 0.737518 -4.285756 -0.882900
C -4.420977 -1.412984 0.765878
C -5.728641 -0.909733 0.735398
C -3.998547 -2.115112 1.901374
C -6.589398 -1.096387 1.816477
H -6.086608 -0.381686 -0.142171
C -4.864180 -2.310401 2.978618
H -2.992770 -2.515399 1.933561
C -6.161724 -1.800245 2.943170
H -7.599077 -0.697892 1.772241
H -4.519721 -2.863946 3.847769
H -6.834511 -1.952434 3.782286
C -4.163654 -1.477716 -1.751364
C -4.825374 -0.496432 -2.500552
C -4.187890 -2.799515 -2.221324
C -5.481143 -0.822329 -3.690879
H -4.842877 0.536461 -2.167432
C -4.836780 -3.125350 -3.411147
H -3.691252 -3.572331 -1.645524
C -5.486010 -2.136820 -4.153662
H -5.983142 -0.041834 -4.255501
H -4.837557 -4.155302 -3.757016
H -5.991050 -2.389628 -5.081367
C 2.438309 -1.071973 2.151129
C 2.637117 0.134582 2.835415
C 1.783723 -2.116465 2.821776
C 2.189399 0.296556 4.149206
H 3.149019 0.963095 2.356129
C 1.329865 -1.953846 4.129971
H 1.632611 -3.059389 2.308786
C 1.530926 -0.745612 4.801165

H	2.356719	1.241329	4.658440
H	0.822194	-2.775400	4.627474
H	1.182362	-0.620301	5.822232
C	4.369311	-1.916222	0.739539
C	4.722660	-2.914277	-0.178410
C	5.337309	-1.469370	1.648812
C	6.010778	-3.449610	-0.186165
H	3.982490	-3.278286	-0.880317
C	6.627933	-1.998859	1.634259
H	5.083459	-0.714083	2.384681
C	6.970504	-2.992998	0.717631
H	6.262377	-4.226925	-0.902231
H	7.362429	-1.638080	2.348781
H	7.973231	-3.410537	0.710748
C	0.947775	2.861682	-0.893988
H	2.001205	2.977021	-1.163664
H	0.898778	2.598052	0.175399
C	0.157790	4.141052	-1.168107
H	0.196707	4.376698	-2.240484
H	-0.903926	3.983514	-0.930612
C	0.667279	5.350914	-0.367250
H	1.731507	5.509559	-0.594036
H	0.614920	5.121987	0.706773
C	-0.109910	6.643772	-0.648296
H	-0.056068	6.873735	-1.722227
H	-1.174815	6.482239	-0.426322
C	0.394587	7.851141	0.152315
H	1.459045	8.011196	-0.067842
H	0.338892	7.621741	1.225360
C	-0.384815	9.138424	-0.135728
H	-0.319862	9.411752	-1.195241
H	-0.000171	9.980948	0.448787
H	-1.446967	9.021701	0.108909
C	-2.643599	0.955251	0.907015
C	-1.730401	0.594028	1.907801
C	-3.507976	2.031623	1.143354
C	-1.694043	1.285829	3.117960
H	-1.037752	-0.223858	1.736778
C	-3.472789	2.727221	2.353872
H	-4.214946	2.330582	0.372676
C	-2.566548	2.353971	3.346053
H	-0.977446	0.991018	3.878949
H	-4.148974	3.561893	2.516760
H	-2.534484	2.894574	4.287879
C	3.827248	0.193532	-1.270095
C	4.914050	1.024745	-0.969238
C	3.760907	-0.390812	-2.540388
C	5.920311	1.256255	-1.907029
H	4.973401	1.497706	0.008288
C	4.765521	-0.160588	-3.481123
H	2.908867	-1.009103	-2.801157
C	5.849570	0.661195	-3.167553
H	6.753808	1.906112	-1.655543
H	4.696138	-0.618116	-4.464132
H	6.628479	0.843546	-3.902442
H	2.707010	0.852211	0.405418
H	-3.192744	0.877486	-1.141819
H	0.701573	2.026425	-2.302348

^{1}[Ni^{0}]

B3LYP/BSI SCF energy: -2168.542621a.u.

M06/BSII SCF energy in solution: -2167.596388a.u.

M06/BSII free energy in solution: -2166.966887a.u.

C	1.267990	-0.210762	-0.453720
N	1.516939	-0.190868	0.816229
O	2.355354	-0.312711	-1.277100
C	3.518606	-0.634640	-0.450236
C	2.987380	-0.257223	1.001926
H	3.186054	-1.082244	1.684735
C	3.543271	1.007049	1.632217
C	4.658115	0.917356	2.474423
C	2.976476	2.265495	1.398014
C	5.207340	2.058416	3.059463
H	5.100120	-0.055166	2.678396
C	3.521671	3.408236	1.984490
H	2.092219	2.346993	0.774860
C	4.639726	3.309585	2.814317
H	6.071835	1.968430	3.711151
H	3.066656	4.376929	1.797437
H	5.060884	4.199844	3.272708
C	-1.353352	0.019139	-0.443269
N	-1.593504	0.013760	0.831965
O	-2.440085	0.178453	-1.257961
C	-3.552220	0.629933	-0.407350
C	-3.064902	0.145127	1.018392
H	-3.250968	0.933309	1.748170
C	-3.669183	-1.134912	1.564845
C	-4.601503	-1.064233	2.606380
C	-3.315137	-2.394467	1.064156
C	-5.180075	-2.221439	3.130109
H	-4.872508	-0.094482	3.016971
C	-3.890030	-3.552344	1.585727
H	-2.576033	-2.471511	0.273599
C	-4.826449	-3.470140	2.618907
H	-5.899151	-2.146130	3.940913
H	-3.601976	-4.521611	1.188467
H	-5.270601	-4.373694	3.026737
Ni	-0.039661	-0.089028	1.814762
C	-0.049867	-0.144023	-1.228883
C	-0.178951	-1.471378	-2.034287
H	0.695638	-1.608544	-2.672410
H	-0.251016	-2.327177	-1.356755
H	-1.073485	-1.438330	-2.659798
C	0.057781	1.055860	-2.214816
H	-0.839043	1.109622	-2.833088
H	0.154371	1.998142	-1.667243
H	0.931634	0.931296	-2.856679
C	-3.578704	2.161914	-0.545901
C	-4.245810	2.740489	-1.637923
C	-2.866377	3.007557	0.318700
C	-4.220302	4.117767	-1.849252
H	-4.793044	2.105278	-2.326132
C	-2.841983	4.388535	0.107129
H	-2.309008	2.605251	1.157841
C	-3.520904	4.950106	-0.973094
H	-4.749657	4.540120	-2.698752
H	-2.287819	5.022247	0.793742
H	-3.504556	6.024504	-1.132554
C	-4.838961	-0.017463	-0.891924
C	-6.059571	0.377876	-0.323932
C	-4.844749	-1.010100	-1.876848
C	-7.255614	-0.211047	-0.727551
H	-6.073737	1.158395	0.431293
C	-6.045498	-1.596367	-2.285289
H	-3.908913	-1.318147	-2.327320
C	-7.253671	-1.202086	-1.712549
H	-8.190457	0.106597	-0.274623

H -6.031805 -2.362924 -3.055095
H -8.186309 -1.659157 -2.030359
C 4.697972 0.193861 -0.957281
C 6.013920 -0.187548 -0.660887
C 4.490267 1.361688 -1.702317
C 7.093360 0.584099 -1.089305
H 6.200263 -1.099145 -0.102755
C 5.571654 2.128337 -2.138433
H 3.480114 1.665484 -1.948802
C 6.877542 1.745716 -1.832137
H 8.105004 0.270211 -0.848057
H 5.389353 3.027523 -2.720371
H 7.718438 2.343354 -2.171979
C 3.788551 -2.138869 -0.624499
C 3.614545 -2.706466 -1.896588
C 4.264664 -2.957416 0.406504
C 3.883179 -4.054393 -2.123098
H 3.266378 -2.080722 -2.711005
C 4.541167 -4.309023 0.179331
H 4.432918 -2.556826 1.400848
C 4.346439 -4.864333 -1.083389
H 3.734489 -4.472112 -3.114885
H 4.905158 -4.924258 0.997135
H 4.556050 -5.915560 -1.258200

^{1}[Ni^0]·2

B3LYP/BSI SCF energy: -2404.4814a.u.

M06/BSII SCF energy in solution: -2403.401699a.u.

M06/BSII free energy in solution: -2402.611152a.u.

C -1.402194 -0.074594 -1.073594
N -1.411398 0.276379 0.160973
O -2.598815 -0.232667 -1.686005
C -3.620039 0.312983 -0.762410
C -2.820406 0.310854 0.607116
C 1.046347 -0.488589 -1.107204
N 1.382291 0.187318 -0.065730
O 1.873399 -1.487717 -1.495123
C 2.820792 -1.701493 -0.379698
C 2.710553 -0.310072 0.369743
Ni 0.091739 1.143215 1.124743
C -0.187203 -0.263288 -1.965484
C -0.419927 -1.452997 -2.922719
H -1.306658 -1.265735 -3.530183
H -0.559411 -2.388315 -2.374897
H 0.441470 -1.575285 -3.581841
C 0.003678 1.043629 -2.789016
H 0.841546 0.925418 -3.483201
H 0.205481 1.889348 -2.128153
H -0.900145 1.251159 -3.367864
C 4.192278 -2.014869 -0.954031
C 5.234940 -2.356376 -0.079865
C 4.450629 -1.970700 -2.327395
C 6.508895 -2.636383 -0.568806
H 5.045633 -2.412037 0.988174
C 5.727137 -2.258800 -2.817565
H 3.650977 -1.716332 -3.012684
C 6.760354 -2.589208 -1.942321
H 7.304931 -2.895346 0.123346
H 5.909449 -2.224302 -3.887996
H 7.752435 -2.811607 -2.324406
C 2.244788 -2.875023 0.429524
C 1.359897 -2.681451 1.501716
C 2.528299 -4.190972 0.029412

C	0.795105	-3.773649	2.165374
H	1.080760	-1.683552	1.824384
C	1.963927	-5.279837	0.691357
H	3.200621	-4.363132	-0.804179
C	1.097858	-5.075185	1.767354
H	0.111078	-3.596014	2.989606
H	2.204233	-6.288420	0.366948
H	0.662829	-5.923133	2.288967
C	-3.919287	1.729668	-1.278014
C	-3.183750	2.846505	-0.848957
C	-4.880885	1.912529	-2.284415
C	-3.421507	4.108807	-1.397603
H	-2.408233	2.748557	-0.096106
C	-5.116209	3.172850	-2.831731
H	-5.452448	1.061921	-2.639677
C	-4.389679	4.278609	-2.386593
H	-2.844160	4.958879	-1.045582
H	-5.869960	3.289530	-3.605304
H	-4.575891	5.262451	-2.807765
C	-4.839988	-0.591496	-0.791043
C	-4.860011	-1.789096	-1.512443
C	-5.980263	-0.220615	-0.062682
C	-5.997100	-2.601334	-1.504309
H	-3.987284	-2.082607	-2.083390
C	-7.110920	-1.034197	-0.050184
H	-5.984923	0.713852	0.490809
C	-7.124091	-2.229698	-0.773081
H	-5.997510	-3.526496	-2.073830
H	-7.983409	-0.732385	0.522067
H	-8.006828	-2.862495	-0.767029
C	0.755169	2.411059	2.446486
H	-2.993054	1.250552	1.130333
C	-3.116312	-0.812881	1.586252
C	-3.804296	-0.525244	2.770864
C	-2.719457	-2.133774	1.343630
C	-4.102940	-1.533398	3.688777
H	-4.101410	0.499126	2.980676
C	-3.016941	-3.144123	2.257797
H	-2.167676	-2.376902	0.441313
C	-3.712050	-2.847851	3.432702
H	-4.634085	-1.289437	4.604404
H	-2.702259	-4.163257	2.052007
H	-3.941809	-3.635145	4.145102
H	2.684900	-0.474793	1.446862
C	3.813779	0.693122	0.082356
C	4.776987	0.952606	1.063570
C	3.908895	1.360100	-1.145345
C	5.817585	1.851457	0.825511
H	4.706832	0.453688	2.026857
C	4.945822	2.260499	-1.386632
H	3.163918	1.184362	-1.914700
C	5.905172	2.508151	-0.402195
H	6.552722	2.044000	1.601685
H	5.003124	2.772282	-2.343181
H	6.711129	3.211764	-0.589702
C	-0.624384	2.053874	2.641308
H	-0.900172	1.360004	3.439827
H	-1.395728	2.807569	2.446631
H	1.488175	1.967811	3.131393
C	1.116053	3.836704	2.041070
H	1.060185	4.483292	2.932668
H	0.352362	4.219963	1.347950
C	2.497065	4.000396	1.394981
H	3.266751	3.619034	2.081538

H 2.545125 3.361456 0.506578
C 2.856982 5.440406 0.992096
H 3.784077 5.418024 0.403719
H 2.080647 5.831516 0.318226
C 3.041169 6.409895 2.166532
H 3.340594 7.405050 1.818615
H 2.120049 6.527238 2.746173
H 3.817508 6.050981 2.853128

^{1}**IM2**-*R*

B3LYP/BSI SCF energy: -2699.063481a.u.

M06/BSII SCF energy in solution: -2697.868744a.u.

M06/BSII free energy in solution: -2697.070272a.u.

C -1.821535 -0.390826 -1.311449
N -1.678128 -0.019764 -0.093554
O -3.081014 -0.551959 -1.762878
C -3.973081 0.044452 -0.729295
C -3.014666 0.051018 0.529891
C 0.611820 -0.794394 -1.607047
N 1.043489 -0.124237 -0.596018
O 1.422662 -1.756173 -2.092000
C 2.463931 -1.978882 -1.057398
C 2.425703 -0.591869 -0.305710
Ni -0.067660 0.941698 0.561715
C -0.712366 -0.594675 -2.329286
C -1.041402 -1.808689 -3.225204
H -1.996984 -1.643860 -3.725693
H -1.102753 -2.732630 -2.644371
H -0.264427 -1.937247 -3.980870
C -0.621781 0.696810 -3.194661
H 0.131805 0.563572 -3.976797
H -0.348969 1.558538 -2.580908
H -1.586642 0.888152 -3.671376
C 3.778746 -2.310132 -1.742107
C 4.875471 -2.681822 -0.951105
C 3.931222 -2.258213 -3.130924
C 6.102562 -2.982002 -1.538028
H 4.765985 -2.742272 0.127897
C 5.160071 -2.569489 -3.718664
H 3.088247 -1.978406 -3.751581
C 6.249299 -2.928329 -2.926386
H 6.943428 -3.262884 -0.910573
H 5.261679 -2.529149 -4.799500
H 7.204488 -3.167699 -3.384564
C 1.945839 -3.161297 -0.222371
C 1.356070 -3.014845 1.039530
C 1.990419 -4.443809 -0.796005
C 0.835531 -4.124441 1.713455
H 1.287691 -2.046730 1.521454
C 1.469095 -5.547101 -0.125898
H 2.442403 -4.574207 -1.773981
C 0.890284 -5.391193 1.136671
H 0.383165 -3.985814 2.690661
H 1.519199 -6.529475 -0.586947
H 0.489371 -6.251824 1.664690
C -4.300887 1.453275 -1.246499
C -3.556275 2.584398 -0.876231
C -5.317671 1.605576 -2.203409
C -3.836112 3.832380 -1.439588
H -2.747586 2.519590 -0.155683
C -5.593379 2.850629 -2.764626
H -5.899275 0.741556 -2.507238
C -4.853858 3.971676 -2.381753

H	-3.249019	4.694105	-1.136034
H	-6.388277	2.944471	-3.499094
H	-5.069725	4.944248	-2.814666
C	-5.208007	-0.827996	-0.587633
C	-5.349590	-2.039420	-1.271206
C	-6.236830	-0.407502	0.268200
C	-6.496894	-2.818575	-1.099169
H	-4.564350	-2.370518	-1.940417
C	-7.376612	-1.188549	0.443914
H	-6.147665	0.538873	0.793724
C	-7.511650	-2.399027	-0.240713
H	-6.593271	-3.755266	-1.640910
H	-8.161622	-0.849778	1.113759
H	-8.402136	-3.006203	-0.106729
C	1.047810	3.804559	1.255553
C	0.561222	2.384189	1.618038
C	2.319304	3.514608	2.081693
H	2.594384	4.292445	2.804918
H	3.191772	3.247376	1.474220
N	1.613620	2.367482	2.658988
O	-0.708139	2.082083	1.835212
C	2.167261	1.308140	3.348590
C	3.502269	1.351768	3.803619
C	1.390220	0.164515	3.644113
C	4.040354	0.282120	4.518625
H	4.110159	2.228153	3.600000
C	1.949965	-0.896632	4.352154
H	0.350395	0.143562	3.338069
C	3.276337	-0.854234	4.794386
H	5.070164	0.340992	4.862541
H	1.332552	-1.763176	4.577086
H	3.700082	-1.682906	5.353394
H	-3.098991	1.002753	1.053882
C	-3.190690	-1.050717	1.561697
C	-3.641144	-0.717995	2.844482
C	-2.891938	-2.390016	1.279170
C	-3.804532	-1.700892	3.822262
H	-3.852348	0.320922	3.083646
C	-3.053729	-3.374058	2.253523
H	-2.523917	-2.669516	0.296927
C	-3.512902	-3.032912	3.528126
H	-4.150228	-1.422736	4.813683
H	-2.816074	-4.407299	2.017181
H	-3.636242	-3.799906	4.287481
H	2.523207	-0.742221	0.768439
C	3.473285	0.425076	-0.725314
C	4.494598	0.759105	0.171888
C	3.466794	1.021443	-1.992098
C	5.496939	1.660717	-0.192881
H	4.500347	0.322668	1.167082
C	4.467163	1.919537	-2.359139
H	2.674588	0.790563	-2.696854
C	5.487644	2.240844	-1.461342
H	6.281133	1.909637	0.516544
H	4.447844	2.373097	-3.345920
H	6.265119	2.943130	-1.747894
Br	1.410984	4.152645	-0.734027
C	0.161222	4.926140	1.758547
H	-0.001538	4.778295	2.832617
H	0.618796	5.906403	1.598617
H	-0.812358	4.897896	1.264472

[1]**IM2**-*S*

B3LYP/BSI SCF energy: -2699.065264a.u.

M06/BSII SCF energy in solution: -2697.870805a.u.

M06/BSII free energy in solution: -2697.072415a.u.

C	-1.642965	-1.094953	-1.201623
N	-1.555799	-0.448291	-0.099059
O	-2.881501	-1.392912	-1.644110
C	-3.816386	-0.570820	-0.820440
C	-2.920406	-0.289170	0.446572
C	0.837190	-1.319520	-1.401978
N	1.166648	-0.349147	-0.620413
O	1.793503	-2.229429	-1.676136
C	2.880512	-2.003479	-0.690283
C	2.611940	-0.498437	-0.303634
Ni	-0.070474	0.817976	0.269138
C	-0.498716	-1.527730	-2.099891
C	-0.688769	-3.015193	-2.479954
H	-1.656043	-3.144988	-2.968418
H	-0.648469	-3.660179	-1.598112
H	0.099560	-3.331203	-3.164984
C	-0.527118	-0.647723	-3.383220
H	0.262485	-0.968579	-4.069181
H	-0.377409	0.405905	-3.135720
H	-1.491798	-0.757958	-3.883856
C	4.218551	-2.268789	-1.358417
C	5.381598	-2.226751	-0.575698
C	4.330023	-2.548193	-2.723794
C	6.630785	-2.446986	-1.151248
H	5.307514	-2.029013	0.489783
C	5.583228	-2.777856	-3.297759
H	3.436756	-2.591346	-3.335790
C	6.736387	-2.724837	-2.516468
H	7.521966	-2.406888	-0.531758
H	5.653280	-2.998964	-4.359025
H	7.709854	-2.901930	-2.964244
C	2.601052	-2.997302	0.448307
C	1.972741	-2.633523	1.647226
C	2.909088	-4.352067	0.237661
C	1.674193	-3.599308	2.613081
H	1.702629	-1.604538	1.855152
C	2.607603	-5.313222	1.198995
H	3.392923	-4.651552	-0.686360
C	1.989623	-4.938632	2.394863
H	1.191904	-3.292998	3.536414
H	2.859469	-6.353974	1.016179
H	1.759221	-5.685503	3.149238
C	-4.099648	0.685819	-1.658524
C	-3.495580	1.927888	-1.411917
C	-4.947784	0.560783	-2.772071
C	-3.750068	3.017417	-2.251784
H	-2.818879	2.081385	-0.577665
C	-5.193898	1.645226	-3.610189
H	-5.421683	-0.394048	-2.976908
C	-4.597006	2.881772	-3.349833
H	-3.278111	3.970861	-2.032850
H	-5.856762	1.526491	-4.462716
H	-4.794323	3.731490	-3.997274
C	-5.074677	-1.378063	-0.555561
C	-5.198567	-2.718005	-0.935358
C	-6.146546	-0.756215	0.101571
C	-6.372493	-3.424572	-0.660847
H	-4.379715	-3.205973	-1.450455
C	-7.312495	-1.464694	0.382456
H	-6.070217	0.289169	0.386349
C	-7.430669	-2.803379	0.000435

H	-6.455581	-4.463570	-0.967144
H	-8.131498	-0.968907	0.895360
H	-8.341947	-3.354244	0.214310
C	0.691486	3.824896	0.135421
C	0.298623	2.585413	0.951353
C	1.883959	3.993440	1.100217
H	2.001518	4.975134	1.565854
H	2.833140	3.687870	0.635673
N	1.288934	2.971777	1.971021
O	-0.908677	2.135051	1.204342
C	1.961190	2.179104	2.878580
C	3.277956	2.485740	3.283584
C	1.325815	1.051793	3.449172
C	3.935736	1.684396	4.216538
H	3.774548	3.361825	2.877654
C	2.003230	0.260507	4.373945
H	0.297759	0.837795	3.180581
C	3.311953	0.561849	4.765427
H	4.947333	1.945401	4.517807
H	1.491736	-0.594686	4.809562
H	3.827676	-0.055843	5.494309
C	0.915750	3.752023	-1.355145
H	1.667273	2.984446	-1.564167
H	1.266500	4.707886	-1.754517
H	-0.009957	3.476898	-1.868148
H	-3.039529	0.742827	0.771540
C	-3.125226	-1.173227	1.665811
C	-3.649985	-0.608769	2.834337
C	-2.789352	-2.533502	1.667184
C	-3.847637	-1.385925	3.977055
H	-3.893606	0.450200	2.853444
C	-2.984905	-3.312134	2.807003
H	-2.369182	-2.990120	0.776398
C	-3.516864	-2.740916	3.965557
H	-4.251483	-0.929063	4.875904
H	-2.719863	-4.365560	2.790904
H	-3.666539	-3.347697	4.854063
H	2.749841	-0.357039	0.767153
C	3.451688	0.546787	-1.018252
C	4.390277	1.285236	-0.287642
C	3.321683	0.792708	-2.391932
C	5.191207	2.241106	-0.917075
H	4.483670	1.125444	0.783279
C	4.119069	1.747597	-3.021635
H	2.592955	0.239579	-2.975913
C	5.058756	2.473841	-2.286045
H	5.913109	2.806221	-0.334471
H	4.006056	1.925896	-4.087194
H	5.678490	3.218639	-2.776790
Br	-0.684547	5.375454	0.449500

4

B3LYP/BSI SCF energy: -237.100557a.u.

M06/BSII SCF energy in solution: -236.944231a.u.

M06/BSII free energy in solution: -236.786731a.u.

C	-1.415104	2.904624	0.000000
H	-0.901040	3.299813	0.883884
H	-0.901040	3.299813	-0.883884
C	-1.415104	1.372298	0.000000
H	-1.965666	1.005857	0.877353
H	-1.965666	1.005857	-0.877353
C	-0.005504	0.766926	0.000000
H	0.545688	1.134307	0.877862

H	0.545688	1.134307	-0.877862
C	0.005504	-0.766926	0.000000
H	-0.545688	-1.134307	0.877862
H	-0.545688	-1.134307	-0.877862
C	1.415104	-1.372298	0.000000
H	1.965666	-1.005857	0.877353
H	1.965666	-1.005857	-0.877353
C	1.415104	-2.904624	0.000000
H	0.901040	-3.299813	0.883884
H	2.433753	-3.306781	0.000000
H	0.901040	-3.299813	-0.883884
H	-2.433753	3.306781	0.000000

^{1}TS7-*R*

B3LYP/BSI SCF energy: -2699.059166a.u.

M06/BSII SCF energy in solution: -2697.871105a.u.

M06/BSII free energy in solution: -2697.076798a.u.

C	1.228439	-1.733840	-0.851582
N	1.400660	-0.688754	-0.116651
O	2.324785	-2.469642	-1.110457
C	3.385519	-2.020446	-0.188082
C	2.850457	-0.576792	0.177851
C	-1.294618	-1.689900	-0.929003
N	-1.399596	-0.751256	-0.065253
O	-2.438465	-2.237759	-1.377719
C	-3.535016	-1.327996	-0.922042
C	-2.835769	-0.608088	0.288341
Ni	0.001550	0.510677	0.502924
C	-0.026651	-2.235654	-1.549674
C	0.025206	-1.759775	-3.032299
H	0.920596	-2.155026	-3.518558
H	0.034769	-0.668436	-3.094546
H	-0.856163	-2.127573	-3.562022
C	-0.070052	-3.784376	-1.502258
H	-0.979893	-4.136193	-1.990534
H	-0.059484	-4.150033	-0.471691
H	0.794327	-4.197253	-2.023574
C	-4.753605	-2.159271	-0.566261
C	-5.897177	-1.497829	-0.095520
C	-4.782172	-3.549995	-0.705200
C	-7.040066	-2.217015	0.245068
H	-5.894110	-0.415995	0.001861
C	-5.932602	-4.269259	-0.369324
H	-3.907031	-4.070725	-1.076057
C	-7.062145	-3.607556	0.109031
H	-7.914881	-1.690597	0.614937
H	-5.940779	-5.349475	-0.483736
H	-7.954505	-4.167749	0.372072
C	-3.798541	-0.396423	-2.116105
C	-3.249263	0.891113	-2.213438
C	-4.548063	-0.887133	-3.198017
C	-3.452920	1.667327	-3.358009
H	-2.659534	1.316013	-1.409061
C	-4.748576	-0.113569	-4.338975
H	-4.981980	-1.880043	-3.140778
C	-4.202309	1.169546	-4.422502
H	-3.021312	2.662908	-3.402809
H	-5.337114	-0.511385	-5.160758
H	-4.363926	1.776203	-5.308885
C	3.362479	-2.988539	1.005027
C	3.632113	-2.583135	2.317958
C	3.126212	-4.350697	0.761576
C	3.645114	-3.510371	3.363994

H	3.842214	-1.544377	2.550609
C	3.133370	-5.274050	1.804787
H	2.936116	-4.685120	-0.252245
C	3.390419	-4.856837	3.112804
H	3.850877	-3.171119	4.374681
H	2.942704	-6.322456	1.594427
H	3.395400	-5.575709	3.926535
C	4.713527	-2.072361	-0.935823
C	4.757237	-2.007475	-2.334345
C	5.918776	-2.156544	-0.226181
C	5.979336	-2.024773	-3.006760
H	3.834617	-1.953935	-2.899859
C	7.140286	-2.162055	-0.899555
H	5.907323	-2.231032	0.856036
C	7.175619	-2.097741	-2.292744
H	5.993617	-1.981083	-4.091926
H	8.063901	-2.226976	-0.331983
H	8.126341	-2.109988	-2.817393
C	1.062342	2.295037	1.694849
C	-0.204168	2.603172	1.087725
C	1.651692	3.480588	0.917846
H	2.408106	3.240977	0.165551
H	1.948698	4.316799	1.556743
N	0.294091	3.655152	0.340622
O	-1.286215	1.942740	1.004429
C	-0.203178	4.445666	-0.682111
C	0.665218	5.263941	-1.425057
C	-1.580937	4.447996	-0.976872
C	0.163308	6.057382	-2.454596
H	1.724180	5.278855	-1.187890
C	-2.062201	5.247647	-2.010420
H	-2.251926	3.845573	-0.376366

C	-1.198997	6.052710	-2.760185
H	0.845124	6.688817	-3.017676
H	-3.129023	5.255384	-2.218063
H	-1.585361	6.678170	-3.559013
C	1.507113	1.614680	2.943174
H	1.646520	2.364849	3.730246
H	2.455525	1.080452	2.798605
H	0.751591	0.905966	3.293552
H	2.959993	-0.408224	1.247207
C	3.497536	0.591946	-0.546501
C	4.608001	1.214973	0.038601
C	3.030696	1.065611	-1.777997
C	5.244969	2.280742	-0.595933
H	4.976031	0.868527	1.000995
C	3.661251	2.138319	-2.411543
H	2.157642	0.614318	-2.237017
C	4.771694	2.746657	-1.824520
H	6.101995	2.753610	-0.125800
H	3.278406	2.501762	-3.360559
H	5.260443	3.582553	-2.316030
H	-3.081320	0.451354	0.290214
C	-3.078404	-1.133383	1.693512
C	-3.497082	-0.237060	2.683672
C	-2.840425	-2.469983	2.044699
C	-3.688817	-0.667618	3.998169
H	-3.645013	0.809162	2.432981
C	-3.035036	-2.901734	3.355330
H	-2.510304	-3.180571	1.292844
C	-3.461585	-2.001416	4.335559
H	-4.002353	0.044512	4.755297
H	-2.853246	-3.941408	3.612740
H	-3.607939	-2.338501	5.357683

Br -0.143300 4.582868 3.757864

[1]TS7-*S*

B3LYP/BSI SCF energy: -2699.059542a.u.

M06/BSII SCF energy in solution: -2697.8683764a.u.

M06/BSII free energy in solution: -2697.07314a.u.

C	-1.154229	-1.693144	-0.966154
N	-1.233353	-0.813265	-0.033194
O	-2.298288	-2.253654	-1.378571
C	-3.397777	-1.393268	-0.824245
C	-2.647781	-0.741113	0.398594
C	1.345862	-1.633001	-0.997605
N	1.512376	-0.481471	-0.441039
O	2.396233	-2.464458	-0.988690
C	3.395560	-1.900441	-0.042661
C	2.918230	-0.391356	0.028660
Ni	0.055751	0.707313	0.030523
C	0.097056	-2.142353	-1.701866
C	0.108108	-3.687358	-1.780778
H	-0.801680	-4.028930	-2.276422
H	0.159094	-4.139933	-0.786964
H	0.972901	-4.028157	-2.352198
C	0.057442	-1.544027	-3.138727
H	0.923341	-1.894280	-3.708158
H	0.065379	-0.452290	-3.112384
H	-0.852273	-1.873640	-3.645244
C	4.784978	-2.102632	-0.621342
C	5.898339	-1.732327	0.147476
C	4.989695	-2.635666	-1.897577
C	7.188336	-1.882643	-0.355944
H	5.755740	-1.332173	1.147032
C	6.284898	-2.792136	-2.398375
H	4.138349	-2.933075	-2.498226
C	7.386579	-2.414438	-1.632652
H	8.039313	-1.587844	0.250879
H	6.427805	-3.211677	-3.389955
H	8.392417	-2.535348	-2.023636
C	3.176679	-2.667812	1.270253
C	2.253397	-2.239623	2.237700
C	3.831353	-3.893272	1.471373
C	2.007853	-3.005575	3.379138
H	1.700758	-1.314594	2.115237
C	3.586800	-4.656856	2.611723
H	4.539783	-4.250286	0.732070
C	2.676333	-4.213985	3.572711
H	1.289272	-2.651511	4.112152
H	4.110837	-5.598274	2.748466
H	2.489446	-4.806189	4.463725
C	-3.733456	-0.400016	-1.944191
C	-3.431785	0.968153	-1.890511
C	-4.322969	-0.913612	-3.113595
C	-3.718568	1.807290	-2.974087
H	-2.967999	1.426858	-1.024393
C	-4.601968	-0.081182	-4.194027
H	-4.566259	-1.970512	-3.169880
C	-4.302187	1.283536	-4.125927
H	-3.468301	2.862608	-2.888501
H	-5.060544	-0.496692	-5.087254
H	-4.526584	1.934225	-4.966376
C	-4.572622	-2.283496	-0.463255
C	-4.451054	-3.673001	-0.356464
C	-5.813224	-1.686919	-0.195804
C	-5.550887	-4.452106	0.008992

H	-3.499304	-4.146132	-0.567881
C	-6.906172	-2.465148	0.181007
H	-5.925772	-0.611540	-0.295747
C	-6.779549	-3.852245	0.282718
H	-5.443267	-5.530863	0.078661
H	-7.859518	-1.986983	0.385935
H	-7.633623	-4.459580	0.567877
C	0.550886	2.858244	-0.744412
C	-0.357086	2.838724	0.376848
C	1.381860	3.882416	0.038063
H	1.387408	4.881616	-0.408135
H	2.388389	3.569878	0.334034
N	0.382732	3.733264	1.125726
O	-1.266531	2.012035	0.698738
C	0.408741	4.095950	2.466609
C	1.445884	4.913379	2.946520
C	-0.601142	3.661661	3.346107
C	1.475830	5.280559	4.290305
H	2.213377	5.267121	2.265106
C	-0.552659	4.039992	4.684734
H	-1.407219	3.045633	2.966504
C	0.481513	4.846322	5.168718
H	2.280387	5.916582	4.648949
H	-1.337430	3.703153	5.356400
H	0.507062	5.138342	6.214070
C	0.448528	2.644836	-2.216571
H	1.340198	2.142128	-2.608064
H	0.340781	3.614887	-2.714226
H	-0.441162	2.062475	-2.468191
H	-2.926084	0.305540	0.490860
C	-2.840202	-1.386016	1.761974
C	-3.716396	-0.785905	2.675157
C	-2.169265	-2.554763	2.143229
C	-3.928220	-1.345234	3.935626
H	-4.231394	0.130223	2.398469
C	-2.376400	-3.114444	3.404419
H	-1.470690	-3.028287	1.460523
C	-3.258530	-2.512802	4.303945
H	-4.609783	-0.863666	4.630690
H	-1.847387	-4.021507	3.683413
H	-3.418338	-2.947805	5.286324
H	2.916115	-0.070673	1.071051
C	3.716730	0.629571	-0.761037
C	4.504729	1.559695	-0.072581
C	3.700339	0.664261	-2.161348
C	5.265608	2.503579	-0.765445
H	4.523075	1.546938	1.014656
C	4.457212	1.607172	-2.855985
H	3.092049	-0.044411	-2.714715
C	5.242305	2.529512	-2.160080
H	5.869714	3.219045	-0.215378
H	4.431943	1.624149	-3.941527
H	5.827931	3.265539	-2.702563
Br	-1.942117	5.013270	-1.417146

^{1}**IM3**-R^{+}

B3LYP/BSI SCF energy: -2685.513197a.u.

M06/BSII SCF energy in solution: -2684.357353a.u.

M06/BSII free energy in solution: -2683.554229a.u.

C	1.255771	-1.003230	-1.034898
N	1.403811	-0.295081	0.038785
O	2.353384	-1.588969	-1.509777
C	3.392220	-1.520889	-0.448210

C	2.855877	-0.284385	0.371669
C	-1.249082	-0.955718	-1.099126
N	-1.356714	-0.353480	0.032912
O	-2.376850	-1.367722	-1.671279
C	-3.502883	-0.717942	-0.917119
C	-2.784318	-0.406954	0.448809
Ni	0.016683	0.577349	1.016893
C	0.015650	-1.216891	-1.882561
C	0.059332	-0.212985	-3.074952
H	0.958860	-0.387059	-3.670018
H	0.053592	0.822283	-2.724098
H	-0.816613	-0.369219	-3.707882
C	-0.008670	-2.668164	-2.426515
H	-0.913776	-2.818947	-3.015717
H	0.009778	-3.400269	-1.614536
H	0.860552	-2.838734	-3.062189
C	-4.658986	-1.695984	-0.824237
C	-5.886668	-1.232897	-0.328584
C	-4.535370	-3.039960	-1.190231
C	-6.966649	-2.102453	-0.192679
H	-6.001752	-0.187205	-0.058335
C	-5.623411	-3.906915	-1.063426
H	-3.595177	-3.409970	-1.581913
C	-6.838824	-3.443518	-0.561865
H	-7.910482	-1.730206	0.193881
H	-5.516761	-4.946142	-1.359731
H	-7.682824	-4.119267	-0.463516
C	-3.845853	0.531569	-1.737343
C	-3.418490	1.822217	-1.392474
C	-4.553420	0.359261	-2.939245
C	-3.702873	2.911135	-2.221794
H	-2.865403	2.010134	-0.478943
C	-4.832088	1.444143	-3.766666
H	-4.892524	-0.632075	-3.221472
C	-4.409317	2.727005	-3.408769
H	-3.367467	3.901624	-1.928889
H	-5.385636	1.288792	-4.687759
H	-4.633114	3.574608	-4.049494
C	3.300858	-2.848847	0.317283
C	3.452339	-2.943847	1.705920
C	3.129501	-4.031328	-0.421023
C	3.413967	-4.187454	2.344255
H	3.618955	-2.061088	2.314533
C	3.083671	-5.269041	0.216063
H	3.039138	-3.978680	-1.500614
C	3.223552	-5.351916	1.603772
H	3.534964	-4.238479	3.421984
H	2.949109	-6.171177	-0.373081
H	3.192919	-6.316758	2.100367
C	4.747993	-1.344375	-1.118388
C	4.857788	-0.850953	-2.424233
C	5.916183	-1.659090	-0.410929
C	6.111955	-0.676629	-3.010442
H	3.964776	-0.614564	-2.990578
C	7.168722	-1.470756	-0.994110
H	5.851711	-2.067653	0.592294
C	7.271262	-0.980951	-2.296898
H	6.179916	-0.303942	-4.028066
H	8.063840	-1.720496	-0.432673
H	8.246335	-0.845281	-2.754536
C	1.062110	1.681829	2.431999
C	-0.242265	2.283788	2.288683
C	1.608937	3.104377	2.149136
H	2.130443	3.243100	1.200746

H	2.165788	3.567786	2.968957
N	0.180045	3.566067	2.070633
O	-1.312754	1.649415	2.017076
C	-0.395579	4.632182	1.350096
C	0.423683	5.641067	0.826819
C	-1.789165	4.705478	1.193475
C	-0.149777	6.708526	0.136191
H	1.499337	5.595187	0.963568
C	-2.345593	5.779839	0.501071
H	-2.421512	3.937083	1.623051
C	-1.533016	6.783341	-0.033005
H	0.491096	7.487716	-0.265163
H	-3.424673	5.838077	0.391891
H	-1.974491	7.619997	-0.564810
C	1.527317	0.818845	3.581451
H	1.637165	1.414269	4.498514
H	2.501084	0.360451	3.381899
H	0.814718	0.015261	3.790643
H	2.958191	-0.479320	1.435761
C	3.508763	1.052674	0.068258
C	4.555037	1.491135	0.890083
C	3.121507	1.851468	-1.014604
C	5.203310	2.700278	0.636714
H	4.870375	0.881099	1.732797
C	3.764424	3.065013	-1.267075
H	2.309147	1.538453	-1.662418
C	4.807438	3.492569	-0.442837
H	6.014016	3.023329	1.282659
H	3.451389	3.674295	-2.109704
H	5.308993	4.434774	-0.641292
H	-3.082604	0.575233	0.806280
C	-2.979313	-1.388712	1.591865
C	-3.777509	-1.012136	2.678701
C	-2.384338	-2.657294	1.598127
C	-3.987835	-1.886800	3.745386
H	-4.233414	-0.025847	2.693575
C	-2.590292	-3.532013	2.664710
H	-1.756220	-2.971372	0.769900
C	-3.394343	-3.149590	3.740551
H	-4.609583	-1.578686	4.580462
H	-2.124751	-4.513066	2.654402
H	-3.554226	-3.830890	4.570699

[1]IM3-*S*+

B3LYP/BSI SCF energy: -2685.510646a.u.

M06/BSII SCF energy in solution: -2684.35822a.u.

M06/BSII free energy in solution: -2683.554669a.u.

C	-1.336837	-1.629123	0.321610
N	-1.382723	-0.504980	-0.305864
O	-2.497487	-2.159213	0.692279
C	-3.570895	-1.391639	-0.028343
C	-2.797283	-0.052041	-0.332636
C	1.146580	-1.591052	0.542739
N	1.375719	-0.607145	-0.268916
O	2.137625	-1.924543	1.362935
C	3.124973	-0.797695	1.333890
C	2.799603	-0.198733	-0.084730
Ni	0.063610	0.323582	-1.283786
C	-0.103978	-2.445463	0.645441
C	-0.234795	-3.039272	2.068750
H	-1.147315	-3.632770	2.132240
H	-0.270613	-2.254869	2.829098
H	0.621329	-3.679669	2.283147

C	-0.005430	-3.599250	-0.398599
H	0.859805	-4.227168	-0.170427
H	0.091966	-3.209705	-1.414869
H	-0.906064	-4.214763	-0.344116
C	4.523721	-1.364598	1.490245
C	5.585083	-0.476773	1.719666
C	4.789881	-2.734131	1.393463
C	6.889758	-0.951232	1.837796
H	5.389680	0.586819	1.821544
C	6.097270	-3.208066	1.524383
H	3.978426	-3.432975	1.228067
C	7.150007	-2.320226	1.742043
H	7.701503	-0.252338	2.014976
H	6.288775	-4.274761	1.457455
H	8.165337	-2.690921	1.843242
C	2.723734	0.105819	2.507019
C	2.016764	1.306693	2.354843
C	3.015857	-0.337442	3.808591
C	1.627110	2.052160	3.471617
H	1.756409	1.687995	1.373922
C	2.622032	0.401767	4.920851
H	3.561698	-1.264870	3.946812
C	1.928566	1.603665	4.756049
H	1.090365	2.984948	3.327884
H	2.863500	0.042956	5.916712
H	1.631026	2.186048	5.622780
C	-3.878210	-2.236354	-1.270207
C	-3.480310	-1.888561	-2.568324
C	-4.538411	-3.462591	-1.077095
C	-3.751257	-2.737726	-3.646104
H	-2.962506	-0.957251	-2.769358
C	-4.799573	-4.310754	-2.149524
H	-4.854334	-3.746112	-0.078277
C	-4.409576	-3.948301	-3.441840
H	-3.447191	-2.443385	-4.646138
H	-5.315490	-5.250661	-1.978312
H	-4.621993	-4.603588	-4.281066
C	-4.767358	-1.249335	0.894438
C	-4.672475	-1.467186	2.273145
C	-5.995879	-0.849467	0.349099
C	-5.789595	-1.291226	3.092134
H	-3.731285	-1.783546	2.707131
C	-7.105987	-0.662591	1.170855
H	-6.088199	-0.694984	-0.721798
C	-7.006830	-0.885428	2.545816
H	-5.705098	-1.474124	4.159159
H	-8.050685	-0.352668	0.734604
H	-7.874020	-0.749110	3.184636
C	1.182448	1.195252	-2.785939
C	0.001977	2.002177	-2.591998
C	1.963969	2.516416	-2.589980
H	2.537447	2.855387	-3.457542
H	2.563274	2.612116	-1.680139
N	0.637593	3.205008	-2.436195
O	-1.141362	1.545020	-2.260870
C	0.322164	4.362821	-1.690665
C	1.284489	5.375285	-1.577607
C	-0.936174	4.512978	-1.089161
C	0.991024	6.530365	-0.854804
H	2.250028	5.263356	-2.061787
C	-1.214137	5.677127	-0.374183
H	-1.678693	3.729024	-1.178063
C	-0.258240	6.687697	-0.251342
H	1.740020	7.311996	-0.771477

H	-2.187188	5.786615	0.094762
H	-0.485087	7.590800	0.306278
C	1.431552	0.218998	-3.910542
H	2.292934	-0.420645	-3.710999
H	1.619270	0.755434	-4.851168
H	0.561143	-0.425751	-4.069254
H	-3.036799	0.294401	-1.335004
C	-3.028959	1.112766	0.616401
C	-3.932775	2.115244	0.240890
C	-2.383363	1.216486	1.854494
C	-4.199138	3.190029	1.089710
H	-4.434312	2.053351	-0.721335
C	-2.642799	2.294464	2.702083
H	-1.667999	0.461416	2.164986
C	-3.553511	3.282412	2.324290
H	-4.909490	3.953145	0.785801
H	-2.133400	2.359040	3.659017
H	-3.758128	4.118251	2.986466
H	2.854814	0.887758	-0.040829
C	3.684335	-0.667116	-1.226764
C	4.673094	0.195715	-1.715401
C	3.563948	-1.942751	-1.792612
C	5.522888	-0.202535	-2.748175
H	4.785766	1.186087	-1.281224
C	4.411056	-2.343471	-2.826287
H	2.804314	-2.630135	-1.434763
C	5.392138	-1.474069	-3.307989
H	6.283685	0.479856	-3.114734
H	4.304841	-3.335222	-3.255529
H	6.049636	-1.786243	-4.113422

^{2}**IM3**-*S*

B3LYP/BSI SCF energy: -2685.687081a.u.

M06/BSII SCF energy in solution: -2684.508334a.u.

M06/BSII free energy in solution: -2683.71261a.u.

C	1.700685	-1.086100	-0.839367
N	1.688171	-0.305251	0.180432
O	2.911402	-1.483061	-1.291282
C	3.906973	-1.094790	-0.269882
C	3.092527	-0.011042	0.550530
C	-0.801356	-1.400222	-0.974318
N	-1.065065	-0.763168	0.104148
O	-1.846316	-1.959233	-1.629701
C	-3.065945	-1.353429	-1.038108
C	-2.520451	-0.915833	0.368644
Ni	0.134653	0.511987	1.139392
C	0.534826	-1.601052	-1.660484
C	0.732406	-3.114742	-1.938738
H	1.667889	-3.275432	-2.476164
H	0.771036	-3.684786	-1.005897
H	-0.096912	-3.486849	-2.542228
C	0.511234	-0.817735	-3.005643
H	-0.316841	-1.172857	-3.622147
H	0.374820	0.252729	-2.829220
H	1.449132	-0.974509	-3.544293
C	-3.425148	-0.180943	-1.968356
C	-3.685151	-0.485647	-3.316334
C	-3.479019	1.156881	-1.559926
C	-3.979774	0.518948	-4.233044
H	-3.659740	-1.521019	-3.642842
C	-3.777841	2.165452	-2.484206
H	-3.300943	1.447150	-0.530223
C	-4.025624	1.853751	-3.818441

H	-4.179012	0.261003	-5.269585
H	-3.804724	3.196725	-2.144163
H	-4.256251	2.640306	-4.531513
C	-4.177920	-2.389240	-1.021260
C	-5.442581	-2.006616	-0.551279
C	-3.988855	-3.700294	-1.468435
C	-6.491386	-2.922352	-0.517445
H	-5.606575	-0.986378	-0.216550
C	-5.044908	-4.615269	-1.441902
H	-3.016929	-4.004403	-1.839132
C	-6.296980	-4.231869	-0.964566
H	-7.463311	-2.611426	-0.145399
H	-4.883378	-5.629633	-1.796102
H	-7.116623	-4.944228	-0.942681
C	5.147242	-0.562278	-0.968165
C	6.254834	-0.185939	-0.193673
C	5.218699	-0.431056	-2.358079
C	7.402967	0.321586	-0.797220
H	6.220696	-0.296407	0.886275
C	6.373253	0.073410	-2.962932
H	4.373706	-0.728381	-2.967519
C	7.466605	0.453702	-2.186760
H	8.249521	0.611983	-0.181910
H	6.413460	0.166185	-4.044496
H	8.362701	0.846938	-2.657738
C	4.182529	-2.374942	0.536082
C	5.182213	-3.263056	0.110148
C	3.391557	-2.743488	1.636410
C	5.399160	-4.470801	0.772156
H	5.798935	-3.005909	-0.743924
C	3.609457	-3.953007	2.299194
H	2.588292	-2.103539	1.986178
C	4.615782	-4.819345	1.873255
H	6.183234	-5.138174	0.426159
H	2.987680	-4.213043	3.150887
H	4.787107	-5.757408	2.393228
C	-0.617586	1.834452	2.478146
C	-1.898248	2.367616	1.926765
C	-0.068336	3.292512	2.278734
H	0.119234	3.848514	3.208831
H	0.779416	3.426226	1.598422
N	-1.367041	3.637766	1.678734
O	-3.043952	1.953837	1.719922
C	-1.803715	4.751446	0.971497
C	-0.925967	5.821991	0.725982
C	-3.133101	4.830921	0.507415
C	-1.366423	6.941505	0.021458
H	0.096185	5.771061	1.088143
C	-3.552951	5.957129	-0.196245
H	-3.809842	4.012708	0.722592
C	-2.678708	7.019189	-0.447987
H	-0.674673	7.760282	-0.159519
H	-4.582064	6.009108	-0.543078
H	-3.017594	7.894510	-0.994003
C	-0.657778	1.297604	3.912086
H	-1.286618	0.403980	3.973863
H	0.339298	1.012314	4.265905
H	-1.060076	2.035232	4.627930
C	-2.751032	-1.860066	1.538493
C	-3.458250	-1.388129	2.651513
C	-2.257913	-3.171942	1.554751
C	-3.682091	-2.217864	3.753031
H	-3.804265	-0.358268	2.657225
C	-2.481162	-4.000014	2.653729

H -1.703881 -3.554044 0.702615
C -3.197153 -3.525646 3.756043
H -4.229902 -1.836995 4.610429
H -2.098842 -5.017160 2.649351
H -3.370374 -4.171630 4.612294
H -2.913678 0.054886 0.662488
H 3.217447 -0.205610 1.616796
C 3.418832 1.453911 0.317275
C 4.022549 2.194104 1.340326
C 3.109699 2.097842 -0.888304
C 4.321421 3.546580 1.164116
H 4.252996 1.711678 2.287103
C 3.404745 3.448265 -1.066793
H 2.628011 1.545956 -1.689164
C 4.013290 4.176774 -0.041483
H 4.785974 4.106052 1.970875
H 3.154608 3.934268 -2.005274
H 4.239064 5.229936 -0.180950

Br•

B3LYP/BSI SCF energy: -13.341985a.u.

M06/BSII SCF energy in solution: -13.307356a.u.

M06/BSII free energy in solution: -13.324186a.u.

Br 0.000000 0.000000 0.000000

[1]IM4-*R*

B3LYP/BSI SCF energy: -2699.11303a.u.

M06/BSII SCF energy in solution: -2697.90701976a.u.

M06/BSII free energy in solution: -2697.108542a.u.

C -1.030232 0.774093 -1.204154
N -1.284598 0.466343 0.017397
O -1.939853 1.529972 -1.840324
C -2.865384 2.060639 -0.803902
C -2.612266 1.034409 0.368627
C 1.427906 0.157698 -1.254486
N 1.583124 0.014506 0.011111
O 2.524103 0.055392 -2.022232
C 3.706914 -0.090861 -1.141727
C 2.988201 -0.391545 0.250135
Ni 0.080492 -0.131137 1.448575
C 0.150343 0.347188 -2.060679
C 0.388144 1.392501 -3.178048
H -0.498604 1.468414 -3.807933
H 0.591301 2.383740 -2.763420
H 1.233929 1.084256 -3.793046
C -0.189535 -1.031821 -2.707473
H 0.607003 -1.306830 -3.402788
H -0.272245 -1.815728 -1.953000
H -1.127250 -0.952107 -3.264552
C 4.496025 -1.282542 -1.699904
C 5.712685 -1.110367 -2.374743
C 3.950914 -2.576292 -1.621399
C 6.387359 -2.202477 -2.923784
H 6.141769 -0.121275 -2.476904
C 4.627018 -3.663967 -2.173493
H 2.988506 -2.751520 -1.150152
C 5.850952 -3.484810 -2.820303
H 7.332075 -2.043912 -3.436070
H 4.188212 -4.654615 -2.097207
H 6.376636 -4.335182 -3.245399
C 4.471257 1.234053 -1.165400
C 5.672435 1.362956 -0.448015

C	4.003486	2.341988	-1.880770
C	6.374947	2.565790	-0.440581
H	6.060226	0.519403	0.111462
C	4.707418	3.549575	-1.871375
H	3.092419	2.263194	-2.459612
C	5.893862	3.667863	-1.151160
H	7.299288	2.640882	0.124637
H	4.323380	4.395279	-2.434734
H	6.440945	4.606087	-1.143824
C	-4.277431	2.064688	-1.365919
C	-5.307539	2.621541	-0.594145
C	-4.583429	1.535274	-2.623090
C	-6.617839	2.635764	-1.066333
H	-5.081399	3.051230	0.377359
C	-5.897152	1.558119	-3.099048
H	-3.795261	1.109159	-3.232503
C	-6.918019	2.104145	-2.323085
H	-7.404386	3.066469	-0.453678
H	-6.117850	1.146785	-4.079932
H	-7.938918	2.119460	-2.693237
C	-2.360333	3.482922	-0.512555
C	-2.648357	4.495027	-1.444052
C	-1.553883	3.798620	0.590227
C	-2.154881	5.786100	-1.274693
H	-3.268072	4.267901	-2.305410
C	-1.063975	5.097425	0.760471
H	-1.291295	3.057552	1.337954
C	-1.361603	6.094011	-0.166533
H	-2.394765	6.553019	-2.005695
H	-0.449652	5.319561	1.628073
H	-0.982131	7.102396	-0.028201
C	0.069614	-2.095799	1.892482
C	0.191242	-2.961756	0.669025
C	-1.326170	-2.767110	2.090794
H	-1.396891	-3.423823	2.967591
H	-2.203487	-2.117566	2.040967
N	-1.073085	-3.512900	0.839687
O	1.025727	-3.163091	-0.211807
C	-1.790511	-4.511572	0.182660
C	-3.029526	-4.934111	0.692301
C	-1.276210	-5.116591	-0.980539
C	-3.743416	-5.940786	0.043685
H	-3.428802	-4.468507	1.587179
C	-2.005069	-6.119658	-1.614558
H	-0.311190	-4.797551	-1.355160
C	-3.240603	-6.539184	-1.113283
H	-4.699514	-6.259721	0.450201
H	-1.598543	-6.582023	-2.510230
H	-3.799748	-7.323900	-1.613891
C	1.086739	-2.336243	3.008613
H	2.098056	-2.046323	2.709284
H	0.830742	-1.752566	3.897420
H	1.123766	-3.398547	3.298491
C	3.596184	0.200494	1.501670
C	4.520564	-0.564347	2.227486
C	3.265344	1.480017	1.961510
C	5.117438	-0.053254	3.380085
H	4.772815	-1.565620	1.886000
C	3.851765	1.986776	3.121682
H	2.539612	2.077628	1.420023
C	4.782017	1.225164	3.830596
H	5.831176	-0.658073	3.932034
H	3.574199	2.975341	3.474499
H	5.234522	1.620763	4.735262

H	2.954389	-1.475925	0.381106
H	-2.509895	1.562177	1.315439
C	-3.653211	-0.051478	0.571578
C	-4.422906	-0.037065	1.741475
C	-3.874811	-1.063148	-0.371960
C	-5.408819	-1.001975	1.957295
H	-4.239894	0.727197	2.491924
C	-4.857067	-2.028935	-0.157810
H	-3.278662	-1.104550	-1.278231
C	-5.630298	-1.997560	1.005557
H	-5.995503	-0.977517	2.871050
H	-5.010788	-2.812653	-0.892737
H	-6.395050	-2.751110	1.170891
Br	-0.554247	1.134279	3.437498

^{1}IM4-*S*

B3LYP/BSI SCF energy: -2699.117137a.u.

M06/BSII SCF energy in solution: -2697.91800088a.u.

M06/BSII free energy in solution: -2697.119513a.u.

C	0.285516	1.861331	-0.218872
N	0.788885	0.674869	-0.235470
O	1.150272	2.889884	-0.195500
C	2.484655	2.339946	-0.532113
C	2.267752	0.830435	-0.134267
C	-2.159183	1.223204	-0.101279
N	-1.949814	-0.027793	0.081794
O	-3.440414	1.632929	-0.142905
C	-4.258818	0.498371	0.363752
C	-3.274637	-0.702995	0.107275
Ni	-0.177420	-1.084471	0.139910
C	-1.153557	2.334691	-0.265726
C	-1.383538	3.371384	0.869933
H	-0.705379	4.216033	0.742694
H	-1.210589	2.923678	1.852881
H	-2.414210	3.726739	0.832920
C	-1.382924	3.012332	-1.648161
H	-2.397947	3.411266	-1.698222
H	-1.240413	2.297818	-2.463876
H	-0.670274	3.828532	-1.777735
C	-5.560234	0.440870	-0.417261
C	-6.539795	-0.482358	-0.023365
C	-5.812129	1.270108	-1.514197
C	-7.741352	-0.580566	-0.721128
H	-6.363514	-1.120380	0.837763
C	-7.021658	1.176186	-2.207748
H	-5.065617	1.992419	-1.822699
C	-7.987727	0.250560	-1.816790
H	-8.487810	-1.303620	-0.405634
H	-7.204717	1.830594	-3.055269
H	-8.926597	0.177376	-2.357781
C	-4.488798	0.805373	1.851293
C	-3.731841	0.219893	2.876614
C	-5.439778	1.782471	2.190874
C	-3.937400	0.595002	4.208100
H	-2.975429	-0.530236	2.668213
C	-5.638125	2.157319	3.517351
H	-6.030996	2.246846	1.408185
C	-4.888002	1.560356	4.533837
H	-3.346685	0.122070	4.987159
H	-6.382028	2.911634	3.757563
H	-5.046372	1.845710	5.569956
C	2.642461	2.601090	-2.037399
C	2.620913	1.596934	-3.012600

C	2.744244	3.942695	-2.448901
C	2.711960	1.934222	-4.368688
H	2.538242	0.546832	-2.753688
C	2.825799	4.273034	-3.798077
H	2.763892	4.729302	-1.700723
C	2.812930	3.264602	-4.766726
H	2.702226	1.140032	-5.109497
H	2.905977	5.315295	-4.093907
H	2.883841	3.518191	-5.820739
C	3.537100	3.074508	0.282715
C	3.203638	3.839309	1.405293
C	4.885016	2.959811	-0.085189
C	4.199652	4.481280	2.144111
H	2.165107	3.936186	1.699022
C	5.878889	3.591504	0.660149
H	5.155921	2.381326	-0.963248
C	5.539271	4.357803	1.777063
H	3.923980	5.077358	3.009390
H	6.918629	3.489684	0.363176
H	6.312960	4.855956	2.353999
C	1.016650	-2.494831	-0.601515
C	2.325628	-2.194618	-1.300260
C	1.874137	-3.376059	0.362030
H	1.661638	-4.450871	0.327610
H	1.926964	-3.038528	1.398544
N	3.056199	-2.994175	-0.438728
O	2.695242	-1.543332	-2.274901
C	4.405797	-3.325781	-0.330975
C	4.839034	-4.145141	0.724611
C	5.340395	-2.848272	-1.270148
C	6.187871	-4.477640	0.839256
H	4.119228	-4.513283	1.448695

C	6.684274	-3.190027	-1.136697
H	4.992548	-2.226140	-2.086075
C	7.119512	-4.003175	-0.086452
H	6.510275	-5.113079	1.659686
H	7.398756	-2.818915	-1.866762
H	8.168948	-4.265989	0.006763
C	0.047754	-3.268615	-1.510303
H	-0.802960	-3.656697	-0.945415
H	0.551626	-4.124333	-1.987803
H	-0.336103	-2.632619	-2.315283
H	2.735160	0.188030	-0.875194
C	2.773575	0.419765	1.240819
C	4.033875	-0.183387	1.343118
C	2.029584	0.626237	2.409119
C	4.544416	-0.562954	2.584279
H	4.615944	-0.372450	0.445841
C	2.536982	0.244375	3.651671
H	1.038497	1.064969	2.355585
C	3.796854	-0.348561	3.743377
H	5.518044	-1.040375	2.639683
H	1.940900	0.401597	4.545782
H	4.188105	-0.651837	4.710206
H	-3.286414	-1.391703	0.949454
C	-3.489260	-1.520041	-1.156086
C	-3.924393	-2.845607	-1.040800
C	-3.253405	-0.996202	-2.434271
C	-4.131955	-3.629082	-2.177150
H	-4.083302	-3.271523	-0.054170
C	-3.457349	-1.777713	-3.571046
H	-2.906692	0.026438	-2.547944
C	-3.899519	-3.096657	-3.445460
H	-4.464344	-4.657369	-2.068775

H -3.269719 -1.357167 -4.554847
H -4.054633 -3.706224 -4.330962
Br -1.009975 -2.580568 1.919410

Br⁻

B3LYP/BSI SCF energy: -13.471995a.u.
M06/BSII SCF energy in solution: -13.51096a.u.
M06/BSII free energy in solution: -13.527136a.u.

Br 0.000000 0.000000 0.000000

[1]TS8-*R*

B3LYP/BSI SCF energy: -5894.946536 a.u.
M06/BSII SCF energy in solution: -5893.6337591a.u.
M06/BSII free energy in solution: -5892.619721 a.u.

C -1.112224 2.811616 -0.285036
N -0.451872 1.999445 0.445361
O -0.574242 4.043862 -0.483909
C 0.806815 3.983944 0.036690
C 0.688381 2.763785 1.020733
H 1.572443 2.129120 0.991231
C 0.351575 3.094387 2.476502
C 0.976105 2.386595 3.509578
C -0.635996 4.033873 2.816201
C 0.649532 2.631042 4.846397
H 1.683479 1.602037 3.275044
C -0.961577 4.280245 4.148418
H -1.148625 4.589540 2.039502
C -0.313895 3.583257 5.171907
H 1.142837 2.059860 5.627466
H -1.721177 5.019596 4.387455
H -0.567729 3.776051 6.210620
C -3.107238 1.273617 -0.652386
N -2.572257 0.151272 -0.341175
O -4.443545 1.272435 -0.862545
C -4.983191 -0.032474 -0.490617
C -3.643845 -0.870438 -0.248959
H -3.642059 -1.221334 0.783389
C -3.453129 -2.084595 -1.141175
C -3.861204 -3.338327 -0.664030
C -2.943054 -1.991286 -2.440622
C -3.778785 -4.474613 -1.469133
H -4.248692 -3.430315 0.348069
C -2.850518 -3.129018 -3.244080
H -2.576818 -1.042127 -2.813103
C -3.274107 -4.370868 -2.766900
H -4.103077 -5.436478 -1.082049
H -2.443316 -3.039274 -4.247071
H -3.208224 -5.252007 -3.399184
C -2.508356 2.659735 -0.855874
C -2.535776 3.000338 -2.370447
H -2.134421 4.004686 -2.520689
H -1.932906 2.286692 -2.934628
H -3.567653 2.980332 -2.730509
C -3.395505 3.689182 -0.089663
H -4.410817 3.673658 -0.486140
H -3.432440 3.464083 0.979916
H -2.980422 4.689451 -0.222674
Ni -0.521190 -0.195702 0.111179
Br -0.133821 0.207962 -2.525220
Si 2.728003 -1.293816 0.150368
H 1.196879 -0.710798 0.232841
O 3.826873 -2.007614 1.233504

O	1.953177	-2.881247	-0.110509
C	3.987229	-3.355449	1.615763
H	4.439474	-3.937157	0.794291
H	3.022933	-3.813936	1.853829
C	4.912052	-3.426972	2.827015
H	5.035389	-4.464862	3.157773
H	5.903853	-3.024476	2.589656
H	4.499589	-2.845398	3.657242
C	0.807752	-3.076335	-0.897440
H	-0.011191	-2.405926	-0.582638
H	0.991789	-2.810158	-1.952823
C	0.319566	-4.521350	-0.782107
H	0.141881	-4.784002	0.264601
H	-0.607379	-4.660557	-1.347434
H	1.052425	-5.244742	-1.170779
O	3.224788	0.301455	0.714621
C	3.785042	0.611204	1.971209
H	3.478042	1.636025	2.231017
H	3.408948	-0.052935	2.755258
C	5.317277	0.582458	1.978566
H	5.713119	1.262808	1.211357
H	5.707822	0.914461	2.948775
H	5.655931	-0.442947	1.807249
C	-5.872148	-0.510994	-1.641834
C	-6.810548	-1.531537	-1.437538
C	-5.760256	0.051126	-2.919447
C	-7.608902	-1.985446	-2.486390
H	-6.928760	-1.970539	-0.452796
C	-6.566490	-0.397786	-3.967022
H	-5.048383	0.848118	-3.096630
C	-7.492315	-1.418819	-3.756553
H	-8.328772	-2.778663	-2.306163

H	-6.466937	0.055443	-4.949250
H	-8.119253	-1.767737	-4.571956
C	-5.808874	0.174094	0.788041
C	-6.531367	1.366206	0.946659
C	-5.922701	-0.809629	1.777930
C	-7.324325	1.576059	2.072966
H	-6.467102	2.132312	0.182340
C	-6.724708	-0.603825	2.903644
H	-5.392183	-1.751462	1.684186
C	-7.423858	0.591566	3.058590
H	-7.868066	2.510446	2.179235
H	-6.794011	-1.380356	3.659797
H	-8.041291	0.755165	3.936833
C	1.159873	5.328250	0.665622
C	2.364309	5.450383	1.372587
C	0.355381	6.460754	0.501400
C	2.746038	6.674060	1.917437
H	3.006934	4.583880	1.497364
C	0.741172	7.688542	1.045612
H	-0.575540	6.381130	-0.048348
C	1.935280	7.800423	1.756069
H	3.678777	6.747893	2.469221
H	0.103371	8.557804	0.910706
H	2.234062	8.754970	2.179464
C	1.739208	3.777953	-1.172783
C	1.552739	4.615218	-2.286016
C	2.808226	2.876517	-1.180897
C	2.417250	4.562958	-3.375676
H	0.728585	5.321122	-2.291510
C	3.691349	2.835367	-2.269777
H	2.970459	2.162780	-0.378866
C	3.501714	3.678199	-3.366521

H	2.255890	5.223973	-4.223107
H	4.498797	2.104693	-2.262450
H	4.195373	3.653188	-4.204110
O	3.966961	-2.126694	-3.591994
P	4.612124	-1.479052	-2.352806
O	5.455042	-2.461836	-1.503598
O	5.307732	-0.123922	-2.602960
O	3.262682	-1.091994	-1.431623
K	2.705327	0.269198	-3.798240
K	6.915619	-0.569012	-0.626231
K	3.547696	-4.217233	-1.968129
C	0.204497	-1.468819	2.761578
N	-0.280284	-2.778403	2.751315
C	0.124486	-3.971229	3.341108
C	1.278557	-4.015683	4.147854
C	-0.630398	-5.141809	3.150722
C	1.659408	-5.217564	4.738408
H	1.846171	-3.104959	4.294928
C	-0.231852	-6.336255	3.750609
H	-1.525089	-5.110010	2.536488
C	0.914030	-6.385342	4.546154
H	2.550296	-5.240008	5.360559
H	-0.825955	-7.233028	3.594984
H	1.219283	-7.316616	5.013855
C	-1.825428	0.026503	2.769567
H	-2.746536	0.303237	2.248397
H	-1.278665	0.946333	2.977817
H	-2.117042	-0.411610	3.737869
C	-0.966346	-0.944561	1.953067
C	-1.483544	-2.429909	1.987389
H	-1.559572	-2.957313	1.032322
H	-2.408249	-2.583577	2.563941
O	1.196665	-1.006797	3.303653

[1]**TS8**-*S*

B3LYP/BSI SCF energy: -5894.957302 a.u.

M06/BSII SCF energy in solution: -5893.6433102a.u.

M06/BSII free energy in solution: -5892.631265 a.u.

C	-1.106958	2.794825	-0.383428
N	-0.442982	2.030214	0.388309
O	-0.585511	4.031011	-0.628250
C	0.737493	4.080547	-0.004296
C	0.692840	2.808238	0.939625
H	1.594014	2.199220	0.835158
C	0.439003	3.060591	2.421918
C	1.240297	2.423104	3.374686
C	-0.615548	3.873448	2.865786
C	1.008709	2.606550	4.740845
H	2.029144	1.759776	3.040902
C	-0.847888	4.057867	4.227201
H	-1.254344	4.377224	2.147139
C	-0.033361	3.426284	5.171172
H	1.641189	2.101293	5.465488
H	-1.665510	4.695111	4.552113
H	-0.215493	3.569954	6.232450
C	-3.053743	1.194462	-0.770599
N	-2.481126	0.098962	-0.441422
O	-4.382763	1.125823	-1.024243
C	-4.853071	-0.196033	-0.580501
C	-3.490654	-0.993650	-0.490265
H	-3.418638	-1.534145	0.451712
C	-3.226649	-1.998832	-1.601124
C	-3.184229	-3.360495	-1.273601

C	-3.075332	-1.619513	-2.940693
C	-3.017057	-4.326100	-2.269484
H	-3.263048	-3.657483	-0.232173
C	-2.908054	-2.581956	-3.934591
H	-3.078349	-0.570419	-3.213227
C	-2.884202	-3.939312	-3.603555
H	-2.994081	-5.378677	-2.000016
H	-2.795873	-2.270449	-4.969192
H	-2.764702	-4.688691	-4.381873
C	-2.485295	2.591131	-0.987216
C	-2.453392	2.861534	-2.516473
H	-2.124929	3.887110	-2.699996
H	-1.770041	2.166268	-3.008944
H	-3.457693	2.741024	-2.929917
C	-3.425784	3.622626	-0.297160
H	-4.423373	3.563045	-0.733281
H	-3.504904	3.428708	0.776347
H	-3.034229	4.630623	-0.444805
Ni	-0.535079	-0.211012	0.134902
Br	0.068224	0.043738	-2.454870
Si	2.626356	-1.316403	0.225393
H	1.108225	-0.810506	0.294214
O	3.405746	-2.192435	1.451472
O	1.955347	-2.756060	-0.592789
C	3.774586	-3.563557	1.402049
H	4.188177	-3.811938	0.417579
H	2.899362	-4.201964	1.572876
C	4.808968	-3.835402	2.490855
H	5.092224	-4.894711	2.498425
H	5.716434	-3.237378	2.335078
H	4.411048	-3.579439	3.477581
C	0.628293	-3.212967	-0.641630
H	-0.041515	-2.568750	-0.050910
H	0.242453	-3.140192	-1.673735
C	0.492012	-4.654228	-0.147678
H	0.761768	-4.741142	0.907635
H	-0.541029	-4.996779	-0.267830
H	1.142982	-5.334361	-0.714693
O	2.866593	0.202775	1.106057
C	3.995964	0.518903	1.893133
H	3.952170	1.603235	2.092052
H	3.928453	0.017429	2.872061
C	5.378160	0.214431	1.299500
H	5.467826	0.555761	0.265047
H	6.149862	0.693944	1.917881
H	5.539409	-0.867660	1.319677
C	-5.869709	-0.709399	-1.597733
C	-6.534943	-1.918975	-1.352587
C	-6.177067	0.004885	-2.760131
C	-7.472870	-2.409464	-2.257806
H	-6.322703	-2.479202	-0.447421
C	-7.124666	-0.483876	-3.663104
H	-5.676572	0.944775	-2.959685
C	-7.773686	-1.692564	-3.418433
H	-7.972816	-3.351812	-2.053161
H	-7.351968	0.085960	-4.559863
H	-8.508896	-2.072825	-4.121758
C	-5.550178	0.004922	0.776518
C	-6.387744	1.120472	0.935627
C	-5.448643	-0.914867	1.827849
C	-7.093844	1.320543	2.119139
H	-6.485171	1.831971	0.122491
C	-6.166748	-0.716251	3.011541
H	-4.795421	-1.778606	1.760561

C	-6.988176	0.398438	3.163686
H	-7.732589	2.193237	2.223523
H	-6.068619	-1.437487	3.817667
H	-7.540726	0.550502	4.086466
C	0.895062	5.454166	0.656665
C	1.939050	5.693623	1.561364
C	0.050838	6.515920	0.310430
C	2.124224	6.958390	2.115787
H	2.602235	4.887220	1.855126
C	0.236400	7.783115	0.868069
H	-0.755457	6.351649	-0.394423
C	1.272768	8.010507	1.772097
H	2.933083	7.118875	2.822653
H	-0.432674	8.592532	0.589644
H	1.416891	8.995767	2.205732
C	1.806717	3.982902	-1.107275
C	1.508298	4.355983	-2.424683
C	3.132374	3.649929	-0.789577
C	2.512635	4.419008	-3.394723
H	0.487201	4.604181	-2.688444
C	4.140609	3.723774	-1.754327
H	3.392921	3.329431	0.213341
C	3.836967	4.118283	-3.061660
H	2.259382	4.717661	-4.408295
H	5.159561	3.461282	-1.485325
H	4.622165	4.185781	-3.809566
O	3.994500	-1.403521	-3.503331
P	4.744159	-1.420660	-2.155667
O	5.134815	-2.850408	-1.689582
O	5.894194	-0.404745	-2.035393
O	3.603188	-0.855166	-1.065948
K	3.026047	0.974303	-2.956604
K	7.299264	-1.985462	-0.636581
K	2.829616	-3.625278	-2.907404
C	-1.464740	-2.161900	2.229292
C	0.171381	-1.115726	2.992140
H	0.118025	-0.673083	3.995741
H	1.179274	-1.005315	2.592310
N	-0.344120	-2.500389	2.974131
O	-2.439606	-2.846179	1.915943
C	0.060453	-3.649342	3.650966
C	1.251812	-3.634143	4.396143
C	-0.714948	-4.824070	3.605502
C	1.658007	-4.776819	5.083708
H	1.856957	-2.733784	4.418540
C	-0.290571	-5.955633	4.297951
H	-1.635129	-4.822080	3.034014
C	0.893317	-5.944406	5.041026
H	2.581648	-4.751454	5.655970
H	-0.896760	-6.856987	4.257938
H	1.213869	-6.831675	5.578900
C	-2.001487	0.272558	2.695334
H	-2.950904	0.309842	2.158423
H	-1.583246	1.279760	2.708110
H	-2.234863	-0.002892	3.736124
C	-1.027787	-0.724013	2.063252

[1]**IM5**-*R*

B3LYP/BSI SCF energy: -2686.266634a.u.

M06/BSII SCF energy in solution: -2685.086705a.u.

M06/BSII free energy in solution: -2684.277698a.u.

C	0.590344	-1.977761	-0.052910
N	1.079976	-0.908827	-0.572639

O	1.395540	-2.694989	0.759506
C	2.535438	-1.802262	1.076873
C	2.525837	-0.891680	-0.195297
H	2.811856	0.131553	0.031982
C	3.418746	-1.365685	-1.334127
C	4.600242	-0.657516	-1.592903
C	3.121010	-2.486014	-2.120877
C	5.469409	-1.067909	-2.604545
H	4.813545	0.238716	-1.018965
C	3.986572	-2.894479	-3.135745
H	2.206067	-3.044201	-1.951475
C	5.166267	-2.188469	-3.378735
H	6.377653	-0.503012	-2.794700
H	3.738544	-3.764297	-3.737935
H	5.838922	-2.505405	-4.170730
C	-1.830874	-1.479767	-0.253237
N	-1.657129	-0.241597	-0.542061
O	-3.082109	-1.871325	0.055855
C	-3.974852	-0.745300	-0.318630
C	-2.945755	0.451496	-0.313456
H	-3.142686	1.110139	-1.156501
C	-2.897833	1.316975	0.936106
C	-3.624746	2.514008	0.958962
C	-2.147820	0.964471	2.063864
C	-3.616913	3.333298	2.087225
H	-4.196256	2.810828	0.083178
C	-2.133061	1.785354	3.192249
H	-1.549313	0.059825	2.060070
C	-2.870094	2.969991	3.209224
H	-4.183873	4.259825	2.084880
H	-1.530906	1.500475	4.049840
H	-2.853865	3.611847	4.085382

C	-0.788583	-2.575233	-0.287930
C	-1.114576	-3.650592	0.771346
H	-0.382402	-4.457513	0.716417
H	-1.087144	-3.238360	1.783024
H	-2.108870	-4.059195	0.586073
C	-0.824333	-3.209347	-1.709862
H	-1.827610	-3.591223	-1.917070
H	-0.558864	-2.471440	-2.469854
H	-0.119591	-4.044677	-1.760561
Ni	-0.087373	0.676366	-1.290782
H	-0.999912	1.682811	-1.775993
C	-5.087659	-0.638517	0.709213
C	-6.207914	0.155620	0.424647
C	-5.009754	-1.278827	1.950387
C	-7.224649	0.311143	1.365130
H	-6.289404	0.646231	-0.540747
C	-6.033658	-1.129249	2.888185
H	-4.150535	-1.897705	2.179728
C	-7.141966	-0.333150	2.601301
H	-8.084259	0.931649	1.128943
H	-5.960790	-1.637706	3.845316
H	-7.936602	-0.216872	3.332483
C	-4.492485	-1.103564	-1.719772
C	-5.495325	-2.081298	-1.832916
C	-3.942711	-0.573478	-2.895772
C	-5.947660	-2.503581	-3.080617
H	-5.925272	-2.509821	-0.933495
C	-4.400351	-0.995306	-4.147682
H	-3.146028	0.161536	-2.859517
C	-5.403815	-1.957090	-4.245695
H	-6.727542	-3.257156	-3.142823
H	-3.963946	-0.566659	-5.045145

H	-5.759452	-2.280296	-5.219803
C	3.780637	-2.648584	1.288380
C	4.932633	-2.042408	1.809019
C	3.815951	-4.010633	0.972256
C	6.098238	-2.782056	1.998174
H	4.914132	-0.989818	2.075038
C	4.981759	-4.752942	1.173335
H	2.930534	-4.490071	0.572157
C	6.126765	-4.142519	1.683346
H	6.982962	-2.295644	2.398749
H	4.991306	-5.811213	0.927758
H	7.033258	-4.720663	1.837505
C	2.145773	-1.067496	2.370949
C	1.712793	-1.848162	3.456659
C	2.221378	0.321876	2.526697
C	1.354733	-1.256522	4.664650
H	1.662772	-2.927311	3.348426
C	1.866567	0.912814	3.745329
H	2.547255	0.965951	1.716704
C	1.432604	0.131829	4.814135
H	1.023787	-1.877839	5.492238
H	1.929991	1.992236	3.846920
H	1.162780	0.596221	5.758601
C	2.219393	2.467196	-1.113194
N	1.792030	3.761580	-1.352476
C	2.106670	5.006356	-0.810187
C	3.077068	5.129277	0.202710
C	1.453857	6.156063	-1.285402
C	3.374022	6.386801	0.721383
H	3.579727	4.237555	0.556098
C	1.765062	7.406311	-0.753419
H	0.706580	6.064235	-2.067396
C	2.724949	7.532578	0.252170
H	4.125478	6.471685	1.502186
H	1.251727	8.286934	-1.130427
H	2.965326	8.508521	0.663299
C	1.879723	1.184882	-3.347119
H	1.112306	1.003055	-4.107952
H	2.333743	0.223789	-3.103178
H	2.659918	1.809944	-3.811459
C	1.251530	1.863440	-2.122892
C	0.845491	3.359104	-2.401715
H	-0.186870	3.634395	-2.181074
H	1.135063	3.739608	-3.391426
O	3.090891	2.081473	-0.330141

^{1}IM5-*S*

B3LYP/BSI SCF energy: -2686.268376a.u.

M06/BSII SCF energy in solution: -2685.0886123a.u.

M06/BSII free energy in solution: -2684.278731a.u.

C	2.060087	-1.016004	-0.630596
N	1.823350	0.209966	-0.328978
O	3.278633	-1.500191	-0.322737
C	3.912289	-0.503025	0.574819
C	3.075068	0.789647	0.216308
H	2.839287	1.338894	1.127158
C	3.720652	1.767938	-0.750399
C	4.202336	2.987575	-0.263078
C	3.860989	1.485798	-2.114246
C	4.816446	3.906390	-1.114922
H	4.085253	3.226462	0.791033
C	4.471522	2.402164	-2.969206
H	3.480139	0.553010	-2.517485

C	4.952839	3.615425	-2.472346
H	5.178542	4.850860	-0.719036
H	4.567863	2.170545	-4.026183
H	5.424532	4.330292	-3.140317
C	-0.306061	-1.698543	-0.877723
N	-0.819288	-0.568204	-0.554226
O	-1.112709	-2.778014	-0.847499
C	-2.492998	-2.252781	-0.717425
C	-2.206519	-0.841210	-0.091467
H	-2.864880	-0.088438	-0.513937
C	-2.302285	-0.734711	1.424279
C	-3.318310	0.051055	1.982894
C	-1.412562	-1.394630	2.283139
C	-3.451414	0.165098	3.368328
H	-3.990117	0.592461	1.324038
C	-1.548485	-1.286809	3.666799
H	-0.608389	-2.000767	1.877042
C	-2.569904	-0.506758	4.214756
H	-4.240546	0.786268	3.782261
H	-0.857467	-1.816933	4.316374
H	-2.673061	-0.420779	5.292886
C	1.114951	-1.964530	-1.344353
C	1.511515	-3.431708	-1.074628
H	2.526187	-3.611556	-1.433324
H	1.482826	-3.667785	-0.007933
H	0.825711	-4.100147	-1.596136
C	1.174796	-1.674366	-2.873168
H	0.458383	-2.314807	-3.395073
H	0.929101	-0.630018	-3.078156
H	2.177522	-1.891754	-3.253589
Ni	0.168211	1.214779	-0.641678
H	0.962513	2.421520	-0.678329
C	-3.304530	-3.207131	0.144733
C	-4.679565	-2.976114	0.293257
C	-2.728090	-4.306228	0.788975
C	-5.458240	-3.821226	1.080409
H	-5.142650	-2.136245	-0.216323
C	-3.512957	-5.158669	1.569691
H	-1.667375	-4.497445	0.676442
C	-4.877702	-4.918662	1.721161
H	-6.521150	-3.625454	1.188461
H	-3.051518	-6.011381	2.060001
H	-5.486242	-5.581180	2.329854
C	-3.052106	-2.208463	-2.150352
C	-2.986494	-3.393908	-2.903338
C	-3.623062	-1.070274	-2.732420
C	-3.468366	-3.440340	-4.208011
H	-2.556326	-4.284908	-2.456118
C	-4.113800	-1.124635	-4.043012
H	-3.696126	-0.130475	-2.194119
C	-4.037251	-2.300574	-4.784538
H	-3.407310	-4.366591	-4.772664
H	-4.553832	-0.231991	-4.478237
H	-4.418602	-2.334023	-5.801223
C	5.393783	-0.421491	0.247115
C	6.227536	0.356822	1.063182
C	5.951471	-1.091035	-0.846277
C	7.587695	0.470271	0.784522
H	5.811793	0.868800	1.926201
C	7.317281	-0.982380	-1.120324
H	5.318832	-1.701380	-1.479756
C	8.138941	-0.201046	-0.309651
H	8.218041	1.080014	1.425220
H	7.735848	-1.512797	-1.970904

H	9.200170	-0.116743	-0.524528
C	3.660067	-1.022697	1.999211
C	4.493308	-2.031428	2.510502
C	2.575553	-0.597319	2.780507
C	4.263923	-2.582734	3.769255
H	5.331654	-2.382765	1.918362
C	2.349268	-1.146582	4.045536
H	1.884043	0.155025	2.418488
C	3.192535	-2.136942	4.546245
H	4.925880	-3.357953	4.144401
H	1.507158	-0.792849	4.632475
H	3.017384	-2.559085	5.531620
C	-2.653648	2.475952	-0.756725
C	-1.173016	3.919316	-0.526415
H	-1.062495	4.775294	-1.206041
H	-0.485920	4.020516	0.316248
N	-2.570086	3.700352	-0.117483
O	-3.636582	1.743543	-0.895845
C	-3.462224	4.482068	0.614597
C	-3.021575	5.687233	1.186536
C	-4.802248	4.084049	0.783664
C	-3.905164	6.475614	1.921765
H	-1.990150	5.998386	1.053342
C	-5.669561	4.884510	1.522790
H	-5.136221	3.161852	0.324303
C	-5.232734	6.081378	2.097794
H	-3.550256	7.404976	2.359294
H	-6.702521	4.569616	1.646567
H	-5.918036	6.698734	2.670908
C	-1.114761	2.563390	-2.757577
H	-1.410648	1.605826	-3.200009
H	-0.092727	2.779698	-3.081852
H	-1.774433	3.335737	-3.187141
C	-1.207909	2.511621	-1.221258

[1]**TS9**-*R*

B3LYP/BSI SCF energy: -2922.092849a.u.

M06/BSII SCF energy in solution: -2920.785776a.u.

M06/BSII free energy in solution: -2919.820852a.u.

C	1.479386	-1.786115	-0.466884
N	1.577009	-0.568054	-0.073908
O	2.562103	-2.568515	-0.268137
C	3.548358	-1.818660	0.506962
C	2.951384	-0.346436	0.439043
C	-0.992913	-1.926249	-0.845134
N	-1.375254	-1.809811	0.358619
O	-1.886245	-1.687480	-1.842985
C	-3.156943	-1.310567	-1.210572
C	-2.763879	-1.322627	0.344842
Ni	0.386742	0.999010	-0.239875
C	0.368944	-2.449652	-1.271236
C	0.660483	-2.161077	-2.765186
H	1.636018	-2.575389	-3.036274
H	0.655396	-1.086740	-2.961400
H	-0.101522	-2.632131	-3.389309
C	0.359074	-3.979879	-1.024107
H	-0.461168	-4.428053	-1.591943
H	0.206196	-4.199801	0.034424
H	1.295538	-4.434677	-1.354069
C	-4.216298	-2.330939	-1.642396
C	-5.562629	-2.121704	-1.308862
C	-3.880304	-3.473095	-2.377754
C	-6.541714	-3.036836	-1.687553

H	-5.847756	-1.238250	-0.747538
C	-4.863488	-4.388892	-2.762359
H	-2.848317	-3.640116	-2.662405
C	-6.196899	-4.176898	-2.417580
H	-7.577325	-2.856998	-1.413421
H	-4.581303	-5.267275	-3.336627
H	-6.961295	-4.888459	-2.716455
C	-3.534114	0.074982	-1.744035
C	-4.183215	1.028833	-0.951751
C	-3.286244	0.376169	-3.092363
C	-4.574384	2.256356	-1.494519
H	-4.371026	0.838781	0.098501
C	-3.666825	1.604314	-3.630127
H	-2.787699	-0.357016	-3.716993
C	-4.316625	2.550426	-2.832518
H	-5.070761	2.984855	-0.860127
H	-3.461151	1.820430	-4.674973
H	-4.616417	3.506985	-3.250959
C	3.552679	-2.394298	1.931483
C	3.952845	-1.628318	3.034147
C	3.220817	-3.740290	2.136070
C	4.009235	-2.189741	4.311189
H	4.221381	-0.583040	2.913384
C	3.270426	-4.298917	3.412585
H	2.915171	-4.348133	1.292501
C	3.664970	-3.526626	4.506048
H	4.315194	-1.575371	5.152852
H	2.998645	-5.341328	3.551432
H	3.700863	-3.961715	5.500308
C	4.916343	-2.026459	-0.149465
C	5.056862	-2.793092	-1.311650
C	6.064087	-1.463008	0.425788
C	6.314008	-2.983089	-1.890761
H	4.183546	-3.248201	-1.762482
C	7.316129	-1.646523	-0.156526
H	5.985869	-0.878731	1.336124
C	7.447737	-2.409001	-1.319124
H	6.401604	-3.584870	-2.790995
H	8.191120	-1.195853	0.302769
H	8.424706	-2.556008	-1.770096
C	0.078338	1.250879	1.824165
C	-1.275078	1.848291	2.124461
N	-0.732182	3.037611	2.621621
C	-1.263541	4.236698	3.094955
C	-2.657608	4.421698	3.172556
C	-0.405202	5.271056	3.504025
C	-3.165538	5.627492	3.648142
H	-3.309475	3.613419	2.863813
C	-0.933493	6.471529	3.977031
H	0.670060	5.129160	3.452428
C	-2.314251	6.660370	4.052411
H	-4.242802	5.760379	3.705342
H	-0.257430	7.262950	4.289802
H	-2.721771	7.596289	4.422658
C	0.318424	0.004035	2.687555
H	1.359740	-0.328752	2.674671
H	-0.290614	-0.830220	2.333834
H	0.064960	0.185813	3.744896
C	-0.701292	2.714547	-0.697869
H	-0.934141	3.204996	0.235811
H	-1.548274	2.419308	-1.312619
C	0.552103	2.845317	-1.269402
H	0.450038	0.664816	-1.663050
H	1.336188	3.309786	-0.666557

C	0.827565	2.862526	-2.755485
H	0.029181	2.328645	-3.282275
H	1.768355	2.345515	-2.971911
C	0.919348	4.310956	-3.277341
H	-0.025099	4.832146	-3.070529
H	1.697532	4.852522	-2.720656
C	1.228887	4.385188	-4.778185
H	0.455519	3.837524	-5.333713
H	2.174240	3.862102	-4.977750
C	1.317373	5.820893	-5.305367
H	0.374904	6.358298	-5.150446
H	1.539256	5.841166	-6.377555
H	2.105276	6.383764	-4.791745
C	-3.640819	-2.106094	1.304773
C	-3.696837	-3.506349	1.287448
C	-4.383515	-1.413585	2.269142
C	-4.499758	-4.196470	2.193568
H	-3.109814	-4.058468	0.560354
C	-5.192287	-2.103601	3.175617
H	-4.294892	-0.332485	2.330765
C	-5.256848	-3.496228	3.136809
H	-4.535019	-5.282192	2.165012
H	-5.760185	-1.551292	3.919336
H	-5.883271	-4.035073	3.842480
C	3.710433	0.673021	-0.392723
C	4.305366	1.767737	0.245028
C	3.850615	0.545007	-1.781111
C	5.033265	2.712968	-0.481964
H	4.196109	1.885213	1.320712
C	4.579718	1.483469	-2.509203
H	3.388109	-0.290520	-2.295971
C	5.174532	2.570467	-1.862016
H	5.486820	3.556439	0.030592
H	4.684985	1.366747	-3.584024
H	5.742202	3.300444	-2.431697
H	2.864173	0.051389	1.448670
H	-2.729967	-0.292639	0.708917
C	0.652068	2.577614	2.439237
H	1.190338	2.462248	3.390485
H	1.236337	3.223725	1.771382
O	-2.451910	1.517782	2.025218

[1]**TS9**-*S*

B3LYP/BSI SCF energy: -2922.09832a.u.

M06/BSII SCF energy in solution: -2920.789465a.u.

M06/BSII free energy in solution: -2919.826655a.u.

C	-0.743477	-2.040961	-0.547013
N	-1.116596	-0.890960	-0.134789
O	-1.727644	-2.928212	-0.842552
C	-3.002185	-2.190089	-0.840207
C	-2.600079	-0.885215	-0.046218
C	1.769227	-1.636338	-0.460059
N	1.727476	-0.364145	-0.441957
O	2.983709	-2.227355	-0.275304
C	3.925193	-1.181038	0.121600
C	3.094438	0.137160	-0.220953
Ni	-0.099163	0.774629	0.380189
C	0.648787	-2.623126	-0.739448
C	0.783382	-3.847268	0.206538
H	-0.020842	-4.557128	0.006079
H	0.731175	-3.541540	1.255529
H	1.740527	-4.343517	0.039710
C	0.793533	-3.088602	-2.216929

H	1.786408	-3.518324	-2.371910
H	0.659073	-2.248567	-2.903134
H	0.042095	-3.846573	-2.444796
C	5.223163	-1.387772	-0.661010
C	6.343132	-0.590138	-0.386387
C	5.327783	-2.370308	-1.652035
C	7.531044	-0.762255	-1.093691
H	6.294156	0.168390	0.387707
C	6.521324	-2.547114	-2.355945
H	4.476301	-3.003698	-1.868643
C	7.626679	-1.743527	-2.082682
H	8.384930	-0.130114	-0.867716
H	6.582497	-3.318500	-3.118575
H	8.554317	-1.880592	-2.630618
C	4.176761	-1.339896	1.629922
C	4.513454	-0.252127	2.446154
C	4.137428	-2.617862	2.205737
C	4.791415	-0.433547	3.803015
H	4.565306	0.751535	2.035820
C	4.408379	-2.798921	3.561242
H	3.888701	-3.471923	1.586371
C	4.736157	-1.707085	4.367212
H	5.047031	0.425751	4.416205
H	4.365623	-3.797247	3.987478
H	4.947095	-1.848169	5.423117
C	-3.375784	-1.945016	-2.313031
C	-3.886252	-0.731577	-2.789668
C	-3.260148	-3.024740	-3.204522
C	-4.264650	-0.604957	-4.130965
H	-3.971127	0.140242	-2.149667
C	-3.629356	-2.892367	-4.540048
H	-2.881225	-3.975101	-2.842414

C	-4.137568	-1.677940	-5.009488
H	-4.651198	0.347132	-4.482737
H	-3.527525	-3.739053	-5.213275
H	-4.429983	-1.572382	-6.050322
C	-4.069032	-3.065452	-0.188551
C	-3.775135	-4.324333	0.344524
C	-5.392102	-2.603306	-0.142258
C	-4.782987	-5.103031	0.919689
H	-2.758855	-4.698498	0.307737
C	-6.393751	-3.376247	0.439751
H	-5.641081	-1.636562	-0.569024
C	-6.093576	-4.632164	0.973187
H	-4.537640	-6.080619	1.325539
H	-7.411742	-2.998819	0.471578
H	-6.875913	-5.237377	1.421925
C	-0.588314	1.770863	-1.420950
C	-1.897344	2.458391	-1.195383
C	-0.017067	3.205093	-1.673829
H	0.204467	3.421132	-2.728022
H	0.818112	3.549961	-1.057842
N	-1.332027	3.735644	-1.269104
O	-3.074831	2.137823	-1.009390
C	-1.826630	5.032508	-1.159258
C	-0.978590	6.129145	-1.391281
C	-3.174760	5.260636	-0.819998
C	-1.470253	7.428864	-1.280205
H	0.059593	5.956950	-1.658564
C	-3.646684	6.566130	-0.714669
H	-3.823070	4.408421	-0.655693
C	-2.804582	7.659081	-0.940942
H	-0.802157	8.266686	-1.462169
H	-4.688903	6.730911	-0.453423

H	-3.183579	8.673147	-0.855959
C	-0.499871	0.855609	-2.640991
H	-1.165266	-0.005444	-2.560253
H	0.520192	0.479069	-2.767113
H	-0.773121	1.379571	-3.571368
C	0.412652	2.463336	1.336855
H	0.009844	3.297597	0.778745
H	1.479018	2.516458	1.562058
C	-0.425480	1.741180	2.200752
H	0.212407	0.013259	1.575976
H	-1.497357	1.930400	2.128377
C	0.006176	1.233149	3.564616
H	1.071469	0.970820	3.542902
H	-0.544939	0.320914	3.819468
C	-0.241761	2.290267	4.657218
H	0.312121	3.206211	4.409365
H	-1.305481	2.566963	4.659630
C	0.159377	1.811089	6.058424
H	1.220057	1.524031	6.052560
H	-0.400123	0.897322	6.300800
C	-0.079471	2.861582	7.147853
H	0.494605	3.774240	6.950683
H	0.214672	2.490477	8.135487
H	-1.137183	3.143995	7.200058
C	3.572471	0.989127	-1.383072
C	3.495116	0.542048	-2.708501
C	4.098572	2.261408	-1.133409
C	3.939494	1.346836	-3.756258
H	3.081889	-0.438302	-2.923649
C	4.545709	3.070676	-2.179960
H	4.151530	2.627993	-0.110788
C	4.467963	2.613546	-3.495303
H	3.871028	0.986199	-4.778574
H	4.947275	4.057179	-1.966570
H	4.811323	3.240515	-4.313059
C	-3.065600	-0.779457	1.397428
C	-3.932761	0.262097	1.749939
C	-2.653225	-1.682190	2.386532
C	-4.391425	0.390234	3.063572
H	-4.221845	0.987252	0.994417
C	-3.110297	-1.555546	3.697553
H	-1.973683	-2.490306	2.133501
C	-3.984238	-0.519505	4.039568
H	-5.061884	1.204983	3.322498
H	-2.786807	-2.266635	4.452682
H	-4.340765	-0.422300	5.061222
H	-2.943948	0.010776	-0.560765
H	3.060888	0.775165	0.662461

[1]IM6-*R*

B3LYP/BSI SCF energy: -2922.147394a.u.

M06/BSII SCF energy in solution: -2920.837461a.u.

M06/BSII free energy in solution: -2919.86781a.u.

C	0.502206	-1.072607	-1.474057
N	1.005010	-0.714036	-0.345912
O	1.134558	-2.059073	-2.146361
C	2.064335	-2.677763	-1.188179
C	2.268185	-1.475755	-0.184182
C	-1.766582	-0.267170	-1.079121
N	-1.561464	0.337564	0.028219
O	-2.988790	-0.764565	-1.318441
C	-3.835829	-0.491660	-0.132223
C	-2.834926	0.368020	0.781457

Ni	0.277238	0.878718	0.783925
C	-0.718584	-0.473077	-2.159444
C	-1.240773	-1.394191	-3.278367
H	-0.475808	-1.513409	-4.047963
H	-1.506496	-2.384418	-2.903099
H	-2.130208	-0.951501	-3.730154
C	-0.333233	0.909502	-2.758213
H	-1.212593	1.370162	-3.216441
H	0.046790	1.581611	-1.986800
H	0.429094	0.779627	-3.532638
C	-5.045528	0.285066	-0.670420
C	-6.204143	-0.403071	-1.065245
C	-4.984377	1.669919	-0.892301
C	-7.283240	0.273689	-1.632809
H	-6.266596	-1.476262	-0.927244
C	-6.068128	2.344722	-1.458166
H	-4.096334	2.241130	-0.644948
C	-7.222806	1.654282	-1.825081
H	-8.170358	-0.281535	-1.924444
H	-6.000923	3.417984	-1.611663
H	-8.064557	2.184635	-2.261606
C	-4.209817	-1.836497	0.488670
C	-5.165857	-1.883173	1.515669
C	-3.594744	-3.029104	0.094743
C	-5.489830	-3.088165	2.134174
H	-5.658115	-0.970029	1.832933
C	-3.919178	-4.238531	0.715672
H	-2.863094	-3.020490	-0.703878
C	-4.865937	-4.273632	1.737229
H	-6.230402	-3.100225	2.928638
H	-3.422617	-5.150230	0.396026
H	-5.118461	-5.213205	2.220301
C	3.301628	-3.139406	-1.949595
C	4.165960	-4.083562	-1.379210
C	3.608970	-2.620310	-3.213109
C	5.317372	-4.490428	-2.052347
H	3.934724	-4.514449	-0.410456
C	4.755965	-3.036491	-3.890108
H	2.945965	-1.895390	-3.669264
C	5.616260	-3.970053	-3.312298
H	5.975596	-5.222572	-1.593567
H	4.975080	-2.627430	-4.872222
H	6.508901	-4.292762	-3.840101
C	1.314679	-3.868364	-0.565283
C	0.487941	-4.644564	-1.392935
C	1.465469	-4.251161	0.773049
C	-0.183084	-5.757952	-0.891105
H	0.374687	-4.370407	-2.436030
C	0.795606	-5.370643	1.276011
H	2.109319	-3.690835	1.442960
C	-0.033558	-6.125185	0.448661
H	-0.817280	-6.344127	-1.550150
H	0.924953	-5.646301	2.318460
H	-0.553584	-6.994307	0.840560
C	-0.136975	2.778900	1.192598
C	-1.111534	3.353963	0.180137
C	0.916202	3.721037	0.498958
H	1.245076	4.579448	1.100564
H	1.783587	3.238334	0.042058
N	-0.128768	4.092723	-0.468953
O	-2.317309	3.305474	-0.044216
C	-0.120488	4.964888	-1.555039
C	1.074102	5.606045	-1.925832
C	-1.295831	5.211952	-2.290985

C 1.091630 6.474011 -3.016535
H 1.979955 5.424489 -1.355642
C -1.257647 6.082111 -3.377329
H -2.214621 4.725398 -1.987394
C -0.070066 6.718070 -3.751123
H 2.022348 6.963743 -3.290594
H -2.170715 6.268149 -3.936947
H -0.052175 7.396268 -4.598956
C -0.496629 3.134924 2.635476
H -1.403652 2.611082 2.950260
H 0.299374 2.852100 3.328969
H -0.677870 4.214528 2.761114
C 1.715886 0.867634 2.085636
H 2.152545 -0.140270 2.016231
H 1.136731 0.878491 3.023246
C 2.863352 1.872093 2.193196
H 3.418732 1.915764 1.246626
H 2.469664 2.881820 2.364465
C 3.862964 1.561145 3.323417
H 3.325431 1.524465 4.282440
H 4.280535 0.555359 3.170228
C 5.010918 2.573956 3.421089
H 4.594468 3.581417 3.567439
H 5.546042 2.605090 2.460731
C 6.011213 2.276729 4.545968
H 5.476978 2.242397 5.505492
H 6.432383 1.272174 4.399652
C 7.149083 3.299175 4.632945
H 6.760778 4.307999 4.814793
H 7.845696 3.060256 5.443845
H 7.723875 3.332854 3.699941
C -2.680970 -0.068915 2.224758
C -3.428098 0.586522 3.211657
C -1.817564 -1.102934 2.608238
C -3.328990 0.209774 4.552317
H -4.088108 1.402597 2.927420
C -1.711208 -1.475705 3.948098
H -1.224219 -1.615320 1.858226
C -2.467984 -0.823254 4.924127
H -3.914708 0.730997 5.304249
H -1.034628 -2.277466 4.230348
H -2.381899 -1.114428 5.967153
H -3.155018 1.409729 0.790538
H 2.322346 -1.845746 0.837697
C 3.499218 -0.620051 -0.439850
C 4.673875 -0.890609 0.272573
C 3.511150 0.408872 -1.388191
C 5.838052 -0.159234 0.037398
H 4.678234 -1.678144 1.021954
C 4.673513 1.144531 -1.624582
H 2.606494 0.653035 -1.934022
C 5.841414 0.861663 -0.914650
H 6.738531 -0.382901 0.601927
H 4.663354 1.942453 -2.361676
H 6.744904 1.436176 -1.096868

[1]**IM6**-*R*

B3LYP-D3BJ/BSI SCF energy in solution:

-2922.572805a.u.

M06/BSII SCF energy in solution: -2920.846321a.u.

M06/BSII free energy in solution: -2919.86801a.u.

C 0.456529 -0.691879 -1.555061
N 0.931264 -0.475927 -0.378004

O	1.092074	-1.605733	-2.310859
C	1.957547	-2.354572	-1.379639
C	2.175002	-1.266801	-0.271625
C	-1.783348	-0.006051	-1.056244
N	-1.592014	0.548538	0.079903
O	-2.910487	-0.704718	-1.235824
C	-3.712017	-0.589529	0.009564
C	-2.718419	0.206814	0.967030
Ni	0.222965	0.941780	0.908881
C	-0.753720	-0.004122	-2.165641
C	-1.257120	-0.742797	-3.412687
H	-0.485172	-0.735859	-4.184295
H	-1.519532	-1.779122	-3.193305
H	-2.142708	-0.236266	-3.802315
C	-0.376802	1.457572	-2.514785
H	-1.258080	1.985944	-2.886430
H	0.000143	1.981773	-1.636119
H	0.389518	1.463969	-3.294593
C	-4.949870	0.205922	-0.392402
C	-6.144549	-0.446314	-0.723764
C	-4.874050	1.598056	-0.544117
C	-7.249124	0.278012	-1.173631
H	-6.216340	-1.523544	-0.629480
C	-5.978906	2.319730	-0.996020
H	-3.962244	2.133402	-0.302961
C	-7.171574	1.664709	-1.308430
H	-8.168969	-0.244809	-1.418960
H	-5.905756	3.398655	-1.099102
H	-8.032153	2.229716	-1.654643
C	-4.016574	-1.984483	0.514549
C	-4.822295	-2.122350	1.654093
C	-3.463239	-3.124737	-0.069938
C	-5.060011	-3.378928	2.203529
H	-5.254518	-1.240583	2.115809
C	-3.708297	-4.386412	0.478608
H	-2.828982	-3.031705	-0.942785
C	-4.501637	-4.518328	1.616658
H	-5.678445	-3.468891	3.091565
H	-3.268330	-5.262914	0.014911
H	-4.684906	-5.498958	2.045790
C	3.213146	-2.778311	-2.110504
C	4.001129	-3.814653	-1.597665
C	3.646168	-2.087848	-3.247880
C	5.211152	-4.147285	-2.206638
H	3.672075	-4.361509	-0.720767
C	4.851269	-2.428805	-3.861242
H	3.041926	-1.283557	-3.648593
C	5.640242	-3.455932	-3.340726
H	5.815201	-4.950664	-1.795697
H	5.174399	-1.886647	-4.744931
H	6.580457	-3.717264	-3.816983
C	1.125704	-3.537788	-0.882484
C	0.238413	-4.159779	-1.772241
C	1.247842	-4.045817	0.415387
C	-0.521494	-5.255406	-1.366432
H	0.143593	-3.780798	-2.783366
C	0.487334	-5.145671	0.821033
H	1.938259	-3.599356	1.121947
C	-0.400969	-5.751538	-0.065930
H	-1.206185	-5.722654	-2.067915
H	0.592953	-5.524584	1.832872
H	-0.991780	-6.605204	0.250987
C	-0.216266	2.750438	1.616798
C	-1.375399	3.326387	0.847472

C	0.670909	3.730236	0.762595
H	1.073819	4.596040	1.303789
H	1.454384	3.280088	0.148020
N	-0.547933	4.069796	0.009007
O	-2.602573	3.292935	0.897745
C	-0.739417	4.765296	-1.175190
C	0.362183	5.333030	-1.837664
C	-2.024788	4.882093	-1.739392
C	0.178510	5.997938	-3.049137
H	1.352489	5.249123	-1.401611
C	-2.187632	5.548109	-2.951293
H	-2.868938	4.452026	-1.214868
C	-1.092910	6.109489	-3.616543
H	1.038570	6.432230	-3.551435
H	-3.183053	5.630602	-3.379284
H	-1.230034	6.628450	-4.560171
C	-0.293402	2.986410	3.118326
H	-1.067340	2.354158	3.566022
H	0.647803	2.747277	3.618152
H	-0.538041	4.032917	3.360771
C	1.719661	0.819460	2.125146
H	2.134553	-0.188024	1.984545
H	1.254893	0.811890	3.122866
C	2.875659	1.817915	2.106504
H	3.279439	1.902832	1.090952
H	2.517916	2.819412	2.376515
C	4.031419	1.445705	3.047061
H	3.669602	1.430662	4.085843
H	4.354857	0.420536	2.820243
C	5.237731	2.381894	2.933870
H	4.936409	3.405242	3.201026
H	5.557757	2.421701	1.883367

C	6.426979	1.959840	3.801422
H	6.118968	1.934799	4.855721
H	6.710159	0.930152	3.542247
C	7.641762	2.876359	3.643575
H	7.396427	3.909273	3.917884
H	8.477084	2.554528	4.275500
H	7.995062	2.887486	2.605431
C	-2.202949	-0.533943	2.180967
C	-2.611176	-0.136353	3.455985
C	-1.313487	-1.610433	2.052456
C	-2.142487	-0.801797	4.591723
H	-3.296504	0.700501	3.557792
C	-0.848620	-2.278007	3.182381
H	-0.987041	-1.931078	1.070301
C	-1.260131	-1.874010	4.456600
H	-2.465506	-0.480805	5.577732
H	-0.168226	-3.116086	3.066843
H	-0.895185	-2.394345	5.337415
H	-3.184282	1.132248	1.300353
H	2.228119	-1.733516	0.707547
C	3.404503	-0.408007	-0.469500
C	4.586539	-0.757361	0.190429
C	3.405091	0.696748	-1.327011
C	5.753334	-0.018676	-0.003465
H	4.590397	-1.611395	0.861618
C	4.571894	1.435671	-1.526221
H	2.493173	0.995112	-1.831174
C	5.749412	1.080601	-0.865120
H	6.662062	-0.297086	0.521764
H	4.558535	2.291645	-2.194661
H	6.656575	1.658007	-1.016199

[1]IM6-*S*

B3LYP/BSI SCF energy: -2922.144112a.u.

M06/BSII SCF energy in solution: -2920.8374738a.u.

M06/BSII free energy in solution: -2919.868554a.u.

C	-0.633678	-1.638290	-1.091686
N	-0.973131	-0.509027	-0.602226
O	-1.412354	-2.702480	-0.832777
C	-2.531721	-2.257671	0.013357
C	-2.247352	-0.685399	0.126120
C	1.777495	-1.288347	-1.169442
N	1.809624	-0.149849	-0.573974
O	2.905347	-2.032259	-1.143476
C	3.772836	-1.426367	-0.120108
C	3.207270	0.049514	-0.116902
Ni	0.189809	1.200903	-0.591009
C	0.602951	-1.881174	-1.937745
C	0.813524	-3.383057	-2.207941
H	-0.051209	-3.780927	-2.742025
H	0.939452	-3.954438	-1.286213
H	1.702745	-3.526202	-2.824659
C	0.444356	-1.126048	-3.288114
H	1.351884	-1.251926	-3.887024
H	0.248253	-0.065151	-3.127938
H	-0.400278	-1.542424	-3.842583
C	5.221971	-1.594870	-0.562043
C	6.260172	-1.511883	0.375479
C	5.545229	-1.809339	-1.907832
C	7.591043	-1.626985	-0.025564
H	6.032204	-1.369665	1.426732
C	6.876440	-1.934991	-2.305859
H	4.753547	-1.885128	-2.643156
C	7.904956	-1.840795	-1.368140
H	8.381608	-1.558547	0.716145
H	7.107226	-2.106464	-3.353328
H	8.940902	-1.937794	-1.679554
C	3.492028	-2.183394	1.189287
C	3.500615	-1.564927	2.445564
C	3.278303	-3.569632	1.129517
C	3.284805	-2.308161	3.610029
H	3.680615	-0.499070	2.539298
C	3.059515	-4.310479	2.289601
H	3.286329	-4.066067	0.165345
C	3.059427	-3.681340	3.537132
H	3.293026	-1.805903	4.572942
H	2.897629	-5.382379	2.218850
H	2.892041	-4.257514	4.442327
C	-3.817765	-2.612309	-0.735626
C	-4.961600	-1.809018	-0.688421
C	-3.875701	-3.822647	-1.443625
C	-6.135055	-2.200206	-1.339125
H	-4.957655	-0.865744	-0.154137
C	-5.042470	-4.208512	-2.099208
H	-2.998305	-4.459044	-1.483975
C	-6.179778	-3.398600	-2.048058
H	-7.005338	-1.552562	-1.295056
H	-5.063946	-5.144635	-2.650036
H	-7.089685	-3.699499	-2.559159
C	-2.441077	-3.026979	1.335446
C	-1.256515	-3.658798	1.732209
C	-3.550633	-3.084333	2.190427
C	-1.180418	-4.325962	2.956530
H	-0.388107	-3.638335	1.084886
C	-3.472395	-3.745495	3.414763

H	-4.483433	-2.614969	1.897122
C	-2.286302	-4.370825	3.803933
H	-0.249108	-4.805828	3.243365
H	-4.343564	-3.776019	4.062823
H	-2.227814	-4.889908	4.756308
C	-1.090549	2.426992	-1.463602
C	-2.272849	1.700656	-2.082163
C	-2.168434	3.200050	-0.625138
H	-2.266016	4.269384	-0.855714
H	-2.139355	3.056687	0.457777
N	-3.200603	2.386551	-1.292378
O	-2.456231	0.850830	-2.941640
C	-4.590636	2.409807	-1.227431
C	-5.238149	3.244820	-0.298884
C	-5.363065	1.603407	-2.088012
C	-6.630845	3.270706	-0.233751
H	-4.646803	3.870816	0.362043
C	-6.753168	1.645418	-2.007455
H	-4.855703	0.969135	-2.804936
C	-7.399252	2.473207	-1.084046
H	-7.116350	3.923270	0.487095
H	-7.338288	1.027229	-2.683603
H	-8.483541	2.500053	-1.032345
C	-0.377436	3.287284	-2.519260
H	0.128043	2.653741	-3.255800
H	0.377878	3.937280	-2.072796
H	-1.086314	3.925129	-3.072439
C	1.264677	2.660850	0.083243
H	1.900665	2.138900	0.818975
H	1.937086	3.026450	-0.704630
C	0.622926	3.844970	0.810420
H	-0.078652	3.482957	1.575591
H	0.029168	4.450877	0.114503
C	1.643089	4.776182	1.492890
H	2.357303	5.141017	0.740241
H	2.236443	4.196853	2.216162
C	1.003594	5.972941	2.209173
H	0.414995	6.555581	1.485355
H	0.284184	5.606597	2.956489
C	2.013936	6.900316	2.896721
H	2.734645	7.265436	2.152041
H	2.599389	6.321442	3.624695
C	1.360737	8.093690	3.601790
H	0.799455	8.713133	2.892635
H	2.105842	8.734733	4.085343
H	0.657395	7.760599	4.374039
H	-3.012654	-0.163191	-0.448372
C	-2.205563	-0.086426	1.519691
C	-3.298224	0.651620	1.989649
C	-1.091439	-0.246952	2.355047
C	-3.289432	1.202592	3.273304
H	-4.156189	0.819828	1.344446
C	-1.079622	0.304903	3.635197
H	-0.229162	-0.802316	2.002214
C	-2.180382	1.028482	4.100808
H	-4.146142	1.774043	3.619020
H	-0.208540	0.169525	4.270020
H	-2.169406	1.458189	5.098474
H	3.187513	0.441517	0.898327
C	3.973180	1.037853	-0.983382
C	4.982682	1.809551	-0.395299
C	3.722919	1.188001	-2.351542
C	5.734339	2.703636	-1.156127
H	5.179004	1.715363	0.669789

C 4.470652 2.085777 -3.115428

H 2.923397 0.625962 -2.821426

C 5.480107 2.844183 -2.521624

H 6.510490 3.296523 -0.680890

H 4.257103 2.196888 -4.174721

H 6.058491 3.545582 -3.115921

^{1}**IM6**-*R*

B3LYP-D3BJ/BSI SCF energy in solution:

-2922.572248a.u.

M06/BSII SCF energy in solution: -2920.845968a.u.

M06/BSII free energy in solution: -2919.868636a.u.

C -0.709961 -1.587705 -1.199136

N -1.025380 -0.403735 -0.840136

O -1.465520 -2.600210 -0.745405

C -2.471747 -2.039612 0.166442

C -2.175883 -0.478884 0.075655

C 1.683953 -1.307051 -1.241532

N 1.700603 -0.146150 -0.684339

O 2.809498 -2.038486 -1.157995

C 3.632130 -1.386532 -0.116462

C 3.079389 0.076759 -0.203882

Ni 0.203701 1.219885 -0.874458

C 0.523669 -1.924358 -2.008218

C 0.704604 -3.441800 -2.161636

H -0.162890 -3.862064 -2.674417

H 0.815021 -3.941855 -1.197358

H 1.593432 -3.647840 -2.760444

C 0.436676 -1.249984 -3.400555

H 1.382697 -1.386413 -3.932076

H 0.212288 -0.186438 -3.309355

H -0.361088 -1.722001 -3.979302

C 5.092290 -1.536400 -0.481860

C 6.069671 -1.450587 0.515628

C 5.486811 -1.665045 -1.818391

C 7.423514 -1.477472 0.179869

H 5.774445 -1.358341 1.555410

C 6.840213 -1.700062 -2.151106

H 4.735405 -1.731426 -2.595591

C 7.813111 -1.601011 -1.154489

H 8.172246 -1.406065 0.963210

H 7.133582 -1.800647 -3.191851

H 8.866870 -1.623858 -1.415846

C 3.278924 -2.092377 1.193421

C 3.051410 -1.416738 2.396346

C 3.225180 -3.494539 1.183642

C 2.772261 -2.128740 3.566915

H 3.096942 -0.335333 2.447093

C 2.946869 -4.203456 2.349418

H 3.407007 -4.027332 0.256463

C 2.721943 -3.520758 3.548535

H 2.596850 -1.588274 4.492004

H 2.910996 -5.288494 2.324017

H 2.509370 -4.071674 4.459604

C -3.845954 -2.422878 -0.358108

C -4.970559 -1.642857 -0.067343

C -4.010187 -3.616441 -1.072161

C -6.240131 -2.053221 -0.477720

H -4.867859 -0.710343 0.476563

C -5.276438 -4.014994 -1.497892

H -3.144383 -4.228316 -1.298024

C -6.397175 -3.237271 -1.197238

H -7.100010 -1.433711 -0.246590

H	-5.387953	-4.937387	-2.060304
H	-7.384006	-3.551066	-1.524345
C	-2.217552	-2.636236	1.547899
C	-1.040447	-3.334913	1.831829
C	-3.149233	-2.431565	2.573995
C	-0.802303	-3.823663	3.118373
H	-0.305234	-3.499891	1.054088
C	-2.902798	-2.905152	3.860264
H	-4.068044	-1.893358	2.373143
C	-1.727359	-3.606738	4.138049
H	0.113712	-4.368471	3.317724
H	-3.632758	-2.728857	4.644772
H	-1.537224	-3.981356	5.139491
C	-1.044335	2.463315	-1.767964
C	-2.097863	1.675683	-2.517823
C	-2.235731	3.021600	-0.917386
H	-2.472116	4.080815	-1.082531
H	-2.221210	2.806784	0.151415
N	-3.123355	2.128710	-1.684225
O	-2.150292	0.944234	-3.497446
C	-4.485646	1.882216	-1.601541
C	-5.243948	2.480476	-0.579826
C	-5.115324	1.021436	-2.521118
C	-6.610786	2.220933	-0.484500
H	-4.759128	3.145256	0.127588
C	-6.481583	0.779909	-2.413129
H	-4.519442	0.561381	-3.299438
C	-7.240598	1.373874	-1.399607
H	-7.186021	2.690130	0.308852
H	-6.957083	0.111360	-3.125089
H	-8.305767	1.177997	-1.324408
C	-0.357397	3.490844	-2.668601
H	0.313097	2.998540	-3.381346
H	0.243101	4.198681	-2.093219
H	-1.086811	4.075495	-3.253087
C	1.242716	2.686451	-0.166908
H	2.008699	2.206795	0.457023
H	1.784881	3.251673	-0.936543
C	0.453578	3.633175	0.740454
H	-0.220597	3.054766	1.386553
H	-0.191759	4.291433	0.145964
C	1.334504	4.507872	1.645669
H	1.997995	5.128810	1.025781
H	1.993443	3.857760	2.240220
C	0.529062	5.402461	2.592017
H	-0.130513	6.055386	2.001997
H	-0.134273	4.771091	3.201150
C	1.395012	6.263633	3.516490
H	2.048899	6.902726	2.907445
H	2.061682	5.610896	4.096918
C	0.575061	7.133359	4.471256
H	-0.083735	7.813996	3.918801
H	1.218038	7.743757	5.115206
H	-0.058639	6.517746	5.120980
H	-3.023515	0.015734	-0.394068
C	-1.869989	0.215448	1.382321
C	-2.843047	1.015047	1.985665
C	-0.626330	0.058587	2.009174
C	-2.582633	1.653884	3.200750
H	-3.804444	1.144798	1.497590
C	-0.365494	0.693233	3.221443
H	0.134706	-0.562243	1.551528
C	-1.342376	1.494919	3.820333
H	-3.345813	2.276932	3.657787

H	0.600972	0.563088	3.699158
H	-1.136065	1.992849	4.763270
H	3.054762	0.526417	0.784232
C	3.859649	0.992134	-1.123941
C	4.934066	1.716339	-0.596562
C	3.567326	1.101839	-2.486151
C	5.718542	2.522164	-1.419485
H	5.159971	1.640201	0.463349
C	4.347138	1.914776	-3.311086
H	2.724134	0.566973	-2.906921
C	5.426972	2.622941	-2.782204
H	6.552614	3.074869	-0.997147
H	4.107526	1.993827	-4.367555
H	6.034173	3.253632	-3.424803

[1]TS10-*R*

B3LYP/BSI SCF energy: -2922.086423a.u.

M06/BSII SCF energy in solution: -2920.78595a.u.

M06/BSII free energy in solution: -2919.822017a.u.

C	0.694164	-0.760374	-1.727861
N	1.176237	-0.342841	-0.611444
O	1.387754	-1.739812	-2.358739
C	2.379371	-2.262568	-1.418415
C	2.436635	-1.079152	-0.354494
C	-1.704273	-0.037079	-1.564494
N	-1.740707	-0.129663	-0.294468
O	-2.841911	0.314617	-2.217667
C	-3.915665	0.457603	-1.221829
C	-3.080546	0.279264	0.143973
Ni	0.352003	0.659359	0.867173
C	-0.514816	-0.240643	-2.491226
C	-0.916076	-1.229646	-3.612686
H	-0.085519	-1.374374	-4.305570
H	-1.198458	-2.205589	-3.208130
H	-1.763918	-0.819477	-4.163158
C	-0.143625	1.131270	-3.126835
H	-0.985173	1.492053	-3.723236
H	0.078053	1.871075	-2.355075
H	0.720832	1.006686	-3.786809
C	-4.456840	1.884583	-1.390268
C	-5.762338	2.157302	-1.817957
C	-3.583719	2.965350	-1.170682
C	-6.198263	3.473947	-1.988457
H	-6.448737	1.345304	-2.024499
C	-4.020530	4.277063	-1.347590
H	-2.548874	2.801078	-0.880620
C	-5.332176	4.539163	-1.749750
H	-7.217603	3.660266	-2.315254
H	-3.325740	5.093681	-1.173249
H	-5.671481	5.562646	-1.883318
C	-4.945534	-0.641668	-1.474558
C	-6.086199	-0.744281	-0.660617
C	-4.783192	-1.572197	-2.507145
C	-7.028216	-1.749035	-0.870888
H	-6.238984	-0.036594	0.146461
C	-5.726743	-2.581528	-2.717087
H	-3.918730	-1.502707	-3.154806
C	-6.851873	-2.675921	-1.900680
H	-7.900114	-1.808239	-0.225730
H	-5.578462	-3.291329	-3.526291
H	-7.585547	-3.460158	-2.063984
C	3.664640	-2.528806	-2.200690
C	4.597931	-3.474003	-1.755444

C	3.945129	-1.810476	-3.370990
C	5.785018	-3.688795	-2.456299
H	4.396810	-4.057985	-0.864118
C	5.127651	-2.032408	-4.076108
H	3.230288	-1.083280	-3.736749
C	6.054905	-2.970339	-3.620844
H	6.494393	-4.426929	-2.092982
H	5.322658	-1.468535	-4.983884
H	6.975928	-3.142082	-4.170312
C	1.795266	-3.553294	-0.822564
C	0.949269	-4.351029	-1.606885
C	2.130069	-4.002315	0.461895
C	0.437342	-5.550227	-1.113494
H	0.691417	-4.024347	-2.607769
C	1.623588	-5.207797	0.954357
H	2.795320	-3.422649	1.094462
C	0.771440	-5.984232	0.170736
H	-0.221600	-6.148258	-1.736626
H	1.895793	-5.533646	1.954119
H	0.373244	-6.918654	0.555185
C	0.158359	2.273784	2.010092
C	0.382818	2.730663	0.545771
C	1.557174	2.904194	2.287708
H	1.515411	3.775622	2.955260
H	2.349100	2.227084	2.615819
N	1.644290	3.283653	0.874928
O	-0.341003	3.001696	-0.426431
C	2.520374	4.135048	0.224729
C	3.671623	4.600560	0.888896
C	2.278758	4.551415	-1.101851
C	4.561694	5.451569	0.237625
H	3.867860	4.285427	1.909029
C	3.182778	5.399647	-1.735487
H	1.377541	4.215196	-1.599068
C	4.330772	5.856092	-1.079122
H	5.445165	5.799304	0.766800
H	2.981506	5.715501	-2.756124
H	5.026597	6.520822	-1.582035
C	-1.029515	2.965229	2.670530
H	-1.969439	2.625425	2.227421
H	-1.077799	2.783452	3.749876
H	-0.966996	4.049871	2.514700
C	-0.054243	0.487388	2.760763
H	0.078924	-0.514199	2.255075
H	-1.110931	0.553964	3.030228
C	0.824287	0.487200	4.011727
H	1.877480	0.374913	3.725909
H	0.746039	1.448325	4.539178
C	0.453228	-0.637250	4.994181
H	-0.606982	-0.544430	5.268387
H	0.551629	-1.608409	4.488944
C	1.311012	-0.633792	6.266075
H	1.207586	0.337756	6.770692
H	2.372070	-0.718768	5.989556
C	0.956164	-1.753937	7.252525
H	-0.104361	-1.671112	7.526796
H	1.063449	-2.725289	6.750501
C	1.814973	-1.736554	8.521157
H	1.700747	-0.790922	9.063461
H	1.538668	-2.546589	9.204257
H	2.878402	-1.851778	8.281620
C	-3.636440	-0.636346	1.215558
C	-4.367253	-0.092675	2.279857
C	-3.418513	-2.019393	1.184664

C	-4.889838	-0.913052	3.281652
H	-4.529058	0.982147	2.323282
C	-3.934188	-2.840387	2.186857
H	-2.836137	-2.447923	0.375564
C	-4.675100	-2.291197	3.236296
H	-5.455329	-0.474464	4.099145
H	-3.755795	-3.911451	2.149645
H	-5.075199	-2.932309	4.016742
H	-2.981683	1.273249	0.589690
H	2.368419	-1.503434	0.646779
C	3.663966	-0.186583	-0.393342
C	4.785185	-0.545913	0.366513
C	3.721797	0.974109	-1.171665
C	5.946080	0.226094	0.335654
H	4.752053	-1.439168	0.986299
C	4.878321	1.755081	-1.196256
H	2.849825	1.289256	-1.734214
C	5.995176	1.379927	-0.448682
H	6.806552	-0.069113	0.929704
H	4.894661	2.669414	-1.781071
H	6.893204	1.990656	-0.466438

[1]**TS10**-*R*

B3LYP-D3BJ/BSI SCF energy in solution:
-2922.503027a.u.

M06/BSII SCF energy in solution: -2920.794502a.u.

M06/BSII free energy in solution: -2919.821961a.u.

B3LYP-D3BJ/BSI SCF energy: -2922.503027a.u.

B3LYP-D3BJ/BSII SCF energy in solution:
-2923.158794a.u.

B3LYP-D3BJ /BSII free energy in solution:
-2922.186253 a.u.

C	0.652421	0.534539	-1.764326
N	1.161093	0.501748	-0.585699
O	1.203628	-0.285216	-2.693184
C	2.065863	-1.217386	-1.957402
C	2.329342	-0.393982	-0.629056
C	-1.710330	1.077680	-1.322830
N	-1.672448	0.649465	-0.118630
O	-2.918117	1.368569	-1.844346
C	-3.899565	1.275107	-0.739350
C	-3.044431	0.547740	0.386609
Ni	0.027239	0.545573	1.076140
C	-0.509592	1.403815	-2.194217
C	-0.843548	1.198850	-3.680995
H	0.031429	1.426715	-4.293333
H	-1.149748	0.170613	-3.888220
H	-1.654695	1.868360	-3.970974
C	-0.154055	2.890310	-1.916879
H	-1.005714	3.525695	-2.174874
H	0.094048	3.038931	-0.863613
H	0.698571	3.184036	-2.535668
C	-4.212110	2.721768	-0.368728
C	-5.366568	3.356992	-0.840935
C	-3.279381	3.471861	0.368955
C	-5.601494	4.704990	-0.564819
H	-6.087811	2.798468	-1.426163
C	-3.513766	4.820048	0.636942
H	-2.356943	3.027086	0.731487
C	-4.677285	5.440720	0.177052
H	-6.506478	5.177968	-0.934757
H	-2.781995	5.383530	1.208377

H	-4.859667	6.489314	0.393139
C	-5.082850	0.461829	-1.207029
C	-6.183508	0.311084	-0.352091
C	-5.075219	-0.209163	-2.432025
C	-7.255910	-0.498831	-0.717476
H	-6.196107	0.826563	0.602850
C	-6.153111	-1.018687	-2.799167
H	-4.226321	-0.099976	-3.095897
C	-7.244475	-1.168191	-1.944312
H	-8.099627	-0.609626	-0.042907
H	-6.134657	-1.533464	-3.755367
H	-8.080406	-1.800250	-2.228935
C	3.298215	-1.481908	-2.801758
C	4.016115	-2.676594	-2.684383
C	3.788094	-0.478030	-3.648370
C	5.208468	-2.859194	-3.387250
H	3.651320	-3.470939	-2.043886
C	4.975512	-0.663239	-4.353348
H	3.239837	0.450461	-3.748354
C	5.694330	-1.853500	-4.222305
H	5.753628	-3.792504	-3.281038
H	5.341044	0.126253	-5.003442
H	6.621952	-1.995723	-4.768779
C	1.240782	-2.468366	-1.661683
C	0.160057	-2.811461	-2.483300
C	1.570973	-3.313687	-0.594194
C	-0.581732	-3.966949	-2.234173
H	-0.100007	-2.171041	-3.317968
C	0.835890	-4.475014	-0.351441
H	2.409352	-3.079080	0.053065
C	-0.247220	-4.802995	-1.167208
H	-1.421742	-4.213049	-2.877086
H	1.109289	-5.116649	0.480063
H	-0.823838	-5.702301	-0.973363
C	0.469619	0.807372	3.001356
C	0.724102	2.062705	2.156203
C	2.009367	0.774717	3.215214
H	2.303148	0.958854	4.256392
H	2.547566	-0.094598	2.830680
N	2.130301	1.970794	2.377253
O	0.064687	3.062242	1.797617
C	3.186779	2.820873	2.164052
C	4.475820	2.481286	2.624030
C	3.002305	4.048250	1.488774
C	5.543604	3.350907	2.418351
H	4.630469	1.536114	3.134577
C	4.083339	4.902358	1.295868
H	2.008832	4.304759	1.142863
C	5.362019	4.568109	1.755870
H	6.530479	3.068744	2.775693
H	3.923900	5.844332	0.776976
H	6.199233	5.241148	1.598655
C	-0.389537	1.021211	4.235393
H	-1.416606	1.268056	3.950296
H	-0.420311	0.141005	4.887326
H	0.003778	1.860164	4.824300
C	-0.287608	-0.889850	2.399339
H	-0.521855	-1.265081	1.378534
H	-1.238689	-0.850587	2.934464
C	0.627762	-1.940809	3.019609
H	1.557882	-2.004220	2.440417
H	0.913887	-1.678427	4.046695
C	-0.054619	-3.315958	3.012741
H	-0.943583	-3.282798	3.657756

H	-0.424328	-3.520158	2.000059
C	0.857803	-4.463097	3.450975
H	1.224801	-4.279473	4.470738
H	1.748649	-4.481968	2.806300
C	0.169646	-5.830377	3.396102
H	-0.708178	-5.819427	4.056060
H	-0.216753	-5.996349	2.381113
C	1.091164	-6.986319	3.788090
H	1.468862	-6.863712	4.810104
H	0.570926	-7.949310	3.739766
H	1.959971	-7.044950	3.121349
C	-3.390461	-0.888253	0.708489
C	-4.100855	-1.178989	1.877055
C	-2.986021	-1.938010	-0.123047
C	-4.412564	-2.498175	2.209969
H	-4.404044	-0.366360	2.532001
C	-3.295661	-3.256733	0.207345
H	-2.409021	-1.728806	-1.017770
C	-4.010060	-3.540980	1.374018
H	-4.961581	-2.710068	3.122860
H	-2.968097	-4.062353	-0.441188
H	-4.244811	-4.569365	1.632536
H	-3.124176	1.124056	1.309051
H	2.288779	-1.065413	0.225760
C	3.636660	0.360930	-0.587800
C	4.782528	-0.300515	-0.130966
C	3.748334	1.674671	-1.051150
C	6.024507	0.330971	-0.160864
H	4.700792	-1.319728	0.237021
C	4.989557	2.310478	-1.075932
H	2.862375	2.205481	-1.379921
C	6.131337	1.639270	-0.638351
H	6.906733	-0.194538	0.193075
H	5.060623	3.336202	-1.423324
H	7.096070	2.137312	-0.654092

[1]**TS10**-*R*

*w*B97XD/BSI SCF energy in gas:

-2921.280011a.u.

*w*B97XD/BSII SCF energy in solution:

-2921.930066a.u.

*w*B97XD/BSII free energy in solution:

-2920.943388a.u.

C	0.750712	0.727469	-1.745337
N	1.220911	0.641915	-0.559354
O	1.374721	0.004276	-2.700812
C	2.147115	-0.989014	-1.978960
C	2.404069	-0.220276	-0.630601
C	-1.641478	1.001009	-1.342987
N	-1.573371	0.395381	-0.229526
O	-2.860769	1.316009	-1.810431
C	-3.841226	0.757959	-0.882395
C	-2.912194	0.326136	0.329643
Ni	0.074546	0.458092	1.103494
C	-0.470179	1.514835	-2.157456
C	-0.750527	1.370644	-3.659175
H	0.097052	1.744546	-4.237431
H	-0.918845	0.327967	-3.938703
H	-1.639196	1.947912	-3.919455
C	-0.281727	3.000766	-1.776738
H	-1.164570	3.569452	-2.080198
H	-0.154625	3.102120	-0.697678
H	0.594208	3.408185	-2.289875

C	-4.768629	1.911921	-0.520031
C	-6.101765	1.965476	-0.924861
C	-4.229446	2.999102	0.181820
C	-6.895886	3.063874	-0.601430
H	-6.528620	1.150281	-1.497939
C	-5.022103	4.095237	0.497463
H	-3.182974	3.005211	0.480700
C	-6.361398	4.128322	0.114464
H	-7.934184	3.085105	-0.917802
H	-4.587756	4.926766	1.043150
H	-6.981054	4.983168	0.367367
C	-4.488805	-0.446765	-1.544414
C	-5.530785	-1.122659	-0.900048
C	-3.987730	-0.968987	-2.735373
C	-6.055795	-2.291598	-1.434473
H	-5.921842	-0.739920	0.037026
C	-4.512295	-2.143693	-3.270463
H	-3.185405	-0.453121	-3.249113
C	-5.545217	-2.810080	-2.622485
H	-6.860946	-2.803009	-0.916703
H	-4.109902	-2.534590	-4.200405
H	-5.953237	-3.725606	-3.039105
C	3.386528	-1.321925	-2.783246
C	4.002126	-2.568231	-2.673897
C	3.983552	-0.336424	-3.573073
C	5.204914	-2.819674	-3.328640
H	3.545288	-3.351276	-2.078159
C	5.179847	-0.590736	-4.231236
H	3.513009	0.635976	-3.656890
C	5.798397	-1.832189	-4.106836
H	5.674766	-3.793252	-3.230484
H	5.633820	0.186830	-4.837196

H	6.736953	-2.027884	-4.615728
C	1.214823	-2.187831	-1.762870
C	0.193919	-2.427681	-2.686005
C	1.359239	-3.065895	-0.686789
C	-0.670724	-3.505128	-2.531808
H	0.078969	-1.759017	-3.530936
C	0.503053	-4.154821	-0.538208
H	2.142704	-2.914944	0.050438
C	-0.515754	-4.377464	-1.458304
H	-1.469460	-3.660391	-3.249961
H	0.631999	-4.824696	0.306466
H	-1.188551	-5.220683	-1.338029
C	0.041800	0.966975	2.994556
C	-0.045562	2.211147	2.095073
C	1.441884	1.508338	3.394417
H	1.529052	1.785598	4.452708
H	2.295454	0.888448	3.103402
N	1.224597	2.658292	2.527020
O	-0.940386	2.867959	1.542904
C	2.125509	3.565105	2.003654
C	3.464643	3.550559	2.415458
C	1.703162	4.525649	1.069918
C	4.362927	4.474947	1.896556
H	3.797795	2.813875	3.139405
C	2.617902	5.433126	0.553245
H	0.656718	4.556087	0.791646
C	3.951601	5.420519	0.961211
H	5.398490	4.446721	2.222335
H	2.278448	6.172279	-0.166994
H	4.657344	6.142040	0.561874
C	-1.018234	0.894638	4.079869
H	-1.999263	0.711118	3.632468

H	-0.820267	0.097730	4.805196
H	-1.065890	1.846400	4.622505
C	0.060297	-0.892175	2.454654
H	0.072342	-1.356869	1.423997
H	-0.897610	-1.193129	2.889229
C	1.208867	-1.527727	3.229081
H	2.163598	-1.288502	2.741163
H	1.272388	-1.124157	4.249071
C	1.051369	-3.048084	3.303943
H	0.112470	-3.286473	3.821758
H	0.938815	-3.446150	2.286094
C	2.215926	-3.749446	3.999499
H	2.334359	-3.342538	5.013214
H	3.150604	-3.517648	3.469203
C	2.044949	-5.266216	4.080759
H	1.110670	-5.493997	4.609706
H	1.926678	-5.671445	3.066705
C	3.211875	-5.962750	4.777657
H	3.335852	-5.588666	5.799562
H	3.059350	-7.044543	4.833784
H	4.152132	-5.783453	4.245204
C	-3.166486	-0.997476	1.002923
C	-3.925775	-1.041796	2.170863
C	-2.604718	-2.173992	0.507084
C	-4.131835	-2.248849	2.834556
H	-4.351826	-0.122787	2.566885
C	-2.802628	-3.377005	1.172000
H	-1.994301	-2.139129	-0.390315
C	-3.567466	-3.419117	2.336470
H	-4.720647	-2.271233	3.746406
H	-2.349759	-4.283182	0.782534
H	-3.717261	-4.360649	2.855943
H	-2.957290	1.101230	1.100922
H	2.377512	-0.918784	0.205985
C	3.694090	0.565469	-0.561209
C	4.875245	-0.111847	-0.249018
C	3.746646	1.927545	-0.848786
C	6.094445	0.552588	-0.257457
H	4.839997	-1.175525	-0.026672
C	4.968507	2.594788	-0.856637
H	2.830189	2.476451	-1.036763
C	6.143556	1.910059	-0.568444
H	7.006641	0.012436	-0.023868
H	4.990996	3.659032	-1.063845
H	7.095065	2.432745	-0.574808

^{1}**TS10**-*S*

B3LYP/BSI SCF energy: -2922.088159a.u.

M06/BSII SCF energy in solution: -2920.7929746a.u.

M06/BSII free energy in solution: -2919.826071a.u.

C	0.793819	-1.824364	-0.006332
N	1.070759	-0.576316	0.075099
O	1.761782	-2.644088	-0.474630
C	2.949988	-1.840225	-0.790711
C	2.480261	-0.385096	-0.332557
C	-1.704829	-1.682272	0.251907
N	-1.803165	-0.411571	0.327566
O	-2.848088	-2.383085	0.023573
C	-3.878249	-1.412325	-0.357205
C	-3.223992	-0.058154	0.179354
Ni	-0.068094	0.942325	0.689343
C	-0.465362	-2.541833	0.458941
C	-0.623977	-3.887823	-0.284076

H	0.261631	-4.503857	-0.127189
H	-0.759595	-3.743743	-1.359814
H	-1.496339	-4.417673	0.100354
C	-0.304810	-2.807373	1.988024
H	-1.190197	-3.330832	2.362019
H	-0.167856	-1.872431	2.537736
H	0.566076	-3.449606	2.150774
C	-5.182838	-1.847229	0.308199
C	-6.425938	-1.572995	-0.276057
C	-5.155149	-2.512816	1.542195
C	-7.611040	-1.943517	0.361671
H	-6.475995	-1.078266	-1.239683
C	-6.338817	-2.889071	2.174855
H	-4.202987	-2.744056	2.004459
C	-7.573293	-2.603874	1.589116
H	-8.563989	-1.721601	-0.110316
H	-6.294046	-3.407096	3.128609
H	-8.495171	-2.897530	2.082734
C	-3.972762	-1.440829	-1.890368
C	-4.368122	-0.321745	-2.635501
C	-3.718731	-2.639019	-2.574334
C	-4.491527	-0.392764	-4.025548
H	-4.593433	0.618719	-2.142094
C	-3.835275	-2.709135	-3.961939
H	-3.426787	-3.517921	-2.011154
C	-4.220510	-1.585048	-4.695016
H	-4.797408	0.489110	-4.581050
H	-3.628066	-3.646519	-4.470498
H	-4.311179	-1.639241	-5.775932
C	4.105121	-2.433566	0.022493
C	5.045994	-1.657097	0.704789
C	4.252972	-3.830721	0.032833
C	6.107977	-2.260562	1.386359
H	4.968329	-0.576291	0.731157
C	5.304282	-4.431922	0.718724
H	3.535197	-4.445138	-0.500707
C	6.240144	-3.646478	1.398052
H	6.814918	-1.634346	1.920989
H	5.395958	-5.514447	0.720495
H	7.061912	-4.114053	1.932820
C	3.227924	-1.989221	-2.289725
C	2.274894	-2.527557	-3.161520
C	4.459531	-1.571414	-2.814538
C	2.542683	-2.637467	-4.528318
H	1.325916	-2.875126	-2.771101
C	4.723091	-1.674453	-4.178521
H	5.218660	-1.168199	-2.152282
C	3.764892	-2.208770	-5.043124
H	1.791471	-3.063025	-5.187923
H	5.682129	-1.342113	-4.565424
H	3.973057	-2.294265	-6.105738
C	0.163933	2.488604	1.968333
C	0.707706	1.186752	2.574446
C	1.643753	2.976294	1.965985
H	1.821418	3.806088	2.664271
H	2.093211	3.209287	0.998118
N	2.049875	1.683049	2.522355
O	0.257566	0.265134	3.277440
C	3.254223	1.265907	3.046745
C	4.416457	2.048078	2.869317
C	3.351416	0.052367	3.765533
C	5.634924	1.626806	3.400154
H	4.353500	2.986892	2.327110
C	4.577747	-0.347211	4.285552

H	2.453733	-0.535338	3.913966
C	5.729990	0.428502	4.111735
H	6.516214	2.247700	3.259158
H	4.633758	-1.280259	4.840308
H	6.679267	0.109920	4.531885
C	-0.800496	3.250916	2.869026
H	-1.730421	2.692982	2.997423
H	-1.045813	4.245520	2.479849
H	-0.352862	3.382251	3.862942
C	-0.756731	2.733695	0.259842
H	-0.854638	2.027887	-0.610527
H	-1.765798	2.941572	0.627328
C	-0.116070	4.004672	-0.304326
H	0.869404	3.763094	-0.719065
H	0.046097	4.749939	0.486884
C	-0.967639	4.649941	-1.411115
H	-1.960127	4.900646	-1.009428
H	-1.137607	3.914815	-2.210673
C	-0.326727	5.908547	-2.009548
H	-0.147713	6.641772	-1.209831
H	0.663446	5.651116	-2.412067
C	-1.166628	6.563043	-3.114144
H	-2.155617	6.821744	-2.711517
H	-1.347496	5.830010	-3.912493
C	-0.514394	7.815219	-3.708948
H	-0.352542	8.580323	-2.940938
H	-1.136520	8.258707	-4.493546
H	0.461367	7.581752	-4.150531
H	3.018879	-0.111360	0.576321
C	2.650276	0.746120	-1.328603
C	3.697761	1.660292	-1.165616
C	1.782516	0.902970	-2.417950
C	3.886326	2.702072	-2.077937
H	4.362755	1.569080	-0.310289
C	1.966983	1.942059	-3.328279
H	0.957844	0.210675	-2.550727
C	3.021962	2.844426	-3.163435
H	4.704519	3.402442	-1.935140
H	1.286624	2.048160	-4.168534
H	3.166050	3.652522	-3.875046
H	-3.300551	0.704051	-0.598122
C	-3.816619	0.521891	1.454508
C	-4.985042	1.292202	1.368593
C	-3.236512	0.311256	2.710932
C	-5.579556	1.819723	2.513473
H	-5.439647	1.475926	0.397845
C	-3.831550	0.843179	3.858609
H	-2.293484	-0.217403	2.796723
C	-5.005127	1.591374	3.766202
H	-6.486603	2.411410	2.427239
H	-3.362159	0.679132	4.824343
H	-5.463848	2.003809	4.660447

[1]**TS10**-*S*

B3LYP-D3BJ/BSI SCF energy in solution:

-2922.511477a.u.

M06/BSII SCF energy in solution: -2920.799952a.u.

M06/BSII free energy in solution: -2919.826138a.u.

B3LYP-D3BJ/BSI SCF energy: -2922.511477a.u.

B3LYP-D3BJ/BSII SCF energy in solution:

-2923.165507a.u.

B3LYP-D3BJ /BSII free energy in solution:

-2922.191693 a.u.

C	1.031772	-1.646560	0.999102
N	1.255179	-0.396609	0.834805
O	1.873171	-2.512544	0.401565
C	2.878573	-1.741264	-0.333379
C	2.421894	-0.239729	-0.051282
C	-1.394319	-1.605719	1.194597
N	-1.552945	-0.358033	0.956921
O	-2.433662	-2.419739	0.896737
C	-3.314435	-1.624815	0.020339
C	-2.920379	-0.165036	0.461923
Ni	-0.066113	1.089856	1.122258
C	-0.136790	-2.242870	1.767384
C	-0.181225	-3.771407	1.618349
H	0.734617	-4.207706	2.021106
H	-0.281438	-4.078453	0.575504
H	-1.029598	-4.169422	2.178283
C	-0.008736	-1.854413	3.262891
H	-0.880738	-2.223961	3.810784
H	0.075687	-0.772179	3.387341
H	0.885232	-2.327991	3.680024
C	-4.749979	-2.011297	0.298009
C	-5.729172	-1.858890	-0.689291
C	-5.130529	-2.435534	1.577365
C	-7.070133	-2.115294	-0.399445
H	-5.447930	-1.538185	-1.686344
C	-6.469118	-2.697898	1.863035
H	-4.378463	-2.555071	2.347819
C	-7.444866	-2.535024	0.877163
H	-7.819557	-1.990899	-1.175550
H	-6.750120	-3.026691	2.859192
H	-8.487861	-2.736853	1.102309
C	-2.862418	-1.915490	-1.412884
C	-2.936792	-0.955322	-2.428992
C	-2.377684	-3.192755	-1.727111
C	-2.513181	-1.257961	-3.725816
H	-3.329006	0.035399	-2.227950
C	-1.954224	-3.494005	-3.020504
H	-2.327998	-3.948945	-0.952375
C	-2.012870	-2.524580	-4.024466
H	-2.576348	-0.498388	-4.499185
H	-1.577265	-4.487797	-3.242938
H	-1.675763	-2.756058	-5.030047
C	4.240879	-2.074414	0.256157
C	5.306123	-1.173757	0.158829
C	4.460796	-3.326666	0.843992
C	6.571984	-1.520012	0.633782
H	5.153735	-0.192879	-0.277494
C	5.721637	-3.665438	1.333028
H	3.641957	-4.032489	0.922142
C	6.784065	-2.764776	1.225515
H	7.385634	-0.806499	0.552005
H	5.874413	-4.636022	1.795991
H	7.766968	-3.030839	1.602848
C	2.793511	-2.150446	-1.800916
C	1.716138	-2.899010	-2.283969
C	3.769863	-1.708861	-2.703477
C	1.616123	-3.197571	-3.644607
H	0.946590	-3.241016	-1.603380
C	3.662126	-1.995638	-4.062035
H	4.613665	-1.129304	-2.348041
C	2.583096	-2.743245	-4.539675
H	0.772971	-3.779860	-4.001146
H	4.423611	-1.635383	-4.747425

H	2.500403	-2.970392	-5.598418
C	0.050798	3.039897	1.689500
C	0.817290	2.097563	2.587542
C	1.430205	3.638998	1.300922
H	1.549670	4.687806	1.598246
H	1.745506	3.510608	0.263711
N	2.072675	2.708205	2.231895
O	0.558607	1.429334	3.616110
C	3.369869	2.261125	2.293211
C	4.393504	2.921134	1.582644
C	3.700773	1.140176	3.088427
C	5.712578	2.478983	1.683225
H	4.148509	3.781759	0.968059
C	5.021924	0.714702	3.171541
H	2.906304	0.637269	3.626937
C	6.041483	1.380137	2.480376
H	6.489912	3.004340	1.134234
H	5.260063	-0.154285	3.779194
H	7.069434	1.041161	2.556141
C	-0.978947	3.930910	2.356505
H	-1.781502	3.325626	2.787223
H	-1.429643	4.657620	1.672449
H	-0.506756	4.490070	3.175209
C	-1.005608	2.525253	0.098986
H	-1.005572	1.601549	-0.511679
H	-2.017765	2.682376	0.479386
C	-0.618125	3.626772	-0.884587
H	0.376021	3.418543	-1.292866
H	-0.567380	4.610569	-0.401033
C	-1.613066	3.690496	-2.050563
H	-2.616668	3.919796	-1.664790
H	-1.679600	2.695277	-2.510896

C	-1.229050	4.712525	-3.123454
H	-1.197592	5.717419	-2.679323
H	-0.209036	4.499276	-3.471388
C	-2.180581	4.715380	-4.323169
H	-3.201599	4.924537	-3.975837
H	-2.207638	3.707924	-4.760953
C	-1.789111	5.728160	-5.400734
H	-1.782336	6.749306	-5.001633
H	-2.486299	5.706899	-6.245622
H	-0.785911	5.521308	-5.792144
H	3.202382	0.277797	0.505895
C	2.074246	0.578194	-1.272423
C	2.935376	1.595933	-1.689106
C	0.923035	0.305718	-2.025007
C	2.666913	2.321198	-2.854125
H	3.817271	1.821919	-1.096441
C	0.658047	1.020648	-3.189702
H	0.238700	-0.473323	-1.708370
C	1.531999	2.028796	-3.610819
H	3.344418	3.109975	-3.167958
H	-0.229241	0.787696	-3.770205
H	1.324531	2.583683	-4.521020
H	-2.892313	0.485525	-0.409746
C	-3.810006	0.485462	1.497243
C	-4.972714	1.140740	1.073938
C	-3.514967	0.433554	2.862537
C	-5.838800	1.717311	2.001161
H	-5.204368	1.187747	0.013544
C	-4.377459	1.018733	3.792335
H	-2.605468	-0.050020	3.199020
C	-5.542737	1.657166	3.365678
H	-6.740639	2.217029	1.659767

H -4.134744 0.975959 4.850141
H -6.213756 2.109990 4.089653

[1]**TS10**-*S*

*w*B97XD/BSI SCF energy in gas:

-2921.292245a.u.

*w*B97XD/BSII SCF energy in solution:

-2921.939113a.u.

*w*B97XD/BSII free energy in solution:

-2920.948575a.u.

C 0.895462 -1.747020 0.688467
N 1.160731 -0.500487 0.658731
O 1.818683 -2.584856 0.187873
C 2.825689 -1.788394 -0.481673
C 2.493941 -0.337424 0.065795
C -1.556108 -1.625068 0.650779
N -1.611661 -0.376310 0.409103
O -2.677654 -2.341870 0.429538
C -3.540848 -1.493512 -0.373077
C -2.975285 -0.065981 -0.005360
Ni -0.093747 1.028030 1.005334
C -0.373182 -2.400046 1.200904
C -0.443976 -3.868959 0.752024
H 0.407357 -4.417096 1.158367
H -0.425272 -3.972118 -0.335502
H -1.364626 -4.318205 1.127354
C -0.411368 -2.328241 2.744989
H -1.326733 -2.806124 3.106342
H -0.376005 -1.293304 3.095592
H 0.444884 -2.875159 3.151064
C -4.977099 -1.757049 0.040494
C -6.025516 -1.685338 -0.875185
C -5.267333 -2.004647 1.385148
C -7.343458 -1.842436 -0.452631
H -5.821817 -1.511782 -1.926126
C -6.581038 -2.165180 1.804677
H -4.460013 -2.057522 2.106245
C -7.625585 -2.080699 0.887057
H -8.147982 -1.784068 -1.178944
H -6.788780 -2.350891 2.853504
H -8.652665 -2.204068 1.215850
C -3.258298 -1.835766 -1.837886
C -3.486862 -0.914898 -2.862628
C -2.794227 -3.109367 -2.174298
C -3.240879 -1.251329 -4.190943
H -3.873252 0.074571 -2.636789
C -2.543786 -3.446090 -3.500937
H -2.631984 -3.838579 -1.389128
C -2.763361 -2.517048 -4.514679
H -3.422685 -0.519382 -4.971350
H -2.177783 -4.439457 -3.741463
H -2.568222 -2.778597 -5.549895
C 4.192107 -2.274977 -0.034143
C 5.312848 -1.452162 -0.159794
C 4.349097 -3.552067 0.503300
C 6.569472 -1.902523 0.229204
H 5.205079 -0.446522 -0.553524
C 5.603462 -3.995664 0.910375
H 3.483470 -4.194798 0.613285
C 6.719342 -3.175559 0.770023
H 7.428791 -1.247428 0.125833
H 5.708737 -4.988071 1.337603
H 7.697755 -3.525226 1.084173

C	2.592518	-1.950081	-1.985783
C	1.329034	-2.300744	-2.469314
C	3.598028	-1.656832	-2.908030
C	1.071022	-2.335147	-3.835510
H	0.523642	-2.533682	-1.782808
C	3.342233	-1.691784	-4.274258
H	4.591241	-1.393295	-2.564320
C	2.076501	-2.026080	-4.745050
H	0.074638	-2.594362	-4.179503
H	4.138176	-1.453329	-4.972636
H	1.877697	-2.049369	-5.811941
C	0.014449	2.721028	2.078361
C	0.372938	1.505857	2.897124
C	1.514852	3.102410	2.191883
H	1.693734	4.046107	2.720259
H	2.084006	3.085516	1.257365
N	1.735201	1.926887	3.036338
O	-0.248700	0.698658	3.609490
C	2.867792	1.135462	3.103794
C	4.120072	1.640010	2.727016
C	2.768934	-0.200566	3.529951
C	5.239125	0.810433	2.735398
H	4.210388	2.677628	2.419823
C	3.892689	-1.014566	3.521239
H	1.801298	-0.572722	3.843500
C	5.134817	-0.522494	3.120283
H	6.200591	1.214268	2.430132
H	3.797302	-2.052997	3.825095
H	6.001903	-1.173264	3.099706
C	-1.005448	3.650039	2.715747
H	-1.971377	3.147025	2.800932
H	-1.147429	4.579736	2.154876
H	-0.673281	3.911531	3.727669
C	-0.696768	2.727491	0.274223
H	-0.715665	1.941158	-0.525586
H	-1.738155	2.994259	0.481789
C	0.058964	3.899295	-0.345294
H	1.088013	3.591680	-0.562611
H	0.120082	4.747461	0.349270
C	-0.589106	4.367028	-1.648216
H	-1.628873	4.667659	-1.457967
H	-0.636479	3.517694	-2.343736
C	0.164614	5.516104	-2.314121
H	0.161840	6.390345	-1.648468
H	1.217716	5.229057	-2.436688
C	-0.413006	5.908698	-3.673255
H	-1.470346	6.177706	-3.552621
H	-0.395892	5.032431	-4.335362
C	0.337741	7.062333	-4.334947
H	0.307827	7.960610	-3.708895
H	-0.094558	7.317277	-5.307074
H	1.391212	6.806539	-4.493199
H	3.188804	-0.119505	0.880775
C	2.553640	0.798112	-0.929141
C	3.602730	1.714203	-0.862594
C	1.591896	0.946993	-1.933516
C	3.716976	2.737142	-1.803440
H	4.327715	1.632779	-0.057071
C	1.709190	1.957559	-2.878218
H	0.753000	0.261435	-1.979230
C	2.777585	2.851918	-2.821866
H	4.539133	3.442864	-1.737091
H	0.963165	2.049436	-3.661282
H	2.867687	3.639039	-3.564259

H -2.928486 0.557740 -0.900377
C -3.735615 0.698095 1.058433
C -4.910539 1.361274 0.696117
C -3.310327 0.738503 2.385119
C -5.666582 2.033350 1.647652
H -5.247916 1.331339 -0.337299
C -4.065983 1.420128 3.337638
H -2.366420 0.292436 2.683344
C -5.245415 2.061875 2.976156
H -6.582230 2.536712 1.353510
H -3.711158 1.456857 4.362873
H -5.830977 2.591493 3.721333

^{3}**TS10**-*R*

B3LYP/BSI SCF energy: -2922.064527a.u.

M06/BSII SCF energy in solution: -2920.73421a.u.

M06/BSII free energy in solution: -2919.771554a.u.

C 0.639579 -0.739596 -1.824695
N 1.076286 -0.451492 -0.634115
O 1.296230 -1.741838 -2.470643
C 2.111852 -2.431227 -1.471154
C 2.250399 -1.298971 -0.360578
C -1.643989 0.133561 -1.594173
N -1.562183 0.469809 -0.347815
O -2.885006 -0.025553 -2.103959
C -3.840684 0.494462 -1.106033
C -2.929178 0.525280 0.200084
Ni 0.207063 0.829093 0.583495
C -0.487762 -0.047069 -2.562485
C -0.927484 -0.878022 -3.786253
H -0.090230 -0.987216 -4.478219
H -1.265652 -1.876422 -3.497772
H -1.746155 -0.371650 -4.299317
C -0.025595 1.366202 -3.040912
H -0.832742 1.836499 -3.610696
H 0.213742 2.013421 -2.195443
H 0.848145 1.264252 -3.693388
C -4.229878 1.898493 -1.593305
C -5.373641 2.084116 -2.385741
C -3.395424 3.003176 -1.350885
C -5.693404 3.341345 -2.897913
H -6.021502 1.242431 -2.602881
C -3.717667 4.259028 -1.869139
H -2.471337 2.915394 -0.786764
C -4.868530 4.436687 -2.636870
H -6.588062 3.461613 -3.502599
H -3.053250 5.094221 -1.667957
H -5.118069 5.416925 -3.033626
C -5.021302 -0.461072 -1.024620
C -6.110675 -0.138862 -0.201051
C -5.050686 -1.662581 -1.739027
C -7.197803 -1.002613 -0.088466
H -6.111801 0.798902 0.346495
C -6.143728 -2.526420 -1.629478
H -4.220225 -1.918155 -2.386024
C -7.218774 -2.202520 -0.803713
H -8.030649 -0.736559 0.556168
H -6.151171 -3.453793 -2.195437
H -8.067649 -2.874868 -0.718984
C 3.408879 -2.864720 -2.150557
C 4.134140 -3.971514 -1.691378
C 3.912529 -2.135065 -3.236459
C 5.339784 -4.332930 -2.294569

H	3.754211	-4.565471	-0.867379
C	5.112889	-2.501826	-3.843349
H	3.356904	-1.283865	-3.610525
C	5.834038	-3.600385	-3.373404
H	5.886306	-5.195281	-1.923428
H	5.484117	-1.925671	-4.686083
H	6.769244	-3.885113	-3.846812
C	1.290277	-3.630366	-0.968372
C	0.398585	-4.264351	-1.847031
C	1.435914	-4.155720	0.322521
C	-0.339480	-5.374926	-1.439662
H	0.287285	-3.879785	-2.854393
C	0.701654	-5.272828	0.730076
H	2.129756	-3.707046	1.026630
C	-0.193186	-5.883954	-0.147454
H	-1.027342	-5.846139	-2.136052
H	0.833041	-5.661264	1.735930
H	-0.765660	-6.751004	0.169001
C	0.210639	2.534844	1.985630
C	0.406494	3.052677	0.562306
C	1.694033	2.942526	2.217813
H	1.795515	3.802690	2.893616
H	2.399143	2.164104	2.521441
N	1.788272	3.322881	0.805999
O	-0.333673	3.497168	-0.339091
C	2.739027	4.081334	0.154642
C	3.959424	4.392373	0.792881
C	2.517570	4.548059	-1.162007
C	4.930338	5.134418	0.126476
H	4.142756	4.041558	1.803991
C	3.504349	5.284143	-1.809164
H	1.566111	4.340206	-1.636135

C	4.719841	5.583502	-1.180886
H	5.863193	5.362716	0.636044
H	3.317624	5.637886	-2.820272
H	5.480608	6.162124	-1.695764
C	-0.873867	3.288916	2.743149
H	-1.851659	3.098235	2.291414
H	-0.928222	3.018398	3.802047
H	-0.691460	4.368617	2.674712
C	-0.131198	0.726271	2.791176
H	-0.027805	-0.242963	2.270962
H	-1.187034	0.843877	3.029932
C	0.748915	0.701146	4.033903
H	1.787593	0.480967	3.754332
H	0.767053	1.685663	4.522220
C	0.292439	-0.343864	5.069807
H	-0.748056	-0.137078	5.355855
H	0.285015	-1.338952	4.603858
C	1.172748	-0.372906	6.326579
H	1.181931	0.625785	6.786672
H	2.213214	-0.581702	6.037881
C	0.722888	-1.403846	7.369861
H	-0.316567	-1.195740	7.658027
H	0.715485	-2.402418	6.911790
C	1.606087	-1.422131	8.621720
H	1.605097	-0.446468	9.121081
H	1.260089	-2.166523	9.346504
H	2.645719	-1.661539	8.370058
C	-3.154135	-0.575766	1.223313
C	-3.840477	-0.292848	2.410728
C	-2.673790	-1.875943	1.018406
C	-4.057205	-1.286919	3.367315
H	-4.208279	0.715064	2.588989

C	-2.884455	-2.870020	1.973447
H	-2.123475	-2.111071	0.112972
C	-3.579942	-2.579738	3.150435
H	-4.592519	-1.048262	4.282103
H	-2.500021	-3.870557	1.796759
H	-3.742862	-3.354307	3.894452
H	-3.075014	1.485983	0.699158
H	2.123048	-1.754601	0.622767
C	3.554327	-0.517112	-0.334964
C	4.630208	-1.014606	0.414269
C	3.717766	0.686034	-1.030993
C	5.847936	-0.336807	0.449939
H	4.516579	-1.943489	0.968697
C	4.932556	1.373116	-0.987872
H	2.884247	1.106936	-1.582259
C	6.002147	0.860337	-0.252888
H	6.672227	-0.738873	1.032544
H	5.031100	2.319024	-1.511005
H	6.945705	1.397135	-0.216516

^{3}TS10-*S*

B3LYP/BSI SCF energy: -2922.065909a.u.

M06/BSII SCF energy in solution: -2920.760588a.u.

M06/BSII free energy in solution: -2919.798559a.u.

C	1.018871	-1.820098	0.411810
N	1.170971	-0.555348	0.189579
O	2.061398	-2.619925	0.086513
C	3.109216	-1.787500	-0.531180
C	2.562387	-0.322985	-0.241345
C	-1.430644	-1.767416	0.693643
N	-1.614822	-0.489632	0.600448
O	-2.511470	-2.549095	0.442391
C	-3.530232	-1.693659	-0.172962
C	-3.036806	-0.254850	0.298447
Ni	-0.107093	0.857139	0.674840
C	-0.155668	-2.480972	1.105887
C	-0.226004	-3.980483	0.747644
H	0.701332	-4.471948	1.045238
H	-0.370502	-4.136433	-0.324569
H	-1.058756	-4.449202	1.274861
C	0.037099	-2.327848	2.650876
H	-0.798498	-2.810032	3.169782
H	0.083574	-1.276584	2.947371
H	0.962467	-2.829653	2.951632
C	-4.887539	-2.150181	0.356816
C	-6.053721	-1.999484	-0.404009
C	-4.989705	-2.709652	1.638373
C	-7.293206	-2.387732	0.107263
H	-5.998976	-1.588742	-1.406267
C	-6.226868	-3.105083	2.144588
H	-4.096095	-2.842005	2.236450
C	-7.385242	-2.943605	1.382774
H	-8.185184	-2.262731	-0.500066
H	-6.283795	-3.540084	3.138326
H	-8.348692	-3.251841	1.778406
C	-3.409899	-1.876641	-1.694343
C	-3.745025	-0.864115	-2.603472
C	-3.007563	-3.120695	-2.203446
C	-3.664980	-1.082339	-3.981497
H	-4.081471	0.106075	-2.251316
C	-2.922017	-3.337448	-3.577995
H	-2.760362	-3.919104	-1.513182
C	-3.248455	-2.317953	-4.474447

H	-3.928441	-0.281053	-4.665874
H	-2.603966	-4.307903	-3.948655
H	-3.182455	-2.486543	-5.545310
C	4.413070	-2.166797	0.178985
C	5.210616	-1.272288	0.898450
C	4.815006	-3.512165	0.105394
C	6.381427	-1.709207	1.527537
H	4.942168	-0.227947	0.999997
C	5.973556	-3.949281	0.739425
H	4.207653	-4.217077	-0.453726
C	6.766166	-3.044904	1.452950
H	6.975849	-0.992939	2.086205
H	6.260508	-4.995076	0.675208
H	7.672968	-3.382760	1.946472
C	3.177928	-2.136769	-2.020104
C	2.143422	-2.825241	-2.662848
C	4.292429	-1.738758	-2.773025
C	2.217566	-3.103828	-4.030238
H	1.282133	-3.156125	-2.094802
C	4.363477	-2.011991	-4.137260
H	5.108546	-1.213500	-2.287314
C	3.324989	-2.696819	-4.772765
H	1.405861	-3.642908	-4.510674
H	5.234287	-1.693897	-4.703286
H	3.382792	-2.914764	-5.835329
C	-0.246485	2.818914	1.767222
C	0.397916	1.732747	2.603306
C	1.179166	3.409556	1.598799
H	1.322261	4.330215	2.181693
H	1.561852	3.561086	0.585553
N	1.718063	2.228720	2.285936
O	0.039578	0.956838	3.514831
C	2.976932	1.987069	2.786901
C	4.070876	2.800190	2.405929
C	3.206238	0.908622	3.676744
C	5.347406	2.532943	2.895069
H	3.909420	3.639116	1.735314
C	4.489648	0.663415	4.150624
H	2.360871	0.308769	3.991893
C	5.575015	1.463291	3.767773
H	6.172542	3.174651	2.595416
H	4.645606	-0.164396	4.837846
H	6.570517	1.267852	4.154575
C	-1.321559	3.623714	2.473438
H	-2.171745	2.982768	2.718309
H	-1.684674	4.467509	1.878505
H	-0.924886	4.017757	3.417774
C	-1.141675	2.728546	-0.052136
H	-1.059318	1.877715	-0.753088
H	-2.173370	2.780895	0.295454
C	-0.696594	3.985504	-0.785565
H	0.307789	3.835389	-1.200922
H	-0.622351	4.837347	-0.094808
C	-1.643896	4.374862	-1.936761
H	-2.653552	4.539154	-1.534314
H	-1.727143	3.530124	-2.635215
C	-1.191207	5.623998	-2.705053
H	-1.103954	6.469494	-2.007549
H	-0.181062	5.455899	-3.105459
C	-2.131880	6.011926	-3.853184
H	-3.140111	6.183731	-3.452024
H	-2.222712	5.164760	-4.546991
C	-1.669983	7.254003	-4.622274
H	-1.601391	8.126103	-3.961921

H	-2.361972	7.505071	-5.433030
H	-0.679757	7.099362	-5.066188
H	3.082663	0.078751	0.631661
C	2.669974	0.709751	-1.347351
C	3.692231	1.666013	-1.301949
C	1.752951	0.751175	-2.407539
C	3.808339	2.635336	-2.301414
H	4.391125	1.667176	-0.469929
C	1.866088	1.720215	-3.404346
H	0.951584	0.021391	-2.450836
C	2.895733	2.664205	-3.356173
H	4.606852	3.370099	-2.249287
H	1.150510	1.736448	-4.221592
H	2.982510	3.417696	-4.133957
H	-3.099139	0.434494	-0.545694
C	-3.789601	0.378005	1.460416
C	-5.001950	1.035302	1.203104
C	-3.309206	0.335620	2.774203
C	-5.733420	1.615779	2.237365
H	-5.381464	1.087097	0.185362
C	-4.040862	0.922834	3.810619
H	-2.341458	-0.102886	2.989650
C	-5.255167	1.557164	3.548829
H	-6.671926	2.117793	2.019413
H	-3.645782	0.892850	4.821864
H	-5.820295	2.013407	4.356704

[1]**TS10**-*R*'

B3LYP/BSI SCF energy: -2922.079057a.u.

M06/BSII SCF energy in solution: -2920.7621011a.u.

M06/BSII free energy in solution: -2919.800417a.u.

C	1.186511	-2.108130	-0.739436
N	1.405960	-0.994988	-0.138512
O	2.272572	-2.773449	-1.206860
C	3.466050	-2.124921	-0.649320
C	2.858972	-0.730623	-0.178327
C	-1.334920	-2.096459	-0.469761
N	-1.377668	-1.064885	0.288146
O	-2.521075	-2.662828	-0.805085
C	-3.562218	-1.670538	-0.440017
C	-2.801150	-0.830396	0.650307
Ni	0.097300	0.176796	0.735294
C	-0.126764	-2.798691	-1.055970
C	-0.282910	-2.847781	-2.602862
H	0.557432	-3.388221	-3.043754
H	-0.317483	-1.839142	-3.024622
H	-1.212693	-3.356790	-2.861688
C	-0.088187	-4.245825	-0.490724
H	-1.027373	-4.752923	-0.720860
H	0.051505	-4.239052	0.594068
H	0.733702	-4.802851	-0.943591
C	-4.799912	-2.403559	0.047245
C	-5.928613	-1.650159	0.401475
C	-4.856884	-3.796897	0.146969
C	-7.083924	-2.278665	0.859724
H	-5.902222	-0.567746	0.314130
C	-6.019922	-4.426499	0.599038
H	-3.992499	-4.388398	-0.131447
C	-7.134688	-3.671561	0.959780
H	-7.947065	-1.679895	1.135827
H	-6.049780	-5.510415	0.667835
H	-8.037282	-4.161600	1.313223
C	-3.844510	-0.894549	-1.738529

C	-3.438388	0.428237	-1.958057
C	-4.481166	-1.584610	-2.784692
C	-3.666849	1.041691	-3.195502
H	-2.955475	1.011231	-1.181448
C	-4.704913	-0.973502	-4.015090
H	-4.805514	-2.608627	-2.626911
C	-4.296758	0.346713	-4.225309
H	-3.347905	2.069795	-3.339226
H	-5.201980	-1.525569	-4.807992
H	-4.473950	0.828186	-5.183015
C	3.935981	-2.993908	0.527385
C	4.562032	-2.457207	1.659586
C	3.797556	-4.387505	0.440946
C	5.020812	-3.287636	2.685788
H	4.707904	-1.386240	1.757831
C	4.248329	-5.215938	1.466784
H	3.331799	-4.820161	-0.437245
C	4.860850	-4.669034	2.596394
H	5.499088	-2.847317	3.555774
H	4.123941	-6.291700	1.381322
H	5.211760	-5.314025	3.396512
C	4.517215	-2.054183	-1.755228
C	4.137080	-2.045218	-3.103941
C	5.880102	-1.966945	-1.442129
C	5.096210	-1.951581	-4.112353
H	3.088827	-2.121362	-3.366909
C	6.837851	-1.864123	-2.451192
H	6.201274	-1.991724	-0.406395
C	6.450712	-1.858016	-3.791275
H	4.780840	-1.952379	-5.151874
H	7.889136	-1.798137	-2.185962
H	7.196678	-1.785228	-4.577287
C	-0.767124	3.548578	1.982864
C	-1.853610	3.318192	1.028572
C	-0.432520	4.829820	1.193161
H	0.563375	4.876196	0.736798
H	-0.648798	5.781002	1.699760
N	-1.489801	4.429383	0.244147
O	-2.726986	2.464422	0.840447
C	-1.958190	5.004718	-0.924699
C	-1.306665	6.122855	-1.478599
C	-3.108486	4.493876	-1.563254
C	-1.789794	6.706622	-2.648130
H	-0.425716	6.527435	-0.989483
C	-3.575038	5.093203	-2.729947
H	-3.619362	3.647127	-1.120923
C	-2.923572	6.199019	-3.286027
H	-1.273081	7.568747	-3.061959
H	-4.467699	4.694514	-3.205653
H	-3.297130	6.659928	-4.195343
C	-0.939388	3.479174	3.473347
H	-1.670278	4.213452	3.844638
H	-0.001039	3.662906	4.007830
H	-1.300806	2.487194	3.769256
H	3.180019	-0.528124	0.842763
C	3.196018	0.490200	-1.017405
C	4.320757	1.255240	-0.684293
C	2.420246	0.878284	-2.115937
C	4.670355	2.377718	-1.435694
H	4.928363	0.974921	0.172863
C	2.764149	2.003495	-2.866260
H	1.528968	0.315602	-2.372305
C	3.891166	2.755418	-2.530455
H	5.545939	2.959173	-1.161413

H	2.145090	2.296265	-3.709387
H	4.156175	3.632892	-3.113120
H	-3.008193	0.231393	0.538858
C	-3.040448	-1.178003	2.111118
C	-3.518665	-0.179477	2.969090
C	-2.766157	-2.448447	2.635641
C	-3.734577	-0.451529	4.322376
H	-3.691238	0.817701	2.573425
C	-2.980690	-2.719715	3.985943
H	-2.388577	-3.233359	1.987388
C	-3.468720	-1.721753	4.833610
H	-4.104429	0.333285	4.976427
H	-2.768102	-3.710734	4.377475
H	-3.635380	-1.934187	5.886016
C	0.608024	1.952208	1.414309
H	0.717162	2.448033	0.452108
H	-0.367679	1.420122	1.724806
C	1.738018	2.067978	2.423249
H	2.451043	1.244150	2.262363
H	1.348261	1.903634	3.435925
C	2.544935	3.377079	2.391260
H	1.903598	4.222396	2.667396
H	2.879066	3.565377	1.361300
C	3.767281	3.348280	3.318179
H	3.440618	3.146236	4.348842
H	4.415074	2.504262	3.037618
C	4.590755	4.642365	3.295483
H	3.947844	5.484885	3.584559
H	4.911928	4.847705	2.265023
C	5.816534	4.597524	4.213723
H	5.524271	4.427444	5.256383
H	6.382336	5.534418	4.176075
H	6.496680	3.786849	3.927078

[3]**TS10**-*S'*

B3LYP/BSI SCF energy: -2922.074063a.u.

M06/BSII SCF energy in solution: -2920.756264a.u.

M06/BSII free energy in solution: -2919.793954a.u.

C	2.126668	-1.710040	-0.303412
N	1.875241	-0.451539	-0.214619
O	3.411265	-2.087838	-0.094537
C	4.110634	-0.940001	0.498159
C	3.150933	0.247124	0.056260
C	-0.253033	-2.524133	-0.392768
N	-0.749218	-1.387301	-0.085120
O	-1.116765	-3.565128	-0.491769
C	-2.473502	-2.966820	-0.492944
C	-2.193664	-1.583421	0.198863
Ni	0.110813	0.367502	-0.381370
C	1.194957	-2.845057	-0.705017
C	1.324971	-3.054370	-2.243111
H	2.355008	-3.318838	-2.498236
H	1.042440	-2.147197	-2.783370
H	0.665012	-3.865227	-2.560177
C	1.594533	-4.148276	0.029721
H	0.917000	-4.952586	-0.260950
H	1.543040	-4.023447	1.114994
H	2.614390	-4.430242	-0.236081
C	-3.425072	-3.900950	0.238893
C	-4.777805	-3.543944	0.334375
C	-3.001478	-5.108320	0.802929
C	-5.683318	-4.371879	0.993419
H	-5.124110	-2.616656	-0.112509

C	-3.913141	-5.941090	1.457175
H	-1.959362	-5.396826	0.731106
C	-5.254597	-5.576186	1.557445
H	-6.726426	-4.077293	1.063221
H	-3.568756	-6.876958	1.888556
H	-5.962065	-6.223458	2.067583
C	-2.880904	-2.840041	-1.972518
C	-3.441594	-1.683637	-2.529987
C	-2.708961	-3.969269	-2.791157
C	-3.810477	-1.663318	-3.880379
H	-3.598598	-0.781457	-1.946198
C	-3.071145	-3.941925	-4.134847
H	-2.289234	-4.875017	-2.364704
C	-3.625776	-2.783617	-4.686656
H	-4.241430	-0.756581	-4.294946
H	-2.927705	-4.826180	-4.749727
H	-3.913217	-2.759301	-5.733992
C	4.114870	-1.161144	2.019696
C	4.071405	-0.103477	2.936869
C	4.232464	-2.468532	2.515519
C	4.126586	-0.345728	4.312126
H	3.999506	0.924540	2.596421
C	4.281437	-2.711227	3.886946
H	4.282613	-3.297537	1.818839
C	4.226575	-1.649835	4.792754
H	4.085450	0.490576	5.003744
H	4.366007	-3.732237	4.248098
H	4.263130	-1.838217	5.861607
C	5.526045	-0.908415	-0.072051
C	5.816228	-1.508138	-1.304708
C	6.556769	-0.253969	0.615434
C	7.104576	-1.451680	-1.836343
H	5.034569	-2.029208	-1.844317
C	7.842525	-0.190001	0.078293
H	6.361848	0.197907	1.582100
C	8.122781	-0.789859	-1.149671
H	7.310710	-1.928103	-2.790590
H	8.627033	0.322335	0.627759
H	9.125462	-0.746461	-1.564783
C	-2.054346	2.809010	-1.992716
C	-3.150177	2.402459	-1.125932
C	-2.450007	4.267737	-1.725551
H	-2.918926	4.798160	-2.567069
H	-1.694414	4.918124	-1.266675
N	-3.438205	3.737831	-0.762590
O	-3.658505	1.343194	-0.737033
C	-4.384751	4.363296	0.032628
C	-4.420384	5.767627	0.119231
C	-5.328681	3.605557	0.756278
C	-5.373771	6.395660	0.918106
H	-3.700214	6.358511	-0.438750
C	-6.272884	4.252574	1.547669
H	-5.306776	2.525791	0.671536
C	-6.305932	5.647601	1.639922
H	-5.385876	7.481096	0.974500
H	-6.996328	3.656658	2.098425
H	-7.046801	6.141862	2.261002
C	-1.735134	2.218481	-3.331465
H	-0.824707	2.657209	-3.760577
H	-2.541617	2.361831	-4.066992
H	-1.566425	1.138314	-3.242014
H	2.988834	0.916752	0.899551
C	3.611939	1.086238	-1.123977
C	4.387409	2.227474	-0.882390

C	3.301664	0.752587	-2.446943
C	4.852361	3.013242	-1.936963
H	4.629902	2.506193	0.140222
C	3.760636	1.540054	-3.503982
H	2.679588	-0.111414	-2.653012
C	4.539257	2.670806	-3.253296
H	5.451151	3.895331	-1.729322
H	3.504587	1.270606	-4.524789
H	4.894131	3.284021	-4.076524
H	-2.740127	-0.782029	-0.291951
C	-2.455423	-1.462567	1.691972
C	-3.306514	-0.446699	2.144350
C	-1.841735	-2.303324	2.630974
C	-3.549630	-0.283254	3.510535
H	-3.754320	0.229025	1.420814
C	-2.085923	-2.141076	3.993496
H	-1.175613	-3.094006	2.299156
C	-2.943166	-1.130567	4.437479
H	-4.207905	0.512858	3.846893
H	-1.607533	-2.803456	4.709751
H	-3.131343	-1.003727	5.500035
C	-0.182543	2.181946	-0.818036
H	0.256238	2.462654	-1.770856
H	-1.048986	1.366897	-0.744560
C	-0.101405	3.209258	0.300794
H	0.102161	2.702955	1.254881
H	-1.065193	3.715953	0.451688
C	0.986101	4.263664	0.047660
H	0.759143	4.797304	-0.886379
H	1.947258	3.760006	-0.122194
C	1.133293	5.276426	1.189935
H	0.170114	5.779138	1.358482
H	1.359326	4.741195	2.123937
C	2.217423	6.332199	0.938765
H	1.989872	6.869108	0.007642
H	3.179527	5.829177	0.768387
C	2.362158	7.338344	2.085215
H	1.424998	7.880298	2.256090
H	3.140871	8.079286	1.875026
H	2.625675	6.835713	3.023153

3-*S*

B3LYP/BSI SCF energy: -753.564757a.u.

M06/BSII SCF energy in solution: -753.190053a.u.

M06/BSII free energy in solution: -752.87812a.u.

C	0.067212	1.157324	0.502841
C	-1.219263	1.396007	-0.324184
C	-0.694154	-0.089354	1.070341
H	-0.921326	-0.053663	2.141654
H	-0.257116	-1.062311	0.818772
N	-1.834255	0.278146	0.216412
O	-1.587391	2.218898	-1.135436
C	-3.087229	-0.320463	0.039093
C	-3.412900	-1.481336	0.755528
C	-4.018516	0.238090	-0.853950
C	-4.660993	-2.078137	0.579077
H	-2.694100	-1.913203	1.445257
C	-5.259295	-0.372491	-1.016448
H	-3.753582	1.135247	-1.400605
C	-5.590260	-1.529753	-0.305558
H	-4.904562	-2.977132	1.138287
H	-5.975495	0.062895	-1.707666
H	-6.560771	-1.997438	-0.440146

C	0.330804	2.271878	1.518839
H	0.637458	3.187867	1.003958
H	1.120052	1.995251	2.224369
H	-0.571668	2.499685	2.095407
C	1.277610	0.828592	-0.387886
H	0.976055	0.062210	-1.115062
H	1.518958	1.723684	-0.975552
C	2.529682	0.345288	0.358154
H	2.271353	-0.511288	0.997402
H	2.890377	1.131590	1.033322
C	3.667807	-0.058783	-0.588812
H	3.919769	0.793130	-1.236069
H	3.316342	-0.854837	-1.260537
C	4.930813	-0.531732	0.141793
H	5.282882	0.265462	0.812378
H	4.678427	-1.382581	0.791080
C	6.070222	-0.936910	-0.801859
H	6.320953	-0.087044	-1.451167
H	5.719596	-1.734977	-1.470424
C	7.328729	-1.405136	-0.064258
H	7.724989	-0.615759	0.584817
H	8.121397	-1.687958	-0.764590
H	7.117303	-2.275541	0.567455

3-*R*

B3LYP/BSI SCF energy: -753.564757a.u.

M06/BSII SCF energy in solution: -753.190053a.u.

M06/BSII free energy in solution: -752.87812a.u.

C	0.06721200	1.15732400	-0.50284100
C	-1.21926300	1.39600700	0.32418400
C	-0.69415400	-0.08935400	-1.0703410
H	-0.92132600	-0.05366300	-2.1416540
H	-0.25711600	-1.06231100	-0.8187720
N	-1.83425500	0.27814600	-0.2164120
O	-1.58739100	2.21889800	1.13543600
C	-3.08722900	-0.32046300	-0.0390930
C	-3.41290000	-1.48133600	-0.7555280
C	-4.01851600	0.23809000	0.85395000
C	-4.66099300	-2.07813700	-0.5790770
H	-2.69410000	-1.91320300	-1.4452570
C	-5.25929500	-0.37249100	1.01644800
H	-3.75358200	1.13524700	1.40060500
C	-5.59026000	-1.52975300	0.30555800
H	-4.90456200	-2.97713200	-1.1382870
H	-5.97549500	0.06289500	1.70766600
H	-6.56077100	-1.99743800	0.4401460
C	0.33080400	2.27187800	-1.51883900
H	0.63745800	3.18786700	-1.00395800
H	1.12005200	1.99525100	-2.22436900
H	-0.57166800	2.49968500	-2.0954070
C	1.27761000	0.82859200	0.38788600
H	0.97605500	0.06221000	1.11506200
H	1.51895800	1.72368400	0.97555200
C	2.52968200	0.34528800	-0.35815400
H	2.27135300	-0.51128800	-0.9974020
H	2.89037700	1.13159000	-1.03332200
C	3.66780700	-0.05878300	0.58881200
H	3.91976900	0.79313000	1.23606900
H	3.31634200	-0.85483700	1.26053700
C	4.93081300	-0.53173200	-0.14179300
H	5.28288200	0.26546200	-0.81237800
H	4.67842700	-1.38258100	-0.7910800
C	6.07022200	-0.93691000	0.80185900

H 6.32095300 -0.08704400 1.45116700

H 5.71959600 -1.73497700 1.47042400

C 7.32872900 -1.40513600 0.06425800

H 7.72498900 -0.61575900 -0.5848170

H 8.12139700 -1.68795800 0.76459000

H 7.11730300 -2.27554100 -0.5674550

[1]**TS11**-*R*

B3LYP/BSI SCF energy: -2699.09813a.u.

M06/BSII SCF energy in solution: -2697.8970785a.u.

M06/BSII free energy in solution: -2697.102795a.u.

C -1.414220 -1.298553 1.055741

N -1.509626 -0.626057 -0.030284

O -2.566774 -1.652018 1.663351

C -3.653949 -1.396979 0.683748

C -2.944991 -0.367106 -0.282956

H -3.155010 -0.620684 -1.321042

C -3.279905 1.103654 -0.100763

C -4.099969 1.735253 -1.043843

C -2.793141 1.852566 0.978292

C -4.445075 3.080533 -0.902360

H -4.462185 1.171840 -1.899377

C -3.131057 3.198044 1.119304

H -2.138007 1.387616 1.708338

C -3.963437 3.814615 0.182204

H -5.083660 3.553547 -1.643031

H -2.739459 3.767277 1.957063

H -4.227075 4.862416 0.294184

C 1.123288 -1.291316 1.137729

N 1.330579 -0.633141 0.050934

O 2.209800 -1.616730 1.867243

C 3.333939 -0.801279 1.358330

C 2.799678 -0.438072 -0.083911

H 2.988243 0.611366 -0.301762

C 3.366977 -1.240745 -1.243092

C 4.374724 -0.664117 -2.027871

C 2.923407 -2.532170 -1.553528

C 4.939791 -1.370774 -3.090541

H 4.697835 0.351050 -1.814453

C 3.481701 -3.235963 -2.621165

H 2.127352 -2.987420 -0.972858

C 4.494448 -2.659431 -3.389630

H 5.719460 -0.909687 -3.690215

H 3.120907 -4.233378 -2.855187

H 4.926619 -3.207618 -4.221803

Ni -0.026083 -0.319648 -1.368969

C -0.168835 -1.801602 1.752568

C -0.164674 -3.356090 1.673805

H -1.075844 -3.748550 2.129207

H -0.123753 -3.694407 0.634737

H 0.700901 -3.749675 2.211090

C -0.223134 -1.354996 3.239843

H 0.641562 -1.746264 3.776513

H -0.212635 -0.264312 3.323707

H -1.135603 -1.734779 3.702402

C 3.427035 0.381839 2.336471

C 3.720112 0.077344 3.678368

C 3.191320 1.715817 1.988091

C 3.770412 1.076344 4.645119

H 3.911814 -0.953611 3.959919

C 3.243490 2.719344 2.963394

H 2.979549 2.009665 0.966750

C 3.529731 2.407282 4.289378

H	4.000191	0.818342	5.675127
H	3.050783	3.747428	2.670798
H	3.569043	3.190197	5.041484
C	4.600514	-1.643770	1.384054
C	5.843203	-1.010080	1.245240
C	4.561297	-3.035349	1.519204
C	7.020818	-1.755231	1.232402
H	5.888905	0.071139	1.155936
C	5.742954	-3.779626	1.517901
H	3.606769	-3.535823	1.632323
C	6.975435	-3.144120	1.371746
H	7.975050	-1.248560	1.120772
H	5.695931	-4.859086	1.630666
H	7.893662	-3.724223	1.369152
C	-4.863186	-0.849607	1.423078
C	-6.070646	-0.699243	0.725512
C	-4.809060	-0.475780	2.769324
C	-7.195683	-0.176384	1.359276
H	-6.131692	-1.001991	-0.315682
C	-5.940654	0.040271	3.406104
H	-3.884244	-0.593914	3.321557
C	-7.135353	0.194678	2.704727
H	-8.121619	-0.063991	0.802772
H	-5.883428	0.320838	4.454050
H	-8.013763	0.597093	3.200793
C	-3.933261	-2.762455	0.036635
C	-4.686255	-3.702938	0.760180
C	-3.389099	-3.146622	-1.197590
C	-4.901028	-4.985234	0.260868
H	-5.109698	-3.423712	1.719550
C	-3.609669	-4.433828	-1.697960
H	-2.787782	-2.466573	-1.792240
C	-4.364956	-5.355143	-0.975201
H	-5.490748	-5.694035	0.835387
H	-3.184685	-4.705652	-2.659711
H	-4.537051	-6.352607	-1.369663
C	1.209780	1.961092	-2.842149
C	1.893124	2.674037	-1.753828
C	0.035325	2.929294	-2.701148
H	-0.109821	3.614029	-3.548082
H	-0.921647	2.496284	-2.400518
N	0.807690	3.543855	-1.590030
O	2.980729	2.626434	-1.179800
C	0.603281	4.702803	-0.851640
C	-0.633313	5.367214	-0.921366
C	1.632142	5.226671	-0.043035
C	-0.835544	6.535636	-0.189802
H	-1.430364	4.958926	-1.533842
C	1.408218	6.394341	0.681059
H	2.588270	4.718132	-0.012820
C	0.178690	7.056939	0.616106
H	-1.795307	7.041191	-0.252057
H	2.208435	6.796615	1.296573
H	0.015839	7.968806	1.182497
C	1.782631	1.319297	-4.052190
H	2.660044	0.719740	-3.796681
H	1.048931	0.680489	-4.548679
H	2.105288	2.090183	-4.772553
Br	-1.210592	-0.592269	-3.449201

^{3}**TS11**-*R*

B3LYP/BSI SCF energy: -2699.099879a.u.

M06/BSII SCF energy in solution: -2697.895771a.u.

M06/BSII free energy in solution: -2697.103554a.u.

C	-1.455556	-1.233746	1.106394
N	-1.532824	-0.619226	-0.015559
O	-2.616773	-1.514163	1.732364
C	-3.700685	-1.274965	0.743694
C	-2.957456	-0.334230	-0.289365
H	-3.172852	-0.653624	-1.308428
C	-3.251700	1.154319	-0.212512
C	-4.072546	1.734631	-1.187539
C	-2.728780	1.968536	0.800125
C	-4.384593	3.094398	-1.140863
H	-4.464349	1.118691	-1.992582
C	-3.034018	3.328471	0.846560
H	-2.069723	1.543932	1.550956
C	-3.868878	3.894096	-0.120132
H	-5.024419	3.526878	-1.904804
H	-2.613780	3.948535	1.632618
H	-4.108607	4.952884	-0.080788
C	1.081876	-1.289277	1.194004
N	1.299178	-0.728661	0.057423
O	2.158288	-1.547126	1.960985
C	3.300298	-0.802803	1.383108
C	2.764232	-0.513243	-0.078160
H	2.929123	0.531228	-0.336748
C	3.342262	-1.342067	-1.212541
C	4.219302	-0.727395	-2.116013
C	3.018001	-2.692774	-1.393374
C	4.775295	-1.455150	-3.170056
H	4.441882	0.330201	-2.002509
C	3.567962	-3.417898	-2.449872
H	2.326048	-3.181248	-0.714058
C	4.451707	-2.802032	-3.338693
H	5.452779	-0.965740	-3.864073
H	3.302749	-4.462880	-2.581635
H	4.877422	-3.367352	-4.162698
Ni	-0.057122	-0.584719	-1.394806
C	-0.223741	-1.751193	1.821714
C	-0.261611	-3.306665	1.777089
H	-1.181882	-3.665281	2.242395
H	-0.229750	-3.667704	0.745459
H	0.593676	-3.710967	2.323420
C	-0.270137	-1.269019	3.297071
H	0.581377	-1.671785	3.846054
H	-0.228333	-0.177441	3.356432
H	-1.194439	-1.611702	3.764823
C	3.444976	0.434454	2.285419
C	3.786763	0.204683	3.630715
C	3.199744	1.747999	1.871943
C	3.874460	1.255639	4.537671
H	3.986770	-0.809351	3.963005
C	3.289525	2.804061	2.787130
H	2.954388	1.987307	0.843652
C	3.623159	2.565376	4.117251
H	4.141244	1.054254	5.571383
H	3.086326	3.814408	2.444846
H	3.690939	3.388968	4.822441
C	4.541430	-1.680450	1.450923
C	5.787947	-1.111576	1.153247
C	4.478919	-3.034396	1.793741
C	6.945241	-1.886304	1.185071
H	5.851755	-0.056827	0.902762
C	5.641963	-3.807622	1.835683
H	3.522188	-3.482387	2.035427
C	6.876911	-3.238815	1.528277

H	7.902312	-1.431213	0.947255
H	5.577757	-4.856855	2.109930
H	7.780146	-3.841188	1.558983
C	-4.879584	-0.632455	1.455350
C	-6.092177	-0.491014	0.764953
C	-4.789759	-0.159383	2.768023
C	-7.186879	0.120222	1.372335
H	-6.181419	-0.870250	-0.248759
C	-5.891156	0.445244	3.379092
H	-3.860690	-0.269186	3.314759
C	-7.090874	0.590413	2.684289
H	-8.117331	0.224110	0.821743
H	-5.806392	0.802159	4.401611
H	-7.945913	1.061836	3.159961
C	-4.040297	-2.664814	0.184434
C	-4.825458	-3.526014	0.970095
C	-3.523030	-3.148436	-1.026256
C	-5.100285	-4.825833	0.552436
H	-5.226765	-3.170810	1.913742
C	-3.803918	-4.452966	-1.444754
H	-2.895211	-2.536572	-1.666025
C	-4.592707	-5.293863	-0.662257
H	-5.714274	-5.471578	1.173918
H	-3.397997	-4.801741	-2.389727
H	-4.811450	-6.305089	-0.993430
C	1.157957	2.081949	-3.006718
C	1.902898	2.685594	-1.907141
C	0.028319	3.077857	-2.770819
H	-0.131473	3.813864	-3.571274
H	-0.930662	2.660080	-2.449976
N	0.863783	3.602779	-1.660264
O	2.996270	2.544788	-1.356712
C	0.750660	4.745461	-0.882288
C	-0.450373	5.477052	-0.878388
C	1.839978	5.193048	-0.106413
C	-0.558175	6.632197	-0.106727
H	-1.293267	5.130367	-1.467028
C	1.710174	6.349186	0.657925
H	2.768833	4.636015	-0.134032
C	0.515767	7.076318	0.667511
H	-1.491013	7.189530	-0.113298
H	2.557672	6.692291	1.245499
H	0.426285	7.978347	1.265055
C	1.597586	1.345798	-4.213373
H	2.446251	0.696363	-3.981664
H	0.791945	0.730857	-4.622137
H	1.920879	2.049432	-4.999215
Br	-1.137663	-1.004215	-3.486202

1·

B3LYP/BSI SCF energy: -517.010513a.u.

M06/BSII SCF energy in solution: -516.781316a.u.

M06/BSII free energy in solution: -516.643155a.u.

C	2.625412	-0.312418	0.000298
C	1.631550	0.765468	0.000408
C	1.512158	-1.344678	0.000439
H	1.434459	-1.977107	-0.895013
H	1.434856	-1.977188	0.895892
N	0.586453	-0.190791	0.000745
O	1.616977	1.983189	0.000179
C	-0.798843	-0.119214	0.000321
C	-1.566099	-1.297138	0.000151
C	-1.440974	1.135348	0.000117

C -2.957296 -1.218981 -0.000254
H -1.072722 -2.264373 0.000373
C -2.830994 1.190116 -0.000316
H -0.837656 2.035473 0.000262
C -3.598763 0.020946 -0.000507
H -3.541355 -2.134992 -0.000381
H -3.320930 2.159776 -0.000492
H -4.682928 0.076881 -0.000839
C 4.099722 -0.368131 -0.001031
H 4.514375 0.643487 -0.001570
H 4.488681 -0.899773 0.879409
H 4.486998 -0.900063 -0.882041

1˙

B3LYP-D3BJ/BSI SCF energy in solution:

-517.066973a.u.

M06/BSII SCF energy in solution: -516.78143a.u.

M06/BSII free energy in solution: -516.643758a.u.

C 2.622938 -0.307474 -0.000045
C 1.628945 0.765914 -0.000036
C 1.513077 -1.341615 0.000049
H 1.434838 -1.968243 -0.896740
H 1.434922 -1.968187 0.896885
N 0.586247 -0.184745 0.000054
O 1.609403 1.989031 -0.000067
C -0.794762 -0.114830 0.000031
C -1.556025 -1.296979 -0.000023
C -1.443235 1.136116 0.000065
C -2.947213 -1.224310 -0.000054
H -1.056906 -2.260638 -0.000047
C -2.833819 1.185964 0.000032
H -0.845888 2.039866 0.000112
C -3.595749 0.012903 -0.000024
H -3.526551 -2.143153 -0.000098
H -3.327973 2.153385 0.000057
H -4.680085 0.063332 -0.000045
C 4.093047 -0.372777 0.000003
H 4.524327 0.632101 -0.000087
H 4.470551 -0.912403 0.880482
H 4.470593 -0.912572 -0.880351

^{2}[Ni^I]Br

B3LYP/BSI SCF energy: -2182.075156a.u.

M06/BSII SCF energy in solution: -2181.101356a.u.

M06/BSII free energy in solution: -2180.472837a.u.

C 1.238082 0.296623 -0.994245
N 1.376858 -0.020972 0.238891
O 2.369327 0.413221 -1.727589
C 3.451308 -0.195615 -0.907611
C 2.828350 -0.074125 0.534094
H 3.028505 -0.977333 1.109303
C 3.247883 1.110345 1.388468
C 3.926771 0.875759 2.589200
C 2.957032 2.431964 1.024331
C 4.320694 1.937985 3.404901
H 4.128923 -0.145869 2.898423
C 3.348441 3.494259 1.837385
H 2.423583 2.636120 0.101001
C 4.034063 3.250206 3.029869
H 4.840022 1.736431 4.337190
H 3.116991 4.513490 1.540950
H 4.335277 4.078464 3.664882

C	-1.285746	0.457820	-0.903876
N	-1.426848	-0.052339	0.263896
O	-2.407116	0.928626	-1.501016
C	-3.479532	0.925665	-0.499789
C	-2.868015	-0.020991	0.615200
H	-2.949371	0.463805	1.587668
C	-3.420729	-1.427788	0.746129
C	-4.007665	-1.824339	1.952238
C	-3.332863	-2.355566	-0.300899
C	-4.506048	-3.118767	2.111982
H	-4.059794	-1.122485	2.780918
C	-3.830584	-3.647123	-0.144783
H	-2.877997	-2.068638	-1.243964
C	-4.419429	-4.032382	1.062552
H	-4.948957	-3.412914	3.058772
H	-3.756315	-4.355843	-0.964522
H	-4.800861	-5.041865	1.184864
Ni	-0.021387	-0.782587	1.441052
C	-0.041810	0.537080	-1.771414
C	-0.157307	-0.561872	-2.868281
H	0.720145	-0.526862	-3.516921
H	-0.216330	-1.555988	-2.417134
H	-1.052622	-0.391038	-3.470925
C	0.041959	1.934778	-2.439248
H	-0.831449	2.098168	-3.072434
H	0.077930	2.729557	-1.688482
H	0.940661	1.993041	-3.055706
C	-3.651542	2.375135	-0.013684
C	-3.412238	3.432437	-0.903275
C	-4.112144	2.678006	1.274105
C	-3.607689	4.754970	-0.508645
H	-3.068938	3.215167	-1.908227
C	-4.313905	4.002760	1.667684
H	-4.322003	1.888146	1.988568
C	-4.058434	5.046692	0.780048
H	-3.409642	5.558917	-1.211866
H	-4.665875	4.213466	2.673221
H	-4.209838	6.076913	1.088202
C	-4.761966	0.441162	-1.174535
C	-5.947478	0.353533	-0.431339
C	-4.791692	0.102556	-2.531517
C	-7.130173	-0.076494	-1.028417
H	-5.951624	0.622218	0.619918
C	-5.980131	-0.322181	-3.130812
H	-3.886136	0.172289	-3.121796
C	-7.152300	-0.415870	-2.383100
H	-8.036471	-0.144141	-0.433808
H	-5.984048	-0.579343	-4.186302
H	-8.075403	-0.747790	-2.849059
C	4.735913	0.585012	-1.120394
C	5.906560	0.132354	-0.494382
C	4.792727	1.735541	-1.912494
C	7.105284	0.824727	-0.647864
H	5.879653	-0.769202	0.110798
C	5.998061	2.424567	-2.071998
H	3.895703	2.090270	-2.406180
C	7.155857	1.974942	-1.439472
H	8.001369	0.463389	-0.151851
H	6.027137	3.315141	-2.693497
H	8.091403	2.512634	-1.562816
C	3.566726	-1.646286	-1.402828
C	4.235919	-1.888477	-2.614182
C	2.959143	-2.727690	-0.747344
C	4.306501	-3.172048	-3.150254

H 4.709224 -1.063244 -3.136445
C 3.035138 -4.016174 -1.284882
H 2.416020 -2.596086 0.182523
C 3.707906 -4.244048 -2.483816
H 4.834211 -3.335507 -4.085654
H 2.563637 -4.838416 -0.754836
H 3.768265 -5.247351 -2.895810
Br 0.707735 -2.110318 3.245260

[1]**TS11**-*S*

B3LYP/BSI SCF energy: -2699.100631a.u.

M06/BSII SCF energy in solution: -2697.8994064a.u.

M06/BSII free energy in solution: -2697.105244a.u.

C 0.386534 2.096013 -0.322187
N 0.898927 0.951004 -0.049598
O 1.253431 3.111725 -0.534858
C 2.594965 2.512152 -0.673702
C 2.377148 1.121973 0.033111
C -2.048633 1.390347 -0.181786
N -1.814528 0.137897 -0.040332
O -3.341482 1.781217 -0.127652
C -4.100237 0.625491 0.419661
C -3.120570 -0.557606 0.072373
Ni -0.051291 -0.698546 0.476179
C -1.067927 2.521269 -0.417971
C -1.319207 3.628738 0.643816
H -0.646987 4.469967 0.465874
H -1.148966 3.247010 1.654589
H -2.351715 3.974441 0.577700
C -1.327597 3.091510 -1.841313
H -2.361063 3.436513 -1.916573
H -1.152184 2.329786 -2.606400
H -0.657033 3.930911 -2.031709
C -5.457332 0.558333 -0.258411
C -6.361998 -0.434285 0.146329
C -5.837091 1.450752 -1.265248
C -7.613841 -0.539565 -0.455133
H -6.087453 -1.122442 0.940686
C -7.096305 1.348206 -1.862958
H -5.150250 2.228388 -1.577918
C -7.986501 0.352799 -1.463727
H -8.300558 -1.316913 -0.133047
H -7.377949 2.051490 -2.641591
H -8.964184 0.273155 -1.929935
C -4.218587 0.896040 1.928310
C -3.339277 0.340981 2.870762
C -5.189741 1.808219 2.373193
C -3.443862 0.681675 4.222709
H -2.562245 -0.359800 2.582937
C -5.289248 2.148397 3.720316
H -5.875329 2.250150 1.657480
C -4.416698 1.582249 4.652782
H -2.757251 0.232557 4.934146
H -6.051951 2.852020 4.041862
H -4.497322 1.840549 5.704840
C 2.864664 2.420441 -2.186701
C 3.408518 1.292326 -2.812590
C 2.594216 3.559271 -2.964612
C 3.662589 1.306143 -4.189576
H 3.635980 0.386321 -2.260463
C 2.841930 3.565973 -4.334221
H 2.191607 4.446713 -2.486781
C 3.379593 2.434596 -4.954626

H	4.083041	0.420276	-4.656915
H	2.623006	4.456668	-4.916546
H	3.578166	2.437894	-6.022581
C	3.615886	3.435146	-0.020353
C	3.243921	4.615103	0.630926
C	4.975013	3.098107	-0.091609
C	4.213232	5.441409	1.205479
H	2.197252	4.889556	0.688857
C	5.938874	3.918437	0.489588
H	5.281207	2.193564	-0.608617
C	5.561874	5.096119	1.139967
H	3.907850	6.356227	1.705661
H	6.986815	3.639253	0.430250
H	6.314427	5.737927	1.588655
C	1.202710	-2.354680	-1.703447
C	2.590110	-2.404251	-1.228625
C	1.025720	-3.806049	-1.270728
H	0.893400	-4.528533	-2.088648
H	0.302536	-3.982972	-0.470076
N	2.430170	-3.723855	-0.798905
O	3.572913	-1.659847	-1.196551
C	3.255655	-4.657770	-0.187643
C	2.738722	-5.913474	0.175516
C	4.610309	-4.360955	0.059974
C	3.566258	-6.854599	0.783333
H	1.693194	-6.139168	-0.007817
C	5.419623	-5.315380	0.668807
H	4.999433	-3.393398	-0.232858
C	4.909083	-6.564609	1.034055
H	3.155154	-7.819778	1.065328
H	6.463592	-5.080407	0.857720
H	5.549589	-7.301578	1.508881

C	0.546707	-1.571927	-2.778376
H	0.954984	-0.559094	-2.828322
H	-0.535950	-1.509250	-2.633282
H	0.713202	-2.045114	-3.761173
H	2.838375	0.320075	-0.539061
C	2.842939	0.982181	1.473807
C	3.809927	0.017799	1.781845
C	2.311952	1.766079	2.508085
C	4.246481	-0.152413	3.097722
H	4.199983	-0.615059	0.989915
C	2.747632	1.596458	3.820416
H	1.556352	2.515092	2.291047
C	3.718520	0.636732	4.118687
H	4.990551	-0.910820	3.323168
H	2.328310	2.211484	4.611662
H	4.053415	0.501238	5.143115
H	-3.062464	-1.257082	0.905234
C	-3.407903	-1.365590	-1.182177
C	-3.669635	-2.735476	-1.064080
C	-3.397985	-0.788462	-2.459915
C	-3.928292	-3.512479	-2.195316
H	-3.654109	-3.198170	-0.081327
C	-3.654962	-1.562451	-3.590963
H	-3.195260	0.271974	-2.574419
C	-3.922532	-2.927774	-3.461641
H	-4.127251	-4.574548	-2.084508
H	-3.647989	-1.099497	-4.573751
H	-4.121882	-3.530200	-4.343287
Br	-0.518882	-2.604691	1.833934

[3]**TS11**-*S*

B3LYP/BSI SCF energy: -2699.103194a.u.

M06/BSII SCF energy in solution: -2697.898958a.u.

M06/BSII free energy in solution: -2697.106517a.u.

C	0.253748	-2.141389	-0.022425
N	0.810881	-0.992462	-0.152387
O	1.074798	-3.189978	0.206084
C	2.400183	-2.632614	0.543411
C	2.285529	-1.186952	-0.073662
C	-2.148432	-1.339116	-0.131800
N	-1.862542	-0.091252	-0.066915
O	-3.457306	-1.663131	-0.211087
C	-4.180912	-0.399992	-0.519179
C	-3.135665	0.666003	-0.014159
Ni	-0.085058	0.717581	-0.575412
C	-1.208830	-2.528208	-0.160827
C	-1.382696	-3.250146	-1.529238
H	-0.736114	-4.129946	-1.566286
H	-1.123451	-2.583794	-2.356386
H	-2.420653	-3.566032	-1.649876
C	-1.600557	-3.490846	0.991890
H	-2.651178	-3.770346	0.895636
H	-1.447710	-3.021049	1.967933
H	-0.987463	-4.391737	0.947187
C	-5.507737	-0.398312	0.219841
C	-6.388505	0.674173	0.016916
C	-5.880259	-1.424998	1.092568
C	-7.610381	0.723386	0.683789
H	-6.118852	1.469234	-0.672257
C	-7.110044	-1.378100	1.754825
H	-5.211213	-2.262657	1.250496
C	-7.976785	-0.304786	1.556215
H	-8.278871	1.563210	0.518292
H	-7.387182	-2.185669	2.426557
H	-8.931511	-0.268880	2.072691
C	-4.366644	-0.401731	-2.044705
C	-3.507327	0.282821	-2.917599
C	-5.386211	-1.196893	-2.594394
C	-3.676997	0.181537	-4.301802
H	-2.695517	0.902835	-2.551469
C	-5.550942	-1.298135	-3.973759
H	-6.058156	-1.736128	-1.934586
C	-4.696565	-0.605015	-4.834649
H	-3.003256	0.726240	-4.956499
H	-6.349869	-1.914918	-4.375575
H	-4.827268	-0.677232	-5.910624
C	2.494768	-2.655274	2.080095
C	3.035060	-1.607338	2.835421
C	2.070764	-3.818339	2.744478
C	3.134265	-1.722876	4.227209
H	3.377578	-0.686379	2.374102
C	2.165340	-3.925653	4.129323
H	1.667106	-4.644102	2.167778
C	2.699652	-2.874250	4.879177
H	3.554470	-0.897941	4.795429
H	1.828732	-4.833151	4.622871
H	2.778230	-2.956618	5.959489
C	3.476048	-3.529816	-0.055509
C	3.162646	-4.663271	-0.811983
C	4.823386	-3.221153	0.179413
C	4.178551	-5.471176	-1.329335
H	2.124963	-4.916110	-0.995447
C	5.834548	-4.023062	-0.344231
H	5.082980	-2.353520	0.778360
C	5.516217	-5.153851	-1.100491

H	3.918329	-6.349717	-1.913130
H	6.873159	-3.765958	-0.157654
H	6.305223	-5.781277	-1.504711
C	1.360548	2.423724	2.023416
C	2.737239	2.272843	1.562844
C	1.350151	3.850535	1.494969
H	1.280257	4.643213	2.253249
H	0.665425	4.043564	0.663441
N	2.745668	3.582219	1.064588
O	3.616572	1.406654	1.562646
C	3.679939	4.371671	0.410823
C	3.313023	5.644764	-0.060007
C	5.000036	3.913165	0.228679
C	4.252857	6.441368	-0.709569
H	2.295113	5.996012	0.074668
C	5.922932	4.724755	-0.424123
H	5.275128	2.936843	0.608821
C	5.561036	5.989776	-0.897012
H	3.956987	7.420911	-1.074302
H	6.939435	4.365642	-0.560605
H	6.289241	6.614828	-1.404786
C	0.513966	1.701466	2.998087
H	0.857489	0.670422	3.119857
H	-0.537591	1.686659	2.693801
H	0.552270	2.185056	3.988448
H	2.684216	-0.444383	0.614116
C	2.922158	-0.945035	-1.432739
C	3.917215	0.032833	-1.549349
C	2.521985	-1.647770	-2.578144
C	4.506895	0.298806	-2.787375
H	4.213012	0.596492	-0.669219
C	3.112006	-1.383986	-3.812431
H	1.750580	-2.408619	-2.507827
C	4.107566	-0.409103	-3.920203
H	5.269624	1.068234	-2.863878
H	2.793874	-1.937176	-4.691629
H	4.562229	-0.199845	-4.884310
H	-3.067880	1.493150	-0.719868
C	-3.348042	1.262512	1.367614
C	-3.580435	2.637691	1.488097
C	-3.295280	0.483179	2.531626
C	-3.770107	3.222266	2.742172
H	-3.597752	3.256220	0.594981
C	-3.484298	1.064197	3.784812
H	-3.107738	-0.583887	2.462174
C	-3.724423	2.436411	3.893714
H	-3.946333	4.291482	2.816577
H	-3.443749	0.445845	4.677054
H	-3.869818	2.888703	4.870557
Br	-0.434244	2.810513	-1.649846

[1]**TS12**-*R*

B3LYP/BSI SCF energy: -2922.107089a.u.

M06/BSII SCF energy in solution: -2920.7905919a.u.

M06/BSII free energy in solution: -2919.82777a.u.

C	1.046470	-2.224679	-0.305186
N	1.271738	-1.130424	0.317683
O	2.066697	-2.757438	-1.014858
C	3.280612	-2.007167	-0.649756
C	2.640056	-0.696204	-0.010200
H	3.147791	-0.461862	0.924370
C	2.644060	0.559992	-0.869204
C	3.797578	1.355842	-0.908235

C	1.529608	0.953069	-1.618957
C	3.842080	2.509396	-1.689990
H	4.669002	1.071047	-0.324532
C	1.569407	2.113066	-2.395678
H	0.616871	0.369398	-1.577066
C	2.725449	2.892277	-2.436657
H	4.745962	3.111849	-1.709984
H	0.688838	2.409706	-2.957360
H	2.750606	3.798021	-3.035038
C	-1.426453	-2.088104	-0.171662
N	-1.504918	-1.034571	0.557593
O	-2.524283	-2.426735	-0.883167
C	-3.399167	-1.237613	-0.875568
C	-2.889290	-0.514546	0.435071
H	-2.848729	0.561486	0.283611
C	-3.704586	-0.767669	1.694290
C	-4.620626	0.208351	2.110534
C	-3.589548	-1.948269	2.438541
C	-5.414813	-0.001068	3.239562
H	-4.685806	1.142555	1.560117
C	-4.379715	-2.154950	3.569911
H	-2.871362	-2.707061	2.145286
C	-5.298424	-1.183704	3.971727
H	-6.119395	0.765404	3.550350
H	-4.274785	-3.074483	4.139006
H	-5.912905	-1.344659	4.853004
Ni	-0.014212	-0.341225	1.766818
C	-0.239707	-3.033354	-0.287848
C	-0.203804	-3.952210	0.969204
H	0.667119	-4.611973	0.921066
H	-0.143837	-3.354033	1.881867
H	-1.105750	-4.571018	1.000712
C	-0.354459	-3.895333	-1.562979
H	-1.266360	-4.493149	-1.522721
H	-0.393602	-3.277976	-2.463833
H	0.504630	-4.563239	-1.637072
C	-3.072276	-0.477333	-2.173396
C	-2.956205	-1.226758	-3.357006
C	-2.919322	0.912133	-2.240327
C	-2.671888	-0.606903	-4.570710
H	-3.089405	-2.303024	-3.319564
C	-2.633610	1.532321	-3.462891
H	-3.024307	1.537588	-1.359966
C	-2.504587	0.779768	-4.627828
H	-2.584130	-1.205629	-5.473120
H	-2.506298	2.610697	-3.488772
H	-2.280524	1.265700	-5.573236
C	-4.849095	-1.700658	-0.861404
C	-5.867437	-0.782726	-1.153801
C	-5.196445	-3.019522	-0.548979
C	-7.204588	-1.174913	-1.124279
H	-5.613891	0.240153	-1.414391
C	-6.535909	-3.413181	-0.530692
H	-4.417614	-3.739519	-0.327823
C	-7.544697	-2.493414	-0.815188
H	-7.980713	-0.449556	-1.350864
H	-6.787844	-4.442742	-0.292270
H	-8.586446	-2.800532	-0.799659
C	4.095686	-1.803813	-1.924360
C	5.488709	-1.666265	-1.873515
C	3.457477	-1.720907	-3.169646
C	6.224924	-1.439692	-3.037310
H	6.007576	-1.747061	-0.924517
C	4.195025	-1.503861	-4.332743

H	2.381708	-1.835170	-3.229780
C	5.581798	-1.359095	-4.272084
H	7.304498	-1.336390	-2.975535
H	3.681952	-1.447674	-5.288549
H	6.155190	-1.189741	-5.178862
C	4.028842	-2.863023	0.383004
C	3.988051	-4.261074	0.266804
C	4.805267	-2.302585	1.405189
C	4.683140	-5.073461	1.160895
H	3.404817	-4.710111	-0.529438
C	5.507705	-3.116690	2.297922
H	4.877396	-1.225992	1.520025
C	5.445257	-4.504155	2.183224
H	4.631782	-6.153494	1.055878
H	6.098081	-2.659027	3.086228
H	5.985886	-5.136298	2.881448
C	-1.482787	2.893039	2.325057
C	-2.140211	3.144706	1.050131
C	-0.313630	3.763312	1.879885
H	-0.176564	4.702981	2.433778
H	0.648698	3.257427	1.756784
N	-1.062465	3.940781	0.612929
O	-3.195160	2.848839	0.484693
C	-0.831666	4.747007	-0.489177
C	0.389775	5.433118	-0.615567
C	-1.820637	4.901531	-1.483342
C	0.617810	6.251996	-1.719318
H	1.154332	5.315663	0.145648
C	-1.571268	5.723102	-2.579240
H	-2.767870	4.389006	-1.365225
C	-0.355376	6.401939	-2.709791
H	1.565256	6.777378	-1.802853
H	-2.342297	5.842895	-3.335836
H	-0.173022	7.042910	-3.566887
C	-1.984429	2.494989	3.659380
H	-2.888937	1.889613	3.563721
H	-1.240836	1.918935	4.217634
H	-2.233273	3.381062	4.267019
C	0.799518	0.013347	3.539458
H	1.402415	-0.891299	3.737149
H	-0.058492	-0.068466	4.231178
C	1.629513	1.239068	3.954965
H	1.017489	2.151049	3.902797
H	1.937276	1.162903	5.013700
C	2.898166	1.472207	3.124803
H	3.513873	0.558993	3.151880
H	2.616812	1.622601	2.073106
C	3.745129	2.658609	3.603913
H	4.026926	2.504138	4.655583
H	3.129989	3.570889	3.590643
C	5.011903	2.898789	2.772356
H	5.633812	1.992075	2.791885
H	4.729755	3.050455	1.721440
C	5.841646	4.092346	3.256944
H	6.164417	3.954501	4.295389
H	6.739599	4.235437	2.646061
H	5.260063	5.020632	3.215189

^{1}**TS12**-*R*

B3LYP-D3BJ/BSI SCF energy in solution:

-2922.522219a.u.

M06/BSII SCF energy in solution: -2920.80232a.u.

M06/BSII free energy in solution: -2919.830986a.u.

B3LYP-D3BJ/BSI SCF energy in solution:

-2922.522219a.u.

B3LYP-D3BJ /BSII SCF energy in solution:

-2923.177336 a.u.

B3LYP-D3BJ /BSII free energy in solution: -2922.206002 a.u.

C	1.114141	-2.210893	-0.465925
N	1.291527	-1.244677	0.354471
O	2.163024	-2.558768	-1.240405
C	3.333759	-1.852349	-0.688175
C	2.622363	-0.680721	0.101703
H	3.126985	-0.509980	1.048990
C	2.533194	0.631267	-0.649744
C	3.646572	1.480814	-0.648198
C	1.408680	0.984304	-1.404125
C	3.652268	2.645501	-1.413664
H	4.520169	1.214199	-0.061447
C	1.410992	2.154540	-2.165909
H	0.528619	0.351295	-1.398882
C	2.534545	2.980804	-2.181851
H	4.527345	3.288866	-1.410761
H	0.529366	2.418652	-2.739944
H	2.532441	3.889494	-2.775284
C	-1.332696	-2.075483	-0.459818
N	-1.437604	-1.137734	0.411794
O	-2.381520	-2.248339	-1.292101
C	-3.189550	-1.022742	-1.175678
C	-2.781588	-0.547770	0.269104
H	-2.706707	0.534062	0.293026
C	-3.712146	-0.994699	1.374587
C	-4.786219	-0.167539	1.725505
C	-3.562586	-2.229843	2.013112
C	-5.706359	-0.578140	2.689616
H	-4.880459	0.806028	1.255277
C	-4.479472	-2.637008	2.984203
H	-2.726630	-2.873177	1.761747
C	-5.556583	-1.815636	3.320848
H	-6.536642	0.070948	2.952609
H	-4.351361	-3.597138	3.475815
H	-6.271168	-2.134535	4.074039
Ni	-0.064860	-0.726880	1.807744
C	-0.156774	-3.018886	-0.627176
C	-0.196364	-4.080368	0.505807
H	0.677444	-4.733124	0.428155
H	-0.188185	-3.594071	1.484653
H	-1.099394	-4.689442	0.409175
C	-0.195278	-3.704951	-2.003309
H	-1.121525	-4.272260	-2.106550
H	-0.142683	-2.976243	-2.815423
H	0.647435	-4.391276	-2.097379
C	-2.682646	-0.075730	-2.265295
C	-2.240489	-0.622096	-3.478962
C	-2.676877	1.314760	-2.106084
C	-1.778891	0.202025	-4.503491
H	-2.253402	-1.697099	-3.615335
C	-2.222636	2.139305	-3.139152
H	-3.030521	1.775622	-1.189857
C	-1.766566	1.588654	-4.336231
H	-1.431324	-0.239613	-5.432853
H	-2.213125	3.214095	-2.993782
H	-1.405127	2.233407	-5.131837
C	-4.650266	-1.384302	-1.334473
C	-5.582027	-0.396260	-1.671396

C	-5.099003	-2.681138	-1.060607
C	-6.942777	-0.698346	-1.723800
H	-5.248014	0.611973	-1.890242
C	-6.459172	-2.983202	-1.121351
H	-4.383977	-3.451416	-0.798555
C	-7.386649	-1.992797	-1.449159
H	-7.654350	0.080077	-1.982649
H	-6.792887	-3.994647	-0.908656
H	-8.445902	-2.228391	-1.491866
C	4.198171	-1.403651	-1.850176
C	5.576002	-1.224065	-1.692634
C	3.603952	-1.067733	-3.073679
C	6.344906	-0.701687	-2.734207
H	6.053918	-1.485626	-0.755462
C	4.373037	-0.551210	-4.114169
H	2.537643	-1.202649	-3.206169
C	5.746723	-0.361140	-3.947269
H	7.413136	-0.564955	-2.594218
H	3.896406	-0.293273	-5.055265
H	6.344846	0.045727	-4.757199
C	4.035833	-2.824849	0.256203
C	3.971675	-4.201437	0.003886
C	4.786637	-2.372950	1.348227
C	4.625872	-5.107172	0.838247
H	3.404528	-4.562135	-0.846226
C	5.447074	-3.279922	2.179615
H	4.869817	-1.312271	1.559456
C	5.364842	-4.649917	1.931082
H	4.559242	-6.171269	0.631432
H	6.021494	-2.910793	3.023903
H	5.872785	-5.355180	2.581985
C	-1.833173	2.203070	2.576841
C	-2.444781	2.812466	1.398454
C	-0.506901	2.854818	2.218484
H	-0.142875	3.620724	2.913761
H	0.298223	2.172495	1.938168
N	-1.226833	3.418829	1.050400
O	-3.543959	2.841278	0.842701
C	-0.831622	4.345280	0.104414
C	0.502844	4.784800	0.081299
C	-1.756278	4.858634	-0.826257
C	0.904277	5.725675	-0.863194
H	1.216697	4.377565	0.789242
C	-1.332888	5.793821	-1.766367
H	-2.784615	4.520721	-0.790149
C	-0.005134	6.233746	-1.794110
H	1.938480	6.056235	-0.874638
H	-2.050013	6.186699	-2.481599
H	0.314610	6.964881	-2.529955
C	-2.352082	1.533296	3.782707
H	-3.400410	1.256712	3.652087
H	-1.779263	0.629282	4.014681
H	-2.274363	2.194502	4.659560
C	0.707688	-0.577876	3.636511
H	1.446955	-1.396855	3.721948
H	-0.082367	-0.851452	4.360333
C	1.378679	0.716197	4.132478
H	0.636966	1.525725	4.185873
H	1.761370	0.614288	5.164767
C	2.541734	1.184775	3.252723
H	3.313399	0.399920	3.233537
H	2.188349	1.281598	2.218674
C	3.174857	2.507430	3.693349
H	3.544270	2.416658	4.725115

H 2.399617 3.287382 3.717432
C 4.317733 2.971127 2.785035
H 5.098934 2.198066 2.764727
H 3.946789 3.055821 1.755424
C 4.929995 4.304455 3.218440
H 5.337755 4.242722 4.234555
H 5.743760 4.611661 2.551996
H 4.178137 5.102758 3.213927

[1]**TS12**-*R*

*w*B97XD/BSI SCF energy in gas:

-2921.298623a.u.

*w*B97XD/BSII SCF energy in solution:

-2921.949118a.u.

*w*B97XD/BSII free energy in solution:

-2920.959517a.u.

C -0.474631 -2.441595 -0.462882
N -0.920453 -1.319784 -0.885656
O -1.261201 -3.144859 0.343021
C -2.548904 -2.464374 0.427086
C -2.239649 -1.084836 -0.290242
H -2.947350 -0.944599 -1.107217
C -2.274237 0.145998 0.584549
C -3.502425 0.767329 0.810309
C -1.129314 0.660672 1.188821
C -3.589768 1.890448 1.625517
H -4.399164 0.379810 0.332389
C -1.216082 1.789485 1.992936
H -0.160535 0.201726 1.020477
C -2.440801 2.409333 2.214237
H -4.550435 2.371203 1.781233
H -0.314222 2.208794 2.420191
H -2.487265 3.312569 2.814230
C 1.866612 -1.902948 -0.479774
N 1.752328 -0.706303 -0.904878
O 2.914082 -2.181256 0.305151
C 3.406081 -0.876142 0.748260
C 2.934397 0.028706 -0.435199
H 2.625387 1.017016 -0.090638
C 3.947073 0.220321 -1.542121
C 4.645035 1.427826 -1.598124
C 4.213087 -0.766636 -2.491249
C 5.607906 1.637432 -2.580358
H 4.396532 2.209837 -0.886532
C 5.173052 -0.556097 -3.475731
H 3.665457 -1.704596 -2.469360
C 5.875355 0.645546 -3.519820
H 6.140431 2.582472 -2.618799
H 5.371878 -1.329537 -4.211309
H 6.623097 0.810487 -4.289358
Ni 0.041678 -0.073524 -1.942969
C 0.887089 -3.020673 -0.770723
C 0.950162 -3.383754 -2.271384
H 0.198282 -4.143996 -2.499041
H 0.764707 -2.503294 -2.891085
H 1.937702 -3.789858 -2.506812
C 1.171083 -4.252281 0.095546
H 2.179654 -4.617431 -0.105920
H 1.090650 -4.024200 1.159749
H 0.456273 -5.042342 -0.142183
C 2.663120 -0.566812 2.051302
C 2.325566 -1.623738 2.902721
C 2.346464 0.739044 2.432198

C	1.667669	-1.384317	4.104299
H	2.583373	-2.639024	2.621107
C	1.709760	0.974767	3.649277
H	2.552009	1.583660	1.780043
C	1.358610	-0.080137	4.483264
H	1.404744	-2.218058	4.748599
H	1.476479	1.996318	3.933285
H	0.851033	0.111342	5.423470
C	4.905525	-0.947530	0.938011
C	5.563096	0.093761	1.594586
C	5.651579	-1.997968	0.406943
C	6.947782	0.085343	1.713318
H	4.992700	0.915819	2.014932
C	7.037208	-2.009644	0.535923
H	5.146671	-2.805567	-0.109871
C	7.689894	-0.968136	1.186450
H	7.446594	0.904563	2.221294
H	7.606489	-2.835572	0.121142
H	8.770867	-0.976690	1.284142
C	-2.882031	-2.347190	1.906431
C	-4.186939	-2.471279	2.377080
C	-1.859452	-2.044296	2.811039
C	-4.468666	-2.284950	3.728556
H	-4.991409	-2.717962	1.692995
C	-2.141595	-1.858350	4.156604
H	-0.835652	-1.936928	2.467849
C	-3.449534	-1.975961	4.621305
H	-5.490356	-2.385913	4.080738
H	-1.334691	-1.608864	4.838210
H	-3.670391	-1.826209	5.673334
C	-3.549145	-3.298886	-0.363266
C	-3.347491	-4.671024	-0.520019
C	-4.699056	-2.720747	-0.906060
C	-4.264511	-5.443978	-1.224653
H	-2.465677	-5.132819	-0.090845
C	-5.621143	-3.495495	-1.602924
H	-4.890132	-1.658936	-0.782187
C	-5.403259	-4.859212	-1.770502
H	-4.087889	-6.507912	-1.346712
H	-6.507276	-3.028330	-2.020188
H	-6.116226	-5.462466	-2.323082
C	0.397573	2.124256	-1.631951
C	1.014090	2.891895	-0.583540
C	-0.776735	3.108085	-1.582971
H	-0.922670	3.746150	-2.467247
H	-1.742182	2.712883	-1.249493
N	-0.070796	3.791470	-0.498765
O	2.067488	2.911211	0.070930
C	-0.572622	4.600521	0.499258
C	-1.857690	5.147498	0.370126
C	0.184390	4.891408	1.646598
C	-2.383066	5.944335	1.379660
H	-2.443325	4.932130	-0.518678
C	-0.361442	5.681812	2.649340
H	1.183437	4.477928	1.726656
C	-1.646612	6.212932	2.531042
H	-3.381207	6.357398	1.263826
H	0.229296	5.892298	3.536804
H	-2.059800	6.835888	3.317696
C	1.111728	1.668808	-2.873441
H	1.051775	0.567474	-3.204384
H	0.756772	2.179976	-3.776442
H	2.192810	1.797677	-2.776639
C	-1.226417	-0.309600	-3.406711

H -1.859190 -1.180306 -3.188029

H -0.592481 -0.596337 -4.258388

C -2.100735 0.880713 -3.795219

H -1.489147 1.788563 -3.871759

H -2.521473 0.723770 -4.800647

C -3.247100 1.144594 -2.820972

H -3.958449 0.304627 -2.853028

H -2.840502 1.169231 -1.805710

C -3.990143 2.454091 -3.077718

H -4.501143 2.412980 -4.049549

H -3.255924 3.267687 -3.153827

C -4.992277 2.801260 -1.976863

H -5.738927 1.999224 -1.895558

H -4.464200 2.828092 -1.015166

C -5.693964 4.137462 -2.209201

H -6.238177 4.138635 -3.159928

H -6.409823 4.361275 -1.412558

H -4.966597 4.955894 -2.244652

^{3}**TS12**-*R*

B3LYP/BSI SCF energy: -2922.107717a.u.

M06/BSII SCF energy in solution: -2920.788647a.u.

M06/BSII free energy in solution: -2919.829516a.u.

C -0.535578 -1.892881 1.142616

N -0.922522 -1.133712 0.184821

O -1.482249 -2.621466 1.779104

C -2.693692 -2.550121 0.923669

C -2.399707 -1.211672 0.136869

H -2.711491 -1.312657 -0.902425

C -3.047122 0.049653 0.688777

C -4.297895 0.441165 0.193244

C -2.442680 0.831332 1.679511

C -4.937284 1.577527 0.686949

H -4.771253 -0.142836 -0.590868

C -3.075957 1.974950 2.168516

H -1.460889 0.561945 2.054394

C -4.326461 2.349459 1.676422

H -5.904704 1.866720 0.286654

H -2.583318 2.586319 2.918268

H -4.809566 3.247621 2.048289

C 1.894191 -1.236527 0.997022

N 1.808086 -0.591705 -0.107755

O 3.084218 -1.196676 1.644080

C 3.915120 -0.183543 0.986005

C 3.130610 0.006559 -0.390134

H 2.980492 1.071984 -0.565068

C 3.776354 -0.577942 -1.635096

C 4.621671 0.231043 -2.404794

C 3.563720 -1.901922 -2.036016

C 5.250922 -0.271424 -3.543739

H 4.788865 1.265648 -2.114375

C 4.187509 -2.405806 -3.177823

H 2.887764 -2.533942 -1.470437

C 5.034773 -1.593865 -3.933467

H 5.901203 0.371882 -4.129400

H 4.003696 -3.432421 -3.481109

H 5.516334 -1.986173 -4.824374

Ni 0.235526 -0.545113 -1.433392

C 0.869023 -2.140806 1.652818

C 1.251886 -3.613997 1.321113

H 0.533710 -4.291898 1.786587

H 1.239526 -3.785352 0.241490

H 2.250073 -3.834330 1.707889

C	0.886268	-1.937758	3.190045
H	1.870983	-2.189282	3.586998
H	0.659786	-0.901168	3.456136
H	0.141734	-2.587456	3.652697
C	3.865494	1.078550	1.859977
C	3.692717	0.956863	3.246202
C	4.048888	2.360054	1.322864
C	3.679288	2.084553	4.066343
H	3.564930	-0.027570	3.681056
C	4.045348	3.490006	2.144900
H	4.205184	2.494291	0.256837
C	3.853426	3.357269	3.519728
H	3.536481	1.966477	5.136667
H	4.191595	4.472449	1.705477
H	3.844684	4.235199	4.158723
C	5.332301	-0.748012	0.876884
C	6.441559	0.100920	0.769871
C	5.544963	-2.132885	0.855706
C	7.728293	-0.421303	0.635151
H	6.309326	1.176506	0.803709
C	6.832397	-2.654290	0.730298
H	4.701274	-2.805913	0.948378
C	7.930315	-1.801155	0.616600
H	8.573815	0.255665	0.553938
H	6.974816	-3.731138	0.722263
H	8.932757	-2.207389	0.518820
C	-3.922260	-2.513742	1.815414
C	-5.183291	-2.772311	1.259012
C	-3.837134	-2.186993	3.172920
C	-6.333810	-2.697881	2.042424
H	-5.263826	-3.041991	0.210307
C	-4.989284	-2.122291	3.958845

H	-2.868175	-1.986853	3.614288
C	-6.240782	-2.374511	3.397541
H	-7.302399	-2.898825	1.593854
H	-4.905010	-1.872016	5.012567
H	-7.136282	-2.322150	4.009925
C	-2.634988	-3.809662	0.045995
C	-2.965001	-5.045572	0.628166
C	-2.172312	-3.797914	-1.277546
C	-2.853947	-6.231212	-0.094182
H	-3.315429	-5.074341	1.654864
C	-2.064874	-4.988931	-2.002559
H	-1.873697	-2.875419	-1.763262
C	-2.406686	-6.206562	-1.417621
H	-3.120462	-7.173881	0.375441
H	-1.710561	-4.953549	-3.028620
H	-2.325818	-7.129168	-1.985367
C	0.721498	3.196023	-2.043205
C	-0.593903	3.829621	-1.944086
N	-0.379619	4.084840	-0.562157
C	-1.072335	4.766108	0.421901
C	-2.328564	5.340384	0.137696
C	-0.525022	4.896879	1.712024
C	-3.010154	6.026978	1.137045
H	-2.738251	5.234127	-0.859785
C	-1.226731	5.587351	2.698951
H	0.443481	4.458845	1.934894
C	-2.471690	6.156424	2.422256
H	-3.975754	6.469787	0.907673
H	-0.792513	5.683602	3.690712
H	-3.012163	6.697209	3.193274
C	1.593888	2.825525	-3.178663
H	1.992965	1.808568	-3.082799

H 1.030340 2.874120 -4.113699

H 2.453812 3.509239 -3.264123

C -0.280337 -0.648320 -3.349450

H -0.662300 -1.668521 -3.540514

H 0.691976 -0.628400 -3.873623

C -1.215916 0.358944 -4.043883

H -0.808405 1.374223 -3.962247

H -1.279384 0.151063 -5.127659

C -2.648403 0.391526 -3.497939

H -3.083843 -0.619871 -3.559936

H -2.615680 0.654362 -2.431810

C -3.559809 1.384004 -4.232518

H -3.568403 1.143810 -5.306422

H -3.127831 2.389248 -4.140855

C -5.003176 1.406938 -3.713964

H -5.433412 0.396877 -3.784700

H -4.995903 1.662851 -2.645752

C -5.901537 2.395049 -4.465836

H -5.954870 2.147721 -5.532731

H -6.924899 2.393884 -4.073458

H -5.513928 3.416891 -4.385091

C 0.970195 3.487294 -0.570609

H 1.075520 2.621759 0.094541

H 1.769573 4.211998 -0.351875

O -1.531768 4.103892 -2.673876

^{2}IM7

B3LYP/BSI SCF energy: -2405.084799a.u.

M06/BSII SCF energy in solution: -2403.993115a.u.

M06/BSII free energy in solution: -2403.198293a.u.

C 1.003420 -0.773180 0.966589

N 1.192866 -0.327223 -0.215692

O 2.106152 -1.125735 1.678604

C 3.270686 -0.622056 0.934950

C 2.638817 -0.401291 -0.503643

H 2.941315 0.564244 -0.904515

C 2.939380 -1.452957 -1.558283

C 3.844830 -1.156824 -2.583203

C 2.335686 -2.716395 -1.538041

C 4.151365 -2.102997 -3.562105

H 4.303738 -0.172367 -2.624883

C 2.639036 -3.664013 -2.514760

H 1.614488 -2.958811 -0.763864

C 3.549801 -3.361206 -3.529208

H 4.851434 -1.852250 -4.353819

H 2.160990 -4.639239 -2.485736

H 3.782522 -4.098506 -4.292209

C -1.511259 -0.695713 0.833106

N -1.572172 -0.065254 -0.283632

O -2.681048 -1.148143 1.350521

C -3.691583 -1.024102 0.280311

C -3.001792 0.043922 -0.660862

H -3.111691 -0.266844 -1.700441

C -3.483601 1.481426 -0.565761

C -4.165348 2.046901 -1.649002

C -3.257210 2.266859 0.571956

C -4.620968 3.365345 -1.598304

H -4.331086 1.454117 -2.545195

C -3.710933 3.583365 0.625989

H -2.718601 1.852621 1.418311

C -4.395465 4.136913 -0.458742

H -5.141906 3.789311 -2.451881

H -3.525427 4.180711 1.514119

H	-4.742954	5.165141	-0.417182
Ni	-0.117435	0.678570	-1.459052
C	-0.302757	-0.961148	1.710920
C	-0.344445	0.044762	2.898057
H	0.504037	-0.129830	3.562846
H	-0.296414	1.074889	2.534975
H	-1.269641	-0.090525	3.463955
C	-0.363593	-2.412672	2.254870
H	-1.283304	-2.557962	2.822913
H	-0.349829	-3.140613	1.438315
H	0.492695	-2.599062	2.905323
C	-3.782865	-2.420354	-0.358856
C	-4.623102	-3.386708	0.216115
C	-2.975981	-2.800880	-1.442842
C	-4.671469	-4.686248	-0.285603
H	-5.247835	-3.117188	1.060845
C	-3.026148	-4.103096	-1.945954
H	-2.291288	-2.097054	-1.904328
C	-3.875200	-5.049187	-1.373323
H	-5.334778	-5.414040	0.173096
H	-2.397682	-4.371525	-2.790218
H	-3.916538	-6.059592	-1.769915
C	-5.009297	-0.579212	0.891856
C	-6.135348	-0.458149	0.064096
C	-5.135679	-0.282032	2.251987
C	-7.356712	-0.037130	0.584672
H	-6.056948	-0.700342	-0.991804
C	-6.363183	0.134389	2.774529
H	-4.274265	-0.379847	2.901844
C	-7.475738	0.261000	1.944566
H	-8.216956	0.055830	-0.071850
H	-6.445008	0.359128	3.834231
H	-8.428748	0.586477	2.351272
C	4.383014	-1.663051	1.021630
C	5.678861	-1.329287	0.604261
C	4.141766	-2.955663	1.500093
C	6.705926	-2.269908	0.653676
H	5.891339	-0.325768	0.249484
C	5.173928	-3.894434	1.558106
H	3.147030	-3.225296	1.833569
C	6.458281	-3.557647	1.133196
H	7.702765	-1.992746	0.323044
H	4.968960	-4.891563	1.937652
H	7.260198	-4.288791	1.177982
C	3.697088	0.686120	1.622372
C	3.656270	0.746657	3.024538
C	4.184951	1.795582	0.922473
C	4.069451	1.890451	3.703535
H	3.297459	-0.112119	3.581758
C	4.605420	2.941857	1.603840
H	4.243454	1.790689	-0.160473
C	4.545190	2.996238	2.994483
H	4.023979	1.916775	4.788666
H	4.972678	3.792754	1.037744
H	4.866739	3.889265	3.522280
C	0.626109	1.695632	-2.989484
H	-0.217849	2.118208	-3.564196
H	1.101225	0.981764	-3.682878
C	1.617986	2.842399	-2.711295
H	2.530029	2.447059	-2.231153
H	1.971422	3.311384	-3.648573
C	1.053346	3.954523	-1.818271
H	0.143667	4.357764	-2.287813
H	0.726591	3.510194	-0.867189

C 2.029996 5.105160 -1.543081

H 2.361061 5.542790 -2.496654

H 2.937501 4.704982 -1.064880

C 1.448103 6.213910 -0.657122

H 0.544684 6.618847 -1.133620

H 1.116427 5.777273 0.295139

C 2.432315 7.355210 -0.379301

H 2.756618 7.834084 -1.310697

H 1.986625 8.130014 0.254365

H 3.331489 6.987472 0.129602

^{2}IM7

B3LYP-D3BJ/BSI SCF energy in solution:

-2405.427104a.u.

M06/BSII SCF energy in solution: -2404.003632a.u.

M06/BSII free energy in solution: -2403.199512a.u.

C -1.004416 -0.483409 -0.921253

N -1.168771 -0.208897 0.317725

O -2.119748 -0.752336 -1.637818

C -3.265855 -0.381811 -0.796109

C -2.592782 -0.380827 0.636939

H -2.932221 0.482703 1.204934

C -2.822504 -1.629955 1.460216

C -3.995487 -1.735988 2.216274

C -1.928040 -2.705130 1.437192

C -4.281723 -2.904083 2.920950

H -4.692893 -0.903487 2.242547

C -2.209376 -3.872602 2.149209

H -1.008059 -2.629094 0.868436

C -3.389400 -3.978644 2.886411

H -5.199040 -2.975066 3.497999

H -1.505142 -4.699351 2.124495

H -3.610103 -4.888468 3.436864

C 1.497934 -0.486149 -0.827442

N 1.576502 -0.202227 0.423330

O 2.660393 -0.751585 -1.462945

C 3.672639 -0.952326 -0.400112

C 3.008492 -0.157858 0.781108

H 3.173506 -0.685831 1.719920

C 3.472328 1.274651 0.948903

C 4.484109 1.563161 1.870418

C 2.935364 2.314024 0.180144

C 4.962186 2.865825 2.015291

H 4.900743 0.761981 2.474177

C 3.408228 3.618033 0.327401

H 2.137875 2.111377 -0.525710

C 4.425111 3.897391 1.242729

H 5.749129 3.074725 2.733976

H 2.979126 4.415788 -0.271849

H 4.792274 4.913005 1.357280

Ni 0.187678 0.470671 1.663672

C 0.277086 -0.533841 -1.715011

C 0.316067 0.699036 -2.659827

H -0.516236 0.651172 -3.364957

H 0.238823 1.628179 -2.089049

H 1.251636 0.702857 -3.223527

C 0.306332 -1.842061 -2.542396

H 1.209357 -1.871236 -3.153152

H 0.302598 -2.718432 -1.887634

H -0.565935 -1.887148 -3.195884

C 3.701606 -2.462455 -0.174826

C 4.443504 -3.263677 -1.055100

C 2.913579 -3.087114 0.801664

C	4.412503	-4.652958	-0.953287
H	5.048710	-2.795146	-1.823733
C	2.881908	-4.479810	0.902468
H	2.310935	-2.504712	1.488866
C	3.631610	-5.266844	0.028838
H	4.998109	-5.255598	-1.641217
H	2.268634	-4.945114	1.668502
H	3.608546	-6.349422	0.111382
C	4.992884	-0.373082	-0.847176
C	6.150324	-0.688368	-0.124090
C	5.071531	0.540096	-1.901470
C	7.367925	-0.093117	-0.448537
H	6.096469	-1.398504	0.695010
C	6.293382	1.130052	-2.229986
H	4.178160	0.790465	-2.460483
C	7.443323	0.819031	-1.503754
H	8.257325	-0.342529	0.122440
H	6.342862	1.836739	-3.053167
H	8.392039	1.282176	-1.757997
C	-4.343696	-1.433901	-0.988220
C	-5.693836	-1.127561	-0.789780
C	-3.982942	-2.756701	-1.276747
C	-6.664879	-2.127658	-0.863430
H	-5.994375	-0.108797	-0.574834
C	-4.953858	-3.753127	-1.355642
H	-2.940832	-3.005900	-1.435017
C	-6.299094	-3.443946	-1.143884
H	-7.708352	-1.872664	-0.703451
H	-4.657271	-4.773542	-1.579935
H	-7.054772	-4.221807	-1.200828
C	-3.701369	1.019074	-1.217410
C	-3.466001	1.457438	-2.526515

C	-4.383705	1.865143	-0.334712
C	-3.883910	2.723148	-2.937174
H	-2.953601	0.805596	-3.223767
C	-4.815796	3.125904	-0.750709
H	-4.591019	1.548207	0.681985
C	-4.561104	3.562899	-2.050858
H	-3.683340	3.050000	-3.953253
H	-5.345098	3.766569	-0.052535
H	-4.887433	4.547739	-2.370241
C	-0.467697	1.553909	3.191046
H	0.409132	2.070338	3.625720
H	-0.878244	0.960002	4.026098
C	-1.499735	2.630574	2.797145
H	-2.475256	2.156178	2.608387
H	-1.683582	3.351643	3.615476
C	-1.115757	3.422901	1.541159
H	-0.088033	3.799656	1.650922
H	-1.091018	2.730569	0.688524
C	-2.051227	4.589518	1.213580
H	-1.974004	5.358794	1.995572
H	-3.091480	4.236892	1.232560
C	-1.770745	5.218312	-0.154323
H	-0.721895	5.544022	-0.196837
H	-1.885333	4.447027	-0.927554
C	-2.686058	6.400547	-0.475795
H	-2.554028	7.213141	0.248648
H	-2.485128	6.808399	-1.472931
H	-3.740321	6.102393	-0.446309

^{1}**TS12**-*S*

B3LYP/BSI SCF energy: -2922.094041a.u.

M06/BSII SCF energy in solution: -2920.78895a.u.

M06/BSII free energy in solution: -2919.829821a.u.

C	-0.517074	-1.866917	1.128830
N	-0.911309	-1.084793	0.193032
O	-1.464617	-2.574598	1.788265
C	-2.699806	-2.455324	0.975410
C	-2.391252	-1.115987	0.196879
H	-2.743696	-1.187473	-0.831201
C	-2.977703	0.154528	0.794301
C	-4.227121	0.600857	0.343686
C	-2.319875	0.889997	1.786472
C	-4.812083	1.746557	0.881408
H	-4.742668	0.052694	-0.439618
C	-2.899936	2.041438	2.320873
H	-1.338319	0.578567	2.128133
C	-4.148866	2.471671	1.872416
H	-5.778890	2.079288	0.514767
H	-2.366820	2.615212	3.072650
H	-4.589719	3.376941	2.277927
C	1.919210	-1.244935	0.954380
N	1.826258	-0.550122	-0.120511
O	3.119203	-1.249601	1.584456
C	3.945500	-0.206201	0.968706
C	3.159464	0.028890	-0.398023
H	3.031279	1.100474	-0.548786
C	3.793127	-0.540431	-1.657592
C	4.739850	0.230631	-2.345524
C	3.472456	-1.809576	-2.152278
C	5.361091	-0.256912	-3.494852
H	4.996705	1.221929	-1.979522
C	4.088645	-2.297394	-3.305978
H	2.717966	-2.407524	-1.653323
C	5.035933	-1.525193	-3.979032
H	6.091665	0.356144	-4.014568
H	3.819590	-3.279962	-3.682672
H	5.511704	-1.904746	-4.878537
Ni	0.234162	-0.447776	-1.416335
C	0.894772	-2.167447	1.585475
C	1.238790	-3.625425	1.155950
H	0.521502	-4.317012	1.602751
H	1.190117	-3.731350	0.069111
H	2.243675	-3.886309	1.497686
C	0.958605	-2.057054	3.130594
H	1.948904	-2.348326	3.483867
H	0.759544	-1.034325	3.464118
H	0.214454	-2.719375	3.575149
C	3.879490	1.024973	1.884859
C	3.684805	0.858193	3.263214
C	4.067194	2.323738	1.392042
C	3.653441	1.959239	4.118720
H	3.554108	-0.140204	3.663907
C	4.044891	3.426811	2.249261
H	4.242850	2.491986	0.333880
C	3.830725	3.249298	3.615809
H	3.493697	1.806544	5.182249
H	4.193906	4.423320	1.843784
H	3.807091	4.106322	4.282230
C	5.364655	-0.761275	0.845059
C	6.483033	0.080841	0.886171
C	5.568716	-2.136602	0.663401
C	7.771011	-0.436495	0.737548
H	6.357095	1.145340	1.048015
C	6.855617	-2.653588	0.523417
H	4.717762	-2.806525	0.639607

C	7.963440	-1.805384	0.556567
H	8.623844	0.235211	0.773540
H	6.991007	-3.722867	0.388541
H	8.965912	-2.208711	0.447357
C	-3.896960	-2.396090	1.908329
C	-5.182615	-2.605434	1.389036
C	-3.757735	-2.099867	3.268336
C	-6.304401	-2.511275	2.210958
H	-5.305426	-2.851936	0.338655
C	-4.881441	-2.016129	4.092815
H	-2.769279	-1.938642	3.681594
C	-6.157700	-2.218100	3.568249
H	-7.292722	-2.673587	1.790659
H	-4.755306	-1.790456	5.147934
H	-7.030875	-2.150647	4.210660
C	-2.712510	-3.707073	0.084877
C	-3.016777	-4.942114	0.683243
C	-2.349957	-3.692659	-1.268606
C	-2.974871	-6.124406	-0.051133
H	-3.292911	-4.971365	1.732456
C	-2.313233	-4.880416	-2.006448
H	-2.077817	-2.770852	-1.769790
C	-2.626531	-6.097040	-1.404254
H	-3.218916	-7.066547	0.431590
H	-2.035784	-4.843246	-3.055840
H	-2.600189	-7.017020	-1.981322
C	0.742053	2.883129	-2.084802
C	-0.582704	3.509409	-2.017729
N	-0.359444	3.873783	-0.663471
C	-1.028013	4.671659	0.248665
C	-2.280619	5.229482	-0.079408
C	-0.457970	4.937781	1.507591
C	-2.935435	6.035868	0.845730
H	-2.708113	5.017466	-1.052115
C	-1.132834	5.746764	2.420432
H	0.506319	4.509400	1.764831
C	-2.373423	6.301651	2.099443
H	-3.898511	6.465129	0.582423
H	-0.681337	5.946552	3.388776
H	-2.893047	6.934943	2.812225
C	1.641574	2.583599	-3.222823
H	2.366563	1.798861	-2.986481
H	1.060954	2.254569	-4.087975
H	2.212530	3.477807	-3.526747
C	-0.374285	-0.615119	-3.300367
H	-0.710808	-1.662156	-3.419436
H	0.580638	-0.574313	-3.855066
C	-1.379496	0.304727	-4.015145
H	-1.010481	1.337018	-4.031289
H	-1.486997	0.010191	-5.075247
C	-2.784473	0.334753	-3.401156
H	-3.178714	-0.693772	-3.338685
H	-2.713563	0.710580	-2.371843
C	-3.772936	1.208771	-4.184572
H	-3.831468	0.852001	-5.223886
H	-3.374304	2.230605	-4.226633
C	-5.185102	1.242953	-3.586797
H	-5.579514	0.218071	-3.520435
H	-5.128318	1.617104	-2.555529
C	-6.162223	2.110546	-4.387667
H	-6.265304	1.743483	-5.415679
H	-7.161096	2.120075	-3.936833
H	-5.811471	3.147335	-4.443512
C	1.004612	3.311204	-0.645837

H 1.142971 2.512015 0.091639

H 1.786765 4.074229 -0.508753

O -1.528619 3.724816 -2.756737

[1]**TS12**-*S*

B3LYP-D3BJ/BSI SCF energy in solution:

-2922.517859a.u.

M06/BSII SCF energy in solution: -2920.802255a.u.

M06/BSII free energy in solution: -2919.831643a.u.

B3LYP-D3BJ/BSI SCF energy in solution:

-2922.517859a.u.

B3LYP-D3BJ /BSII SCF energy in solution:

-2923.174778 a.u.

B3LYP-D3BJ /BSII free energy in solution:

-2922.204166 a.u.

C -0.399232 -2.358419 0.418862

N -0.771116 -1.355129 -0.288746

O -1.361962 -3.074410 1.032053

C -2.644647 -2.617159 0.432303

C -2.219982 -1.194367 -0.092338

H -2.707615 -0.986731 -1.041787

C -2.522154 -0.061129 0.864570

C -3.758496 0.586867 0.771671

C -1.642877 0.296426 1.892427

C -4.126999 1.551805 1.707602

H -4.438971 0.322971 -0.031123

C -2.011216 1.260057 2.831321

H -0.669350 -0.175464 1.964238

C -3.257647 1.881834 2.748298

H -5.089542 2.047029 1.621794

H -1.317535 1.528823 3.621632

H -3.542113 2.635321 3.475419

C 1.963058 -1.686087 0.466797

N 1.918696 -0.751521 -0.413136

O 2.972633 -1.635424 1.360282

C 3.570537 -0.300203 1.235694

C 3.118992 0.085822 -0.234189

H 2.833346 1.135470 -0.252792

C 4.145916 -0.152163 -1.318319

C 5.140598 0.812410 -1.522413

C 4.159386 -1.319344 -2.086967

C 6.142499 0.607646 -2.467985

H 5.135327 1.720537 -0.926105

C 5.158665 -1.521966 -3.042006

H 3.387939 -2.067450 -1.943979

C 6.154020 -0.563033 -3.231638

H 6.911594 1.360677 -2.611929

H 5.157991 -2.431918 -3.634912

H 6.932452 -0.722752 -3.971745

Ni 0.415881 -0.529846 -1.752363

C 1.018236 -2.866210 0.569174

C 1.299782 -3.833582 -0.614357

H 0.595082 -4.668398 -0.579349

H 1.186592 -3.313100 -1.568654

H 2.315982 -4.229645 -0.533987

C 1.190161 -3.605165 1.907154

H 2.215536 -3.965850 2.002659

H 0.970330 -2.955783 2.757952

H 0.514128 -4.460374 1.942467

C 2.874606 0.609913 2.246447

C 2.148961 0.073062 3.315975

C 2.948981 2.003113 2.114170

C	1.491853	0.910751	4.219266
H	2.094953	-1.001180	3.441486
C	2.299894	2.841039	3.021774
H	3.514115	2.445649	1.300375
C	1.560681	2.296999	4.073855
H	0.926222	0.475071	5.037365
H	2.367310	3.917735	2.900221
H	1.047527	2.947900	4.775152
C	5.066818	-0.448292	1.441553
C	5.831804	0.556946	2.040273
C	5.714659	-1.593114	0.955115
C	7.219931	0.431097	2.132073
H	5.354059	1.442741	2.440055
C	7.097870	-1.719864	1.051072
H	5.133835	-2.383365	0.496198
C	7.858432	-0.704222	1.635577
H	7.797972	1.223264	2.598579
H	7.581967	-2.612323	0.665903
H	8.937696	-0.800987	1.706645
C	-3.705990	-2.577623	1.504444
C	-5.053019	-2.508235	1.128067
C	-3.369852	-2.496112	2.858860
C	-6.047929	-2.351868	2.091893
H	-5.322370	-2.569608	0.078541
C	-4.367081	-2.344495	3.822953
H	-2.329263	-2.543965	3.156377
C	-5.707824	-2.268243	3.443635
H	-7.088281	-2.295230	1.785730
H	-4.093053	-2.283248	4.872116
H	-6.482325	-2.147316	4.195179
C	-2.927040	-3.616858	-0.683763
C	-3.445752	-4.875641	-0.345300

C	-2.575854	-3.368083	-2.016633
C	-3.624701	-5.857114	-1.317618
H	-3.710661	-5.084356	0.685915
C	-2.756744	-4.352314	-2.991254
H	-2.148179	-2.418995	-2.314662
C	-3.282349	-5.597129	-2.647279
H	-4.031138	-6.824373	-1.037141
H	-2.482334	-4.138585	-4.020071
H	-3.423521	-6.360111	-3.407146
C	0.976716	2.765970	-2.017165
C	-0.156660	3.673223	-2.189921
N	-0.461120	3.585115	-0.814647
C	-1.302839	4.281993	0.032741
C	-2.377119	5.028339	-0.490413
C	-1.082669	4.252021	1.419710
C	-3.214337	5.724373	0.376106
H	-2.531837	5.052158	-1.561742
C	-1.927638	4.962186	2.269116
H	-0.263814	3.666551	1.823211
C	-2.997977	5.699398	1.758104
H	-4.044703	6.292339	-0.033830
H	-1.748813	4.932062	3.340174
H	-3.656306	6.246275	2.425997
C	2.101123	2.373023	-2.886156
H	2.331121	1.307170	-2.799867
H	1.865136	2.591651	-3.931452
H	3.019839	2.922025	-2.626824
C	-0.362647	-0.358856	-3.577220
H	-0.893232	-1.304121	-3.796304
H	0.483105	-0.360082	-4.290203
C	-1.292506	0.813415	-3.948810
H	-0.714030	1.743043	-4.010754

H -1.735062 0.678799 -4.953615
C -2.439259 1.043561 -2.959336
H -3.029935 0.118739 -2.867689
H -2.009366 1.229404 -1.966264
C -3.368120 2.203246 -3.332257
H -3.857434 1.992328 -4.294886
H -2.759495 3.102867 -3.483841
C -4.435202 2.495375 -2.272687
H -5.067992 1.607060 -2.133685
H -3.941956 2.671714 -1.309326
C -5.313968 3.699171 -2.616131
H -5.854312 3.545087 -3.558116
H -6.057022 3.893899 -1.834291
H -4.709072 4.606425 -2.730486
C 0.693177 2.702138 -0.524281
H 0.403450 1.727566 -0.118083
H 1.445188 3.170769 0.121032
O -0.677324 4.316413 -3.093236

[1]**TS12**-*S*

*w*B97XD/BSI SCF energy in gas:

-2921.28445a.u.

*w*B97XD/BSII SCF energy in solution:

-2921.942373a.u.

*w*B97XD/BSII free energy in solution:

-2920.952871a.u.

C -0.431399 -2.278014 0.371225
N -0.890103 -1.382026 -0.417769
O -1.252204 -2.713001 1.331065
C -2.585005 -2.232888 0.976980
C -2.224804 -1.007579 0.064736
H -2.897460 -0.965609 -0.785925
C -2.221493 0.340646 0.743311
C -3.394820 1.092540 0.723009
C -1.110609 0.826332 1.431789
C -3.464285 2.310829 1.388741
H -4.256347 0.727365 0.169796
C -1.182593 2.041818 2.101083
H -0.174952 0.273503 1.432011
C -2.357440 2.786759 2.082799
H -4.376200 2.897452 1.346666
H -0.305678 2.423895 2.611170
H -2.391563 3.756690 2.567954
C 1.932401 -1.751094 0.204236
N 1.838793 -0.759056 -0.591155
O 3.004691 -1.803654 1.008303
C 3.617887 -0.484954 0.939936
C 3.070868 0.016741 -0.451400
H 2.818332 1.071648 -0.385144
C 4.003676 -0.172351 -1.626749
C 4.992449 0.787706 -1.848400
C 3.936198 -1.283570 -2.464708
C 5.914977 0.630102 -2.874503
H 5.048073 1.657825 -1.199183
C 4.855067 -1.439424 -3.499667
H 3.155370 -2.023151 -2.319084
C 5.849058 -0.487826 -3.703080
H 6.682496 1.381204 -3.030783
H 4.790748 -2.306127 -4.150162
H 6.566786 -0.611787 -4.507681
Ni 0.014278 -0.422859 -1.759123
C 0.943395 -2.894304 0.300302
C 1.017023 -3.733469 -0.997505

H	0.272526	-4.532966	-0.956006
H	0.811274	-3.107898	-1.869821
H	2.010124	-4.181748	-1.093020
C	1.219732	-3.773441	1.524878
H	2.229048	-4.183789	1.465086
H	1.126734	-3.212222	2.456571
H	0.504860	-4.597653	1.552480
C	3.023280	0.349447	2.075814
C	2.459063	-0.275001	3.189280
C	3.043577	1.745930	2.029038
C	1.907311	0.478542	4.221226
H	2.453826	-1.357377	3.246808
C	2.508630	2.500278	3.068452
H	3.477838	2.261403	1.177147
C	1.930881	1.868447	4.165411
H	1.461308	-0.023935	5.073587
H	2.530183	3.583775	3.010509
H	1.497656	2.456147	4.968011
C	5.122482	-0.664595	1.026902
C	5.943265	0.301951	1.606003
C	5.711538	-1.791391	0.446456
C	7.327963	0.153516	1.590013
H	5.509383	1.174941	2.080097
C	7.091604	-1.941152	0.435471
H	5.082993	-2.549768	-0.005354
C	7.906948	-0.965598	1.003788
H	7.952258	0.916101	2.044657
H	7.531663	-2.821594	-0.021561
H	8.986022	-1.081470	0.992801
C	-3.319057	-1.884560	2.257936
C	-4.710009	-1.947954	2.325634
C	-2.605902	-1.417184	3.364285
C	-5.378836	-1.539910	3.476061
H	-5.280254	-2.320203	1.481623
C	-3.274393	-1.015350	4.513428
H	-1.525031	-1.354265	3.317670
C	-4.664170	-1.072286	4.573004
H	-6.462207	-1.593858	3.512975
H	-2.707123	-0.649268	5.363188
H	-5.185677	-0.753789	5.469944
C	-3.255804	-3.351962	0.181039
C	-2.913614	-4.681909	0.436396
C	-4.238448	-3.083142	-0.773622
C	-3.523683	-5.717120	-0.263451
H	-2.166062	-4.904589	1.189602
C	-4.852838	-4.119614	-1.470991
H	-4.542813	-2.062198	-0.981307
C	-4.493463	-5.439772	-1.222939
H	-3.243561	-6.744928	-0.054949
H	-5.609319	-3.887980	-2.213518
H	-4.967961	-6.247931	-1.770083
C	0.353084	1.681009	-1.942851
C	-0.659061	2.709778	-2.086711
N	-0.087343	3.502311	-1.055126
C	-0.581361	4.467405	-0.206415
C	-1.839330	5.049306	-0.436076
C	0.174754	4.885050	0.898454
C	-2.325485	6.003976	0.445772
H	-2.408343	4.724988	-1.299826
C	-0.330003	5.840416	1.772632
H	1.151102	4.441455	1.071011
C	-1.584712	6.406073	1.558830
H	-3.302116	6.443029	0.261639
H	0.266107	6.148311	2.627865

H -1.974201 7.155798 2.240045
C 1.067308 1.063492 -3.122559
H 1.091464 -0.075200 -3.193761
H 0.612335 1.361818 -4.072054
H 2.137906 1.301502 -3.132566
C -1.141137 -1.188436 -3.121837
H -1.721409 -2.012555 -2.685886
H -0.449212 -1.638293 -3.848956
C -2.069021 -0.195415 -3.820075
H -1.511153 0.681763 -4.165614
H -2.493214 -0.669083 -4.720282
C -3.206662 0.307154 -2.938615
H -3.799165 -0.553845 -2.586596
H -2.767965 0.798049 -2.063504
C -4.140324 1.304847 -3.615421
H -4.685291 0.817621 -4.437344
H -3.531308 2.104561 -4.048363
C -5.128607 1.918750 -2.624962
H -5.691118 1.117030 -2.122430
H -4.552756 2.433608 -1.845518
C -6.105114 2.897595 -3.272497
H -6.696627 2.405682 -4.053001
H -6.801572 3.318636 -2.540203
H -5.566026 3.728163 -3.739806
C 1.018227 2.568210 -0.878559
H 1.068702 2.168511 0.143586
H 1.993527 3.011677 -1.147271
O -1.625130 2.963167 -2.792952

^{3}**TS12**-*S*

B3LYP /BSI SCF energy in gas: -2922.095743a.u.

M06/BSII SCF energy in solution: -2920.790517a.u.

M06/BSII free energy in solution: -2919.833356a.u.

C -0.535578 -1.892881 1.142616
N -0.922522 -1.133712 0.184821
O -1.482249 -2.621466 1.779104
C -2.693692 -2.550121 0.923669
C -2.399707 -1.211672 0.136869
H -2.711491 -1.312657 -0.902425
C -3.047122 0.049653 0.688777
C -4.297895 0.441165 0.193244
C -2.442680 0.831332 1.679511
C -4.937284 1.577527 0.686949
H -4.771253 -0.142836 -0.590868
C -3.075957 1.974950 2.168516
H -1.460889 0.561945 2.054394
C -4.326461 2.349459 1.676422
H -5.904704 1.866720 0.286654
H -2.583318 2.586319 2.918268
H -4.809566 3.247621 2.048289
C 1.894191 -1.236527 0.997022
N 1.808086 -0.591705 -0.107755
O 3.084218 -1.196676 1.644080
C 3.915120 -0.183543 0.986005
C 3.130610 0.006559 -0.390134
H 2.980492 1.071984 -0.565068
C 3.776354 -0.577942 -1.635096
C 4.621671 0.231043 -2.404794
C 3.563720 -1.901922 -2.036016
C 5.250922 -0.271424 -3.543739
H 4.788865 1.265648 -2.114375
C 4.187509 -2.405806 -3.177823
H 2.887764 -2.533942 -1.470437

C	5.034773	-1.593865	-3.933467
H	5.901203	0.371882	-4.129400
H	4.003696	-3.432421	-3.481109
H	5.516334	-1.986173	-4.824374
Ni	0.235526	-0.545113	-1.433392
C	0.869023	-2.140806	1.652818
C	1.251886	-3.613997	1.321113
H	0.533710	-4.291898	1.786587
H	1.239526	-3.785352	0.241490
H	2.250073	-3.834330	1.707889
C	0.886268	-1.937758	3.190045
H	1.870983	-2.189282	3.586998
H	0.659786	-0.901168	3.456136
H	0.141734	-2.587456	3.652697
C	3.865494	1.078550	1.859977
C	3.692717	0.956863	3.246202
C	4.048888	2.360054	1.322864
C	3.679288	2.084553	4.066343
H	3.564930	-0.027570	3.681056
C	4.045348	3.490006	2.144900
H	4.205184	2.494291	0.256837
C	3.853426	3.357269	3.519728
H	3.536481	1.966477	5.136667
H	4.191595	4.472449	1.705477
H	3.844684	4.235199	4.158723
C	5.332301	-0.748012	0.876884
C	6.441559	0.100920	0.769871
C	5.544963	-2.132885	0.855706
C	7.728293	-0.421303	0.635151
H	6.309326	1.176506	0.803709
C	6.832397	-2.654290	0.730298
H	4.701274	-2.805913	0.948378
C	7.930315	-1.801155	0.616600
H	8.573815	0.255665	0.553938
H	6.974816	-3.731138	0.722263
H	8.932757	-2.207389	0.518820
C	-3.922260	-2.513742	1.815414
C	-5.183291	-2.772311	1.259012
C	-3.837134	-2.186993	3.172920
C	-6.333810	-2.697881	2.042424
H	-5.263826	-3.041991	0.210307
C	-4.989284	-2.122291	3.958845
H	-2.868175	-1.986853	3.614288
C	-6.240782	-2.374511	3.397541
H	-7.302399	-2.898825	1.593854
H	-4.905010	-1.872016	5.012567
H	-7.136282	-2.322150	4.009925
C	-2.634988	-3.809662	0.045995
C	-2.965001	-5.045572	0.628166
C	-2.172312	-3.797914	-1.277546
C	-2.853947	-6.231212	-0.094182
H	-3.315429	-5.074341	1.654864
C	-2.064874	-4.988931	-2.002559
H	-1.873697	-2.875419	-1.763262
C	-2.406686	-6.206562	-1.417621
H	-3.120462	-7.173881	0.375441
H	-1.710561	-4.953549	-3.028620
H	-2.325818	-7.129168	-1.985367
C	0.721498	3.196023	-2.043205
C	-0.593903	3.829621	-1.944086
N	-0.379619	4.084840	-0.562157
C	-1.072335	4.766108	0.421901
C	-2.328564	5.340384	0.137696
C	-0.525022	4.896879	1.712024

C	-3.010154	6.026978	1.137045
H	-2.738251	5.234127	-0.859785
C	-1.226731	5.587351	2.698951
H	0.443481	4.458845	1.934894
C	-2.471690	6.156424	2.422256
H	-3.975754	6.469787	0.907673
H	-0.792513	5.683602	3.690712
H	-3.012163	6.697209	3.193274
C	1.593888	2.825525	-3.178663
H	1.992965	1.808568	-3.082799
H	1.030340	2.874120	-4.113699
H	2.453812	3.509239	-3.264123
C	-0.280337	-0.648320	-3.349450
H	-0.662300	-1.668521	-3.540514
H	0.691976	-0.628400	-3.873623
C	-1.215916	0.358944	-4.043883
H	-0.808405	1.374223	-3.962247
H	-1.279384	0.151063	-5.127659
C	-2.648403	0.391526	-3.497939
H	-3.083843	-0.619871	-3.559936
H	-2.615680	0.654362	-2.431810
C	-3.559809	1.384004	-4.232518
H	-3.568403	1.143810	-5.306422
H	-3.127831	2.389248	-4.140855
C	-5.003176	1.406938	-3.713964
H	-5.433412	0.396877	-3.784700
H	-4.995903	1.662851	-2.645752
C	-5.901537	2.395049	-4.465836
H	-5.954870	2.147721	-5.532731
H	-6.924899	2.393884	-4.073458
H	-5.513928	3.416891	-4.385091
C	0.970195	3.487294	-0.570609
H	1.075520	2.621759	0.094541
H	1.769573	4.211998	-0.351875
O	-1.531768	4.103892	-2.673876

1a

B3LYP/BSI SCF energy: -443.626262a.u.

M06/BSII SCF energy in solution: -443.389673a.u.

M06/BSII free energy in solution: -443.216755a.u.

C	1.655233	1.068081	-0.292797
H	1.487017	1.536257	0.690403
H	1.725032	1.858538	-1.049802
N	0.694496	0.000117	-0.599971
C	-0.651460	0.000020	-0.266079
C	-1.352677	1.211557	-0.108171
C	-1.352546	-1.211544	-0.108073
C	-2.712329	1.202899	0.194766
H	-0.829428	2.155083	-0.231078
C	-2.712234	-1.202988	0.194843
H	-0.829249	-2.155058	-0.230876
C	-3.405038	-0.000083	0.350263
H	-3.234157	2.149094	0.312360
H	-3.233949	-2.149238	0.312500
H	-4.464274	-0.000110	0.587733
C	3.750188	-0.000133	0.900275
H	4.397128	-0.884333	0.881397
H	4.397401	0.883870	0.881555
H	3.211187	-0.000133	1.854258
C	2.785893	0.000099	-0.276118
H	3.337541	0.000343	-1.221548
C	1.655312	-1.067984	-0.293262
H	1.725148	-1.858034	-1.050705

H 1.487086 -1.536641 0.689711

1a·

B3LYP/BSI SCF energy: -442.959445a.u.

M06/BSII SCF energy in solution: -442.725588a.u.

M06/BSII free energy in solution: -442.568679a.u.

C 1.668739 1.072605 -0.224470

H 1.408824 1.610536 0.707371

H 1.823305 1.824836 -1.013382

N 0.712621 -0.000019 -0.574072

C -0.636812 0.000003 -0.254651

C -1.341579 1.211446 -0.108212

C -1.341603 -1.211438 -0.108226

C -2.704914 1.202745 0.177307

H -0.818547 2.155378 -0.228162

C -2.704929 -1.202723 0.177291

H -0.818577 -2.155371 -0.228198

C -3.399631 0.000020 0.324902

H -3.228166 2.149127 0.286586

H -3.228206 -2.149092 0.286557

H -4.461877 0.000025 0.548386

C 4.050305 0.000027 0.583085

H 4.631894 -0.886320 0.304198

H 4.631803 0.886397 0.304069

H 3.979723 0.000103 1.685445

C 2.718333 -0.000049 -0.064064

C 1.668678 -1.072637 -0.224401

H 1.823193 -1.824979 -1.013227

H 1.408755 -1.610501 0.707490

1b

B3LYP/BSI SCF energy: -517.669019a.u.

M06/BSII SCF energy in solution: -517.43722a.u.

M06/BSII free energy in solution: -517.283454a.u.

C -1.607374 0.769265 -0.228421

C -1.504298 -1.332495 -0.289145

H -1.500628 -1.971230 0.601681

H -1.338279 -1.944386 -1.182331

N -0.595719 -0.179418 -0.184902

O -1.593391 1.979152 -0.163259

C 0.797065 -0.108031 -0.064538

C 1.554602 -1.287959 -0.038946

C 1.437094 1.140205 0.029124

C 2.942507 -1.218928 0.078842

H 1.059960 -2.251864 -0.110751

C 2.823765 1.188317 0.146136

H 0.839527 2.043785 0.010186

C 3.585004 0.016156 0.171653

H 3.521210 -2.138117 0.097805

H 3.314175 2.155036 0.218410

H 4.665676 0.066058 0.263112

C -3.745488 -0.364968 0.732156

H -4.364764 0.536122 0.695500

H -4.401106 -1.234314 0.618138

H -3.282927 -0.413322 1.723683

C -2.689407 -0.322582 -0.366520

H -3.156510 -0.298937 -1.357100

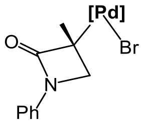

B3LYP/BSI SCF energy: -2656.08749a.u.

M06/BSII SCF energy in solution: -2654.849682a.u.

M06/BSII free energy in solution: -2654.05067a.u.

C	-0.248695	1.952373	0.014822
N	-0.808214	0.813787	0.245363
O	-1.075927	3.010324	-0.122285
C	-2.402024	2.576710	0.365504
C	-2.277934	1.034230	0.128785
C	2.186637	1.229904	-0.188219
N	1.988333	-0.029800	-0.066027
O	3.462482	1.631139	-0.378465
C	4.228452	0.403648	-0.716191
C	3.326521	-0.680609	-0.031317
C	1.201995	2.372200	-0.128838
C	1.346160	3.208898	-1.432869
H	0.696049	4.082975	-1.388661
H	1.079930	2.615557	-2.312484
H	2.381340	3.534010	-1.541395
C	1.564727	3.259129	1.099373
H	2.584843	3.633587	0.994542
H	1.488918	2.690884	2.030673
H	0.876094	4.103676	1.156379
C	5.634141	0.525041	-0.154795
C	6.592638	-0.429900	-0.523810
C	5.998184	1.543503	0.731532
C	7.885241	-0.372359	-0.007043
H	6.327260	-1.215744	-1.224866
C	7.297696	1.605822	1.240288
H	5.267402	2.289611	1.019794
C	8.243652	0.648603	0.876340
H	8.614404	-1.122152	-0.299840
H	7.567039	2.406295	1.923465
H	9.253033	0.697467	1.274262
C	4.222444	0.331058	-2.250973
C	3.408797	-0.550349	-2.976737
C	5.017247	1.248539	-2.958683
C	3.404154	-0.519765	-4.375273
H	2.767853	-1.269416	-2.477678
C	5.006985	1.280656	-4.350624
H	5.651006	1.938205	-2.409872
C	4.200130	0.391642	-5.065979
H	2.772886	-1.217394	-4.917823
H	5.632428	1.995998	-4.877101
H	4.195654	0.409745	-6.152071
C	-2.451180	2.988303	1.846027
C	-2.674959	2.096130	2.901489
C	-2.247490	4.348198	2.140094
C	-2.689933	2.561625	4.222196
H	-2.846576	1.039232	2.726631
C	-2.255599	4.804912	3.454517
H	-2.085265	5.049078	1.326585
C	-2.478354	3.908676	4.504468
H	-2.868537	1.855922	5.028293
H	-2.095667	5.859602	3.660177
H	-2.490575	4.262011	5.531633
C	-3.478487	3.282639	-0.443689
C	-3.202006	3.884460	-1.676241
C	-4.792175	3.308139	0.044899
C	-4.220349	4.502130	-2.404676
H	-2.190406	3.873585	-2.064255
C	-5.809751	3.914816	-0.689519
H	-5.017067	2.859776	1.007845
C	-5.526855	4.517373	-1.916868
H	-3.988494	4.970891	-3.356819

H	-6.822616	3.922904	-0.297416
H	-6.317929	4.997048	-2.485890
C	-1.369484	-2.333280	1.107338
C	-2.821541	-1.915408	1.337454
C	-1.974309	-3.450187	0.194998
H	-1.800970	-4.475284	0.538860
H	-1.749037	-3.368005	-0.868675
N	-3.311319	-2.934853	0.552437
O	-3.418570	-1.085178	2.019025
C	-4.594624	-3.394469	0.248924
C	-4.750178	-4.469046	-0.641086
C	-5.730114	-2.796825	0.826827
C	-6.026417	-4.934160	-0.953183
H	-3.874463	-4.931284	-1.085495
C	-6.997460	-3.274005	0.500044
H	-5.597602	-1.978266	1.523825
C	-7.157165	-4.340777	-0.388681
H	-6.134579	-5.766265	-1.643342
H	-7.869817	-2.808818	0.951184
H	-8.149416	-4.706922	-0.634413
C	-0.755559	-2.826196	2.428127
H	0.197560	-3.331028	2.262863
H	-1.436393	-3.534617	2.926240
H	-0.596215	-1.990539	3.116946
H	-2.780566	0.494031	0.922641
C	-2.810833	0.518892	-1.201448
C	-4.109127	-0.006019	-1.243216
C	-2.064373	0.560666	-2.385773
C	-4.654127	-0.468390	-2.440945
H	-4.689277	-0.069187	-0.327940
C	-2.607388	0.098427	-3.585260
H	-1.046348	0.935402	-2.376047
C	-3.904646	-0.415524	-3.617161
H	-5.657774	-0.882822	-2.448244
H	-2.012178	0.134013	-4.493404
H	-4.324440	-0.781150	-4.549909
H	3.289741	-1.588949	-0.627577
C	3.708700	-1.082432	1.386021
C	4.306396	-2.330984	1.595826
C	3.497593	-0.245196	2.488600
C	4.695066	-2.730598	2.874566
H	4.450231	-3.001929	0.754420
C	3.880996	-0.643597	3.769504
H	3.024883	0.722238	2.354955
C	4.483072	-1.887656	3.966211
H	5.152049	-3.705460	3.017593
H	3.708279	0.018321	4.613489
H	4.778624	-2.199602	4.963828
Br	1.214870	-3.307029	-0.489590
Pd	0.126345	-1.129415	0.300589

^{2}[Pd^I]-Br

B3LYP/BSI SCF energy: -2139.019324a.u.

M06/BSII SCF energy in solution: -2138.005027a.u.

M06/BSII free energy in solution: -2137.376969a.u.

C	1.219063	0.371400	-1.017437
N	1.366689	-0.120170	0.154588
O	2.339668	0.757872	-1.664732
C	3.480385	0.194350	-0.889781
C	2.797807	-0.035008	0.515205
H	3.101804	-0.994197	0.931977
C	3.024806	1.016354	1.588889
C	3.770009	0.677870	2.723935

C	2.496260	2.309989	1.489618
C	3.995905	1.614537	3.734057
H	4.158928	-0.331466	2.826305
C	2.719175	3.247089	2.497406
H	1.903606	2.589584	0.623755
C	3.472249	2.902433	3.622397
H	4.570900	1.332048	4.611094
H	2.303201	4.246525	2.405624
H	3.642405	3.631869	4.409161
C	-1.305973	0.467180	-0.946658
N	-1.517149	-0.252078	0.091900
O	-2.335422	1.224466	-1.379844
C	-3.445386	1.099271	-0.426880
C	-2.897292	-0.025256	0.566039
H	-2.818490	0.401903	1.567088
C	-3.674357	-1.322658	0.678429
C	-4.393291	-1.593082	1.848133
C	-3.677690	-2.271905	-0.352180
C	-5.113960	-2.780977	1.985085
H	-4.377563	-0.877455	2.666918
C	-4.393156	-3.460172	-0.216792
H	-3.113110	-2.085750	-1.260133
C	-5.115600	-3.716939	0.951131
H	-5.661098	-2.977929	2.902267
H	-4.382981	-4.189280	-1.021631
H	-5.668607	-4.645606	1.056709
C	-0.062579	0.505075	-1.826767
C	-0.127512	-0.712718	-2.797044
H	0.765771	-0.726520	-3.426279
H	-0.180078	-1.649657	-2.237383
H	-1.007685	-0.628701	-3.441436
C	-0.018668	1.818848	-2.641937
H	-0.904659	1.897722	-3.273379
H	0.014354	2.695044	-1.988400
H	0.868861	1.824019	-3.275364
C	-3.609036	2.464669	0.252730
C	-3.340596	3.632893	-0.475377
C	-4.084532	2.588541	1.564353
C	-3.523608	4.888547	0.101324
H	-2.982557	3.554803	-1.495752
C	-4.276207	3.847212	2.139161
H	-4.314818	1.706979	2.154452
C	-3.992291	5.001751	1.411826
H	-3.301743	5.781013	-0.476822
H	-4.642343	3.918936	3.159047
H	-4.134621	5.980353	1.860474
C	-4.702727	0.733075	-1.219937
C	-5.956703	0.753532	-0.593348
C	-4.633492	0.371559	-2.570192
C	-7.108723	0.406515	-1.295833
H	-6.039377	1.048042	0.447223
C	-5.790053	0.031962	-3.275881
H	-3.675870	0.365023	-3.076608
C	-7.031190	0.044499	-2.642222
H	-8.069676	0.425143	-0.790107
H	-5.715202	-0.241222	-4.324637
H	-7.930147	-0.220794	-3.190737
C	4.620573	1.197764	-0.895095
C	5.849309	0.822143	-0.332701
C	4.486183	2.481167	-1.433342
C	6.916561	1.716689	-0.300238
H	5.972146	-0.177563	0.073680
C	5.560673	3.374151	-1.408135
H	3.543387	2.779558	-1.876454

C	6.776761	2.997539	-0.840455
H	7.859976	1.410906	0.142573
H	5.442100	4.366196	-1.834823
H	7.610651	3.693136	-0.820352
C	3.851959	-1.106960	-1.616727
C	4.540813	-1.009476	-2.838198
C	3.463878	-2.376831	-1.166024
C	4.842140	-2.146307	-3.583490
H	4.845530	-0.033470	-3.202374
C	3.773050	-3.517591	-1.914171
H	2.918356	-2.514367	-0.238030
C	4.461102	-3.408786	-3.120746
H	5.379436	-2.046949	-4.522489
H	3.468901	-4.490530	-1.539846
H	4.701558	-4.298061	-3.696510
Br	1.365725	-3.153495	2.311140
Pd	-0.086883	-1.477468	1.102602

^{2}TS13

B3LYP/BSI SCF energy: -5377.934524a.u.

M06/BSII SCF energy in solution: -5376.837258a.u.

M06/BSII free energy in solution: -5375.99452a.u.

C	-1.117930	1.865566	-1.273914
N	-0.454055	1.272614	-0.348062
O	-0.594660	3.013441	-1.759429
C	0.792586	3.070849	-1.239193
C	0.648742	2.195519	0.051394
H	1.542091	1.607560	0.246684
C	0.273039	2.914782	1.341127
C	1.089490	2.745080	2.466427
C	-0.874040	3.714541	1.450909
C	0.770385	3.372211	3.673738
H	1.963398	2.104864	2.385049
C	-1.191101	4.339770	2.655440
H	-1.521046	3.863395	0.592115
C	-0.368304	4.170970	3.772047
H	1.412851	3.230120	4.538467
H	-2.082159	4.957603	2.722770
H	-0.616955	4.657984	4.711011
C	-3.183939	0.513829	-0.961346
N	-2.688546	-0.449044	-0.279463
O	-4.516896	0.714884	-0.869093
C	-4.988797	-0.093379	0.267307
C	-3.803830	-1.143418	0.402735
H	-3.531947	-1.260215	1.451018
C	-4.058288	-2.525218	-0.172053
C	-4.287877	-3.595528	0.698213
C	-4.089783	-2.759585	-1.551154
C	-4.551051	-4.873953	0.204968
H	-4.249051	-3.432314	1.772529
C	-4.351015	-4.035010	-2.048399
H	-3.893106	-1.947363	-2.243619
C	-4.583975	-5.096675	-1.171670
H	-4.721065	-5.694618	0.895882
H	-4.367222	-4.202118	-3.121659
H	-4.782906	-6.091363	-1.559848
C	-2.423802	1.421254	-1.918168
C	-2.091547	0.613611	-3.206333
H	-1.465523	1.215346	-3.870771
H	-1.568194	-0.311967	-2.954452
H	-3.015343	0.359151	-3.734862
C	-3.268879	2.660220	-2.288690
H	-4.201769	2.344921	-2.759252

H	-3.516838	3.255595	-1.406476
H	-2.714868	3.288513	-2.987115
Ni	-0.652367	-0.680326	0.025434
Br	-0.318945	-3.092387	-0.363183
Si	3.166649	-0.874521	1.231634
H	1.706425	-0.432245	0.844642
O	4.767316	-1.219899	1.925305
O	2.448995	-2.023077	2.337459
C	5.120838	-2.191592	2.882957
H	5.460176	-3.111528	2.373186
H	4.257852	-2.470812	3.493806
C	6.226862	-1.673672	3.808035
H	6.505121	-2.439993	4.541375
H	7.142922	-1.394023	3.266642
H	5.892076	-0.782607	4.348640
C	1.180526	-1.893552	2.941903
H	0.508583	-1.285021	2.314290
H	0.724763	-2.892293	3.006441
C	1.274888	-1.276423	4.336430
H	1.669115	-0.256137	4.281776
H	0.285035	-1.233806	4.807302
H	1.937489	-1.863559	4.981704
O	3.484972	0.834061	1.310147
C	4.679348	1.510465	1.677961
H	4.384511	2.466765	2.136855
H	5.222375	0.933106	2.430770
C	5.587496	1.782541	0.476051
H	5.749353	0.867912	-0.104588
H	5.143583	2.513412	-0.208395
H	6.554041	2.186141	0.806561
C	-6.356622	-0.667481	-0.090306
C	-7.131632	-1.285671	0.900484

C	-6.861092	-0.598629	-1.393768
C	-8.375815	-1.832290	0.592044
H	-6.769083	-1.332229	1.922129
C	-8.112415	-1.137475	-1.699456
H	-6.277490	-0.117219	-2.169050
C	-8.873322	-1.758833	-0.710354
H	-8.959687	-2.309879	1.373511
H	-8.489796	-1.069476	-2.715878
H	-9.845998	-2.178611	-0.949434
C	-5.092612	0.858268	1.470742
C	-5.508537	2.181079	1.254366
C	-4.857486	0.440909	2.786545
C	-5.661788	3.064973	2.320470
H	-5.711885	2.515235	0.242977
C	-5.017169	1.325486	3.856518
H	-4.550056	-0.577643	3.000291
C	-5.414737	2.641192	3.628041
H	-5.980457	4.085777	2.129149
H	-4.823028	0.980392	4.867752
H	-5.533895	3.329410	4.459556
C	1.181464	4.525796	-1.033256
C	2.420840	4.818737	-0.446578
C	0.357006	5.579597	-1.440108
C	2.821057	6.140383	-0.263519
H	3.074201	4.011762	-0.129168
C	0.763068	6.904134	-1.259517
H	-0.603303	5.366167	-1.894532
C	1.993745	7.189845	-0.670850
H	3.781846	6.350016	0.197533
H	0.111424	7.711897	-1.580912
H	2.307991	8.219961	-0.530301
C	1.687272	2.416621	-2.310317

C	1.417875	2.664568	-3.666647
C	2.791413	1.618792	-1.979407
C	2.218535	2.106579	-4.663275
H	0.580037	3.298065	-3.939625
C	3.602421	1.059203	-2.978113
H	3.039454	1.403278	-0.945595
C	3.309618	1.298334	-4.323085
H	1.994110	2.309448	-5.707297
H	4.429774	0.409809	-2.684747
H	3.931691	0.862599	-5.100107
O	3.714683	-3.032253	-2.271815
P	4.600673	-2.389840	-1.192704
O	5.201855	-3.409800	-0.192666
O	5.589653	-1.293886	-1.620828
O	3.389883	-1.574900	-0.291071
K	1.726835	-1.475375	-2.397194
K	7.052749	-1.712311	0.431888
K	2.715762	-4.191423	0.370911

^{2}TS13-Br

B3LYP/BSI SCF energy: -2712.507852a.u.

M06/BSII SCF energy in solution: -2711.2742a.u.

M06/BSII free energy in solution: -2710.483708a.u.

C	0.375327	2.181132	0.303344
N	-0.290696	1.144729	-0.050684
O	-0.336880	3.253700	0.689113
C	-1.747753	2.834850	0.825081
C	-1.738923	1.452288	0.051502
C	2.681052	1.142921	0.099544
N	2.272259	-0.037503	-0.188245
O	4.011093	1.315031	0.208764
C	4.645045	0.086148	-0.348993
C	3.451431	-0.937218	-0.235925
Ni	0.404303	-0.602910	-0.746905
C	1.876328	2.410826	0.308521
C	2.219652	3.393954	-0.848606
H	1.683375	4.334438	-0.704837
H	1.942528	2.971380	-1.818276
H	3.292923	3.593095	-0.852567
C	2.288589	3.038065	1.667676
H	3.364573	3.218613	1.677196
H	2.032927	2.377196	2.500877
H	1.768353	3.985891	1.809816
C	5.866531	-0.257908	0.485134
C	6.654294	-1.353219	0.101780
C	6.233342	0.477729	1.615806
C	7.777520	-1.711375	0.843479
H	6.390537	-1.924121	-0.783800
C	7.365042	0.121659	2.354414
H	5.636699	1.330228	1.918242
C	8.138147	-0.973764	1.974067
H	8.373672	-2.565443	0.535805
H	7.638724	0.705308	3.228800
H	9.016283	-1.250902	2.549884
C	5.012850	0.449054	-1.795887
C	4.194557	0.129157	-2.889826
C	6.169276	1.213664	-2.023573
C	4.537944	0.551969	-4.177459
H	3.283953	-0.449541	-2.771577
C	6.506209	1.637119	-3.307099
H	6.811742	1.472510	-1.188303
C	5.691563	1.303836	-4.391917
H	3.893680	0.284770	-5.009886

H	7.408199	2.222859	-3.460133
H	5.956582	1.626726	-5.394668
C	-2.016743	2.694249	2.331497
C	-2.865437	1.712704	2.857705
C	-1.447571	3.634634	3.205030
C	-3.122392	1.667569	4.231822
H	-3.346746	0.967991	2.232080
C	-1.698459	3.579704	4.574138
H	-0.806622	4.414286	2.807260
C	-2.538288	2.592140	5.094951
H	-3.785446	0.897698	4.615357
H	-1.242840	4.313199	5.233558
H	-2.737459	2.549676	6.161963
C	-2.628878	3.925498	0.222233
C	-2.089319	5.008516	-0.479434
C	-4.020025	3.844097	0.376323
C	-2.924252	5.989535	-1.019731
H	-1.015714	5.091732	-0.599539
C	-4.851516	4.818295	-0.171627
H	-4.455840	3.019935	0.931785
C	-4.306947	5.897480	-0.871241
H	-2.487564	6.826420	-1.557536
H	-5.927165	4.735649	-0.046299
H	-4.955526	6.659666	-1.293145
C	-3.067458	-2.932939	2.030112
C	-4.138904	-2.063483	1.483450
C	-3.572424	-4.056778	1.123070
H	-4.013632	-4.919540	1.640510
H	-2.874731	-4.390921	0.349951
N	-4.582820	-3.087246	0.644471
O	-4.538626	-0.914902	1.635572
C	-5.570234	-3.185312	-0.330903
C	-5.676275	-4.353074	-1.104142
C	-6.472724	-2.124123	-0.539074
C	-6.666338	-4.452025	-2.079655
H	-4.980953	-5.171350	-0.945833
C	-7.453968	-2.242561	-1.520003
H	-6.391340	-1.234947	0.074398
C	-7.559273	-3.400907	-2.295911
H	-6.736033	-5.357648	-2.675442
H	-8.148920	-1.421534	-1.674003
H	-8.328795	-3.483236	-3.057292
C	-2.550142	-3.022234	3.421781
H	-2.328198	-2.026649	3.813811
H	-1.638566	-3.624282	3.472300
H	-3.299152	-3.488897	4.081472
H	-2.182330	0.677044	0.673794
C	-2.406003	1.390413	-1.310823
C	-3.596354	0.666174	-1.446943
C	-1.855883	2.014364	-2.438477
C	-4.229078	0.570169	-2.687495
H	-4.021773	0.167121	-0.580617
C	-2.487392	1.918437	-3.677773
H	-0.928671	2.573882	-2.354682
C	-3.675696	1.195346	-3.805075
H	-5.143959	-0.008176	-2.777208
H	-2.048817	2.403417	-4.545170
H	-4.161628	1.114114	-4.773173
H	3.380575	-1.535829	-1.142735
C	3.442685	-1.893969	0.944083
C	3.494279	-3.270534	0.699496
C	3.354176	-1.444491	2.268139
C	3.474259	-4.182538	1.756166
H	3.529484	-3.630801	-0.324807

C	3.333792	-2.352696	3.324685
H	3.302213	-0.380802	2.479688
C	3.396201	-3.725368	3.071414
H	3.508847	-5.247953	1.548315
H	3.266766	-1.989634	4.346268
H	3.376947	-4.432733	3.895599
Br	0.849793	-2.134312	-2.523504
Br	-1.029798	-1.983745	0.765408

K-^{2}TS13-Br

B3LYP/BSI SCF energy: -5154.763112a.u.

M06/BSII SCF energy in solution: -5153.578684a.u.

M06/BSII free energy in solution: -5152.783033a.u.

C	-1.157243	2.019029	-0.422541
N	-0.687163	0.990043	0.183148
O	-0.272381	2.935726	-0.847452
C	1.093820	2.394862	-0.638182
C	0.787528	1.125220	0.282972
C	-3.601386	1.304826	-0.438697
N	-3.409053	0.146505	0.073469
O	-4.872680	1.627374	-0.752902
C	-5.739299	0.611404	-0.094806
C	-4.710130	-0.565699	0.106877
Ni	-1.763712	-0.484420	1.050010
C	-2.596861	2.401233	-0.730631
C	-2.962934	3.637380	0.141192
H	-2.285927	4.463103	-0.086602
H	-2.886816	3.401511	1.206290
H	-3.987939	3.945559	-0.072016
C	-2.704585	2.771010	-2.235971
H	-3.726597	3.076780	-2.466197
H	-2.440314	1.919100	-2.869455
H	-2.024337	3.592093	-2.464544
C	-6.909295	0.292427	-1.009127
C	-7.884464	-0.609559	-0.559052
C	-7.053164	0.869908	-2.274050
C	-8.972608	-0.936604	-1.364425
H	-7.794114	-1.052234	0.428662
C	-8.149593	0.546220	-3.078196
H	-6.310468	1.574664	-2.629077
C	-9.110037	-0.358330	-2.629044
H	-9.715961	-1.640826	-1.002387
H	-8.249025	1.005881	-4.057505
H	-9.960818	-0.609998	-3.255472
C	-6.197928	1.266892	1.217893
C	-5.578962	1.016425	2.451560
C	-7.220480	2.229135	1.162856
C	-5.986777	1.703011	3.599560
H	-4.776207	0.293167	2.553767
C	-7.621199	2.914345	2.307255
H	-7.707886	2.437814	0.216039
C	-7.006304	2.650618	3.533773
H	-5.497568	1.485953	4.544510
H	-8.417504	3.650444	2.241747
H	-7.322227	3.178717	4.429003
C	1.558172	2.004714	-2.051207
C	0.973757	0.900960	-2.700676
C	2.469329	2.790078	-2.768003
C	1.277446	0.612049	-4.032562
H	0.273072	0.257235	-2.178715
C	2.789212	2.490815	-4.095217
H	2.940635	3.637812	-2.285947
C	2.187045	1.407586	-4.738001

H	0.805183	-0.239812	-4.513999
H	3.512702	3.105326	-4.622093
H	2.424898	1.182811	-5.774203
C	1.935160	3.478082	0.023897
C	1.403131	4.745019	0.288637
C	3.257646	3.189372	0.406120
C	2.164494	5.717535	0.942634
H	0.387525	4.974429	-0.010521
C	3.994773	4.160575	1.088476
H	3.721843	2.218560	0.183391
C	3.461259	5.424298	1.358525
H	1.732558	6.694532	1.140299
H	4.982407	3.905964	1.469900
H	4.044787	6.164861	1.899203
C	0.928707	-3.999981	-0.814089
C	2.217791	-3.229786	-0.869032
C	1.425741	-4.591814	0.515643
H	1.647590	-5.666329	0.481637
H	0.830358	-4.353519	1.401163
N	2.624551	-3.739506	0.354358
O	2.724856	-2.446295	-1.658547
C	3.658996	-3.467166	1.263894
C	3.819350	-4.307088	2.379164
C	4.497092	-2.354851	1.093938
C	4.804078	-4.027850	3.325390
H	3.168383	-5.166180	2.510660
C	5.462951	-2.077795	2.063417
H	4.408535	-1.677834	0.251750
C	5.628273	-2.908703	3.176973
H	4.912837	-4.678337	4.188810
H	6.045549	-1.166215	1.940010
H	6.372870	-2.676549	3.934374
C	0.382227	-4.811097	-1.949051
H	0.244812	-4.188888	-2.837112
H	-0.580085	-5.261222	-1.690058
H	1.081607	-5.621117	-2.204264
H	1.240626	0.244840	-0.177446
C	1.232871	1.162783	1.736098
C	2.473042	0.605624	2.080438
C	0.427717	1.720167	2.737884
C	2.888881	0.605173	3.412813
H	3.141830	0.223029	1.310116
C	0.847631	1.714628	4.068568
H	-0.540394	2.143864	2.489066
C	2.078859	1.152756	4.409458
H	3.847838	0.162797	3.665704
H	0.206267	2.136924	4.836816
H	2.401458	1.137495	5.447004
H	-4.825394	-1.005775	1.096586
C	-4.728383	-1.700049	-0.903721
C	-5.017714	-2.996329	-0.464412
C	-4.444307	-1.493365	-2.260152
C	-5.038600	-4.064916	-1.362775
H	-5.209026	-3.173340	0.590305
C	-4.463839	-2.557985	-3.158962
H	-4.208235	-0.496588	-2.620363
C	-4.763985	-3.847866	-2.712940
H	-5.261332	-5.065489	-1.003651
H	-4.244980	-2.381704	-4.208381
H	-4.778669	-4.677390	-3.414238
Br	-2.574528	-1.635268	3.000893
Br	-0.627398	-2.410116	-0.337315
O	4.738254	0.532582	-0.244477
P	6.156113	0.308879	-0.893263

O	6.292097	-1.188948	-1.350344
O	7.305421	0.655858	0.146387
O	6.256751	1.268400	-2.137717
K	8.258841	-1.693010	0.039522
K	6.844385	3.105983	-0.540958
K	4.126615	-0.472432	-2.744768

^{2}TS13-OA

B3LYP/BSI SCF energy: -2712.491152a.u.

M06/BSII SCF energy in solution: -2711.268108a.u.

M06/BSII free energy in solution: -2710.473766a.u.

C	1.802573	-1.574588	-0.718865
N	1.731955	-0.419652	-0.157183
O	3.028775	-2.096796	-0.867965
C	3.946968	-1.287966	-0.013571
C	3.120127	0.044089	0.119020
C	-0.693860	-2.046027	-0.776198
N	-1.063873	-1.025439	-0.085893
O	-1.650509	-2.921361	-1.111545
C	-2.960260	-2.273770	-0.797295
C	-2.504515	-1.213441	0.262140
Ni	0.073721	0.454058	0.652372
C	0.685101	-2.434232	-1.282584
C	0.696131	-2.293714	-2.833425
H	1.656560	-2.635990	-3.226316
H	0.536103	-1.257400	-3.139457
H	-0.100809	-2.906801	-3.258973
C	0.945070	-3.915863	-0.894814
H	0.166023	-4.547883	-1.321988
H	0.949729	-4.045500	0.191331
H	1.914922	-4.231734	-1.279259
C	-3.921396	-3.334146	-0.285988
C	-5.242835	-2.954638	-0.009866
C	-3.542465	-4.665581	-0.090736
C	-6.160899	-3.886782	0.467540
H	-5.555817	-1.928379	-0.178627
C	-4.468166	-5.601363	0.378703
H	-2.526732	-4.974486	-0.308676
C	-5.776989	-5.215915	0.662942
H	-7.179223	-3.575516	0.680821
H	-4.160296	-6.633424	0.520769
H	-6.494830	-5.943751	1.029471
C	-3.447993	-1.675872	-2.125426
C	-3.633697	-0.305398	-2.347066
C	-3.690597	-2.570205	-3.183459
C	-4.045485	0.160817	-3.601834
H	-3.464067	0.432641	-1.570708
C	-4.098176	-2.105155	-4.429999
H	-3.564030	-3.636488	-3.021082
C	-4.276974	-0.734344	-4.643178
H	-4.159935	1.230952	-3.745955
H	-4.281781	-2.812353	-5.234129
H	-4.595433	-0.369646	-5.615496
C	4.065014	-2.076126	1.300327
C	3.311704	-1.771167	2.443916
C	4.900869	-3.205133	1.329409
C	3.405301	-2.569674	3.587825
H	2.644927	-0.916124	2.477268
C	4.988772	-4.001229	2.468716
H	5.490342	-3.457512	0.454057
C	4.241932	-3.683934	3.605886
H	2.819086	-2.308099	4.463667
H	5.645405	-4.866478	2.469797

H	4.315381	-4.299289	4.497896
C	5.284658	-1.152911	-0.719275
C	5.531288	-1.715538	-1.975144
C	6.311645	-0.447421	-0.075481
C	6.781774	-1.567178	-2.581662
H	4.750118	-2.273591	-2.478076
C	7.554740	-0.294802	-0.684631
H	6.139706	-0.021901	0.909006
C	7.794725	-0.855151	-1.941979
H	6.959694	-2.012219	-3.556410
H	8.337746	0.258849	-0.175105
H	8.765214	-0.739387	-2.415254
H	3.152530	0.407267	1.144564
C	3.494589	1.206330	-0.784807
C	3.864337	2.424424	-0.201665
C	3.460885	1.109020	-2.183077
C	4.198612	3.522769	-0.997273
H	3.876532	2.516892	0.880840
C	3.792676	2.204447	-2.978735
H	3.182679	0.172682	-2.657119
C	4.162373	3.415312	-2.387484
H	4.480678	4.460985	-0.528607
H	3.762396	2.113942	-4.060545
H	4.417076	4.268892	-3.008609
H	-3.005996	-0.263427	0.108037
C	-2.638125	-1.567197	1.735015
C	-3.412645	-0.740242	2.557444
C	-1.983201	-2.668084	2.306173
C	-3.542300	-1.014567	3.920617
H	-3.893023	0.134738	2.130585
C	-2.111710	-2.942423	3.666310
H	-1.375724	-3.322623	1.688242
C	-2.893675	-2.116103	4.477609
H	-4.141355	-0.359457	4.546089
H	-1.602300	-3.801488	4.093578
H	-2.990035	-2.328116	5.538487
Br	-2.470066	3.782782	-2.692018
C	-1.717487	2.449165	0.001907
Br	0.884060	1.571262	2.591276
C	-0.273160	1.513193	-2.213938
H	0.652157	0.954587	-2.043327
H	-1.077955	0.822988	-2.469981
H	-0.105383	2.171956	-3.069176
C	-0.634693	2.328554	-1.017649
C	-1.513807	4.527890	1.470505
C	-0.699626	5.648345	1.711571
C	-2.705886	4.355838	2.200706
C	-1.074421	6.582287	2.673826
H	0.216784	5.781116	1.145931
C	-3.061991	5.300981	3.156747
H	-3.327120	3.492242	2.000385
C	-2.254454	6.415984	3.401742
H	-0.440027	7.445558	2.853062
H	-3.983529	5.166123	3.715951
H	-2.543163	7.147458	4.150351
N	-1.134648	3.602390	0.511366
C	-0.010477	3.603544	-0.445474
H	0.966936	3.485051	0.032017
H	-0.022577	4.454009	-1.133027
O	-2.702638	1.809811	0.360897

^{2}[Ni^{I}]H

B3LYP/BSI SCF energy: -2169.192185a.u.

M06/BSII SCF energy in solution: -2168.244062a.u.

M06/BSII free energy in solution: -2167.606839a.u.

C	1.318891	-0.208039	-0.674260
N	1.410338	-0.121256	0.599175
O	2.475244	-0.298286	-1.375069
C	3.537631	-0.572809	-0.372948
C	2.843578	-0.043309	0.944850
H	3.031641	-0.729241	1.770051
C	3.216131	1.352345	1.417081
C	3.976083	1.499376	2.583082
C	2.813697	2.503320	0.727568
C	4.340222	2.765273	3.044426
H	4.274043	0.615803	3.141272
C	3.174756	3.769430	1.185893
H	2.208593	2.412904	-0.169152
C	3.941816	3.904567	2.345162
H	4.925753	2.859453	3.954495
H	2.853518	4.651885	0.639598
H	4.219435	4.891524	2.704184
C	-1.191547	-0.028750	-0.698590
N	-1.374563	-0.223133	0.554977
O	-2.267928	0.368942	-1.421639
C	-3.341601	0.695293	-0.480350
C	-2.799930	0.032923	0.860848
H	-2.827703	0.770133	1.663056
C	-3.496776	-1.220021	1.358834
C	-4.267904	-1.159180	2.524452
C	-3.384370	-2.444179	0.687104
C	-4.923677	-2.292640	3.007953
H	-4.345631	-0.220689	3.068165
C	-4.036427	-3.578013	1.167872
H	-2.777981	-2.514921	-0.210383
C	-4.810064	-3.505301	2.328775
H	-5.512396	-2.228520	3.918419
H	-3.936823	-4.521336	0.638683
H	-5.313734	-4.391101	2.704741
C	0.061504	-0.282193	-1.518967
C	-0.036489	-1.719501	-2.110398
H	0.849160	-1.929679	-2.713996
H	-0.098535	-2.462016	-1.310636
H	-0.923939	-1.801085	-2.744051
C	0.166618	0.749584	-2.669420
H	-0.692213	0.657183	-3.336177
H	0.191595	1.772814	-2.283363
H	1.078525	0.568172	-3.240597
Ni	-0.017090	-0.763271	1.927183
H	0.674516	-1.375923	3.179123
C	-4.647945	0.124639	-1.032483
C	-5.860834	0.406139	-0.388369
C	-4.667226	-0.673948	-2.181201
C	-7.060363	-0.110347	-0.873360
H	-5.873824	1.037664	0.493687
C	-5.871420	-1.184468	-2.671531
H	-3.739944	-0.891880	-2.696828
C	-7.071675	-0.908287	-2.019351
H	-7.988503	0.115727	-0.356494
H	-5.865793	-1.799437	-3.567109
H	-8.007616	-1.306584	-2.400004
C	-3.417426	2.228911	-0.396422
C	-3.119751	2.993029	-1.534464
C	-3.843118	2.894513	0.760145
C	-3.225536	4.382544	-1.508925
H	-2.800852	2.493055	-2.441973
C	-3.955759	4.286671	0.784040

H	-4.093471	2.339593	1.658819
C	-3.643445	5.036765	-0.348322
H	-2.983305	4.954914	-2.399938
H	-4.282536	4.780574	1.694421
H	-3.725068	6.119484	-0.327921
C	4.799086	0.171020	-0.774163
C	5.974918	-0.046945	-0.040856
C	4.824891	1.077347	-1.838439
C	7.147064	0.634691	-0.360130
H	5.973230	-0.759638	0.778695
C	6.003075	1.755231	-2.162184
H	3.923927	1.248684	-2.415322
C	7.166007	1.539351	-1.424580
H	8.047363	0.455997	0.220670
H	6.007489	2.452881	-2.994993
H	8.080912	2.067633	-1.676716
C	3.723014	-2.098278	-0.388361
C	4.502021	-2.674718	-1.405192
C	3.066500	-2.948161	0.515471
C	4.636005	-4.057570	-1.509204
H	5.010730	-2.032699	-2.116858
C	3.205912	-4.335109	0.410646
H	2.429800	-2.556366	1.303606
C	3.990292	-4.894809	-0.596514
H	5.248482	-4.480209	-2.300773
H	2.693517	-4.972812	1.125163
H	4.098611	-5.973207	-0.671390

^{2}TS14

B3LYP/BSI SCF energy: -2405.032621a.u.

M06/BSII SCF energy in solution: -2403.947505a.u.

M06/BSII free energy in solution: -2403.155597a.u.

C	-1.460664	-0.522886	-1.126026
N	-1.412601	0.403690	-0.240669
O	-2.671885	-1.070407	-1.391620
C	-3.669390	-0.198778	-0.730142
C	-2.760083	0.526310	0.346644
C	0.969776	-0.949227	-1.144227
N	1.346972	0.047062	-0.431944
O	1.792931	-2.029273	-1.183070
C	2.782164	-1.845620	-0.116327
C	2.666668	-0.276229	0.142112
Ni	0.206909	1.655086	0.083978
C	-0.299784	-1.016301	-1.977402
C	-0.564014	-2.450403	-2.477247
H	-1.474726	-2.468825	-3.077634
H	-0.683228	-3.151354	-1.647179
H	0.272221	-2.790329	-3.091322
C	-0.158392	-0.047530	-3.187336
H	0.645480	-0.391742	-3.845817
H	0.068799	0.963852	-2.840052
H	-1.090683	-0.029813	-3.758930
C	4.130845	-2.340905	-0.632739
C	5.160948	-2.662026	0.261363
C	4.374114	-2.459394	-2.006909
C	6.405521	-3.082052	-0.207028
H	4.990387	-2.598242	1.330725
C	5.616540	-2.889212	-2.474416
H	3.585076	-2.223866	-2.710632
C	6.638688	-3.199833	-1.577710
H	7.190436	-3.325353	0.503380
H	5.782963	-2.980366	-3.544094
H	7.605693	-3.533812	-1.942478

C	2.296811	-2.676087	1.083871
C	2.562195	-2.311403	2.409962
C	1.611047	-3.877581	0.848162
C	2.142631	-3.116347	3.472279
H	3.099167	-1.395796	2.635864
C	1.185375	-4.677464	1.907410
H	1.410797	-4.181561	-0.173066
C	1.448402	-4.299735	3.226191
H	2.356192	-2.808949	4.491779
H	0.651541	-5.600922	1.701196
H	1.119582	-4.924134	4.051843
C	-4.174332	0.743228	-1.834559
C	-3.618987	2.012289	-2.057138
C	-5.158506	0.282678	-2.724598
C	-4.051535	2.802861	-3.125188
H	-2.835488	2.398616	-1.414703
C	-5.587813	1.070278	-3.790758
H	-5.593378	-0.700209	-2.576908
C	-5.038219	2.338084	-3.993010
H	-3.610212	3.784143	-3.273866
H	-6.354246	0.693585	-4.462188
H	-5.375672	2.956011	-4.820199
C	-4.774711	-1.065423	-0.152085
C	-4.673983	-2.459182	-0.100752
C	-5.923167	-0.449784	0.367114
C	-5.701192	-3.222559	0.459549
H	-3.794274	-2.944966	-0.505552
C	-6.943709	-1.211547	0.932064
H	-6.022568	0.630746	0.319636
C	-6.836958	-2.603563	0.979542
H	-5.609362	-4.304712	0.487048
H	-7.824683	-0.717501	1.331649
H	-7.634245	-3.198386	1.415706
C	0.946364	3.405935	-0.682896
H	0.477698	3.689299	-1.623415
H	2.034165	3.445647	-0.682968
C	0.252393	3.675980	0.548657
H	-0.319045	2.412353	1.359921
H	-0.732334	4.137098	0.439572
C	1.022289	4.251300	1.735883
H	1.954702	3.686278	1.864128
H	0.446621	4.114158	2.660698
C	1.350671	5.741932	1.550435
H	1.939590	5.870306	0.633602
H	0.416239	6.301447	1.393131
C	2.107167	6.350511	2.738049
H	3.036943	5.787612	2.900374
H	1.512722	6.221625	3.653185
C	2.437439	7.834919	2.549463
H	3.060354	7.991254	1.661113
H	2.978790	8.240894	3.411040
H	1.525889	8.429997	2.419443
C	3.777522	0.591103	-0.423845
C	3.773369	1.033437	-1.751680
C	4.847317	0.953882	0.403172
C	4.821381	1.811739	-2.242786
H	2.934488	0.792294	-2.395577
C	5.898690	1.730107	-0.085686
H	4.857173	0.630850	1.441556
C	5.889090	2.159996	-1.413012
H	4.799374	2.154086	-3.273561
H	6.718473	2.004487	0.572106
H	6.702386	2.769784	-1.795878
C	-2.800098	-0.031503	1.760695

C -3.538211 0.639588 2.742386
C -2.120164 -1.202938 2.114262
C -3.606661 0.148519 4.046487
H -4.056073 1.560603 2.486386
C -2.182511 -1.694661 3.417763
H -1.521666 -1.726282 1.375809
C -2.928136 -1.022034 4.387908
H -4.180841 0.685902 4.795939
H -1.637040 -2.598182 3.674977
H -2.973022 -1.402771 5.404389
H -3.030473 1.581182 0.404339
H 2.611693 -0.091061 1.214388

²IM8-*R*

B3LYP/BSI SCF energy: -2935.508065a.u.

M06/BSII SCF energy in solution: -2934.175118a.u.

M06/BSII free energy in solution: -2933.217466a.u.

C -1.594659 -0.965162 -1.623512
N -1.639040 -0.380498 -0.483247
O -2.773959 -1.239161 -2.215363
C -3.825006 -0.519453 -1.473398
C -3.064485 -0.224804 -0.110587
C 0.920789 -1.282053 -1.700286
N 1.228110 -0.536138 -0.702861
O 1.910354 -2.024135 -2.230301
C 3.033563 -1.997440 -1.268878
C 2.691950 -0.685564 -0.459682
Ni -0.038583 0.768034 0.276976
C -0.396576 -1.400567 -2.446545
C -0.615588 -2.873582 -2.884599
H -1.555524 -2.958160 -3.431141
H -0.647094 -3.545009 -2.021579
H 0.202112 -3.191736 -3.531278
C -0.306998 -0.489954 -3.707723
H 0.518759 -0.825173 -4.340315
H -0.141026 0.552634 -3.427742
H -1.235746 -0.557680 -4.277448
C 4.344179 -1.977054 -2.044513
C 5.550301 -2.000886 -1.330514
C 4.383211 -1.965673 -3.441994
C 6.770219 -1.993746 -2.002271
H 5.536063 -2.031210 -0.245058
C 5.608111 -1.968365 -4.114287
H 3.458083 -1.955285 -4.006356
C 6.804062 -1.978305 -3.398859
H 7.695500 -2.005217 -1.433911
H 5.622179 -1.961341 -5.200475
H 7.755487 -1.977612 -3.922374
C 2.923300 -3.308898 -0.472856
C 3.007842 -3.392271 0.919505
C 2.778532 -4.497066 -1.211346
C 2.945645 -4.636189 1.558643
H 3.118490 -2.509580 1.538997
C 2.708168 -5.731406 -0.574014
H 2.728451 -4.447867 -2.294210
C 2.794127 -5.805616 0.819471
H 3.005661 -4.670890 2.641133
H 2.595574 -6.636372 -1.164351
H 2.745358 -6.768099 1.320479
C -4.149595 0.743935 -2.283552
C -4.473377 1.969061 -1.689314
C -4.195200 0.646800 -3.683433
C -4.814080 3.075252 -2.473188

H	-4.471537	2.084998	-0.610517
C	-4.527554	1.750723	-4.465027
H	-3.970061	-0.302049	-4.158551
C	-4.836764	2.972300	-3.862172
H	-5.055019	4.017318	-1.990148
H	-4.550014	1.655435	-5.546765
H	-5.094923	3.833877	-4.470429
C	-5.040901	-1.437546	-1.373382
C	-4.918635	-2.823441	-1.531773
C	-6.305091	-0.899332	-1.097904
C	-6.036060	-3.651484	-1.417648
H	-3.950696	-3.256000	-1.754606
C	-7.418751	-1.729114	-0.972893
H	-6.426508	0.173751	-0.993377
C	-7.289489	-3.109147	-1.134844
H	-5.923114	-4.723515	-1.551206
H	-8.389854	-1.292782	-0.758315
H	-8.158276	-3.754593	-1.045893
C	-0.498406	2.365496	2.896618
C	0.784614	1.866100	2.480040
C	-1.037371	0.954590	3.040410
H	-1.892479	0.664726	2.422986
H	-1.203240	0.639210	4.073061
N	0.287414	0.448176	2.500695
O	1.894314	2.257957	2.153210
C	0.979540	-0.605364	3.172444
C	0.287922	-1.779010	3.504023
C	2.330331	-0.471033	3.530876
C	0.931004	-2.792902	4.212285
H	-0.756504	-1.881803	3.227722
C	2.963959	-1.497969	4.232493
H	2.848839	0.452416	3.303559
C	2.271123	-2.659393	4.583286
H	0.376941	-3.687474	4.483379
H	4.000501	-1.371647	4.532735
H	2.764400	-3.441951	5.152635
C	-1.161287	3.687495	2.743768
H	-1.923128	3.835752	3.513047
H	-1.639371	3.779919	1.756039
H	-0.425620	4.488712	2.841221
H	-3.218808	0.815835	0.173283
C	-3.448162	-1.100755	1.070740
C	-4.413804	-0.638289	1.974389
C	-2.885141	-2.366071	1.274451
C	-4.813809	-1.422879	3.055764
H	-4.852856	0.346758	1.836132
C	-3.284849	-3.153764	2.355610
H	-2.118755	-2.733548	0.599708
C	-4.249983	-2.685120	3.248510
H	-5.557088	-1.044545	3.750963
H	-2.836340	-4.132110	2.502103
H	-4.554870	-3.295525	4.093173
H	2.838300	-0.843722	0.607585
C	3.446458	0.571408	-0.859773
C	4.237255	1.226068	0.089512
C	3.374735	1.094926	-2.158624
C	4.954363	2.374316	-0.250768
H	4.275415	0.852041	1.107464
C	4.092744	2.238465	-2.501841
H	2.762402	0.607207	-2.910570
C	4.887116	2.880560	-1.547835
H	5.551884	2.876912	0.503434
H	4.031323	2.630232	-3.513038
H	5.442577	3.774961	-1.814450

Br	0.225529	2.661804	5.551631
C	-0.432492	2.406554	-0.794796
H	-0.621940	2.095824	-1.833422
H	-1.399081	2.783045	-0.417150
C	0.610307	3.527587	-0.762774
H	1.567866	3.153415	-1.146568
H	0.811128	3.829416	0.271842
C	0.210774	4.773558	-1.575273
H	-0.744506	5.163610	-1.193633
H	0.021382	4.481520	-2.619514
C	1.262908	5.889934	-1.542557
H	1.447858	6.181322	-0.498910
H	2.218892	5.496753	-1.917738
C	0.873112	7.132959	-2.352376
H	-0.081570	7.525160	-1.975230
H	0.688564	6.842740	-3.396367
C	1.930220	8.241546	-2.310577
H	2.111192	8.576469	-1.282838
H	1.622456	9.115093	-2.895520
H	2.887424	7.891474	-2.714469

^{2}IM8-*S*

B3LYP/BSI SCF energy: -2935.517334a.u.

M06/BSII SCF energy in solution: -2934.180346 a.u.

M06/BSII free energy in solution: -2933.219554a.u.

C	1.109019	-2.528931	-0.752310
N	0.179612	-1.743178	-0.353244
O	0.835519	-3.854858	-0.773435
C	-0.628120	-3.976567	-0.542938
C	-0.940303	-2.582775	0.127731
C	2.847820	-0.751043	-0.746452
N	2.054256	0.246163	-0.593789
O	4.151817	-0.554679	-0.451685
C	4.244354	0.750880	0.240124
C	2.852634	1.410103	-0.151961
Ni	0.059060	0.245394	-0.887089
C	2.478325	-2.128627	-1.276198
C	3.531710	-3.176182	-0.852940
H	3.248189	-4.156929	-1.237685
H	3.621205	-3.238317	0.234847
H	4.509100	-2.906004	-1.256084
C	2.395935	-2.057864	-2.829235
H	3.375831	-1.798132	-3.241421
H	1.665079	-1.307710	-3.141319
H	2.097901	-3.031416	-3.227408
C	5.468462	1.490572	-0.272227
C	5.764166	2.759428	0.248414
C	6.314831	0.945166	-1.242219
C	6.874828	3.469994	-0.200176
H	5.123681	3.191245	1.011727
C	7.430933	1.657774	-1.689519
H	6.102043	-0.038051	-1.644782
C	7.713969	2.921010	-1.173324
H	7.085715	4.452934	0.210964
H	8.079048	1.218802	-2.442783
H	8.581029	3.474089	-1.522428
C	4.335387	0.407135	1.736285
C	3.182897	0.115617	2.485368
C	5.584852	0.269227	2.358550
C	3.281699	-0.291111	3.816907
H	2.196512	0.188399	2.040310
C	5.682120	-0.129105	3.692063
H	6.489459	0.476166	1.798071

C	4.530452	-0.409775	4.427813
H	2.375119	-0.522446	4.368618
H	6.661336	-0.221616	4.153186
H	4.605481	-0.720546	5.466006
C	-1.253889	-4.121784	-1.940535
C	-1.653643	-3.007908	-2.697146
C	-1.348835	-5.394216	-2.525599
C	-2.147242	-3.170412	-3.993767
H	-1.576259	-2.000977	-2.299641
C	-1.839877	-5.553678	-3.820476
H	-1.040785	-6.267581	-1.961050
C	-2.245240	-4.441048	-4.559579
H	-2.454203	-2.294032	-4.557118
H	-1.908960	-6.549533	-4.249082
H	-2.634014	-4.564002	-5.566450
C	-0.890638	-5.189243	0.332335
C	0.131056	-6.055087	0.734151
C	-2.207211	-5.460348	0.733005
C	-0.156916	-7.166232	1.531752
H	1.150532	-5.859482	0.423140
C	-2.492178	-6.565881	1.530778
H	-3.013159	-4.804263	0.416927
C	-1.466083	-7.424386	1.934297
H	0.647894	-7.829743	1.835613
H	-3.516363	-6.757133	1.837394
H	-1.688295	-8.287302	2.555277
C	-4.927721	2.564548	1.175687
N	-3.039716	2.864092	2.021804
C	-1.740772	3.052004	2.509086
C	-1.158786	2.105311	3.369515
C	-1.027087	4.202522	2.143551
C	0.129109	2.324397	3.851096

H	-1.715634	1.217261	3.641652
C	0.261042	4.405201	2.638465
H	-1.477415	4.934327	1.480494
C	0.847260	3.469605	3.492921
H	0.576144	1.590891	4.515489
H	0.801693	5.305515	2.359698
H	1.848158	3.631461	3.881318
C	-5.350541	1.771593	-0.038084
H	-5.959795	0.915314	0.260276
H	-4.453588	1.406574	-0.553409
H	-5.922546	2.389889	-0.734508
H	-1.873045	-2.180947	-0.268481
C	-1.012851	-2.514494	1.645726
C	-2.133172	-1.939187	2.253796
C	0.042047	-2.956413	2.458197
C	-2.211336	-1.815205	3.643629
H	-2.947966	-1.560944	1.643126
C	-0.035289	-2.842814	3.844946
H	0.924039	-3.401335	2.008399
C	-1.163666	-2.272129	4.442421
H	-3.083528	-1.343354	4.083629
H	0.784953	-3.202957	4.460068
H	-1.221765	-2.183061	5.523771
H	2.391001	1.825149	0.746184
C	2.853903	2.499140	-1.209965
C	2.481305	3.800623	-0.856361
C	3.197001	2.233721	-2.542254
C	2.463651	4.821638	-1.808789
H	2.186663	4.011647	0.168560
C	3.179292	3.250313	-3.495118
H	3.477118	1.227571	-2.837895
C	2.814650	4.549019	-3.130692

H	2.167932	5.825851	-1.518451
H	3.447724	3.028974	-4.524141
H	2.798346	5.339975	-3.874961
Br	-6.574258	3.307839	2.123950
C	-1.657377	1.011313	-1.554923
H	-2.503391	0.386098	-1.215596
H	-1.809756	1.990827	-1.065178
C	-1.767448	1.207102	-3.080029
H	-1.611069	0.245958	-3.595305
H	-0.950178	1.855087	-3.429816
C	-3.098578	1.802260	-3.575981
H	-3.262624	2.771236	-3.079900
H	-3.925692	1.152632	-3.250763
C	-3.177448	1.995516	-5.096322
H	-2.352583	2.646368	-5.421926
H	-3.009816	1.028415	-5.594015
C	-4.506797	2.586102	-5.583779
H	-4.674641	3.553587	-5.090364
H	-5.331585	1.936008	-5.260253
C	-4.571405	2.772292	-7.103434
H	-3.778264	3.442961	-7.454122
H	-5.530105	3.199049	-7.417701
H	-4.446714	1.816250	-7.625296
C	-3.811477	3.633591	1.029379
H	-3.341445	3.650091	0.040114
H	-4.075231	4.642940	1.353088
C	-3.991246	1.875605	2.189221
O	-4.026267	0.859042	2.846236

^{2}TS15-*R*

B3LYP/BSI SCF energy: -2935.507587a.u.

M06/BSII SCF energy in solution: -2934.176323a.u.

M06/BSII free energy in solution: -2933.216597a.u.

C	-1.602636	-0.507039	-1.773534
N	-1.648281	-0.225500	-0.522543
O	-2.780499	-0.592077	-2.423736
C	-3.818992	-0.041712	-1.533406
C	-3.077348	-0.148792	-0.136305
C	0.908784	-0.831522	-1.924606
N	1.219848	-0.332576	-0.785163
O	1.895385	-1.442132	-2.608475
C	3.008700	-1.649302	-1.657156
C	2.685099	-0.534350	-0.589716
Ni	-0.031951	0.690125	0.503915
C	-0.409134	-0.768417	-2.673986
C	-0.648948	-2.111446	-3.416055
H	-1.591352	-2.065962	-3.963047
H	-0.685415	-2.951339	-2.716300
H	0.162936	-2.292698	-4.120531
C	-0.305479	0.387089	-3.713508
H	0.519917	0.184284	-4.400339
H	-0.130163	1.345410	-3.219756
H	-1.232148	0.451327	-4.286750
C	4.329475	-1.498861	-2.400273
C	5.525358	-1.662959	-1.687554
C	4.387399	-1.242038	-3.773415
C	6.754271	-1.553666	-2.333403
H	5.496675	-1.884167	-0.624729
C	5.621386	-1.141242	-4.421177
H	3.469975	-1.120976	-4.337646
C	6.807309	-1.292878	-3.705004
H	7.671535	-1.676782	-1.765284
H	5.650473	-0.942846	-5.488821

H	7.765752	-1.212230	-4.209142
C	2.861801	-3.097112	-1.158442
C	2.962472	-3.478379	0.182321
C	2.669247	-4.095771	-2.129707
C	2.866201	-4.827127	0.543984
H	3.108857	-2.751701	0.973249
C	2.566351	-5.435127	-1.767935
H	2.607535	-3.816165	-3.176319
C	2.666204	-5.806939	-0.424083
H	2.936809	-5.093069	1.593153
H	2.417111	-6.189765	-2.535035
H	2.591029	-6.852096	-0.138128
C	-4.071066	1.401828	-1.993138
C	-4.330362	2.454048	-1.107910
C	-4.115024	1.661310	-3.372316
C	-4.606011	3.738402	-1.586794
H	-4.327555	2.295489	-0.034742
C	-4.382991	2.941885	-3.849669
H	-3.940898	0.850387	-4.071681
C	-4.627529	3.988303	-2.956880
H	-4.797103	4.541041	-0.881030
H	-4.406341	3.121980	-4.920534
H	-4.835566	4.987468	-3.327468
C	-5.075205	-0.894859	-1.687958
C	-5.013540	-2.193349	-2.207954
C	-6.318049	-0.387251	-1.285683
C	-6.170196	-2.965689	-2.323695
H	-4.062587	-2.599217	-2.531486
C	-7.470715	-1.164816	-1.390136
H	-6.391135	0.624758	-0.901119
C	-7.402084	-2.456969	-1.912685
H	-6.104680	-3.968190	-2.736663
H	-8.424320	-0.754030	-1.071757
H	-8.301017	-3.059585	-2.002628
C	-0.584534	1.494241	3.107352
C	0.749382	1.045877	2.772308
C	-1.092125	0.060729	3.112245
H	-1.914468	-0.206307	2.445774
H	-1.274528	-0.335152	4.114664
N	0.263724	-0.343000	2.586459
O	1.875804	1.479215	2.576092
C	0.934374	-1.506115	3.048577
C	0.221386	-2.708777	3.162600
C	2.289634	-1.463542	3.415888
C	0.850360	-3.848340	3.660573
H	-0.826312	-2.740736	2.880846
C	2.906749	-2.614347	3.907160
H	2.823398	-0.522636	3.363262
C	2.195110	-3.809812	4.037161
H	0.281905	-4.768639	3.763193
H	3.946867	-2.564619	4.217291
H	2.677592	-4.694504	4.442521
C	-1.236179	2.818785	3.271429
H	-1.932537	2.802175	4.113653
H	-1.788524	3.114001	2.367709
H	-0.483157	3.582401	3.476582
H	-3.221646	0.772366	0.427165
C	-3.493999	-1.316103	0.745152
C	-4.504339	-1.122697	1.696902
C	-2.913585	-2.584078	0.626415
C	-4.931351	-2.173187	2.508175
H	-4.957051	-0.140729	1.809698
C	-3.339267	-3.637344	1.438252
H	-2.111908	-2.748399	-0.085959

C	-4.349194	-3.435699	2.380197
H	-5.710203	-2.002802	3.245272
H	-2.875456	-4.614270	1.336917
H	-4.674836	-4.253579	3.015852
H	2.838632	-0.907504	0.421376
C	3.439484	0.777868	-0.732803
C	4.199618	1.252781	0.340433
C	3.391256	1.531438	-1.914690
C	4.912197	2.448596	0.233316
H	4.211378	0.703737	1.275862
C	4.104506	2.722982	-2.025212
H	2.803915	1.184518	-2.758851
C	4.870650	3.183637	-0.950662
H	5.486768	2.808037	1.081396
H	4.062681	3.292634	-2.949082
H	5.424751	4.113884	-1.035798
Br	0.194484	1.318023	5.886674
C	-0.357434	2.535594	-0.184541
H	-0.471239	2.439911	-1.274995
H	-1.338391	2.857997	0.197507
C	0.711299	3.581508	0.139382
H	1.686884	3.242591	-0.230711
H	0.830789	3.680071	1.224652
C	0.418730	4.971167	-0.458253
H	-0.551872	5.330019	-0.084915
H	0.307163	4.879790	-1.549347
C	1.501133	6.012391	-0.144760
H	1.606581	6.108459	0.945182
H	2.471143	5.644301	-0.508783
C	1.224248	7.394824	-0.749321
H	0.257135	7.764031	-0.380640
H	1.114650	7.298545	-1.838785
C	2.314858	8.424529	-0.435784
H	2.424045	8.568344	0.645192
H	2.088348	9.400025	-0.879681
H	3.288010	8.100958	-0.823305

^{2}TS15-*S*

B3LYP/BSI SCF energy: -2935.508392a.u.

M06/BSII SCF energy in solution: -2934.18253a.u.

M06/BSII free energy in solution: -2933.220028a.u.

C	-1.373855	-0.055530	-1.861816
N	-1.464059	-0.007027	-0.582492
O	-2.528943	-0.042821	-2.554832
C	-3.597451	0.306275	-1.587716
C	-2.906314	-0.132007	-0.242954
C	1.086774	-0.545695	-1.889035
N	1.354350	-0.194531	-0.681747
O	1.988240	-1.315917	-2.515235
C	2.943601	-1.806932	-1.490356
C	2.668266	-0.780992	-0.310376
Ni	0.068293	0.896067	0.560260
C	-0.127390	-0.173296	-2.727902
C	-0.385574	-1.275350	-3.790060
H	-1.249876	-1.001192	-4.395640
H	-0.574284	-2.246685	-3.325373
H	0.486027	-1.375344	-4.437292
C	0.158048	1.169485	-3.458095
H	0.984676	1.030349	-4.160614
H	0.418969	1.960128	-2.754545
H	-0.727423	1.476252	-4.019671
C	4.349701	-1.752876	-2.065580
C	5.411539	-2.257476	-1.300494

C	4.619041	-1.222521	-3.330617
C	6.716259	-2.218059	-1.786091
H	5.215090	-2.690771	-0.324228
C	5.927936	-1.190230	-3.819677
H	3.806362	-0.840572	-3.937275
C	6.979841	-1.683327	-3.049761
H	7.527095	-2.609468	-1.178909
H	6.120248	-0.779311	-4.806609
H	7.996559	-1.656530	-3.430327
C	2.489895	-3.244282	-1.194217
C	1.570062	-3.551038	-0.181253
C	2.920899	-4.279536	-2.041046
C	1.110119	-4.859431	-0.009577
H	1.191398	-2.791115	0.492376
C	2.459992	-5.582973	-1.870505
H	3.624457	-4.062818	-2.837260
C	1.553947	-5.878854	-0.849771
H	0.409871	-5.066475	0.792656
H	2.812047	-6.367480	-2.534174
H	1.199724	-6.896239	-0.710513
C	-3.821629	1.821010	-1.707154
C	-4.053444	2.647969	-0.602027
C	-3.867195	2.387060	-2.991545
C	-4.311874	4.011170	-0.776401
H	-4.038322	2.251086	0.407212
C	-4.116175	3.746389	-3.163692
H	-3.710906	1.752937	-3.858286
C	-4.339700	4.565601	-2.053998
H	-4.486259	4.635360	0.094872
H	-4.143971	4.165339	-4.165571
H	-4.537227	5.625169	-2.186626
C	-4.860393	-0.455870	-1.968975
C	-4.852536	-1.466841	-2.935907
C	-6.067085	-0.140267	-1.328739
C	-6.028259	-2.150020	-3.254168
H	-3.929080	-1.718593	-3.443079
C	-7.236725	-0.830722	-1.639671
H	-6.096836	0.656036	-0.591545
C	-7.222684	-1.838161	-2.606540
H	-6.005558	-2.928885	-4.011093
H	-8.161207	-0.575058	-1.130338
H	-8.135784	-2.371453	-2.854067
C	-0.175190	1.823621	2.965479
C	-0.987120	0.639220	2.780064
N	0.245327	-0.174166	2.565476
C	0.450634	-1.512197	2.979294
C	-0.609571	-2.431922	2.941854
C	1.717654	-1.928753	3.416482
C	-0.392470	-3.746310	3.351005
H	-1.591028	-2.101508	2.627725
C	1.920788	-3.250859	3.812245
H	2.530268	-1.212411	3.481578
C	0.869041	-4.167126	3.782449
H	-1.226007	-4.442764	3.339130
H	2.902691	-3.557454	4.161324
H	1.026596	-5.192419	4.103205
C	-0.477188	3.250437	3.233001
H	-1.559049	3.398988	3.272304
H	-0.058498	3.906437	2.460382
H	-0.063799	3.548863	4.199934
H	-3.129541	0.552291	0.570756
C	-3.238953	-1.540356	0.232622
C	-4.110277	-1.701176	1.317314
C	-2.722775	-2.683625	-0.389669

C	-4.469184	-2.974348	1.760461
H	-4.483503	-0.825906	1.838302
C	-3.077752	-3.958347	0.052982
H	-2.029177	-2.590459	-1.219125
C	-3.955658	-4.108027	1.127872
H	-5.138613	-3.077765	2.609206
H	-2.664023	-4.832760	-0.441344
H	-4.232103	-5.099795	1.474320
H	2.543778	-1.334691	0.621487
C	3.718046	0.289200	-0.069590
C	4.472760	0.255110	1.109427
C	3.963058	1.309986	-0.997284
C	5.453334	1.217546	1.359424
H	4.293723	-0.532971	1.836432
C	4.942667	2.270774	-0.751798
H	3.388003	1.356855	-1.916761
C	5.689720	2.228355	0.427891
H	6.025580	1.178007	2.281482
H	5.120936	3.056167	-1.480315
H	6.448800	2.980825	0.619657
Br	-0.550341	1.094591	5.908702
C	-0.128941	2.779745	-0.099243
H	-0.547238	2.706308	-1.110639
H	-0.906361	3.254547	0.510597
C	1.136370	3.640540	-0.114174
H	1.904251	3.174415	-0.745722
H	1.580204	3.691452	0.890895
C	0.905284	5.081037	-0.612396
H	0.149276	5.563770	0.022829
H	0.471775	5.046796	-1.622771
C	2.177822	5.937811	-0.631893
H	2.603564	5.977692	0.381090
H	2.937609	5.447226	-1.258459
C	1.953114	7.367632	-1.140829
H	1.194667	7.857821	-0.515507
H	1.530055	7.328605	-2.154255
C	3.229845	8.214618	-1.152531
H	3.654729	8.304138	-0.146233
H	3.037313	9.227116	-1.522685
H	3.997143	7.766656	-1.794901
C	1.094132	0.983770	3.027194
H	1.922544	1.227744	2.349333
H	1.462641	0.847930	4.048485
O	-2.155995	0.313855	2.687824

^{1}IM9 /B

B3LYP/BSI SCF energy: -2418.533127a.u.

M06/BSII SCF energy in solution: -2417.419289a.u.

M06/BSII free energy in solution: -2416.616414a.u.

C	-1.540831	-0.163308	-1.172728
N	-1.462674	0.415160	-0.032930
O	-2.761165	-0.372212	-1.693571
C	-3.710569	0.411843	-0.854450
C	-2.852139	0.616250	0.455029
C	0.781839	-0.979942	-1.128746
N	1.244242	-0.314197	-0.124766
O	1.392572	-2.139225	-1.427023
C	2.263383	-2.463094	-0.269951
C	2.482786	-1.018209	0.313518
Ni	0.307823	1.185520	0.764609
C	-0.368673	-0.583391	-2.038429
C	-0.768470	-1.748528	-2.966775
H	-1.597906	-1.437868	-3.603335

H	-1.074534	-2.630728	-2.399921
H	0.076620	-2.028614	-3.598301
C	0.054231	0.648961	-2.890383
H	0.865846	0.369900	-3.569340
H	0.384524	1.470846	-2.251414
H	-0.795848	0.986224	-3.489359
C	3.516656	-3.156046	-0.780075
C	4.359099	-3.801541	0.136565
C	3.865617	-3.153409	-2.134411
C	5.531101	-4.421702	-0.292009
H	4.090675	-3.828643	1.188512
C	5.034735	-3.785079	-2.563907
H	3.220226	-2.663112	-2.853236
C	5.872474	-4.417635	-1.646202
H	6.173291	-4.914776	0.431911
H	5.288182	-3.780155	-3.620122
H	6.781825	-4.907231	-1.982133
C	1.430253	-3.388812	0.628341
C	0.855034	-2.981712	1.838169
C	1.191556	-4.699872	0.180191
C	0.083371	-3.870402	2.593686
H	0.983077	-1.971457	2.209285
C	0.416183	-5.581464	0.927750
H	1.626292	-5.028926	-0.758173
C	-0.135605	-5.170682	2.144574
H	-0.352239	-3.531562	3.528090
H	0.248661	-6.591419	0.564749
H	-0.731234	-5.860730	2.735386
C	-3.963619	1.704812	-1.645238
C	-3.273897	2.898602	-1.387630
C	-4.856665	1.665605	-2.729499
C	-3.488212	4.025214	-2.187815
H	-2.566053	2.980646	-0.569031
C	-5.064715	2.788469	-3.526816
H	-5.395853	0.749059	-2.945338
C	-4.381905	3.976846	-3.255885
H	-2.949226	4.941187	-1.964058
H	-5.763908	2.736233	-4.356676
H	-4.547969	4.856065	-3.871996
C	-4.983611	-0.397645	-0.671469
C	-5.135797	-1.684207	-1.197024
C	-6.046106	0.174215	0.043650
C	-6.327012	-2.389664	-1.005083
H	-4.325070	-2.132647	-1.759171
C	-7.229298	-0.533377	0.241104
H	-5.947502	1.179554	0.442558
C	-7.374977	-1.819977	-0.284204
H	-6.431290	-3.387160	-1.422754
H	-8.039960	-0.077767	0.802218
H	-8.299434	-2.370029	-0.134284
C	1.924258	1.917749	1.531937
H	1.649749	2.260099	2.534831
H	2.663340	1.117118	1.647883
C	2.531126	3.070730	0.729534
H	1.781741	3.860969	0.603775
H	2.803770	2.731314	-0.279616
C	3.783363	3.673292	1.394366
H	4.531519	2.882029	1.545335
H	3.513544	4.037363	2.395882
C	4.415738	4.818073	0.591636
H	4.694906	4.449645	-0.406634
H	3.664577	5.603939	0.426214
C	5.649687	5.433731	1.263354
H	6.400041	4.648368	1.430483

H 5.371445 5.802200 2.260157

C 6.276764 6.574865 0.455225

H 6.596959 6.229870 -0.535111

H 7.153857 6.993869 0.960477

H 5.560544 7.390453 0.302032

H -2.937273 1.641736 0.806800

C -3.154080 -0.292868 1.634545

C -3.618326 0.275517 2.826893

C -2.981084 -1.681433 1.571936

C -3.922887 -0.526503 3.928145

H -3.722357 1.354285 2.896790

C -3.288809 -2.485374 2.668927

H -2.610320 -2.142678 0.661720

C -3.763594 -1.910229 3.850245

H -4.277770 -0.067666 4.846479

H -3.156155 -3.560986 2.599181

H -4.003157 -2.537032 4.704776

H 2.513048 -1.053096 1.400915

C 3.751464 -0.328096 -0.169182

C 4.892015 -0.367312 0.643612

C 3.836470 0.308045 -1.412112

C 6.091766 0.203649 0.221714

H 4.839111 -0.843587 1.619258

C 5.035466 0.883411 -1.836544

H 2.961809 0.376358 -2.049749

C 6.167607 0.831045 -1.023227

H 6.963261 0.165625 0.868684

H 5.080779 1.379153 -2.802007

H 7.098807 1.282637 -1.352265

Br -0.896492 3.041853 1.724289

^{2}**TS16**-*R*

B3LYP/BSI SCF energy: -2935.546199a.u.

M06/BSII SCF energy in solution: -2934.200745a.u.

M06/BSII free energy in solution: -2933.239189a.u.

C 0.380032 -2.015545 -0.764631

N 0.799578 -1.175578 0.107173

O 1.094623 -3.139642 -0.931612

C 2.146689 -3.190387 0.108615

C 1.960106 -1.772907 0.816786

C -1.887113 -0.947188 -1.201670

N -1.855824 -0.089285 -0.250322

O -3.028244 -1.021639 -1.916991

C -3.952453 0.016569 -1.452517

Ni -0.165857 0.520240 0.857624

C -0.842742 -1.950276 -1.665240

C -1.521199 -3.351447 -1.674981

H -0.805705 -4.101380 -2.010759

H -1.866055 -3.633840 -0.675674

H -2.373823 -3.340647 -2.354991

C -0.380222 -1.593873 -3.107642

H -1.250926 -1.510224 -3.761897

H 0.194849 -0.664850 -3.134480

H 0.256840 -2.398534 -3.483679

C -4.105785 1.030360 -2.592776

C -4.400070 2.378603 -2.350736

C -4.023789 0.591975 -3.922387

C -4.594415 3.268984 -3.409396

H -4.484389 2.749898 -1.334086

C -4.208086 1.482626 -4.979106

H -3.813591 -0.451654 -4.127414

C -4.494098 2.825737 -4.727604

H -4.820724 4.310067 -3.198173

H	-4.131544	1.123905	-6.001476
H	-4.639386	3.518826	-5.550860
C	-5.294051	-0.660772	-1.150588
C	-5.396116	-2.052804	-1.043608
C	-6.449008	0.112046	-0.967984
C	-6.622316	-2.656693	-0.758371
H	-4.518479	-2.670440	-1.188074
C	-7.670452	-0.490829	-0.673933
H	-6.401403	1.191197	-1.060773
C	-7.763884	-1.879610	-0.569605
H	-6.679962	-3.738952	-0.684472
H	-8.551657	0.128008	-0.532077
H	-8.717170	-2.349606	-0.346471
C	3.491407	-3.384267	-0.580464
C	4.642063	-3.564212	0.202784
C	3.618006	-3.382790	-1.972559
C	5.889088	-3.728585	-0.394396
H	4.560806	-3.570860	1.285065
C	4.869975	-3.550408	-2.571369
H	2.738738	-3.251212	-2.591409
C	6.008508	-3.722300	-1.786712
H	6.768790	-3.860720	0.228712
H	4.948742	-3.545844	-3.654817
H	6.981174	-3.851124	-2.252682
C	1.756751	-4.379762	1.002546
C	2.284531	-5.655863	0.751727
C	0.783337	-4.246958	2.006326
C	1.875934	-6.760749	1.498509
H	3.023602	-5.788526	-0.029864
C	0.376812	-5.354243	2.753211
H	0.325346	-3.287425	2.228613
C	0.923314	-6.613699	2.507177
H	2.305301	-7.736539	1.289439
H	-0.369101	-5.222394	3.531654
H	0.608752	-7.472330	3.093707
C	0.614303	2.442169	-1.603304
C	1.754053	1.796738	-2.286486
C	1.560997	3.546356	-1.135265
H	1.369192	4.538828	-1.566910
H	1.723116	3.633781	-0.057728
N	2.627619	2.804034	-1.848280
O	1.945547	0.821957	-3.002449
C	3.902543	3.202740	-2.235053
C	4.466698	4.365741	-1.683463
C	4.628219	2.456937	-3.185115
C	5.739670	4.773954	-2.076073
H	3.911373	4.938369	-0.947267
C	5.899553	2.881344	-3.562328
H	4.180467	1.566183	-3.609351
C	6.464681	4.037249	-3.014806
H	6.166559	5.673534	-1.641746
H	6.453411	2.302418	-4.296165
H	7.456608	4.358988	-3.316656
C	-0.757830	2.624163	-2.143817
H	-1.166049	1.690522	-2.535557
H	-1.443451	3.021409	-1.391003
H	-0.750238	3.345300	-2.978261
C	-0.589958	2.371750	1.493654
H	-1.545942	2.225917	2.011592
H	-0.759405	3.079125	0.671990
C	0.446961	2.933215	2.472747
H	0.561755	2.238403	3.312663
H	1.439919	2.996020	1.999590
C	0.089287	4.326345	3.026497

H	-0.016409	5.036647	2.192122
H	-0.897712	4.273964	3.507551
C	1.115568	4.869123	4.029622
H	2.105942	4.909608	3.552035
H	1.212748	4.160304	4.864055
C	0.767780	6.256802	4.583946
H	0.673484	6.966874	3.750351
H	-0.221403	6.217509	5.060388
C	1.797145	6.783832	5.589265
H	2.790533	6.866355	5.132833
H	1.521760	7.774482	5.966980
H	1.887067	6.112485	6.450971
Br	-0.430856	-0.607989	3.113526
C	3.163510	-0.858616	0.915873
C	3.751580	-0.244460	-0.197906
C	3.724444	-0.648189	2.183470
C	4.893710	0.541752	-0.043519
H	3.324804	-0.380938	-1.186606
C	4.865093	0.141157	2.337662
H	3.257435	-1.099806	3.054492
C	5.455561	0.732782	1.221185
H	5.346171	1.005300	-0.913841
H	5.285079	0.294669	3.327653
H	6.345371	1.346032	1.333443
H	1.620200	-1.933833	1.839365
C	-3.167413	0.598913	-0.186238
H	-2.974598	1.659534	-0.349889
C	-3.856576	0.461351	1.157827
C	-4.496021	1.579456	1.706765
C	-3.903125	-0.752027	1.851593
C	-5.184094	1.486864	2.916777
H	-4.447062	2.535974	1.191127
C	-4.588149	-0.847285	3.061589
H	-3.383129	-1.620126	1.462254
C	-5.233965	0.269018	3.595629
H	-5.670074	2.365530	3.330998
H	-4.605206	-1.792955	3.594656
H	-5.762286	0.192646	4.541531

[2]TS16-*S*

B3LYP/BSI SCF energy: -2935.542778a.u.

M06/BSII SCF energy in solution: -2934.209605a.u.

M06/BSII free energy in solution: -2933.246007a.u.

C	2.488427	-0.962908	-0.492347
N	1.942876	0.092379	-0.006992
O	3.838057	-0.999690	-0.541247
C	4.306536	0.104135	0.334316
C	3.062446	1.051711	0.267770
C	0.369888	-2.325453	-0.773687
N	-0.432088	-1.553278	-0.125054
O	-0.140619	-3.477076	-1.250687
C	-1.612160	-3.378676	-1.127186
Ni	-0.088917	0.330193	0.673556
C	1.847219	-2.199327	-1.078595
C	2.558247	-3.458632	-0.503910
H	3.621238	-3.417013	-0.741330
H	2.456055	-3.507792	0.583993
H	2.125602	-4.359219	-0.941021
C	2.040771	-2.159040	-2.622627
H	1.600228	-3.051085	-3.071105
H	1.560466	-1.278868	-3.056444
H	3.107211	-2.132129	-2.857436
C	-2.115106	-2.891270	-2.493745

C	-2.761817	-1.668532	-2.700047
C	-1.880915	-3.724162	-3.603060
C	-3.169072	-1.286905	-3.983882
H	-2.960539	-0.969027	-1.897300
C	-2.282968	-3.342871	-4.879544
H	-1.384769	-4.678949	-3.456985
C	-2.933612	-2.119694	-5.074319
H	-3.658594	-0.325658	-4.101989
H	-2.094439	-4.001987	-5.722345
H	-3.251923	-1.823259	-6.069812
C	-2.165598	-4.755968	-0.791156
C	-1.351963	-5.776998	-0.288331
C	-3.534994	-5.002183	-0.963022
C	-1.897547	-7.020573	0.035784
H	-0.291631	-5.600483	-0.153126
C	-4.080255	-6.240383	-0.627645
H	-4.174720	-4.225294	-1.370438
C	-3.262254	-7.256036	-0.128822
H	-1.251242	-7.805062	0.418965
H	-5.143719	-6.413160	-0.764889
H	-3.685218	-8.223619	0.125100
C	5.598737	0.672273	-0.226724
C	6.203774	1.748618	0.437969
C	6.218063	0.142604	-1.362747
C	7.393755	2.294798	-0.037087
H	5.744384	2.156864	1.333650
C	7.416165	0.686888	-1.833674
H	5.765369	-0.696743	-1.877775
C	8.005489	1.765377	-1.176506
H	7.845858	3.132553	0.485694
H	7.886334	0.262909	-2.716520
H	8.935359	2.189240	-1.544191

C	4.533335	-0.537886	1.714018
C	5.654701	-1.367288	1.884676
C	3.637144	-0.392206	2.782221
C	5.877802	-2.029975	3.088839
H	6.360033	-1.487201	1.068581
C	3.867355	-1.054675	3.992665
H	2.754064	0.232799	2.700876
C	4.983604	-1.873241	4.151326
H	6.753585	-2.663086	3.200135
H	3.164368	-0.919869	4.809483
H	5.160321	-2.382497	5.094520
C	-0.963047	1.922718	-1.800637
C	-2.424212	2.097511	-1.715061
N	-2.267660	3.410873	-1.252615
C	-3.144384	4.449604	-0.963868
C	-4.533170	4.274146	-1.122711
C	-2.643412	5.685459	-0.519834
C	-5.393626	5.329238	-0.832603
H	-4.905849	3.317988	-1.469704
C	-3.522172	6.728649	-0.235306
H	-1.573777	5.819542	-0.392811
C	-4.899816	6.560047	-0.388685
H	-6.463682	5.187834	-0.957312
H	-3.124644	7.678367	0.111511
H	-5.580313	7.376023	-0.165316
C	-0.181873	1.156759	-2.803381
H	0.861142	1.045979	-2.502504
H	-0.624920	0.170847	-2.965857
H	-0.181531	1.679666	-3.775131
C	-1.969990	0.491438	1.199894
H	-2.060059	-0.367263	1.876270
H	-2.505319	0.268919	0.269426

C	-2.598019	1.719533	1.851766
H	-2.194875	1.857501	2.859425
H	-2.342717	2.631105	1.300572
C	-4.134137	1.606970	1.926901
H	-4.537625	1.494159	0.910892
H	-4.407495	0.690143	2.470886
C	-4.794705	2.815121	2.603895
H	-4.503736	3.729463	2.069168
H	-4.402187	2.920884	3.625662
C	-6.325535	2.732254	2.655453
H	-6.712891	2.610464	1.634472
H	-6.624159	1.827158	3.202947
C	-6.975004	3.960259	3.302214
H	-6.723555	4.872741	2.749411
H	-8.067093	3.874841	3.327328
H	-6.628190	4.094806	4.333447
Br	0.596309	2.057453	2.269616
C	3.108723	2.159017	-0.775833
C	3.331882	1.903783	-2.137057
C	2.934702	3.486001	-0.363385
C	3.383675	2.947983	-3.059868
H	3.483842	0.885562	-2.480728
C	2.985283	4.533668	-1.286003
H	2.738675	3.694887	0.683585
C	3.210240	4.268096	-2.636922
H	3.564589	2.731150	-4.108909
H	2.848054	5.556120	-0.945586
H	3.252875	5.081538	-3.355664
H	2.872536	1.517736	1.230508
C	-1.706863	-2.316734	0.025193
H	-2.546769	-1.651981	-0.149855
C	-1.855762	-2.890994	1.428837
C	-3.144249	-3.044032	1.958602
C	-0.760688	-3.285477	2.205074
C	-3.335318	-3.588743	3.227135
H	-4.004711	-2.726681	1.375796
C	-0.948956	-3.826740	3.478374
H	0.248845	-3.152584	1.831598
C	-2.235990	-3.982552	3.992751
H	-4.341809	-3.694933	3.621159
H	-0.085954	-4.119619	4.069464
H	-2.381779	-4.398591	4.985314
C	-0.786299	3.356614	-1.306449
H	-0.303426	3.472599	-0.331563
H	-0.351847	4.057222	-2.032624
O	-3.431338	1.439421	-1.953508

^{2}IM10-*R*

B3LYP/BSI SCF energy: -2935.570518a.u.

M06/BSII SCF energy in solution: -2934.228284a.u.

M06/BSII free energy in solution: -2933.260689a.u.

C	0.683774	-2.001220	-0.412025
N	1.030417	-0.924511	0.187219
O	1.518404	-3.053271	-0.323017
C	2.616116	-2.720238	0.603081
C	2.246985	-1.218819	0.998390
C	-1.713212	-1.357560	-0.999890
N	-1.787391	-0.285898	-0.303245
O	-2.836071	-1.747692	-1.643242
C	-3.856413	-0.713035	-1.481846
Ni	-0.112598	0.815424	0.507833
C	-0.568809	-2.333848	-1.207754
C	-1.079802	-3.730233	-0.738921

H	-0.300896	-4.475990	-0.894081
H	-1.338035	-3.722677	0.324335
H	-1.961750	-4.008587	-1.317178
C	-0.206344	-2.400781	-2.717892
H	-1.094554	-2.657504	-3.298504
H	0.208021	-1.450972	-3.059482
H	0.542460	-3.182527	-2.868350
C	-4.023860	-0.018954	-2.840707
C	-4.444100	1.313464	-2.946723
C	-3.821808	-0.750630	-4.019600
C	-4.644532	1.903661	-4.196589
H	-4.623069	1.907402	-2.055741
C	-4.012593	-0.159185	-5.267659
H	-3.511976	-1.787456	-3.956465
C	-4.424348	1.171271	-5.362328
H	-4.968001	2.938911	-4.253748
H	-3.840823	-0.741886	-6.168083
H	-4.572640	1.631609	-6.334603
C	-5.155841	-1.408694	-1.062801
C	-5.145189	-2.718477	-0.567581
C	-6.383422	-0.739611	-1.159140
C	-6.331777	-3.343096	-0.179939
H	-4.208704	-3.256746	-0.490927
C	-7.566987	-1.360549	-0.763037
H	-6.423182	0.269011	-1.554773
C	-7.547484	-2.667183	-0.273479
H	-6.300998	-4.361931	0.195715
H	-8.506617	-0.821877	-0.844759
H	-8.470483	-3.153331	0.028638
C	3.936839	-2.881306	-0.141508
C	5.145260	-2.733952	0.557734
C	3.982786	-3.166848	-1.509285
C	6.366784	-2.865487	-0.097678
H	5.128423	-2.509434	1.619496
C	5.209285	-3.298053	-2.167026
H	3.059576	-3.285076	-2.062835
C	6.404083	-3.148357	-1.465661
H	7.290544	-2.743804	0.460471
H	5.224128	-3.519341	-3.230474
H	7.356825	-3.250513	-1.977245
C	2.462683	-3.714863	1.768894
C	3.218874	-4.896114	1.808972
C	1.478339	-3.523652	2.752813
C	3.023400	-5.840581	2.817349
H	3.970044	-5.082212	1.050693
C	1.284733	-4.469010	3.761029
H	0.844602	-2.641653	2.753053
C	2.058949	-5.628726	3.802185
H	3.628056	-6.743093	2.828851
H	0.523763	-4.290440	4.515341
H	1.909457	-6.360908	4.590720
C	0.357278	1.851899	-1.208751
C	1.462659	1.264417	-2.092644
C	1.302688	3.095054	-1.087647
H	0.898600	4.035966	-1.480903
H	1.754102	3.267759	-0.107906
N	2.219561	2.420635	-2.018668
O	1.646403	0.240453	-2.733873
C	3.362502	2.875768	-2.679096
C	3.883470	4.142536	-2.371889
C	3.991653	2.080218	-3.654679
C	5.022550	4.604692	-3.029248
H	3.400648	4.755772	-1.617598
C	5.130671	2.558378	-4.298524

H	3.577227	1.107127	-3.888497
C	5.654862	3.817987	-3.993659
H	5.417498	5.586138	-2.781861
H	5.611712	1.938416	-5.050170
H	6.542539	4.181233	-4.502452
C	-0.849674	2.128662	-2.114728
H	-1.280181	1.200720	-2.495469
H	-1.638388	2.690758	-1.608190
H	-0.546821	2.721528	-2.990770
C	-0.971246	2.532664	1.111995
H	-1.859926	2.112860	1.588850
H	-1.262458	3.132202	0.248482
C	-0.151808	3.356455	2.097124
H	0.108378	2.745637	2.965590
H	0.793438	3.686666	1.647232
C	-0.917677	4.609710	2.572824
H	-1.197003	5.224709	1.704570
H	-1.859217	4.296340	3.044027
C	-0.112235	5.462815	3.561707
H	0.831910	5.772255	3.089855
H	0.169200	4.844671	4.425684
C	-0.861712	6.708019	4.053075
H	-1.147035	7.323973	3.188862
H	-1.802625	6.398741	4.528284
C	-0.047386	7.554973	5.036558
H	0.884471	7.907048	4.578953
H	-0.607762	8.435422	5.368654
H	0.222278	6.976688	5.927699
Br	-0.323375	-0.018786	2.924749
C	3.341086	-0.170877	0.946911
C	3.975058	0.235580	-0.235474
C	3.764253	0.384057	2.164469
C	5.018036	1.161337	-0.194741
H	3.658375	-0.175213	-1.187588
C	4.806950	1.311250	2.205715
H	3.266374	0.090149	3.084421
C	5.439399	1.698281	1.024003
H	5.498830	1.464328	-1.119149
H	5.119951	1.728682	3.158581
H	6.254019	2.416835	1.049449
H	1.893216	-1.216084	2.028070
C	-3.178241	0.224336	-0.378994
H	-3.126969	1.239501	-0.770737
C	-3.878911	0.285084	0.965608
C	-4.728076	1.366151	1.235505
C	-3.738410	-0.719296	1.928356
C	-5.435066	1.438417	2.435888
H	-4.834461	2.163746	0.503204
C	-4.442147	-0.648815	3.130055
H	-3.053809	-1.542050	1.756719
C	-5.294550	0.426152	3.386352
H	-6.086584	2.285711	2.629685
H	-4.312974	-1.430294	3.872725
H	-5.837925	0.479817	4.325315

[2]IM10-*S*

B3LYP/BSI SCF energy: -2935.578078a.u.

M06/BSII SCF energy in solution: -2934.23585a.u.

M06/BSII free energy in solution: -2933.268863a.u.

C	2.049771	-1.678395	0.098104
N	1.823857	-0.419228	0.033678
O	3.331835	-2.074026	0.215285
C	4.204352	-0.885354	0.254570

C	3.113409	0.283958	0.277649
C	-0.371686	-2.484187	-0.033920
N	-0.960955	-1.369485	0.183837
O	-1.149968	-3.523753	-0.397475
C	-2.494392	-2.994507	-0.701344
Ni	-0.076677	0.519385	0.475386
C	1.089100	-2.856521	0.143733
C	1.221729	-3.498048	1.560794
H	2.244735	-3.848464	1.710376
H	0.980662	-2.764997	2.335233
H	0.541949	-4.350451	1.639124
C	1.503766	-3.886143	-0.938792
H	0.877083	-4.775060	-0.865160
H	1.391879	-3.472181	-1.945383
H	2.544617	-4.174986	-0.787482
C	-2.605954	-2.962089	-2.234008
C	-3.152018	-1.890697	-2.950500
C	-2.189567	-4.103150	-2.941333
C	-3.270959	-1.960759	-4.343668
H	-3.465872	-0.977025	-2.458257
C	-2.302384	-4.166381	-4.327114
H	-1.777322	-4.945681	-2.395706
C	-2.847759	-3.092046	-5.036120
H	-3.690903	-1.115011	-4.880186
H	-1.970904	-5.056843	-4.853889
H	-2.939903	-3.139997	-6.117393
C	-3.523701	-3.957481	-0.115052
C	-3.175401	-4.898470	0.860109
C	-4.855133	-3.896236	-0.548899
C	-4.137535	-5.760610	1.389421
H	-2.148850	-4.964602	1.200105
C	-5.817100	-4.750046	-0.011939
H	-5.139651	-3.185425	-1.318191
C	-5.461661	-5.688491	0.958611
H	-3.847232	-6.489267	2.141037
H	-6.844209	-4.685829	-0.359358
H	-6.209568	-6.359045	1.371705
C	5.084650	-0.903264	-0.993309
C	6.074977	0.079006	-1.158085
C	4.935692	-1.874131	-1.989152
C	6.885771	0.092088	-2.290355
H	6.210464	0.839292	-0.395687
C	5.747371	-1.859052	-3.127590
H	4.190666	-2.651701	-1.874702
C	6.723790	-0.877379	-3.283463
H	7.643831	0.862469	-2.397283
H	5.615158	-2.623277	-3.888272
H	7.355684	-0.867185	-4.166778
C	5.010809	-1.011122	1.558825
C	6.339407	-1.462111	1.553090
C	4.391784	-0.783912	2.798860
C	7.039943	-1.644102	2.746499
H	6.837292	-1.674485	0.614865
C	5.093800	-0.966064	3.990432
H	3.350453	-0.481515	2.859038
C	6.422821	-1.390156	3.970794
H	8.069935	-1.987887	2.713704
H	4.591441	-0.777181	4.934712
H	6.968926	-1.528407	4.899658
C	-0.457889	1.225827	-1.416717
C	-1.842982	1.761921	-1.715670
N	-1.387207	3.061721	-1.861720
C	-1.996141	4.283305	-2.162023
C	-3.386862	4.359320	-2.359827

C	-1.212292	5.443134	-2.266273
C	-3.970106	5.588861	-2.654559
H	-3.979599	3.455928	-2.281727
C	-1.815373	6.665119	-2.561720
H	-0.138649	5.384143	-2.116043
C	-3.194749	6.747485	-2.757534
H	-5.044907	5.640854	-2.805725
H	-1.199639	7.557051	-2.638633
H	-3.660283	7.701103	-2.987229
C	-0.011509	0.208499	-2.468687
H	1.010551	-0.133080	-2.309221
H	-0.667428	-0.664357	-2.467787
H	-0.066881	0.648243	-3.476199
C	-1.627678	1.595750	1.196931
H	-1.823086	0.975107	2.070796
H	-2.462121	1.536224	0.502209
C	-1.275954	3.020138	1.586075
H	-0.413103	3.021749	2.261223
H	-1.010441	3.617566	0.706430
C	-2.460668	3.719088	2.290525
H	-3.336586	3.702599	1.627509
H	-2.736038	3.144752	3.185070
C	-2.149600	5.168376	2.686866
H	-1.880024	5.740046	1.787187
H	-1.263494	5.184593	3.337498
C	-3.313187	5.868840	3.400523
H	-4.200163	5.846510	2.752732
H	-3.580221	5.298560	4.300700
C	-3.000760	7.317935	3.788144
H	-2.765868	7.922044	2.904132
H	-3.848082	7.789632	4.297323
H	-2.137374	7.370322	4.461552
Br	0.730028	0.321558	2.919576
C	3.342383	1.501521	-0.596251
C	3.327066	1.458549	-1.998064
C	3.594275	2.729325	0.032235
C	3.561399	2.610331	-2.747991
H	3.150116	0.518793	-2.508463
C	3.830917	3.884879	-0.716420
H	3.601773	2.780096	1.117944
C	3.815452	3.827526	-2.110173
H	3.551320	2.555606	-3.832849
H	4.024510	4.825402	-0.208827
H	4.000606	4.722956	-2.696669
H	3.037423	0.652771	1.301889
C	-2.418029	-1.565955	-0.011780
H	-2.761286	-0.793974	-0.700161
C	-3.199863	-1.410887	1.282057
C	-4.478903	-0.842400	1.230958
C	-2.690735	-1.823143	2.518221
C	-5.242260	-0.703277	2.389996
H	-4.872249	-0.492962	0.279891
C	-3.450494	-1.678348	3.679796
H	-1.685333	-2.224132	2.584946
C	-4.729360	-1.122612	3.618806
H	-6.231264	-0.257365	2.334088
H	-3.036310	-1.991661	4.633621
H	-5.318296	-1.007464	4.524277
C	0.024212	2.696789	-1.646076
H	0.485116	3.208210	-0.795707
H	0.645369	2.843440	-2.535947
O	-2.968158	1.291127	-1.842999

^{2}**TS17**-*R*

B3LYP/BSI SCF energy: -2935.54974a.u.

M06/BSII SCF energy in solution: -2934.220763a.u.

M06/BSII free energy in solution: -2933.254866a.u.

C	1.023220	-1.889858	-0.686176
N	1.283452	-0.813721	-0.034474
O	1.863565	-2.933673	-0.508582
C	2.656735	-2.612480	0.699901
C	2.584670	-1.049386	0.663061
C	-1.358929	-1.431024	-1.349522
N	-1.538838	-0.396838	-0.613007
O	-2.455034	-1.979843	-1.920483
C	-3.578487	-1.067171	-1.702952
Ni	0.026637	0.852769	0.365845
C	-0.079689	-2.167600	-1.694371
C	-0.372316	-3.690157	-1.740218
H	0.528819	-4.224684	-2.040049
H	-0.678625	-4.072277	-0.762360
H	-1.163097	-3.894271	-2.461862
C	0.398820	-1.691460	-3.098538
H	-0.380981	-1.897006	-3.836853
H	0.624734	-0.624174	-3.092292
H	1.293659	-2.254093	-3.380924
C	-3.767362	-0.267211	-3.000290
C	-4.288701	1.033287	-3.008517
C	-3.477706	-0.876684	-4.229665
C	-4.498476	1.713241	-4.211236
H	-4.547421	1.532092	-2.079492
C	-3.679194	-0.196179	-5.429412
H	-3.090448	-1.889163	-4.241693
C	-4.189357	1.103299	-5.425771
H	-4.900511	2.721969	-4.192450
H	-3.439314	-0.684749	-6.369339
H	-4.345511	1.633852	-6.360240
C	-4.807946	-1.913730	-1.372757
C	-4.663315	-3.184296	-0.799314
C	-6.100265	-1.427812	-1.610066
C	-5.783333	-3.947561	-0.470853
H	-3.671924	-3.581552	-0.618371
C	-7.220150	-2.188438	-1.272262
H	-6.239720	-0.457247	-2.073180
C	-7.066994	-3.452320	-0.702742
H	-5.649226	-4.932538	-0.033021
H	-8.213182	-1.792808	-1.465187
H	-7.938660	-4.047432	-0.446596
C	4.043396	-3.216981	0.560678
C	4.912477	-3.171879	1.660284
C	4.480280	-3.817298	-0.624108
C	6.195973	-3.706078	1.570725
H	4.579397	-2.724169	2.592162
C	5.763975	-4.361946	-0.708826
H	3.815067	-3.860902	-1.478303
C	6.626626	-4.305831	0.384761
H	6.858259	-3.658344	2.430296
H	6.087155	-4.828984	-1.634978
H	7.625349	-4.727397	0.316804
C	1.887237	-3.251404	1.869682
C	1.795020	-4.653926	1.900808
C	1.238282	-2.513693	2.867754
C	1.076309	-5.302519	2.900954
H	2.297656	-5.237035	1.135127
C	0.523625	-3.169381	3.877944
H	1.271030	-1.429050	2.885799
C	0.438160	-4.559588	3.898994

H	1.020977	-6.387566	2.907355
H	0.039298	-2.577123	4.649137
H	-0.114184	-5.063896	4.687091
C	-0.103882	2.707088	-0.669522
C	1.020500	2.213219	-1.630953
C	0.949845	3.754283	-0.200486
H	0.688010	4.796257	-0.420388
H	1.296319	3.643190	0.827464
N	1.886223	3.190147	-1.186948
O	1.101182	1.424855	-2.557711
C	3.074113	3.723411	-1.702167
C	3.703939	4.777496	-1.022860
C	3.619435	3.240154	-2.903742
C	4.866711	5.343028	-1.543756
H	3.288181	5.143378	-0.089403
C	4.781073	3.818448	-3.408968
H	3.126975	2.423291	-3.415880
C	5.411683	4.870086	-2.738594
H	5.347524	6.157272	-1.008861
H	5.196151	3.443376	-4.340651
H	6.315908	5.315237	-3.142884
C	-1.214798	3.269660	-1.563385
H	-1.759800	2.457918	-2.053344
H	-1.927158	3.893530	-1.016727
H	-0.779133	3.888246	-2.358926
C	-1.371240	2.274701	0.938517
H	-1.389065	1.424225	1.643307
H	-2.267769	2.232379	0.326965
C	-1.307042	3.563649	1.741019
H	-0.374505	3.593140	2.309735
H	-1.327758	4.440168	1.080278
C	-2.487769	3.669660	2.726179
H	-3.438994	3.596001	2.178759
H	-2.460257	2.813192	3.412220
C	-2.466833	4.971926	3.537357
H	-2.491641	5.830956	2.850803
H	-1.513636	5.042562	4.079293
C	-3.626047	5.090349	4.535095
H	-4.579660	5.015433	3.993969
H	-3.598767	4.233579	5.221798
C	-3.597906	6.392440	5.342128
H	-3.659137	7.267914	4.685229
H	-4.434816	6.446511	6.046415
H	-2.670758	6.479017	5.919961
Br	1.358790	1.423940	2.619901
C	3.743883	-0.356170	-0.034105
C	3.974689	-0.465787	-1.411489
C	4.646720	0.378896	0.744119
C	5.098803	0.121465	-1.990358
H	3.276250	-1.007470	-2.040330
C	5.770772	0.969647	0.165092
H	4.455112	0.501411	1.805886
C	6.004065	0.835850	-1.203233
H	5.265210	0.025150	-3.059592
H	6.457822	1.539989	0.783613
H	6.874346	1.300016	-1.657838
H	2.513825	-0.632940	1.663457
C	-3.002681	-0.182788	-0.517701
H	-3.215085	0.862167	-0.734075
C	-3.528022	-0.490075	0.876780
C	-4.685265	0.159001	1.327272
C	-2.894372	-1.402943	1.727451
C	-5.205672	-0.105562	2.593206
H	-5.184593	0.879109	0.683213

C -3.410489 -1.665189 2.997982

H -1.980158 -1.894504 1.413249

C -4.568088 -1.020489 3.433868

H -6.103041 0.408229 2.925614

H -2.896958 -2.369164 3.646108

H -4.966814 -1.221807 4.423846

[2]TS17-*S*

B3LYP/BSI SCF energy: -2935.550673a.u.

M06/BSII SCF energy in solution: -2934.225606a.u.

M06/BSII free energy in solution: -2933.261196a.u.

C -1.183963 -2.260817 0.519003

N -1.397936 -1.095234 0.028443

O -2.214856 -3.135051 0.512640

C -3.237429 -2.576494 -0.402266

C -2.827520 -1.054848 -0.392606

C 1.317502 -2.025845 0.930368

N 1.475287 -0.885114 0.369882

O 2.428219 -2.641447 1.397612

C 3.522204 -1.658674 1.356829

Ni -0.033689 0.512084 -0.378956

C 0.065394 -2.851010 1.146919

C 0.314089 -4.256028 0.525216

H -0.548620 -4.896346 0.708267

H 0.461122 -4.185237 -0.556470

H 1.200823 -4.706176 0.973236

C -0.182076 -3.000245 2.675574

H 0.675532 -3.485894 3.144415

H -0.330549 -2.026386 3.149763

H -1.069668 -3.614143 2.843091

C 3.644135 -1.078056 2.774789

C 3.956463 0.263343 3.028428

C 3.495934 -1.946596 3.868029

C 4.094358 0.730225 4.339433

H 4.100360 0.968618 2.216781

C 3.629851 -1.481499 5.174284

H 3.275272 -2.993112 3.687597

C 3.927068 -0.137982 5.415921

H 4.325908 1.777250 4.509368

H 3.505571 -2.170465 6.004872

H 4.029315 0.226387 6.433775

C 4.792283 -2.392964 0.936808

C 4.723670 -3.547588 0.146002

C 6.051742 -1.906114 1.310193

C 5.887789 -4.198955 -0.260646

H 3.757838 -3.941277 -0.147160

C 7.215874 -2.553036 0.894026

H 6.127745 -1.024680 1.938045

C 7.138851 -3.703405 0.109002

H 5.814320 -5.095817 -0.868978

H 8.182940 -2.159764 1.193819

H 8.044728 -4.211220 -0.208890

C -4.618107 -2.879391 0.158204

C -5.744854 -2.521695 -0.596489

C -4.802717 -3.508241 1.393062

C -7.027975 -2.777288 -0.118675

H -5.615429 -2.049178 -1.565819

C -6.090630 -3.772025 1.867499

H -3.939427 -3.797046 1.980996

C -7.206233 -3.405672 1.116586

H -7.889617 -2.490194 -0.714312

H -6.217159 -4.266402 2.826611

H -8.206742 -3.610127 1.486436

C	-3.005907	-3.287048	-1.745954
C	-3.430821	-4.620997	-1.872609
C	-2.317112	-2.697512	-2.814921
C	-3.184746	-5.343184	-3.037338
H	-3.963108	-5.092977	-1.052950
C	-2.077013	-3.424595	-3.985916
H	-1.957713	-1.674012	-2.769133
C	-2.508149	-4.744120	-4.103363
H	-3.526368	-6.371591	-3.114875
H	-1.551601	-2.943013	-4.805489
H	-2.322723	-5.302768	-5.016535
C	-0.120831	2.077266	1.030931
C	0.322128	3.495999	1.446567
N	-0.936493	4.015873	1.228507
C	-1.504428	5.282025	1.395391
C	-0.730455	6.354372	1.873703
C	-2.857999	5.480068	1.082505
C	-1.320057	7.606352	2.029405
H	0.312551	6.185902	2.113919
C	-3.429298	6.741346	1.245078
H	-3.454091	4.649841	0.716731
C	-2.667316	7.810836	1.718133
H	-0.717449	8.431882	2.398288
H	-4.477397	6.886182	0.997910
H	-3.116406	8.791611	1.842265
C	-0.010705	1.195371	2.287918
H	-0.551678	0.253616	2.195217
H	1.037029	0.982035	2.513055
H	-0.422106	1.736305	3.152951
C	1.299339	2.097194	-0.546585
H	1.588815	1.287844	-1.240291
H	2.088035	2.220196	0.195023
C	1.058599	3.366628	-1.355288
H	0.074974	3.328709	-1.831989
H	1.090860	4.248224	-0.708820
C	2.123078	3.529609	-2.456850
H	3.126669	3.504438	-2.007416
H	2.062935	2.672592	-3.140150
C	1.959101	4.828436	-3.257416
H	2.007938	5.688435	-2.573853
H	0.956013	4.850329	-3.705569
C	3.010384	4.999540	-4.360962
H	4.013553	4.971946	-3.912883
H	2.959105	4.140585	-5.043743
C	2.844214	6.296805	-5.159370
H	2.925870	7.175604	-4.509119
H	3.606531	6.389515	-5.940540
H	1.862955	6.338254	-5.645887
Br	-1.187296	0.978567	-2.671333
C	-3.646525	-0.139583	0.501958
C	-3.628827	-0.243430	1.899514
C	-4.456284	0.837688	-0.089628
C	-4.405922	0.606174	2.686765
H	-3.007586	-0.993770	2.378988
C	-5.239137	1.686978	0.695530
H	-4.458292	0.943699	-1.170685
C	-5.215430	1.574173	2.086465
H	-4.381901	0.511527	3.768720
H	-5.862421	2.438317	0.218925
H	-5.820265	2.235802	2.699778
H	-2.856681	-0.638620	-1.397804
C	2.933927	-0.626476	0.310492
H	3.114541	0.385589	0.665418
C	3.471646	-0.718022	-1.110813

C	4.655093	-0.037116	-1.428777
C	2.827807	-1.454224	-2.111428
C	5.193267	-0.104336	-2.712271
H	5.160451	0.549287	-0.665606
C	3.362362	-1.516575	-3.400215
H	1.888826	-1.953485	-1.898825
C	4.547162	-0.846713	-3.703376
H	6.110439	0.430560	-2.940910
H	2.842611	-2.081153	-4.168582
H	4.959248	-0.891983	-4.707174
C	-1.511515	2.738805	0.764667
H	-1.826670	2.758418	-0.281462
H	-2.322634	2.368752	1.398252
O	1.344580	3.988590	1.886549

B

B3LYP/BSI SCF energy: -2418.533127a.u.

M06/BSII SCF energy in solution: -2417.419289a.u.

M06/BSII free energy in solution: -2416.616414a.u.

C	-1.540831	-0.163308	-1.172728
N	-1.462674	0.415160	-0.032930
O	-2.761165	-0.372212	-1.693571
C	-3.710569	0.411843	-0.854450
C	-2.852139	0.616250	0.455029
C	0.781839	-0.979942	-1.128746
N	1.244242	-0.314197	-0.124766
O	1.392572	-2.139225	-1.427023
C	2.263383	-2.463094	-0.269951
C	2.482786	-1.018209	0.313518
Ni	0.307823	1.185520	0.764609
C	-0.368673	-0.583391	-2.038429
C	-0.768470	-1.748528	-2.966775
H	-1.597906	-1.437868	-3.603335
H	-1.074534	-2.630728	-2.399921
H	0.076620	-2.028614	-3.598301
C	0.054231	0.648961	-2.890383
H	0.865846	0.369900	-3.569340
H	0.384524	1.470846	-2.251414
H	-0.795848	0.986224	-3.489359
C	3.516656	-3.156046	-0.780075
C	4.359099	-3.801541	0.136565
C	3.865617	-3.153409	-2.134411
C	5.531101	-4.421702	-0.292009
H	4.090675	-3.828643	1.188512
C	5.034735	-3.785079	-2.563907
H	3.220226	-2.663112	-2.853236
C	5.872474	-4.417635	-1.646202
H	6.173291	-4.914776	0.431911
H	5.288182	-3.780155	-3.620122
H	6.781825	-4.907231	-1.982133
C	1.430253	-3.388812	0.628341
C	0.855034	-2.981712	1.838169
C	1.191556	-4.699872	0.180191
C	0.083371	-3.870402	2.593686
H	0.983077	-1.971457	2.209285
C	0.416183	-5.581464	0.927750
H	1.626292	-5.028926	-0.758173
C	-0.135605	-5.170682	2.144574
H	-0.352239	-3.531562	3.528090
H	0.248661	-6.591419	0.564749
H	-0.731234	-5.860730	2.735386
C	-3.963619	1.704812	-1.645238
C	-3.273897	2.898602	-1.387630

C	-4.856665	1.665605	-2.729499
C	-3.488212	4.025214	-2.187815
H	-2.566053	2.980646	-0.569031
C	-5.064715	2.788469	-3.526816
H	-5.395853	0.749059	-2.945338
C	-4.381905	3.976846	-3.255885
H	-2.949226	4.941187	-1.964058
H	-5.763908	2.736233	-4.356676
H	-4.547969	4.856065	-3.871996
C	-4.983611	-0.397645	-0.671469
C	-5.135797	-1.684207	-1.197024
C	-6.046106	0.174215	0.043650
C	-6.327012	-2.389664	-1.005083
H	-4.325070	-2.132647	-1.759171
C	-7.229298	-0.533377	0.241104
H	-5.947502	1.179554	0.442558
C	-7.374977	-1.819977	-0.284204
H	-6.431290	-3.387160	-1.422754
H	-8.039960	-0.077767	0.802218
H	-8.299434	-2.370029	-0.134284
C	1.924258	1.917749	1.531937
H	1.649749	2.260099	2.534831
H	2.663340	1.117118	1.647883
C	2.531126	3.070730	0.729534
H	1.781741	3.860969	0.603775
H	2.803770	2.731314	-0.279616
C	3.783363	3.673292	1.394366
H	4.531519	2.882029	1.545335
H	3.513544	4.037363	2.395882
C	4.415738	4.818073	0.591636
H	4.694906	4.449645	-0.406634
H	3.664577	5.603939	0.426214

C	5.649687	5.433731	1.263354
H	6.400041	4.648368	1.430483
H	5.371445	5.802200	2.260157
C	6.276764	6.574865	0.455225
H	6.596959	6.229870	-0.535111
H	7.153857	6.993869	0.960477
H	5.560544	7.390453	0.302032
H	-2.937273	1.641736	0.806800
C	-3.154080	-0.292868	1.634545
C	-3.618326	0.275517	2.826893
C	-2.981084	-1.681433	1.571936
C	-3.922887	-0.526503	3.928145
H	-3.722357	1.354285	2.896790
C	-3.288809	-2.485374	2.668927
H	-2.610320	-2.142678	0.661720
C	-3.763594	-1.910229	3.850245
H	-4.277770	-0.067666	4.846479
H	-3.156155	-3.560986	2.599181
H	-4.003157	-2.537032	4.704776
H	2.513048	-1.053096	1.400915
C	3.751464	-0.328096	-0.169182
C	4.892015	-0.367312	0.643612
C	3.836470	0.308045	-1.412112
C	6.091766	0.203649	0.221714
H	4.839111	-0.843587	1.619258
C	5.035466	0.883411	-1.836544
H	2.961809	0.376358	-2.049749
C	6.167607	0.831045	-1.023227
H	6.963261	0.165625	0.868684
H	5.080779	1.379153	-2.802007
H	7.098807	1.282637	-1.352265
Br	-0.896492	3.041853	1.724289

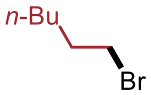

B3LYP/BSI SCF energy: -249.875786a.u.

M06/BSII SCF energy in solution: -249.69958a.u.

M06/BSII free energy in solution: -249.555544a.u.

C	-0.043382	-0.157970	0.000011
H	0.011685	-0.808644	0.880068
H	0.011669	-0.808752	-0.879969
C	-1.379011	0.607024	-0.000051
H	-1.424989	1.265116	-0.878855
H	-1.425085	1.265105	0.878752
C	-2.597457	-0.326168	-0.000105
H	-2.549556	-0.985380	-0.878222
H	-2.549403	-0.985683	0.877778
C	-3.936645	0.421923	0.000120
H	-3.982586	1.081432	-0.877180
H	-3.982486	1.081088	0.877684
C	-5.148636	-0.515054	0.000008
H	-5.148636	-1.162984	-0.883899
H	-6.088197	0.046837	0.000115
H	-5.148590	-1.163259	0.883710
C	1.133325	0.798683	-0.000056
H	1.176596	1.422527	0.891919
H	1.176602	1.422386	-0.892127
Br	2.907252	-0.189726	0.000019

[1]TS18

B3LYP/BSI SCF energy: -2418.505177a.u.

M06/BSII SCF energy in solution: -2417.385676a.u.

M06/BSII free energy in solution: -2416.595956a.u.

C	-1.845992	-1.188378	0.469756
N	-1.674191	-0.052125	-0.095338
O	-3.120882	-1.553020	0.739026
C	-3.987305	-0.603255	-0.009007
C	-2.997403	0.605407	-0.209359
C	0.620681	-1.737808	0.641376
N	1.048544	-0.745832	-0.055101
O	1.554834	-2.512784	1.235853
C	2.849650	-1.807701	1.115044
C	2.531280	-0.782437	-0.048865
Ni	-0.032829	0.558709	-1.065165
C	-0.804231	-2.218596	0.858723
C	-1.000665	-2.581396	2.354813
H	-2.016851	-2.946251	2.513270
H	-0.835486	-1.710505	2.995943
H	-0.292022	-3.357741	2.645783
C	-1.022601	-3.483406	-0.022140
H	-0.310011	-4.261337	0.262555
H	-0.888673	-3.246743	-1.081138
H	-2.037187	-3.859993	0.122567
C	3.933335	-2.823130	0.791977
C	5.258422	-2.382792	0.657403
C	3.653198	-4.182902	0.628282
C	6.277293	-3.282420	0.353355
H	5.494353	-1.331674	0.795276
C	4.677162	-5.085524	0.328068
H	2.635107	-4.536494	0.739315
C	5.990037	-4.639842	0.187075
H	7.296786	-2.923269	0.247615
H	4.442678	-6.139193	0.206104

H	6.784826	-5.341656	-0.047958
C	3.066650	-1.125994	2.476129
C	2.547580	0.148635	2.754757
C	3.706730	-1.824860	3.510957
C	2.683922	0.710835	4.025748
H	2.022888	0.716687	1.993902
C	3.841721	-1.262563	4.779740
H	4.105457	-2.815332	3.321567
C	3.333988	0.010727	5.041629
H	2.280555	1.701225	4.216265
H	4.346699	-1.821186	5.562600
H	3.443804	0.452535	6.027806
C	-4.340560	-1.322529	-1.321409
C	-3.641585	-1.105515	-2.519005
C	-5.345144	-2.304036	-1.300198
C	-3.954376	-1.843197	-3.664727
H	-2.846865	-0.369870	-2.590179
C	-5.651812	-3.040476	-2.442146
H	-5.893253	-2.486817	-0.381723
C	-4.958165	-2.809573	-3.632465
H	-3.405436	-1.651320	-4.581890
H	-6.436632	-3.790668	-2.403232
H	-5.200929	-3.377176	-4.526334
C	-5.218981	-0.295779	0.824206
C	-5.422406	-0.846915	2.092891
C	-6.189361	0.567790	0.295163
C	-6.572205	-0.534138	2.823366
H	-4.683018	-1.521526	2.508240
C	-7.331307	0.883935	1.027217
H	-6.052275	0.989726	-0.696317
C	-7.527631	0.332889	2.296229
H	-6.717153	-0.972495	3.806795

H	-8.070058	1.558827	0.604888
H	-8.419792	0.576579	2.865686
C	1.178110	3.043183	0.383835
H	0.306300	3.279729	-0.215593
H	1.014346	2.863460	1.443718
C	2.539272	3.397294	-0.118988
H	2.616119	3.135766	-1.182678
H	3.310647	2.821140	0.414275
C	2.875520	4.903430	0.035584
H	2.784603	5.186157	1.093352
H	2.122558	5.489768	-0.506485
C	4.276267	5.264990	-0.474517
H	5.026389	4.669171	0.066423
H	4.363211	4.976325	-1.531675
C	4.613652	6.754282	-0.327616
H	4.519536	7.043607	0.728144
H	3.867148	7.348205	-0.871930
C	6.016048	7.110600	-0.831767
H	6.787081	6.556582	-0.283671
H	6.226446	8.178662	-0.712067
H	6.128623	6.867025	-1.894439
H	-3.091864	1.007246	-1.217458
C	-3.092078	1.774899	0.756729
C	-3.348709	3.054438	0.251607
C	-2.895396	1.620460	2.136004
C	-3.421689	4.157032	1.105519
H	-3.471161	3.190918	-0.819278
C	-2.968723	2.719250	2.990339
H	-2.689039	0.637405	2.548260
C	-3.233896	3.991768	2.477399
H	-3.616414	5.143490	0.694827
H	-2.820006	2.582560	4.057893

H	-3.288553	4.847815	3.143940
H	2.896828	0.205344	0.233478
C	3.068603	-1.086542	-1.436023
C	4.025216	-0.236041	-2.001050
C	2.616397	-2.183942	-2.180772
C	4.530016	-0.478609	-3.279840
H	4.368897	0.630936	-1.442539
C	3.118336	-2.428397	-3.457263
H	1.865641	-2.848746	-1.765269
C	4.077912	-1.576774	-4.010389
H	5.266252	0.196425	-3.706131
H	2.756267	-3.281288	-4.024026
H	4.462943	-1.764830	-5.008336
Br	-0.555481	1.951223	-2.925476

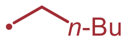

B3LYP/BSI SCF energy: -236.426557a.u.

M06/BSII SCF energy in solution: -236.272528a.u.

M06/BSII free energy in solution: -236.132027a.u.

C	-3.276117	-0.184447	0.000058
H	-3.718950	-0.534864	0.927230
H	-3.718983	-0.534990	-0.927058
C	-1.972599	0.540901	-0.000029
H	-1.906258	1.195234	0.880368
H	-1.906314	1.195170	-0.880483
C	-0.736651	-0.396520	-0.000053
H	-0.786203	-1.054222	-0.877996
H	-0.786222	-1.054242	0.877869
C	0.594685	0.365146	-0.000012
H	0.635718	1.026108	-0.877837
H	0.635689	1.026095	0.877823
C	1.823994	-0.552653	0.000007
H	1.782325	-1.212635	-0.877179
H	1.782301	-1.212646	0.877183
C	3.150787	0.213696	0.000027
H	3.237388	0.856247	-0.883718
H	4.007547	-0.468222	0.000029
H	3.237369	0.856228	0.883787

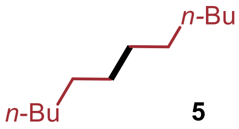

5

B3LYP/BSI SCF energy: -472.995274a.u.

M06/BSII SCF energy in solution: -472.693751a.u.

M06/BSII free energy in solution: -472.37841a.u.

C	0.000173	0.767024	0.000000
H	0.554567	1.129557	0.877640
H	0.554567	1.129557	-0.877640
C	-1.405366	1.381293	0.000000
H	-1.959716	1.018690	0.877630
H	-1.959716	1.018690	-0.877630
C	-1.405366	2.915338	0.000000
H	-0.851031	3.277995	-0.877661
H	-0.851031	3.277995	0.877661
C	-2.810831	3.529452	0.000000
H	-3.365769	3.167441	-0.877664
H	-3.365769	3.167441	0.877664
C	-2.811783	5.063500	0.000000
H	-2.257690	5.425045	-0.877181
H	-2.257690	5.425045	0.877181
C	-4.219363	5.668344	0.000000
H	-4.786282	5.353214	-0.883689

H	-4.186003	6.762936	0.000000
H	-4.786282	5.353214	0.883689
C	-0.000173	-0.767024	0.000000
H	-0.554567	-1.129557	0.877640
H	-0.554567	-1.129557	-0.877640
C	1.405366	-1.381293	0.000000
H	1.959716	-1.018690	0.877630
H	1.959716	-1.018690	-0.877630
C	1.405366	-2.915338	0.000000
H	0.851031	-3.277995	-0.877661
H	0.851031	-3.277995	0.877661
C	2.810831	-3.529452	0.000000
H	3.365769	-3.167441	-0.877664
H	3.365769	-3.167441	0.877664
C	2.811783	-5.063500	0.000000
H	2.257690	-5.425045	-0.877181
H	2.257690	-5.425045	0.877181
C	4.219363	-5.668344	0.000000
H	4.786282	-5.353214	-0.883689
H	4.186003	-6.762936	0.000000
H	4.786282	-5.353214	0.883689

$^{3}LNi^{II}Cl_2$

B3LYP/BSI SCF energy: -1665.537666a.u.

M06/BSII SCF energy in solution: -1665.266492a.u.

M06/BSII free energy in solution: -1665.097161a.u.

C	-0.743161	1.578127	-0.000067
C	-2.664553	0.225907	-0.000422
C	-3.483361	1.361757	-0.000352
C	-2.901909	2.625110	-0.000097
C	-1.514287	2.742328	0.000047
C	0.743158	1.578129	0.000070
C	2.664551	0.225911	0.000425
C	3.483358	1.361761	0.000357
C	2.901905	2.625114	0.000103
C	1.514282	2.742330	-0.000042
H	-4.561084	1.242427	-0.000491
H	-3.523293	3.515388	-0.000019
H	-1.048936	3.719947	0.000262
H	4.561081	1.242433	0.000496
H	3.523288	3.515393	0.000026
H	1.048930	3.719949	-0.000256
C	3.216067	-1.170336	0.000730
H	2.861966	-1.714337	0.883383
H	2.862352	-1.714547	-0.881947
H	4.307996	-1.163399	0.000983
C	-3.216066	-1.170341	-0.000729
H	-2.861969	-1.714337	-0.883386
H	-2.862346	-1.714554	0.881944
H	-4.307996	-1.163405	-0.000977
N	1.325704	0.359415	0.000269
N	-1.325706	0.359413	-0.000266
Ni	0.000001	-1.190432	-0.000001
Cl	0.001198	-1.994857	-2.088766
Cl	-0.001193	-1.994875	2.088758

[Si]H

B3LYP/BSI SCF energy: -753.57108a.u.

M06/BSII SCF energy in solution: -753.392347a.u.

M06/BSII free energy in solution: -753.215797a.u.

Si	0.000061	-0.000084	0.448361
H	0.000049	-0.000175	1.936726

O	-1.273053	-0.875168	-0.121041
O	1.394561	-0.664971	-0.121013
O	-0.121318	1.540077	-0.120910
C	-1.470409	-2.265196	0.147756
H	-0.688764	-2.847802	-0.356319
H	-1.384081	-2.459306	1.227418
C	-2.846596	-2.676201	-0.350052
H	-2.935894	-2.487281	-1.423794
H	-3.016039	-3.742936	-0.169226
H	-3.627908	-2.108364	0.163918
C	-1.226217	2.406011	0.148725
H	-2.122160	2.019677	-0.353795
H	-1.436010	2.429129	1.228655
C	-0.895120	3.802988	-0.350724
H	-0.688450	3.785003	-1.424759
H	-1.734115	4.483047	-0.169236
H	-0.012041	4.196371	0.161604
C	2.696914	-0.141037	0.148638
H	2.810419	0.827970	-0.353987
H	2.821738	0.029267	1.228552
C	3.741196	-1.126371	-0.350568
H	3.622346	-1.296583	-1.424576
H	4.749636	-0.739777	-0.169116
H	3.640302	-2.087730	0.161945

LiF

B3LYP/BSI SCF energy: -107.416714a.u.

M06/BSII SCF energy in solution: -107.468704a.u.

M06/BSII free energy in solution: -107.485726a.u.

F	0.000000	0.000000	0.387959
Li	0.000000	0.000000	-1.163878

2a

B3LYP/BSI SCF energy: -761.209091a.u.

M06/BSII SCF energy in solution: -761.002738a.u.

M06/BSII free energy in solution: -760.87066a.u.

C	-2.278847	1.844061	0.606077
H	-1.693137	2.702570	0.915347
H	-3.356850	1.944078	0.623643
C	-1.688212	0.702661	0.227948
C	-2.566717	-0.476462	-0.129706
C	-0.220108	0.519815	0.123588
C	0.404278	-0.709814	0.379059
C	0.602163	1.605959	-0.237127
C	1.789539	-0.856547	0.297836
H	-0.192020	-1.569443	0.660351
C	1.979348	1.475362	-0.317887
H	0.148083	2.560913	-0.482824
C	2.587802	0.239382	-0.048802
H	2.229699	-1.823813	0.508871
H	2.608261	2.311569	-0.605111
O	3.944781	0.209280	-0.161504
C	4.612594	-1.021917	0.070435
H	5.674045	-0.822010	-0.082818
H	4.457482	-1.379532	1.096301
H	4.285761	-1.797811	-0.633521
F	-2.488566	-1.459429	0.808026
F	-3.870217	-0.145131	-0.229811
F	-2.202396	-1.032748	-1.304518

^{1}TS1-A

B3LYP/BSI SCF energy: -2526.568348a.u.

M06/BSII SCF energy in solution: -2526.126315a.u.

M06/BSII free energy in solution: -2525.74973a.u.

C	3.285849	1.325157	-0.017931
C	1.532819	2.835151	0.295143
C	2.418102	3.916523	0.187891
C	3.767670	3.678359	-0.037484
C	4.213284	2.365493	-0.141777
C	3.690650	-0.104076	-0.105908
C	2.973638	-2.334665	-0.129477
C	4.293987	-2.779433	-0.260386
C	5.327981	-1.852755	-0.308365
C	5.027216	-0.497834	-0.229018
H	2.036993	4.927869	0.278673
H	4.467368	4.503483	-0.131395
H	5.260701	2.163204	-0.323860
H	4.492676	-3.843782	-0.320232
H	6.359010	-2.179469	-0.404534
H	5.823356	0.234447	-0.255614
C	1.826188	-3.299745	-0.068833
H	1.299648	-3.189905	0.884802
H	1.118884	-3.077646	-0.874195
H	2.177754	-4.329656	-0.165008
C	0.068080	3.062316	0.529336
H	-0.522856	2.636741	-0.286466
H	-0.257147	2.564070	1.446646
H	-0.147768	4.130366	0.613019
N	2.698202	-1.017293	-0.054664
N	1.973572	1.567111	0.187827
Ni	0.794804	-0.277475	0.123170
Si	-2.636228	0.335520	0.259075
H	-0.751423	0.206977	0.334023
O	-2.462425	-1.316875	0.612484
O	-4.300771	0.511014	0.208621
O	-2.235786	1.200034	-1.071464
C	-5.181160	0.223156	1.288911
H	-5.020330	0.945460	2.100674
H	-4.975740	-0.776744	1.699669
C	-6.619293	0.296825	0.797487
H	-7.316698	0.093326	1.617089
H	-6.836116	1.291719	0.397150
H	-6.795273	-0.435055	0.002995
C	-3.110811	1.712157	-2.090722
H	-4.047631	2.057027	-1.643481
H	-2.587488	2.574621	-2.517211
C	-3.368714	0.666460	-3.164606
H	-3.953344	-0.166601	-2.760332
H	-3.936384	1.105582	-3.992967
H	-2.422180	0.276355	-3.547788
C	-3.249030	-2.388110	0.055898
H	-3.695429	-2.935809	0.894916
H	-4.067772	-1.960657	-0.532352
C	-2.382038	-3.304647	-0.792564
H	-1.889692	-2.742158	-1.590539
H	-1.605637	-3.776367	-0.182232
H	-2.997327	-4.095869	-1.235642
Cl	0.407156	-0.722210	-2.089113
Cl	0.900690	-0.885984	2.523541
Li	-1.221006	-0.741797	2.032356
F	-2.324989	0.923453	1.795690

^{3}TS1-A

B3LYP/BSI SCF energy: -2526.586206a.u.

M06/BSII SCF energy in solution: -2526.14201a.u.

M06/BSII free energy in solution: -2525.767971a.u.

C	3.320400	1.330342	0.067509
C	1.510262	2.814090	0.147104
C	2.387109	3.906088	0.195650
C	3.758287	3.690253	0.188196
C	4.234807	2.386351	0.118634
C	3.762113	-0.086229	-0.038685
C	3.077869	-2.319495	-0.172588
C	4.409125	-2.744921	-0.252649
C	5.430868	-1.803189	-0.222388
C	5.109677	-0.454274	-0.113517
H	1.979819	4.910135	0.237599
H	4.450766	4.525650	0.229108
H	5.300423	2.199956	0.102138
H	4.626791	-3.804041	-0.336440
H	6.469554	-2.113759	-0.283403
H	5.896987	0.287870	-0.091526
C	1.934229	-3.290907	-0.190732
H	1.375262	-3.216879	0.748530
H	1.253912	-3.040809	-1.011398
H	2.290988	-4.316348	-0.311702
C	0.025978	3.031666	0.144327
H	-0.449168	2.488512	-0.675797
H	-0.424061	2.653608	1.066731
H	-0.200356	4.097324	0.057421
N	2.783638	-1.010468	-0.070359
N	1.984493	1.551653	0.095782
Ni	0.836709	-0.208107	0.041601
Si	-2.771179	0.342241	0.293587
H	-0.744696	0.255525	0.335326
O	-2.509693	-1.268452	0.725152
O	-4.433411	0.475137	0.270203
O	-2.381057	1.105071	-1.093566
C	-5.282636	0.192167	1.379382
H	-5.125386	0.942990	2.165424
H	-5.035126	-0.788265	1.811824
C	-6.731646	0.208423	0.916983
H	-7.404850	0.007170	1.757015
H	-6.987555	1.184444	0.494039
H	-6.900895	-0.551114	0.147469
C	-3.264593	1.559545	-2.134229
H	-4.221980	1.870015	-1.705714
H	-2.775819	2.437289	-2.570276
C	-3.458068	0.482980	-3.190811
H	-4.008309	-0.370460	-2.780358
H	-4.034783	0.880889	-4.033607
H	-2.489303	0.131837	-3.555579
C	-3.213376	-2.407616	0.190553
H	-3.624660	-2.961415	1.042702
H	-4.056895	-2.054677	-0.412641
C	-2.279668	-3.284400	-0.628728
H	-1.827818	-2.717156	-1.447212
H	-1.473122	-3.678840	-0.002903
H	-2.835356	-4.132394	-1.044670
Cl	0.457319	-0.673514	-2.199585
Cl	0.960875	-0.928438	2.508032
Li	-1.129694	-0.593147	1.983234
F	-2.393776	1.041514	1.753690

^{3}TS1-B

B3LYP/BSI SCF energy: -2426.671085a.u.

M06/BSII SCF energy in solution: -2426.202335a.u.

M06/BSII free energy in solution: -2425.878024a.u.

C	-3.577013	0.848302	-0.705396
C	-2.462360	2.864855	-0.286134
C	-3.467033	3.579394	-0.951664
C	-4.550029	2.899933	-1.493305
C	-4.611151	1.516008	-1.369232
C	-3.597692	-0.631112	-0.519331
C	-2.737883	-2.457188	0.655915
C	-3.664127	-3.314732	0.047759
C	-4.546724	-2.806218	-0.896625
C	-4.523279	-1.444708	-1.181959
H	-3.388244	4.658088	-1.029317
H	-5.343408	3.439381	-2.001839
H	-5.460338	0.974426	-1.764870
H	-3.676278	-4.365733	0.315876
H	-5.255809	-3.458011	-1.398077
H	-5.208877	-1.032443	-1.910863
C	-1.764701	-2.981361	1.675359
H	-0.809544	-3.217591	1.196334
H	-1.581637	-2.237549	2.453666
H	-2.147404	-3.895751	2.135859
C	-1.283191	3.572302	0.316240
H	-1.223369	3.351377	1.386759
H	-0.352118	3.223062	-0.141291
H	-1.362818	4.651568	0.168304
N	-2.699719	-1.147388	0.347066
N	-2.534294	1.521322	-0.171994
Ni	-1.085255	0.355045	0.882296
C	0.734505	-0.513968	1.202285
H	1.181834	0.131719	1.956691
H	0.599293	-1.517524	1.607479
C	1.537105	-0.538094	-0.043191
C	1.325237	-1.841952	-0.835164
C	2.998150	-0.150204	0.004438
C	3.727050	0.219334	-1.135483
C	3.695058	-0.239790	1.223072
C	5.088627	0.505414	-1.072069
H	3.220269	0.307598	-2.088639
C	5.053672	0.035350	1.300105
H	3.170523	-0.543700	2.120978
C	5.763216	0.413932	0.153176
H	5.607889	0.801979	-1.975533
H	5.586927	-0.036767	2.242157
O	7.088766	0.671093	0.329521
C	7.857593	1.056907	-0.799838
H	8.874962	1.204829	-0.434632
H	7.861197	0.277612	-1.572490
H	7.491578	1.994429	-1.237328
F	1.879426	-2.865244	-0.143026
F	0.015791	-2.147803	-0.995823
F	1.881405	-1.841417	-2.058047
Cl	0.646119	0.820463	-1.296729
Cl	-1.436078	1.267608	2.969855

^{3}TS1-C

B3LYP/BSI SCF energy: -2419.054739a.u.

M06/BSII SCF energy in solution: -2418.618544a.u.

M06/BSII free energy in solution: -2418.245688a.u.

C	-2.986110	0.839682	-0.556986
C	-1.751298	2.635596	0.324353
C	-2.632265	3.548936	-0.268209
C	-3.706327	3.080957	-1.014570
C	-3.889698	1.708494	-1.167364

C	-3.067776	-0.637838	-0.672153
C	-2.075286	-2.678164	-0.056390
C	-3.045497	-3.385972	-0.774156
C	-4.041224	-2.689114	-1.449586
C	-4.059379	-1.298155	-1.399139
H	-2.458763	4.611681	-0.142767
H	-4.395780	3.777246	-1.481766
H	-4.716412	1.331347	-1.755540
H	-3.007856	-4.469250	-0.795395
H	-4.800387	-3.223577	-2.012117
H	-4.829060	-0.743508	-1.920062
C	-0.973077	-3.368270	0.694383
H	-0.001055	-3.117457	0.256404
H	-0.960196	-3.043277	1.740043
H	-1.097685	-4.452537	0.663624
C	-0.543086	3.067756	1.099473
H	-0.521336	2.582152	2.080953
H	0.362142	2.777199	0.550354
H	-0.535186	4.150377	1.242149
N	-2.105753	-1.332849	-0.023805
N	-1.955619	1.314118	0.177681
Ni	-0.805212	-0.141417	0.963975
Si	1.977480	0.045948	-0.494570
H	0.746406	-0.578317	0.297656
O	2.912274	-0.321136	0.906787
O	2.705264	-0.945824	-1.623348
O	2.257603	1.696587	-0.450543
C	3.988640	-1.558480	-1.530231
H	3.847165	-2.640851	-1.398154
H	4.540053	-1.189901	-0.657658
C	4.771232	-1.293479	-2.810560
H	5.740109	-1.805442	-2.780879
H	4.213820	-1.651530	-3.680961
H	4.948154	-0.221398	-2.939754
C	3.269219	2.370817	0.301808
H	2.920851	3.405441	0.418564
H	3.366414	1.925819	1.296298
C	4.612419	2.360203	-0.417327
H	4.512171	2.768573	-1.427644
H	5.344692	2.964200	0.131223
H	5.002337	1.340522	-0.491698
C	2.827339	-1.517268	1.645114
H	1.771730	-1.795845	1.811491
H	3.293769	-2.351387	1.095622
C	3.515853	-1.342883	2.993841
H	4.567781	-1.073138	2.856093
H	3.031022	-0.548862	3.569828
H	3.469675	-2.270730	3.574837
Cl	-1.229429	-0.181278	3.136787
Cl	0.326665	0.445751	-2.052210

^{1}IM1-A

B3LYP/BSI SCF energy: -2526.604169a.u.

M06/BSII SCF energy in solution: -2526.162861a.u.

M06/BSII free energy in solution: -2525.791218a.u.

C	-3.924951	1.394273	0.202240
C	-2.016464	2.743453	-0.061633
C	-2.831795	3.882704	-0.120171
C	-4.208282	3.768947	0.024318
C	-4.768957	2.502964	0.168419
C	-4.416343	-0.003355	0.175840
C	-3.776950	-2.205944	-0.297841
C	-5.123961	-2.601750	-0.291184

C -6.118815 -1.678670 0.002716
C -5.767146 -0.347599 0.216291
H -2.368264 4.849210 -0.283217
H -4.842186 4.649785 -0.005865
H -5.843285 2.381128 0.226624
H -5.371191 -3.632226 -0.522259
H -7.161299 -1.980926 0.031994
H -6.529491 0.400284 0.396369
C -2.692311 -3.173628 -0.671626
H -1.913405 -2.668520 -1.249513
H -2.212082 -3.583501 0.220882
H -3.112481 -3.995356 -1.258010
C -0.534337 2.850473 -0.261299
H 0.003180 2.679220 0.676235
H -0.191731 2.087284 -0.967943
H -0.275535 3.844009 -0.636658
N -3.444007 -0.932201 -0.005895
N -2.571107 1.525497 0.143634
Ni -1.680389 -0.103508 0.594437
Si 3.678255 0.394646 0.146032
H -0.560448 0.631504 1.128500
O 2.680556 -0.841293 0.620766
O 4.431238 0.200306 -1.282379
O 4.797661 0.569887 1.319795
C 3.833842 0.355162 -2.594788
H 3.871209 1.419316 -2.856388
H 2.783881 0.043822 -2.572340
C 4.627279 -0.471684 -3.589608
H 4.215683 -0.337609 -4.595236
H 5.679600 -0.170330 -3.601076
H 4.574037 -1.535051 -3.337686
C 6.019829 1.315855 1.203551
H 6.450611 1.164085 0.207179
H 5.794106 2.383840 1.317267
C 6.981345 0.857973 2.286666
H 7.217649 -0.203730 2.168786
H 7.914161 1.428702 2.230312
H 6.543874 1.005901 3.278276
C 3.142682 -2.039504 1.303423
H 3.946718 -1.755367 1.988146
H 2.289187 -2.386256 1.891044
C 3.596111 -3.098510 0.311566
H 2.785479 -3.357122 -0.376176
H 4.450855 -2.751924 -0.277530
H 3.894269 -4.004801 0.849941
Cl -0.677669 -1.846528 1.569211
Cl 0.193517 -0.484409 -2.028318
Li 0.808779 -0.770704 0.072217
F 2.670217 1.646070 0.061881

^{3}IM1-A

B3LYP/BSI SCF energy: -2526.60516a.u.

M06/BSII SCF energy in solution: -2526.158808a.u.

M06/BSII free energy in solution: -2525.791667a.u.

C -3.772275 0.950215 -0.867271
C -2.572525 2.878400 -0.296796
C -3.439886 3.665983 -1.065114
C -4.479101 3.063847 -1.763375
C -4.659641 1.689313 -1.655441
C -3.942623 -0.509324 -0.631445
C -3.091070 -2.403130 0.444250
C -4.157727 -3.177088 -0.029593
C -5.128907 -2.586926 -0.829639

C	-5.026161	-1.234121	-1.137807
H	-3.290553	4.739552	-1.102053
H	-5.153746	3.658646	-2.371898
H	-5.479103	1.207059	-2.172418
H	-4.211781	-4.228244	0.232045
H	-5.962293	-3.171439	-1.207847
H	-5.781451	-0.759303	-1.750784
C	-2.003478	-2.990274	1.295237
H	-1.052172	-2.941929	0.753580
H	-1.898434	-2.405732	2.215119
H	-2.217723	-4.032402	1.544198
C	-1.464436	3.507621	0.498883
H	-1.474205	3.128488	1.524097
H	-0.488240	3.245438	0.081317
H	-1.570958	4.595349	0.505281
N	-3.005028	-1.096186	0.137510
N	-2.730968	1.540713	-0.237739
Ni	-1.418217	0.191523	0.696458
Si	3.938321	0.210133	-0.331987
H	-0.061450	1.039709	0.909900
O	2.884978	-0.341324	0.805976
O	4.611835	-0.902152	-1.306066
O	5.077989	1.129384	0.363629
C	3.884545	-1.689918	-2.282807
H	3.797309	-1.095004	-3.198610
H	2.871580	-1.899320	-1.920918
C	4.647890	-2.975763	-2.538391
H	4.130744	-3.570451	-3.298153
H	5.660381	-2.762875	-2.894135
H	4.720654	-3.571932	-1.624030
C	6.285259	1.623140	-0.250677
H	6.642395	0.899961	-0.992222
H	6.046730	2.558815	-0.769952
C	7.327601	1.856987	0.827864
H	7.574518	0.920831	1.336861
H	8.242740	2.261568	0.383384
H	6.959799	2.569312	1.571797
C	3.193925	-0.729018	2.171207
H	4.032677	-1.433959	2.150274
H	3.510988	0.167995	2.712223
C	1.967495	-1.355524	2.805541
H	1.125013	-0.657582	2.825381
H	1.665851	-2.253562	2.258293
H	2.198350	-1.642518	3.836870
Cl	-2.031114	0.250515	2.944583
Cl	-0.150395	-1.020653	-1.283350
Li	1.181965	0.285454	-0.067128
F	2.857328	1.073284	-1.200513

$^{1}LNi^{II}ClH$

B3LYP/BSI SCF energy: -1205.847476a.u.

M06/BSII SCF energy in solution: -1205.576353a.u.

M06/BSII free energy in solution: -1205.395089a.u.

C	-0.334790	1.486663	-0.116879
C	-2.455742	0.694656	0.460796
C	-3.011909	1.949013	0.166458
C	-2.205226	2.971725	-0.314856
C	-0.837428	2.748063	-0.441848
C	1.113554	1.177513	-0.105616
C	2.728552	-0.487088	0.256348
C	3.744493	0.470953	0.162804
C	3.432491	1.800940	-0.092401
C	2.095753	2.162947	-0.213747

H -4.074454 2.103675 0.319333
H -2.629300 3.938717 -0.567946
H -0.183111 3.536763 -0.791776
H 4.774234 0.158475 0.296146
H 4.215407 2.548672 -0.171739
H 1.820746 3.198782 -0.366430
C 3.072172 -1.919436 0.551404
H 2.413111 -2.327467 1.320824
H 2.935895 -2.541502 -0.338440
H 4.110875 -1.997935 0.883475
C -3.310462 -0.403462 1.018234
H -3.426917 -1.206098 0.284376
H -2.828079 -0.857881 1.887842
H -4.289613 -0.015979 1.309838
N 1.429415 -0.132168 0.093429
N -1.138266 0.464940 0.270314
Ni -0.068947 -1.302092 -0.114257
Cl -1.641661 -2.703047 -0.639101
H 0.773182 -2.372163 -0.531983

^{3}LNi^{II}ClH

B3LYP/BSI SCF energy: -1205.858244a.u.

M06/BSII SCF energy in solution: -1205.580644a.u.

M06/BSII free energy in solution: -1205.404303a.u.

C -0.742577 1.415569 0.064049
C -2.664983 0.092937 -0.180736
C -3.482864 1.208473 0.030374
C -2.899786 2.451081 0.259905
C -1.512648 2.562665 0.277391
C 0.742541 1.415583 0.064050
C 2.664972 0.092990 -0.180755
C 3.482832 1.208538 0.030375
C 2.899731 2.451131 0.259928
C 1.512591 2.562689 0.277414
H -4.560960 1.091736 0.014150
H -3.519539 3.327052 0.425425
H -1.045434 3.523070 0.456037
H 4.560931 1.091821 0.014149
H 3.519468 3.327110 0.425466
H 1.045359 3.523081 0.456081
C 3.220443 -1.277637 -0.439823
H 2.837885 -1.988902 0.299968
H 2.895476 -1.633637 -1.424092
H 4.312130 -1.275867 -0.406438
C -3.220428 -1.277705 -0.439783
H -2.895462 -1.633709 -1.424051
H -2.837849 -1.988954 0.300012
H -4.312114 -1.275958 -0.406388
N 1.324409 0.217179 -0.161745
N -1.324422 0.217151 -0.161727
Ni 0.000007 -1.324416 -0.511033
Cl 0.000063 -2.832602 1.163419
H -0.000016 -1.327149 -2.069559

[Si]F-LiCl

B3LYP/BSI SCF energy: -1320.722383a.u.

M06/BSII SCF energy in solution: -1320.559462a.u.

M06/BSII free energy in solution: -1320.388965a.u.

Cl -3.600969 0.068510 -0.928495
Si 0.675996 -0.080634 -0.367760
O 0.608810 1.349705 0.387500
O -0.403412 -1.227124 0.134931

O	2.181640	-0.671564	-0.379848
C	-0.647191	-1.627292	1.511282
H	-0.776544	-0.724118	2.116917
H	0.238928	-2.164039	1.866222
C	-1.887656	-2.497944	1.559274
H	-2.087064	-2.797403	2.593211
H	-2.755528	-1.950005	1.178956
H	-1.747055	-3.405667	0.963630
C	3.406872	0.076944	-0.537144
H	3.589089	0.210195	-1.609731
H	3.292288	1.066357	-0.081283
C	4.540656	-0.695597	0.111404
H	4.638756	-1.687662	-0.338145
H	5.484729	-0.158326	-0.024858
H	4.363188	-0.816800	1.183859
C	-0.496037	2.297031	0.323533
H	-0.342178	2.924110	-0.560938
H	-1.445149	1.762364	0.200888
C	-0.495157	3.131288	1.589721
H	-0.659003	2.503690	2.470747
H	0.455933	3.658279	1.710895
H	-1.299922	3.871545	1.542561
F	0.040384	0.054612	-1.874599
Li	-1.728875	-0.844849	-1.299189

[1]TS2-A

B3LYP/BSI SCF energy: -2066.89789a.u.

M06/BSII SCF energy in solution: -2066.448405a.u.

M06/BSII free energy in solution: -2066.068224a.u.

C	3.157798	1.342509	-0.293273
C	1.549418	2.791892	0.554927
C	2.262073	3.903373	0.086017
C	3.449888	3.703522	-0.611998
C	3.911685	2.405901	-0.808278
C	3.588055	-0.073261	-0.441804
C	2.959012	-2.330560	-0.398862
C	4.261614	-2.722364	-0.723943
C	5.247843	-1.756461	-0.892232
C	4.909093	-0.415742	-0.748896
H	1.883066	4.904181	0.266265
H	4.009978	4.548821	-1.001100
H	4.822785	2.228609	-1.367249
H	4.487670	-3.777569	-0.832531
H	6.268448	-2.043052	-1.127458
H	5.663749	0.354128	-0.851444
C	1.866207	-3.337352	-0.183253
H	1.588016	-3.363673	0.875672
H	0.970480	-3.047652	-0.742631
H	2.185682	-4.336206	-0.491976
C	0.252959	2.938340	1.304040
H	-0.564840	2.486764	0.732273
H	0.319028	2.415094	2.263906
H	0.017874	3.989094	1.493344
N	2.642618	-1.024602	-0.261105
N	1.996759	1.541759	0.353752
Ni	0.801207	-0.483837	0.250261
Si	-2.625156	-0.213328	0.111057
H	-0.766804	-0.246904	0.468620
O	-4.300169	-0.250997	-0.024180
O	-2.282218	-1.711222	-0.495508
C	-5.166825	0.702462	0.576690
H	-4.836979	1.725821	0.342837
H	-5.136323	0.596500	1.670317

C -6.585240 0.482406 0.070842
H -7.275135 1.196235 0.533811
H -6.633566 0.609061 -1.015347
H -6.923315 -0.530536 0.309657
C -3.260980 -2.651785 -0.967191
H -3.958543 -2.900728 -0.159699
H -3.850129 -2.204792 -1.775646
C -2.532216 -3.893037 -1.456041
H -1.953380 -4.346856 -0.645668
H -3.250352 -4.633708 -1.824931
H -1.844603 -3.641208 -2.269016
Cl 1.162969 -1.060546 2.626473
Li -0.906113 -0.547991 2.271583
H 0.464080 -0.385597 -1.176611
F -2.584784 -0.096313 1.809217
O -2.218805 1.241808 -0.574357
C -2.746878 1.707699 -1.821008
H -2.147169 1.281138 -2.635469
H -3.781150 1.365083 -1.948876
C -2.676734 3.226469 -1.859485
H -1.643246 3.566541 -1.744030
H -3.057701 3.600047 -2.816174
H -3.275036 3.664597 -1.054376

^{3}TS2-A

B3LYP/BSI SCF energy: -2066.905646a.u.

M06/BSII SCF energy in solution: -2066.44924a.u.

M06/BSII free energy in solution: -2066.071552a.u.

C 3.315886 1.246860 0.299880
C 1.486738 2.682246 0.547928
C 2.324680 3.702561 1.013001
C 3.690041 3.471023 1.131166
C 4.196257 2.230429 0.762683
C 3.790732 -0.089006 -0.141209
C 3.149085 -2.225692 -0.845876
C 4.486426 -2.610116 -0.982799
C 5.492315 -1.691983 -0.694801
C 5.146316 -0.413714 -0.269570
H 1.895745 4.662424 1.279241
H 4.353369 4.247084 1.500904
H 5.256492 2.029197 0.846856
H 4.724546 -3.616650 -1.309729
H 6.537068 -1.967793 -0.801549
H 5.919179 0.313112 -0.053809
C 2.009635 -3.162628 -1.124116
H 1.445057 -3.337683 -0.201654
H 1.325262 -2.711484 -1.851908
H 2.364713 -4.120563 -1.511233
C 0.012227 2.912197 0.388746
H -0.292364 2.696303 -0.638974
H -0.567666 2.248427 1.033953
H -0.244682 3.947757 0.624135
N 2.829738 -0.983915 -0.439692
N 1.982871 1.472456 0.215938
Ni 0.876722 -0.233402 -0.365655
Si -2.906986 0.025483 0.030317
H -0.864742 -0.014085 -0.186231
O -4.534375 -0.007871 0.382018
O -2.537758 -1.597713 0.305901
C -5.425500 1.093781 0.244881
H -5.312806 1.545331 -0.748624
H -5.180545 1.864530 0.990878
C -6.852884 0.608657 0.450455

H -7.558109 1.442366 0.364087

H -7.114070 -0.144300 -0.299666

H -6.968803 0.158921 1.441414

C -3.535895 -2.629400 0.500572

H -4.164679 -2.369219 1.357531

H -4.182152 -2.665253 -0.381627

C -2.826167 -3.955608 0.709496

H -2.176601 -3.922637 1.590526

H -3.561194 -4.753949 0.857953

H -2.210965 -4.208483 -0.159094

Cl 0.932777 -1.332719 2.055002

Li -1.103879 -0.910909 1.491397

H 0.844388 -0.071405 -1.935124

F -2.345171 0.766309 1.417854

O -2.939907 0.834482 -1.389120

C -2.049256 0.979301 -2.499675

H -1.016663 0.814734 -2.182288

H -2.298513 0.200276 -3.231987

C -2.240266 2.356745 -3.115837

H -1.986005 3.145189 -2.399809

H -1.591713 2.465824 -3.991606

H -3.277174 2.507788 -3.432262

$^{1}LNi^{II}H_2$

B3LYP/BSI SCF energy: -746.188992a.u.

M06/BSII SCF energy in solution: -745.910085a.u.

M06/BSII free energy in solution: -745.721484a.u.

C -0.740747 1.047394 -0.033983

C -2.665086 -0.275852 0.124988

C -3.468702 0.870547 0.095424

C -2.885299 2.127415 -0.018085

C -1.499291 2.219743 -0.076609

C 0.740747 1.047393 -0.033986

C 2.665087 -0.275853 0.124991

C 3.468702 0.870546 0.095416

C 2.885300 2.127412 -0.018106

C 1.499293 2.219741 -0.076628

H -4.545882 0.762743 0.161622

H -3.498658 3.022721 -0.049768

H -1.020915 3.188350 -0.149916

H 4.545883 0.762741 0.161615

H 3.498660 3.022718 -0.049800

H 1.020917 3.188347 -0.149946

C 3.289830 -1.635987 0.256799

H 2.830927 -2.192280 1.077537

H 3.115367 -2.227334 -0.645914

H 4.365353 -1.547605 0.432954

C -3.289828 -1.635989 0.256776

H -3.115379 -2.227317 -0.645953

H -2.830915 -2.192299 1.077495

H -4.365349 -1.547609 0.432946

N 1.317016 -0.181791 0.041905

N -1.317016 -0.181790 0.041900

Ni -0.000001 -1.649807 -0.193893

H -0.885906 -2.756274 -0.496244

H 0.885900 -2.756280 -0.496234

$^{3}LNi^{II}H_2$

B3LYP/BSI SCF energy: -746.169593a.u.

M06/BSII SCF energy in solution: -745.885539a.u.

M06/BSII free energy in solution: -745.701998a.u.

C -0.740205 -1.077062 0.000098

C	-2.662830	0.263478	0.000441
C	-3.480678	-0.869251	0.000476
C	-2.897572	-2.134878	0.000306
C	-1.511645	-2.245010	0.000112
C	0.740205	-1.077062	-0.000100
C	2.662830	0.263478	-0.000441
C	3.480678	-0.869251	-0.000478
C	2.897572	-2.134878	-0.000310
C	1.511645	-2.245010	-0.000116
H	-4.559001	-0.751090	0.000627
H	-3.517126	-3.026498	0.000317
H	-1.042684	-3.221305	-0.000041
H	4.559001	-0.751089	-0.000628
H	3.517126	-3.026498	-0.000323
H	1.042684	-3.221305	0.000035
C	3.213942	1.659389	-0.000593
H	2.855503	2.203314	-0.881964
H	2.855686	2.203424	0.880784
H	4.306306	1.659325	-0.000709
C	-3.213942	1.659389	0.000596
H	-2.855504	2.203312	0.881968
H	-2.855685	2.203426	-0.880780
H	-4.306306	1.659325	0.000711
N	1.320863	0.143890	-0.000253
N	-1.320863	0.143890	0.000253
Ni	0.000000	1.732848	0.000002
H	0.000416	2.085736	1.558806
H	-0.000416	2.085743	-1.558801

[1]TS3-A

B3LYP/BSI SCF energy: -1507.381884a.u.

M06/BSII SCF energy in solution: -1506.9118a.u.

M06/BSII free energy in solution: -1506.571113a.u.

C	-2.552854	1.802936	-0.270525
C	-0.784301	2.997576	0.686404
C	-1.366998	4.208704	0.300754
C	-2.576455	4.209422	-0.384031
C	-3.175222	2.988631	-0.672690
C	-3.192615	0.492313	-0.573484
C	-3.744913	-1.621036	0.188107
C	-4.424048	-1.907478	-1.005230
C	-4.461233	-0.947190	-2.011737
C	-3.842001	0.281890	-1.797358
H	-0.862979	5.137280	0.545848
H	-3.047186	5.141828	-0.680684
H	-4.130435	2.941535	-1.182276
H	-4.907819	-2.870290	-1.136702
H	-4.966121	-1.151762	-2.951478
H	-3.841283	1.048173	-2.564832
C	-3.660154	-2.623413	1.309599
H	-2.668558	-3.086224	1.320318
H	-3.800626	-2.125053	2.271960
H	-4.406709	-3.414628	1.199660
C	0.525842	2.971227	1.418386
H	0.414920	2.426054	2.361352
H	1.284905	2.439951	0.834109
H	0.881964	3.985136	1.619220
N	-3.134787	-0.442897	0.384735
N	-1.378010	1.810961	0.406427
Ni	-0.489703	0.166380	0.964802
C	0.429547	-1.289272	1.887327
H	1.074124	-0.929384	2.680109
H	-0.278105	-2.057830	2.181169

C	0.893962	-1.231442	0.540616
C	0.509472	-2.395478	-0.355294
C	2.281861	-0.705825	0.253867
C	2.572188	0.092477	-0.858319
C	3.352318	-1.071880	1.089061
C	3.870468	0.528389	-1.131205
H	1.765735	0.390158	-1.520408
C	4.649051	-0.644180	0.835271
H	3.164937	-1.711741	1.945616
C	4.919209	0.161653	-0.279298
H	4.047872	1.150354	-2.000828
H	5.472205	-0.928299	1.482835
O	6.222281	0.534285	-0.444077
C	6.553128	1.335299	-1.566436
H	7.629604	1.504926	-1.509894
H	6.317261	0.828218	-2.511028
H	6.034671	2.302815	-1.541909
F	1.330509	-3.450880	-0.126269
F	-0.754542	-2.834891	-0.135580
F	0.603192	-2.115762	-1.673425
H	-0.092674	-0.128433	-0.445063
H	-0.832634	0.411712	2.397689

^{3}TS3-A

B3LYP/BSI SCF energy: -1507.349478a.u.

M06/BSII SCF energy in solution: -1506.870951a.u.

M06/BSII free energy in solution: -1506.535325a.u.

C	-2.300632	2.004592	-0.309592
C	-0.364053	2.879439	0.672200
C	-0.578600	4.114848	0.051610
C	-1.674607	4.273908	-0.791650
C	-2.551831	3.210003	-0.975712
C	-3.212878	0.834422	-0.376788
C	-3.631188	-1.346821	0.356639
C	-4.883522	-1.338804	-0.270123
C	-5.289540	-0.215843	-0.980711
C	-4.450693	0.892513	-1.024992
H	0.112213	4.932031	0.230638
H	-1.850171	5.219924	-1.294956
H	-3.411792	3.322709	-1.624049
H	-5.524009	-2.210437	-0.188255
H	-6.255173	-0.193455	-1.476706
H	-4.766716	1.791175	-1.539378
C	-3.171887	-2.534771	1.151436
H	-2.448401	-3.118864	0.576991
H	-2.674438	-2.200295	2.066525
H	-4.015601	-3.181874	1.404178
C	0.813636	2.631972	1.572391
H	0.494543	2.087693	2.466568
H	1.559920	2.012098	1.062386
H	1.290592	3.570512	1.866058
N	-2.806887	-0.282172	0.275125
N	-1.212567	1.853250	0.473840
Ni	-0.887452	-0.134700	1.137076
C	0.503951	-2.285202	1.443385
H	0.967559	-2.000072	2.376965
H	-0.262469	-3.046364	1.475480
C	0.876410	-1.676194	0.243837
C	0.516407	-2.454404	-1.019229
C	2.223568	-0.988217	0.158918
C	2.483573	0.052092	-0.741395
C	3.287697	-1.432744	0.963041
C	3.745787	0.642218	-0.838643

H	1.681248	0.413652	-1.375080
C	4.546311	-0.850290	0.886850
H	3.122310	-2.259134	1.645557
C	4.787237	0.192782	-0.017355
H	3.901605	1.444090	-1.550891
H	5.363815	-1.195628	1.511405
O	6.055853	0.697557	-0.022381
C	6.362624	1.732903	-0.940961
H	7.416312	1.971320	-0.786841
H	6.214178	1.411405	-1.980023
H	5.760282	2.632304	-0.756481
F	1.368018	-3.489889	-1.192854
F	-0.734035	-2.981672	-0.961942
F	0.570197	-1.717956	-2.150417
H	-0.043247	-0.425770	-0.241948
H	-1.158789	-0.108198	2.698000

^{1}IM2-A

B3LYP/BSI SCF energy: -1507.425572a.u.

M06/BSII SCF energy in solution: -1506.953547a.u.

M06/BSII free energy in solution: -1506.607084a.u.

C	2.615243	0.994719	0.480628
C	2.704900	2.034242	-1.615871
C	3.231559	3.191338	-1.030703
C	3.414637	3.254509	0.346487
C	3.114306	2.134709	1.114574
C	2.379289	-0.285390	1.188726
C	1.822035	-2.539361	0.926141
C	2.168233	-2.795146	2.259926
C	2.594844	-1.754548	3.076027
C	2.726528	-0.483013	2.527976
H	3.489924	4.033119	-1.664164
H	3.803486	4.154188	0.813314
H	3.270014	2.151291	2.186073
H	2.103613	-3.809869	2.638052
H	2.854263	-1.935485	4.114719
H	3.110822	0.331747	3.128563
C	1.411864	-3.675206	0.028582
H	0.326884	-3.812107	0.017882
H	1.732212	-3.487965	-0.997386
H	1.862395	-4.607516	0.380318
C	2.506372	1.956604	-3.103040
H	2.833938	0.987495	-3.485732
H	1.444806	2.046245	-3.352267
H	3.060984	2.754170	-3.605267
N	1.877498	-1.287736	0.420034
N	2.370423	0.963315	-0.857400
Ni	1.069380	-0.452225	-1.289993
C	-0.537888	-1.498529	-1.550863
H	-1.114025	-1.183739	-2.423801
H	-0.248448	-2.538094	-1.741935
C	-1.459487	-1.438596	-0.311323
C	-2.570176	-2.485217	-0.388189
C	-2.048357	-0.060319	-0.041227
C	-1.907272	0.540814	1.210651
C	-2.758059	0.646693	-1.026793
C	-2.432713	1.807597	1.489284
H	-1.373885	0.012423	1.997660
C	-3.291183	1.902347	-0.769147
H	-2.897616	0.205179	-2.007568
C	-3.130577	2.494660	0.492119
H	-2.296531	2.235440	2.475808
H	-3.840383	2.447286	-1.530396

O	-3.690961	3.734509	0.643882
C	-3.572406	4.368490	1.904052
H	-4.085927	5.327279	1.811618
H	-4.047450	3.782226	2.702054
H	-2.522668	4.548125	2.173437
F	-3.363790	-2.340342	-1.472785
F	-2.053957	-3.746916	-0.449188
F	-3.377265	-2.463170	0.700210
H	-0.899340	-1.731034	0.585621
H	0.547480	0.232275	-2.438559

[1]TS4-A

B3LYP/BSI SCF energy: -1507.404713a.u.

M06/BSII SCF energy in solution: -1506.931353a.u.

M06/BSII free energy in solution: -1506.587178a.u.

C	-3.409732	1.629411	0.085003
C	-1.455069	2.752824	-0.517124
C	-2.071378	3.988085	-0.299062
C	-3.388803	4.032643	0.152748
C	-4.071854	2.835100	0.335627
C	-4.072957	0.315577	0.197014
C	-3.746214	-2.001046	0.286000
C	-5.126372	-2.210498	0.266412
C	-5.997520	-1.122039	0.218930
C	-5.462321	0.159779	0.176686
H	-1.517196	4.901866	-0.488514
H	-3.876889	4.982688	0.347413
H	-5.098112	2.834932	0.684265
H	-5.508923	-3.225421	0.292191
H	-7.072424	-1.273085	0.199521
H	-6.110531	1.024768	0.098958
C	-2.783238	-3.152105	0.345639
H	-2.091510	-3.127280	-0.500662
H	-2.167492	-3.078660	1.248671
H	-3.313883	-4.108201	0.345020
C	-0.042300	2.679742	-1.025931
H	0.665837	2.471027	-0.214960
H	0.050679	1.873628	-1.758292
H	0.255146	3.622453	-1.492831
N	-3.226181	-0.746770	0.271445
N	-2.099843	1.582863	-0.291507
Ni	-1.348302	-0.240809	0.331940
C	0.639972	-0.321425	0.838739
H	0.628493	0.757513	1.037370
H	1.104259	-0.753022	1.733887
C	1.565903	-0.626957	-0.363369
C	1.394696	-2.061601	-0.857280
C	3.024425	-0.308041	-0.061014
C	3.674076	0.726219	-0.737220
C	3.749813	-1.002468	0.923919
C	4.997123	1.079382	-0.453076
H	3.142248	1.276437	-1.509914
C	5.065125	-0.669451	1.215844
H	3.283262	-1.823294	1.459279
C	5.700003	0.377965	0.531120
H	5.460528	1.889950	-1.003247
H	5.626980	-1.208737	1.971692
O	6.992337	0.629897	0.895782
C	7.682103	1.674911	0.231476
H	8.681374	1.704148	0.669010
H	7.766689	1.485152	-0.846647
H	7.193345	2.646130	0.385034
F	1.504403	-2.972596	0.141916

F 0.178530 -2.255541 -1.430051

F 2.312292 -2.385598 -1.793432

H 1.258568 -0.016863 -1.218107

H -0.414417 -1.137721 1.028343

2b

B3LYP/BSI SCF energy: -762.446037a.u.

M06/BSII SCF energy in solution: -762.239307a.u.

M06/BSII free energy in solution: -762.084971a.u.

C -2.160945 0.118903 -1.978714

H -2.077204 1.208596 -1.961115

H -3.208860 -0.141224 -2.150244

C -1.649645 -0.496615 -0.663153

C -2.539169 -0.069836 0.500391

C -0.178850 -0.211960 -0.401670

C 0.739253 -1.261585 -0.340462

C 0.310073 1.097296 -0.248057

C 2.104092 -1.037804 -0.136467

H 0.390154 -2.285073 -0.454469

C 1.660936 1.338517 -0.040987

H -0.375832 1.937257 -0.277113

C 2.570996 0.271674 0.015054

H 2.781166 -1.882679 -0.096659

H 2.039326 2.348000 0.082634

O 3.875508 0.615247 0.218863

C 4.839146 -0.423126 0.293543

H 5.798878 0.067437 0.462979

H 4.632689 -1.108511 1.125758

H 4.888737 -0.998663 -0.640009

F -2.577635 1.276646 0.655988

F -3.817377 -0.476428 0.306262

F -2.127505 -0.598235 1.670197

H -1.779306 -1.583761 -0.715444

H -1.566485 -0.253990 -2.816116

^{1}LNi^0

B3LYP/BSI SCF energy: -744.979533a.u.

M06/BSII SCF energy in solution: -744.705227a.u.

M06/BSII free energy in solution: -744.534652a.u.

C -0.719655 0.991086 -0.000041

C -2.706569 -0.332375 0.000096

C -3.506564 0.794999 0.000002

C -2.889404 2.071510 -0.000171

C -1.516805 2.163545 -0.000207

C 0.719656 0.991085 0.000042

C 2.706569 -0.332376 -0.000071

C 3.506565 0.794998 0.000017

C 2.889405 2.071509 0.000168

C 1.516806 2.163544 0.000194

H -4.586019 0.694223 0.000059

H -3.497404 2.971166 -0.000347

H -1.040681 3.137267 -0.000443

H 4.586019 0.694221 -0.000028

H 3.497405 2.971165 0.000333

H 1.040682 3.137266 0.000411

C 3.283314 -1.723305 -0.000131

H 2.951111 -2.279804 0.885621

H 2.951098 -2.279743 -0.885920

H 4.375644 -1.705578 -0.000139

C -3.283313 -1.723304 0.000176

H -2.951097 -2.279821 -0.885561

H -2.951109 -2.279724 0.885979

H	-4.375643	-1.705578	0.000168
N	1.352585	-0.249778	-0.000037
N	-1.352584	-0.249777	0.000050
Ni	-0.000002	-1.612988	-0.000024

1c˙

B3LYP/BSI SCF energy: -518.248831a.u.

M06/BSII SCF energy in solution: -518.012053a.u.

M06/BSII free energy in solution: -517.852849a.u.

C	2.493539	-0.544160	-0.316352
C	1.232566	0.175272	-0.196782
N	0.113139	-0.649149	-0.151886
C	-1.242938	-0.299142	-0.045123
C	-1.695298	1.029351	0.037608
C	-2.179882	-1.348084	-0.021967
C	-3.063490	1.279652	0.140934
H	-0.974892	1.834252	0.019431
C	-3.540813	-1.079105	0.081530
H	-1.834699	-2.378162	-0.085767
C	-3.993813	0.239574	0.164041
H	-3.402420	2.310003	0.204164
H	-4.247751	-1.903738	0.097569
H	-5.055530	0.451453	0.244975
C	3.793233	0.176173	-0.380758
H	4.252114	0.003738	-1.367781
H	3.594436	1.248841	-0.304267
O	1.171716	1.408153	-0.141151
C	4.792370	-0.272849	0.704536
H	5.000324	-1.346488	0.638748
H	5.742118	0.259676	0.593995
H	4.402293	-0.066277	1.705680
H	2.492941	-1.633044	-0.354290
H	0.292526	-1.641525	-0.206042

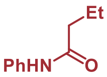

B3LYP/BSI SCF energy: -518.904666a.u.

M06/BSII SCF energy in solution: -518.667159a.u.

M06/BSII free energy in solution: -518.492941a.u.

C	1.283818	0.061880	-0.452167
N	0.162500	-0.709580	-0.236529
O	1.273152	1.284861	-0.501948
C	-1.181441	-0.307495	-0.076371
C	-2.137135	-1.319620	0.109840
C	-1.591956	1.034779	-0.095269
C	-3.482084	-1.000181	0.273624
H	-1.822043	-2.361140	0.125436
C	-2.944038	1.337028	0.070039
H	-0.855099	1.812123	-0.237495
C	-3.895622	0.333222	0.254550
H	-4.206217	-1.797244	0.416027
H	-3.252411	2.378699	0.053531
H	-4.944005	0.584847	0.382200
C	3.818794	0.003068	-0.203456
H	3.818681	0.988348	-0.679938
H	4.702006	-0.533770	-0.569350
H	0.312783	-1.707882	-0.208807
C	3.901486	0.166904	1.317176
H	4.816494	0.693888	1.605609
H	3.052879	0.745510	1.694818
H	3.904490	-0.805080	1.825055
C	2.565039	-0.748434	-0.667923

H	2.633721	-0.951965	-1.745471
H	2.494840	-1.725065	-0.170585

1d

B3LYP/BSI SCF energy: -558.218984a.u.

M06/BSII SCF energy in solution: -557.958025a.u.

M06/BSII free energy in solution: -557.757245a.u.

C	1.057325	0.180021	0.407730
N	-0.053401	-0.631602	0.313455
O	1.011764	1.403653	0.362071
C	-1.407541	-0.278271	0.127247
C	-2.341042	-1.325397	0.058283
C	-1.849426	1.049172	0.011276
C	-3.694185	-1.055063	-0.124595
H	-2.002121	-2.355567	0.148298
C	-3.209303	1.302106	-0.171972
H	-1.129983	1.853361	0.067001
C	-4.138539	0.263412	-0.241571
H	-4.400652	-1.878638	-0.175543
H	-3.541766	2.332650	-0.260817
H	-5.193454	0.476711	-0.384330
C	2.974898	-0.849078	-0.883072
H	2.246016	-1.427836	-1.465533
H	3.850467	-1.498920	-0.757153
C	2.381666	-0.586566	0.529460
H	2.174834	-1.570630	0.974057
C	3.345768	0.169164	1.451437
H	4.326526	-0.317371	1.464396
H	3.465394	1.201719	1.117966
H	2.965983	0.196826	2.477436
H	0.119949	-1.624287	0.380570
C	3.373615	0.403362	-1.671772
H	3.722115	0.125757	-2.671793
H	2.532166	1.092692	-1.780587
H	4.184792	0.948344	-1.179433

1d˙

B3LYP/BSI SCF energy: -557.568719a.u.

M06/BSII SCF energy in solution: -557.309686a.u.

M06/BSII free energy in solution: -557.123727a.u.

C	1.085032	-0.758081	-0.080969
N	0.121525	0.238126	-0.190347
O	0.795535	-1.940503	0.138602
C	-1.277027	0.131915	-0.087986
C	-2.022028	1.315537	-0.239544
C	-1.952172	-1.076644	0.154951
C	-3.410253	1.296399	-0.150693
H	-1.505550	2.254839	-0.428030
C	-3.344554	-1.076943	0.240560
H	-1.380589	-1.985562	0.271937
C	-4.083878	0.097003	0.090583
H	-3.965122	2.222596	-0.270926
H	-3.855288	-2.017393	0.428553
H	-5.167211	0.079399	0.160114
C	3.543606	-1.355682	-0.086689
H	3.110479	-2.319571	0.180532
H	4.116846	-1.469869	-1.018633
H	4.270461	-1.060113	0.683644
C	2.477211	-0.322209	-0.243127
C	2.891674	1.103048	-0.494568
H	3.865172	1.099530	-0.999023
H	2.204799	1.601514	-1.192945

H 0.456389 1.173138 -0.363577
C 3.009957 1.946159 0.797040
H 3.344417 2.962521 0.565680
H 2.051854 2.012562 1.322229
H 3.732988 1.500530 1.486706

^{1}IM4^{1c}-*R*

B3LYP/BSI SCF energy: -2700.356321a.u.

M06/BSII SCF energy in solution: -2699.148904a.u.

M06/BSII free energy in solution: -2698.327217a.u.

C	1.518533	-0.906995	-1.164047
N	1.595193	-0.486593	0.044368
O	2.676894	-1.120254	-1.815703
C	3.749737	-1.086186	-0.785132
C	3.036461	-0.259074	0.345191
H	3.248840	-0.685399	1.322839
C	3.335347	1.229948	0.419585
C	3.866361	1.756290	1.603944
C	3.084489	2.097851	-0.652600
C	4.160668	3.117427	1.710179
H	4.031082	1.097898	2.451683
C	3.382257	3.456272	-0.550872
H	2.667993	1.713200	-1.578291
C	3.925199	3.969907	0.630736
H	4.571133	3.508609	2.636513
H	3.192133	4.113625	-1.394321
H	4.160482	5.027633	0.708022
C	-1.010335	-0.986404	-1.235602
N	-1.250351	-0.462487	-0.082007
O	-2.072189	-1.394543	-1.949263
C	-3.270188	-0.776915	-1.327253
C	-2.723812	-0.562046	0.137594
H	-3.073527	0.381618	0.556631
C	-3.070398	-1.677383	1.115861
C	-4.265730	-1.582597	1.842637
C	-2.257668	-2.800260	1.305042
C	-4.645961	-2.591076	2.725631
H	-4.898376	-0.708283	1.718407
C	-2.632209	-3.807892	2.196954
H	-1.315071	-2.887918	0.775416
C	-3.828433	-3.708926	2.906233
H	-5.574745	-2.498286	3.281048
H	-1.981666	-4.665717	2.340404
H	-4.117619	-4.490972	3.602264
Ni	0.091785	-0.046885	1.388154
C	0.289362	-1.201235	-1.985879
C	0.357328	-2.678392	-2.464653
H	1.277256	-2.834501	-3.029738
H	0.354674	-3.368133	-1.615690
H	-0.499460	-2.900237	-3.102010
C	0.288119	-0.249058	-3.218004
H	-0.590796	-0.447059	-3.833288
H	0.260572	0.797992	-2.903454
H	1.188251	-0.418598	-3.812052
C	-3.510512	0.498639	-2.146691
C	-3.804090	0.331667	-3.513147
C	-3.402306	1.795179	-1.634928
C	-3.980257	1.432014	-4.345787
H	-3.897092	-0.670972	-3.919617
C	-3.582723	2.900864	-2.475920
H	-3.182422	1.977937	-0.588597
C	-3.869319	2.726110	-3.826875
H	-4.208231	1.281197	-5.397304

H	-3.484289	3.898241	-2.058310
H	-4.007338	3.587849	-4.473901
C	-4.434694	-1.747246	-1.452298
C	-5.743374	-1.266003	-1.307416
C	-4.236958	-3.113286	-1.681233
C	-6.830594	-2.135510	-1.379559
H	-5.911789	-0.205559	-1.146372
C	-5.327460	-3.980703	-1.765711
H	-3.230625	-3.498083	-1.796882
C	-6.626701	-3.496890	-1.612376
H	-7.837705	-1.745962	-1.262480
H	-5.157502	-5.037558	-1.950517
H	-7.473766	-4.173624	-1.676899
C	4.987567	-0.437318	-1.379712
C	6.128848	-0.309613	-0.574577
C	5.034358	0.019223	-2.700225
C	7.287019	0.278536	-1.077001
H	6.112045	-0.677573	0.447284
C	6.200077	0.602175	-3.205056
H	4.161991	-0.083987	-3.334839
C	7.327036	0.737432	-2.396293
H	8.160455	0.374938	-0.438814
H	6.222385	0.949318	-4.234170
H	8.231569	1.192335	-2.789154
C	3.997508	-2.557254	-0.414624
C	4.748978	-3.351904	-1.296490
C	3.434328	-3.161327	0.719228
C	4.940379	-4.709268	-1.050189
H	5.190612	-2.900840	-2.179132
C	3.633179	-4.523298	0.966079
H	2.837588	-2.596317	1.428149
C	4.384523	-5.300713	0.086934
H	5.528789	-5.303507	-1.743528
H	3.195699	-4.967843	1.855205
H	4.539325	-6.357507	0.285047
C	-1.566989	2.279532	1.813509
N	-0.718192	3.305482	1.414665
O	-2.770293	2.287163	1.506536
C	-1.017911	4.500425	0.736723
C	0.063052	5.287410	0.300647
C	-2.328798	4.959725	0.513985
C	-0.157539	6.498430	-0.349422
H	1.078400	4.939624	0.472745
C	-2.531316	6.175604	-0.140825
H	-3.161526	4.365610	0.861466
C	-1.457880	6.952246	-0.580048
H	0.693667	7.089456	-0.676708
H	-3.549773	6.521459	-0.297896
H	-1.630472	7.896884	-1.086862
C	-1.860618	0.562137	3.632095
H	-2.789901	0.264532	3.141470
H	-1.395620	-0.346579	4.022761
Br	1.419536	-0.894291	3.293503
C	-0.920992	1.202295	2.607974
H	-0.035558	1.598864	3.119796
C	-2.199099	1.500547	4.803419
H	-2.864679	1.007584	5.521497
H	-1.293198	1.800762	5.342751
H	-2.702109	2.408067	4.451577
H	0.249215	3.196314	1.686749

^{1}IM4^{1c}-*S*

B3LYP/BSI SCF energy: -2700.363449a.u.

M06/BSII SCF energy in solution: -2699.152511a.u.

M06/BSII free energy in solution: -2698.333009a.u.

C	0.367690	-1.974771	0.122547
N	0.855924	-0.787750	0.136385
O	1.253155	-2.989027	0.108410
C	2.586664	-2.408503	0.394138
C	2.336331	-0.899508	0.028258
C	-2.075947	-1.312294	-0.042131
N	-1.878025	-0.046192	-0.003867
O	-3.344317	-1.725900	-0.228510
C	-4.121027	-0.524629	-0.645777
C	-3.201500	0.623416	-0.087206
Ni	-0.077799	0.985309	0.075918
C	-1.077015	-2.439267	0.131655
C	-1.276282	-3.470036	-1.012534
H	-0.593986	-4.310023	-0.876177
H	-1.086638	-3.015922	-1.989532
H	-2.303680	-3.835367	-1.000864
C	-1.355060	-3.117746	1.505170
H	-2.375091	-3.508059	1.523130
H	-1.231879	-2.405124	2.325186
H	-0.655833	-3.942527	1.654940
C	-5.508821	-0.597153	-0.033721
C	-6.415761	0.437824	-0.304509
C	-5.915706	-1.660412	0.777664
C	-7.697550	0.415811	0.239753
H	-6.118366	1.261128	-0.947541
C	-7.204769	-1.684282	1.317356
H	-5.226926	-2.470995	0.984822
C	-8.097528	-0.646973	1.054125
H	-8.385859	1.227951	0.025046
H	-7.507162	-2.518474	1.944060
H	-9.098231	-0.665442	1.475651
C	-4.161349	-0.585190	-2.181068
C	-3.254686	0.115905	-2.991011
C	-5.085026	-1.444972	-2.797791
C	-3.283934	-0.034012	-4.380574
H	-2.516837	0.788544	-2.566749
C	-5.109610	-1.595114	-4.182496
H	-5.792838	-1.995693	-2.187151
C	-4.208976	-0.886370	-4.980923
H	-2.578496	0.525728	-4.987533
H	-5.836143	-2.262170	-4.637704
H	-4.231528	-0.996403	-6.061350
C	2.846938	-2.668216	1.887550
C	3.277055	-1.686958	2.788853
C	2.674512	-3.984164	2.350846
C	3.521130	-2.021846	4.126642
H	3.414216	-0.653057	2.488874
C	2.910304	-4.310068	3.682920
H	2.358512	-4.755876	1.655664
C	3.338167	-3.325751	4.578904
H	3.850718	-1.246385	4.811783
H	2.768999	-5.332911	4.020708
H	3.527909	-3.577321	5.618440
C	3.624708	-3.128792	-0.455934
C	3.273335	-4.085944	-1.412667
C	4.980073	-2.825543	-0.263676
C	4.260071	-4.726657	-2.166431
H	2.229955	-4.334005	-1.567804
C	5.961357	-3.457881	-1.023409
H	5.268976	-2.097825	0.488714
C	5.605064	-4.413866	-1.977741
H	3.971362	-5.471311	-2.902894

H	7.006177	-3.207075	-0.865428
H	6.370866	-4.911109	-2.565805
C	2.158494	2.266947	1.411072
N	2.340301	3.379377	0.608873
O	3.120842	1.606593	1.847452
C	3.536335	3.937622	0.130204
C	3.444330	4.836436	-0.948527
C	4.797637	3.672926	0.693793
C	4.584925	5.450891	-1.456467
H	2.471617	5.040273	-1.389077
C	5.931347	4.294844	0.168894
H	4.871372	2.985001	1.523926
C	5.840089	5.183418	-0.903674
H	4.490271	6.140224	-2.291068
H	6.899652	4.081499	0.614337
H	6.730260	5.662310	-1.300704
C	0.507508	1.076916	2.929374
H	1.114384	0.167634	2.882111
H	-0.542567	0.758845	2.949909
H	2.773660	-0.243387	0.776956
C	2.807319	-0.419140	-1.335050
C	3.814214	0.551387	-1.395054
C	2.253119	-0.897900	-2.530430
C	4.266586	1.030348	-2.626226
H	4.223365	0.953539	-0.473318
C	2.706062	-0.422813	-3.759824
H	1.463866	-1.643781	-2.506718
C	3.715509	0.542304	-3.810613
H	5.035787	1.796197	-2.651924
H	2.268548	-0.802508	-4.678828
H	4.061441	0.917364	-4.769585
H	-3.126813	1.436138	-0.807755
C	-3.568156	1.235510	1.255039
C	-3.822403	2.609794	1.328020
C	-3.632993	0.472900	2.429781
C	-4.147833	3.210453	2.546029
H	-3.743421	3.215564	0.429941
C	-3.957323	1.070579	3.646549
H	-3.438152	-0.594632	2.396670
C	-4.217318	2.442147	3.707785
H	-4.337601	4.279112	2.584858
H	-4.007064	0.466080	4.547703
H	-4.466994	2.907354	4.657004
Br	-0.689878	2.962827	-1.250450
C	0.824317	1.818549	4.241301
H	0.214251	2.723733	4.338525
H	0.622731	1.183987	5.112412
H	1.877631	2.112254	4.271270
H	1.495281	3.716933	0.157905
C	0.739947	1.924823	1.683102
H	0.112195	2.821152	1.685294

^{1}**TS11^{1c}**-*R*

B3LYP/BSI SCF energy: -2700.337946a.u.

M06/BSII SCF energy in solution: -2699.129349a.u.

M06/BSII free energy in solution: -5893.833697a.u.

C	1.671002	-0.943174	-1.169753
N	1.683522	-0.417257	-0.001834
O	2.868211	-1.151072	-1.761150
C	3.893633	-0.974704	-0.698618
C	3.091295	-0.106565	0.344294
H	3.274320	-0.463107	1.356820
C	3.325467	1.395059	0.341803

C	3.820712	2.007910	1.499304
C	3.043277	2.192121	-0.775914
C	4.045944	3.385721	1.536682
H	4.015189	1.403590	2.380879
C	3.269796	3.567474	-0.742768
H	2.652819	1.738494	-1.681498
C	3.775406	4.168808	0.413742
H	4.429993	3.844261	2.443388
H	3.053334	4.169888	-1.620360
H	3.955677	5.239880	0.437712
C	-0.859124	-1.139847	-1.327608
N	-1.142067	-0.694766	-0.153592
O	-1.899959	-1.460242	-2.122064
C	-3.112882	-0.874575	-1.509301
C	-2.622093	-0.697671	-0.014526
H	-2.931811	0.272585	0.373133
C	-3.095354	-1.753406	0.973728
C	-4.278173	-1.522235	1.689095
C	-2.401086	-2.949656	1.187540
C	-4.764383	-2.471333	2.587306
H	-4.813901	-0.587575	1.547159
C	-2.881603	-3.896802	2.093918
H	-1.470633	-3.138349	0.662266
C	-4.066008	-3.662786	2.792889
H	-5.682051	-2.274839	3.134258
H	-2.324696	-4.815118	2.256109
H	-4.437825	-4.399251	3.499397
Ni	0.167931	-0.441498	1.314860
C	0.483375	-1.386371	-1.995959
C	0.617110	-2.910645	-2.274950
H	1.563459	-3.109327	-2.780721
H	0.599084	-3.482108	-1.342611
H	-0.206110	-3.243308	-2.910196
C	0.512222	-0.602471	-3.338969
H	-0.311284	-0.927288	-3.975890
H	0.408240	0.472783	-3.166638
H	1.458122	-0.787729	-3.851548
C	-3.350720	0.433186	-2.279564
C	-3.518744	0.322527	-3.672439
C	-3.388005	1.704161	-1.697682
C	-3.709154	1.450549	-4.463122
H	-3.501082	-0.660651	-4.132742
C	-3.581613	2.838388	-2.496852
H	-3.278077	1.851922	-0.628443
C	-3.740020	2.718597	-3.874313
H	-3.836755	1.341374	-5.536528
H	-3.593147	3.815687	-2.025298
H	-3.887901	3.603047	-4.487569
C	-4.268165	-1.847090	-1.703516
C	-5.584696	-1.383016	-1.575614
C	-4.052480	-3.201234	-1.981313
C	-6.661473	-2.257632	-1.712057
H	-5.767543	-0.331186	-1.378287
C	-5.132199	-4.073776	-2.129661
H	-3.039977	-3.571845	-2.089693
C	-6.439369	-3.607126	-1.992612
H	-7.674899	-1.881038	-1.607529
H	-4.947566	-5.120809	-2.352714
H	-7.278268	-4.287431	-2.107136
C	5.114340	-0.297568	-1.297523
C	6.225578	-0.065550	-0.473691
C	5.172077	0.093747	-2.638411
C	7.363907	0.558296	-0.978573
H	6.201203	-0.380799	0.565483

C 6.317962 0.712941 -3.145456
H 4.323297 -0.089289 -3.286782
C 7.414750 0.950269 -2.318856
H 8.213734 0.734694 -0.325678
H 6.348663 1.008032 -4.190533
H 8.303966 1.432959 -2.713656
C 4.208162 -2.394153 -0.198801
C 5.071559 -3.199621 -0.959850
C 3.599401 -2.951232 0.936135
C 5.329104 -4.518819 -0.593995
H 5.548980 -2.786676 -1.842461
C 3.863163 -4.274344 1.303096
H 2.916760 -2.379086 1.556072
C 4.727014 -5.061381 0.543740
H 6.004635 -5.120938 -1.195080
H 3.387386 -4.680106 2.191049
H 4.932644 -6.087463 0.835212
C -1.983602 2.414242 2.085696
N -1.082449 3.362915 1.613158
O -3.150477 2.331126 1.659245
C -1.321965 4.465606 0.778947
C -0.206159 5.153305 0.268941
C -2.613535 4.939355 0.484663
C -0.376369 6.288814 -0.517673
H 0.794457 4.789865 0.489564
C -2.764346 6.080494 -0.303881
H -3.472485 4.412227 0.875617
C -1.656973 6.762221 -0.812127
H 0.498762 6.805720 -0.901770
H -3.767168 6.442520 -0.515087
H -1.788904 7.649039 -1.424514
C -2.339313 0.678049 3.941926
H -3.274064 0.507747 3.403140
H -1.845309 -0.288603 4.091669
Br 1.228160 -0.734356 3.449918
C -1.453275 1.557606 3.129598
H -0.430299 1.700283 3.464795
C -2.644752 1.291242 5.328636
H -3.278624 0.613212 5.909657
H -1.724455 1.461518 5.896575
H -3.168532 2.247537 5.230452
H -0.126034 3.249318 1.920595

^{1}**TS11**^{1c}-*S*

B3LYP/BSI SCF energy: -2700.345284a.u.

M06/BSII SCF energy in solution: -2699.135386a.u.

M06/BSII free energy in solution: -2698.320622a.u.

C -0.184942 2.021633 -0.058788
N -0.708493 0.857284 -0.184910
O -1.026597 3.041285 0.211036
C -2.348676 2.465914 0.521583
C -2.179118 0.986633 -0.023657
C 2.233911 1.287834 -0.247085
N 1.983290 0.031634 -0.202595
O 3.534576 1.648018 -0.330757
C 4.288456 0.407556 -0.653575
C 3.278076 -0.689795 -0.149584
Ni 0.225118 -0.845931 -0.536201
C 1.261359 2.450678 -0.242624
C 1.374931 3.183060 -1.611702
H 0.710418 4.050154 -1.624071
H 1.104204 2.516273 -2.434839
H 2.401748 3.521705 -1.763355

C	1.661127	3.420072	0.901843
H	2.696041	3.739555	0.768213
H	1.564327	2.937786	1.878951
H	1.013519	4.297516	0.888113
C	5.621692	0.434040	0.072688
C	6.513406	-0.630985	-0.121697
C	5.991332	1.480920	0.922236
C	7.743468	-0.653029	0.531293
H	6.245558	-1.442957	-0.791691
C	7.228931	1.460657	1.571295
H	5.313192	2.312362	1.073975
C	8.106726	0.394837	1.381358
H	8.420206	-1.487662	0.373434
H	7.503199	2.282934	2.226097
H	9.067349	0.379468	1.887752
C	4.458665	0.422321	-2.181285
C	3.590995	-0.258905	-3.048867
C	5.469955	1.221241	-2.739437
C	3.743403	-0.151868	-4.434141
H	2.784544	-0.878113	-2.671209
C	5.618151	1.329435	-4.120438
H	6.149288	1.757769	-2.085187
C	4.755637	0.639087	-4.974919
H	3.064693	-0.695081	-5.085034
H	6.411446	1.949349	-4.528368
H	4.874186	0.715981	-6.051889
C	-2.521840	2.548340	2.046163
C	-3.176421	1.559654	2.791221
C	-2.074546	3.704794	2.705467
C	-3.368449	1.724957	4.167198
H	-3.537018	0.641801	2.337913
C	-2.259106	3.861259	4.076929
H	-1.580566	4.484970	2.136326
C	-2.910092	2.869623	4.815365
H	-3.876354	0.943526	4.724740
H	-1.900499	4.761510	4.568194
H	-3.058862	2.991637	5.884418
C	-3.410827	3.311109	-0.178932
C	-3.069701	4.307219	-1.099736
C	-4.765524	3.088590	0.105021
C	-4.062471	5.063366	-1.727231
H	-2.026744	4.497558	-1.322307
C	-5.755019	3.836300	-0.529609
H	-5.049692	2.332852	0.830204
C	-5.407801	4.829365	-1.448140
H	-3.778383	5.836493	-2.435689
H	-6.799426	3.645533	-0.300627
H	-6.179613	5.416075	-1.937701
C	-2.664406	-2.420693	1.682122
N	-2.775897	-3.276444	0.598441
O	-3.624655	-1.769167	2.139017
C	-3.929494	-3.655120	-0.103803
C	-3.749304	-4.345952	-1.316863
C	-5.234089	-3.412656	0.363778
C	-4.849444	-4.783246	-2.046995
H	-2.741693	-4.523711	-1.683130
C	-6.325258	-3.858541	-0.383140
H	-5.372577	-2.878216	1.292938
C	-6.147499	-4.542824	-1.587056
H	-4.689843	-5.313170	-2.981847
H	-7.328506	-3.668676	-0.010688
H	-7.005583	-4.885784	-2.157551
C	-1.008797	-1.568909	3.463017
H	-1.911674	-1.054048	3.800544

H	-0.269149	-0.795318	3.203281
H	-2.476052	0.269311	0.739281
C	-2.909448	0.625599	-1.304303
C	-4.020437	-0.221693	-1.232180
C	-2.500160	1.107026	-2.554932
C	-4.717285	-0.579358	-2.387956
H	-4.329774	-0.621585	-0.270691
C	-3.196897	0.753390	-3.709227
H	-1.631628	1.754281	-2.630960
C	-4.307769	-0.090157	-3.628221
H	-5.563312	-1.255668	-2.313726
H	-2.869332	1.131732	-4.673503
H	-4.843815	-0.371373	-4.530241
H	3.240645	-1.518582	-0.855844
C	3.499169	-1.280219	1.233654
C	3.708545	-2.657777	1.361360
C	3.466095	-0.491636	2.392258
C	3.893863	-3.237304	2.618391
H	3.705124	-3.283866	0.473644
C	3.651129	-1.067276	3.647942
H	3.300469	0.578639	2.316355
C	3.867559	-2.443127	3.764751
H	4.050355	-4.309044	2.698824
H	3.626976	-0.442492	4.536253
H	4.010924	-2.891171	4.743841
Br	0.358849	-3.134776	-1.208178
C	-0.417265	-2.430508	4.598252
H	0.504429	-2.926766	4.277678
H	-0.179367	-1.807329	5.466734
H	-1.126064	-3.201050	4.917215
H	-1.895818	-3.566153	0.177311
C	-1.325786	-2.362879	2.245437
H	-0.535689	-2.946433	1.779885

[1]TS8[1c]-*R*

B3LYP/BSI SCF energy: -5896.186681a.u.

M06/BSII SCF energy in solution: -5894.866513a.u.

M06/BSII free energy in solution: -5893.833697a.u.

C	-1.543997	2.586853	-0.504415
N	-0.788434	1.959136	0.315128
O	-1.228647	3.878542	-0.774780
C	0.103123	4.120072	-0.185742
C	0.152606	2.961950	0.878908
H	1.143597	2.521884	0.931689
C	-0.291382	3.346118	2.290576
C	0.572871	3.167211	3.375601
C	-1.571800	3.862485	2.541280
C	0.176938	3.498181	4.673948
H	1.556293	2.743471	3.214349
C	-1.970132	4.195863	3.833738
H	-2.264688	4.016967	1.722163
C	-1.095589	4.014800	4.908132
H	0.864032	3.339659	5.500041
H	-2.965305	4.597794	4.002819
H	-1.407345	4.270653	5.916764
C	-3.237667	0.715324	-0.794174
N	-2.542482	-0.267612	-0.354511
O	-4.551359	0.482728	-1.015248
C	-4.893942	-0.842097	-0.506208
C	-3.450115	-1.419041	-0.135069
H	-3.434128	-1.619744	0.936550
C	-3.044644	-2.696180	-0.845870
C	-3.225313	-3.919313	-0.184994

C	-2.568940	-2.703367	-2.162564
C	-2.971136	-5.129175	-0.832679
H	-3.578540	-3.928780	0.843920
C	-2.308689	-3.912134	-2.809333
H	-2.371754	-1.767766	-2.673093
C	-2.519868	-5.127385	-2.153719
H	-3.125345	-6.067401	-0.307166
H	-1.940884	-3.900741	-3.831283
H	-2.327235	-6.065981	-2.665906
C	-2.856096	2.150511	-1.129923
C	-2.836417	2.338206	-2.671212
H	-2.605307	3.380430	-2.901283
H	-2.084905	1.693889	-3.129298
H	-3.823528	2.102539	-3.077660
C	-3.952305	3.089109	-0.534548
H	-4.912716	2.880334	-1.005629
H	-4.058247	2.948213	0.544678
H	-3.682470	4.127567	-0.730903
Ni	-0.444561	-0.269600	0.115764
Br	-0.099706	-0.039253	-2.512301
Si	2.981529	-0.971316	0.037425
H	1.423851	-0.567384	0.282900
O	4.234221	-1.484503	1.094592
O	2.430102	-2.650706	-0.132136
C	4.658488	-2.803253	1.398981
H	5.115248	-3.261849	0.508751
H	3.811606	-3.420396	1.709496
C	5.703211	-2.750369	2.508818
H	6.039217	-3.760777	2.767737
H	6.583399	-2.173263	2.198606
H	5.286299	-2.289034	3.409076
C	1.242513	-3.035680	-0.773050

H	0.371220	-2.525373	-0.323789
H	1.235479	-2.735406	-1.833351
C	1.039195	-4.545350	-0.639791
H	1.074117	-4.843843	0.411652
H	0.079742	-4.846836	-1.069720
H	1.823930	-5.118629	-1.155159
O	3.401444	0.702598	0.515736
C	3.320619	1.150709	1.857846
H	2.914741	2.173879	1.840090
H	2.629789	0.536694	2.447748
C	4.669906	1.231980	2.582823
H	5.350568	1.937952	2.083596
H	4.525789	1.607204	3.603071
H	5.135659	0.246412	2.647097
C	-5.648269	-1.583665	-1.613827
C	-6.408905	-2.722592	-1.316713
C	-5.585672	-1.146782	-2.942717
C	-7.080352	-3.412885	-2.324623
H	-6.488966	-3.070094	-0.292472
C	-6.265641	-1.833586	-3.950165
H	-5.010159	-0.263411	-3.190999
C	-7.013596	-2.970383	-3.646705
H	-7.663231	-4.294098	-2.072528
H	-6.207664	-1.474923	-4.973897
H	-7.542439	-3.504652	-4.430618
C	-5.792047	-0.631416	0.721403
C	-6.683997	0.450841	0.738243
C	-5.802915	-1.520155	1.803657
C	-7.545379	0.649154	1.815744
H	-6.699549	1.140240	-0.098086
C	-6.672251	-1.327063	2.880364
H	-5.137309	-2.376936	1.821481

C	-7.543305	-0.239278	2.893043
H	-8.222363	1.498691	1.811454
H	-6.659214	-2.027530	3.710165
H	-8.214209	-0.084752	3.732848
C	0.143073	5.535001	0.381310
C	1.317943	5.977521	1.006013
C	-0.936248	6.416945	0.271313
C	1.403627	7.267306	1.524173
H	2.172101	5.310791	1.081182
C	-0.846341	7.713656	0.784576
H	-1.846851	6.090916	-0.217345
C	0.320138	8.142843	1.414881
H	2.319669	7.590770	2.010023
H	-1.693873	8.386726	0.688988
H	0.388538	9.150351	1.814709
C	1.127508	4.014641	-1.330440
C	0.909392	4.824214	-2.459190
C	2.277247	3.218867	-1.287713
C	1.817976	4.847055	-3.512897
H	0.023320	5.449836	-2.502458
C	3.206414	3.256793	-2.338470
H	2.484338	2.529717	-0.475601
C	2.980120	4.069227	-3.450730
H	1.630371	5.485155	-4.372319
H	4.085086	2.614560	-2.294089
H	3.707920	4.105464	-4.258099
O	4.244044	-1.601266	-3.767494
P	4.827558	-0.891425	-2.532230
O	5.787731	-1.757376	-1.691875
O	5.256011	0.571586	-2.726197
O	3.428820	-0.746295	-1.557410
K	2.646490	0.566435	-3.902205
K	6.095029	0.356672	-0.137623
K	4.101305	-3.729930	-2.153267
C	0.304587	-1.549013	2.748076
N	0.438416	-2.935645	2.743546
C	1.320938	-3.755434	3.461338
C	2.323991	-3.261712	4.314888
C	1.164803	-5.148683	3.331633
C	3.145421	-4.156417	5.001270
H	2.435070	-2.192759	4.423983
C	1.996275	-6.027313	4.020753
H	0.378576	-5.540725	2.689812
C	2.997912	-5.537668	4.861714
H	3.914229	-3.759446	5.659009
H	1.854139	-7.098280	3.903082
H	3.645721	-6.220451	5.403237
C	-1.665832	-0.033091	2.802441
H	-2.424104	0.449102	2.177567
H	-0.993539	0.759287	3.138656
C	-0.859552	-1.036375	1.963296
O	1.067327	-0.829446	3.398589
H	-0.236769	-3.430346	2.179623
H	-1.500014	-1.888035	1.690320
C	-2.349043	-0.656345	4.031950
H	-1.610743	-1.070248	4.726711
H	-2.935784	0.092113	4.577010
H	-3.032290	-1.466767	3.746700

^{1}TS8^{1c}-*S*

B3LYP/BSI SCF energy: -5896.201179a.u.

M06/BSII SCF energy in solution: -5894.880736a.u.

M06/BSII free energy in solution: -5893.845776a.u.

C	-0.322871	-2.974865	-0.507803
N	-0.638352	-2.196704	0.449043
O	-1.304238	-3.741990	-1.056948
C	-2.570983	-3.391792	-0.414076
C	-2.070705	-2.382175	0.728633
H	-2.560783	-1.414138	0.595951
C	-2.320790	-2.845585	2.159392
C	-3.602087	-2.720115	2.713956
C	-1.309969	-3.415435	2.941474
C	-3.868593	-3.154122	4.011957
H	-4.403907	-2.285631	2.122569
C	-1.570792	-3.843412	4.244994
H	-0.310950	-3.508518	2.531050
C	-2.850458	-3.716528	4.784939
H	-4.870395	-3.051695	4.420009
H	-0.769723	-4.275866	4.837955
H	-3.053914	-4.050843	5.798209
C	2.139356	-2.289160	-0.732480
N	2.092200	-1.063202	-0.353886
O	3.372074	-2.792711	-0.968888
C	4.364103	-1.842404	-0.451706
C	3.487481	-0.535357	-0.320048
H	3.636529	-0.084375	0.657328
C	3.729904	0.546141	-1.361814
C	4.389986	1.718275	-0.967878
C	3.354140	0.398439	-2.703183
C	4.687010	2.715284	-1.899287
H	4.655261	1.853508	0.076207
C	3.648790	1.394419	-3.633366
H	2.808859	-0.482019	-3.022740
C	4.321890	2.552875	-3.236534
H	5.197965	3.617639	-1.575472
H	3.348899	1.264973	-4.669380
H	4.556033	3.324524	-3.965137
C	1.058255	-3.318706	-1.036867
C	1.028443	-3.534715	-2.574848
H	0.327246	-4.335662	-2.815644
H	0.716310	-2.620630	-3.082961
H	2.022519	-3.826457	-2.920973
C	1.453160	-4.661295	-0.349266
H	2.408442	-5.015607	-0.737116
H	1.542506	-4.541531	0.734051
H	0.689634	-5.412079	-0.563163
Ni	0.547972	0.187378	0.053222
Br	-0.255655	-0.067667	-2.414703
Si	-1.828733	2.489905	0.445488
H	-0.680152	1.329824	0.262803
O	-2.353863	3.587808	1.664216
O	-0.543308	3.615316	-0.028745
C	-2.147806	4.986302	1.774004
H	-2.772723	5.500053	1.027569
H	-1.101185	5.243017	1.582559
C	-2.547489	5.446138	3.172072
H	-2.396445	6.525946	3.282146
H	-3.604994	5.234323	3.370879
H	-1.950684	4.934179	3.933461
C	0.317563	3.325858	-1.124757
H	0.996867	2.499285	-0.871761
H	-0.264197	2.972614	-1.987566
C	1.158504	4.543023	-1.501331
H	1.745199	4.899530	-0.651877
H	1.849148	4.275378	-2.306766
H	0.550649	5.389560	-1.855077
O	-2.734870	1.252180	1.314342

C	-2.411990	0.888665	2.650585
H	-1.877879	-0.069882	2.653288
H	-1.757132	1.633993	3.119952
C	-3.670850	0.758571	3.508859
H	-4.417296	0.128707	3.011511
H	-3.439135	0.289122	4.470489
H	-4.111660	1.740137	3.726540
C	5.523439	-1.769123	-1.442336
C	6.681618	-1.061948	-1.090130
C	5.469416	-2.396402	-2.691042
C	7.754559	-0.972456	-1.974068
H	6.747786	-0.585843	-0.116762
C	6.550391	-2.314992	-3.572322
H	4.584193	-2.953425	-2.973605
C	7.694369	-1.600943	-3.220136
H	8.641652	-0.415940	-1.685577
H	6.492828	-2.812412	-4.536539
H	8.533653	-1.536574	-3.906622
C	4.863682	-2.420536	0.884788
C	5.214801	-3.781025	0.912341
C	5.041802	-1.656674	2.044181
C	5.715502	-4.367542	2.070917
H	5.097069	-4.378570	0.014050
C	5.553470	-2.248718	3.205250
H	4.768299	-0.607913	2.081975
C	5.889182	-3.599739	3.226137
H	5.977409	-5.422026	2.070840
H	5.677904	-1.640317	4.096362
H	6.283191	-4.053535	4.131118
C	-3.197927	-4.702770	0.083909
C	-4.586928	-4.879922	0.136700
C	-2.378676	-5.754293	0.518643
C	-5.139897	-6.070349	0.611983
H	-5.248611	-4.090857	-0.201864
C	-2.929939	-6.945716	0.987779
H	-1.301849	-5.645143	0.487608
C	-4.314810	-7.110293	1.037360
H	-6.219997	-6.183128	0.640281
H	-2.273063	-7.746981	1.314447
H	-4.744521	-8.039171	1.401043
C	-3.471563	-2.737447	-1.466901
C	-3.421597	-3.194383	-2.793033
C	-4.430163	-1.772377	-1.130451
C	-4.313314	-2.708824	-3.749928
H	-2.679085	-3.933767	-3.070597
C	-5.336158	-1.290487	-2.082985
H	-4.481864	-1.370870	-0.124269
C	-5.283865	-1.763568	-3.396730
H	-4.257554	-3.077756	-4.770483
H	-6.036441	-0.509918	-1.802196
H	-5.988227	-1.395634	-4.138173
O	-3.826604	3.417977	-2.990076
P	-4.217625	3.152053	-1.528218
O	-4.400227	4.415416	-0.663715
O	-5.210280	2.016816	-1.264419
O	-2.714359	2.505913	-0.955868
K	-3.213993	0.775281	-3.055630
K	-5.100840	2.767141	1.315443
K	-2.205717	5.271221	-1.918663
C	2.279752	1.632796	2.030639
N	1.942563	2.971011	1.889886
C	2.743914	4.106299	2.104250
C	4.103638	4.059390	2.465506
C	2.124857	5.365325	1.975853

C	4.805368	5.246659	2.677539
H	4.582822	3.099459	2.583896
C	2.841086	6.539865	2.188341
H	1.073395	5.410329	1.708761
C	4.191674	6.492077	2.539599
H	5.854060	5.189024	2.957745
H	2.336199	7.496890	2.084301
H	4.751763	7.407105	2.707364
C	1.408242	-0.604953	2.774923
H	2.333577	-1.081724	2.441076
H	0.603385	-1.316542	2.576734
C	1.147977	0.663019	1.953947
O	3.457810	1.291720	2.238138
H	0.992928	3.166862	1.585977
H	0.251078	1.155762	2.334116
C	1.501984	-0.352134	4.289580
H	0.579889	0.101088	4.673549
H	1.661711	-1.288990	4.836744

[1]IM6[1c]-*S*

B3LYP/BSI SCF energy: -2923.386146a.u.

M06/BSII SCF energy in solution: -2922.065426a.u.

M06/BSII free energy in solution: -2921.076505a.u.

C	-0.524187	-2.097389	-0.846351
N	-0.966271	-0.900744	-0.721641
O	-1.326199	-3.108532	-0.465980
C	-2.668432	-2.516161	-0.248704
C	-2.290393	-0.997895	-0.049472
C	1.881473	-1.624075	-0.712312
N	1.800056	-0.369548	-0.446028
O	3.036964	-2.250625	-0.397123
C	3.768790	-1.337754	0.494193
C	3.142141	0.039265	0.046445
Ni	0.108643	0.861375	-0.861566
C	0.834754	-2.493356	-1.392401
C	1.114308	-3.990031	-1.146649
H	0.341079	-4.589156	-1.629593
H	1.119980	-4.232765	-0.081893
H	2.085452	-4.259193	-1.565369
C	0.859778	-2.206559	-2.920465
H	1.837305	-2.476352	-3.332061
H	0.656420	-1.154568	-3.124516
H	0.097961	-2.813023	-3.418328
C	5.261560	-1.517505	0.242013
C	6.194525	-1.111077	1.205323
C	5.728807	-2.066874	-0.958689
C	7.562991	-1.239119	0.968033
H	5.854528	-0.704259	2.151965
C	7.097656	-2.204134	-1.190138
H	5.019760	-2.393634	-1.709468
C	8.020511	-1.787958	-0.230402
H	8.270411	-0.917426	1.726870
H	7.441015	-2.637732	-2.125019
H	9.085909	-1.894638	-0.412361
C	3.387156	-1.731525	1.931340
C	3.259985	-0.798339	2.967138
C	3.218177	-3.092053	2.233833
C	2.956858	-1.211348	4.268255
H	3.397532	0.261682	2.781366
C	2.909082	-3.503630	3.528637
H	3.330189	-3.828104	1.445355
C	2.774921	-2.563429	4.553103
H	2.861775	-0.468634	5.054939

H	2.778102	-4.561270	3.738741
H	2.535513	-2.883181	5.562916
C	-3.449765	-2.830313	-1.535144
C	-3.892677	-1.859784	-2.441332
C	-3.687944	-4.185866	-1.825279
C	-4.564932	-2.244936	-3.608198
H	-3.730932	-0.800765	-2.269909
C	-4.349408	-4.563589	-2.989512
H	-3.353705	-4.946674	-1.126370
C	-4.794645	-3.589150	-3.888195
H	-4.905186	-1.476925	-4.296855
H	-4.523919	-5.616505	-3.193000
H	-5.317388	-3.879652	-4.795257
C	-3.310313	-3.184398	0.957595
C	-2.604639	-4.054848	1.793704
C	-4.656356	-2.912557	1.240983
C	-3.233093	-4.641977	2.894917
H	-1.566208	-4.278438	1.579252
C	-5.278398	-3.490008	2.345566
H	-5.220256	-2.252657	0.588302
C	-4.568803	-4.360078	3.176995
H	-2.672764	-5.320829	3.531813
H	-6.320530	-3.265022	2.553227
H	-5.055786	-4.815604	4.034394
C	-1.171875	1.858457	-2.037984
C	-2.544576	2.041768	-1.491553
N	-2.718173	3.148347	-0.674614
O	-3.499463	1.281189	-1.733884
C	-3.861662	3.596861	0.002735
C	-3.699360	4.666143	0.904429
C	-5.148888	3.059952	-0.186078
C	-4.787403	5.181697	1.601875
H	-2.709672	5.091193	1.054630
C	-6.228217	3.586991	0.524634
H	-5.279801	2.245914	-0.883904
C	-6.064007	4.644134	1.420574
H	-4.634036	6.007755	2.291141
H	-7.215674	3.161039	0.365996
H	-6.913794	5.044969	1.964966
C	-1.173850	1.112038	-3.382913
H	-1.639054	0.128282	-3.264223
H	-0.133402	0.935733	-3.686763
C	1.122954	2.464901	-0.448962
H	1.703015	2.126908	0.424354
H	1.842263	2.621142	-1.265444
C	0.519938	3.817643	-0.056006
H	-0.230307	3.681985	0.738152
H	0.006412	4.281746	-0.911597
C	1.564239	4.830891	0.451056
H	2.337338	4.961970	-0.319435
H	2.077695	4.407035	1.326443
C	0.977384	6.198838	0.822949
H	0.470765	6.628359	-0.053730
H	0.197958	6.065183	1.588080
C	2.019630	7.198915	1.340228
H	2.800785	7.331594	0.578974
H	2.523215	6.772688	2.218907
C	1.425415	8.563436	1.704260
H	0.943725	9.029472	0.836930
H	2.194561	9.253545	2.067345
H	0.666602	8.468870	2.489715
H	-2.995139	-0.359692	-0.575781
C	-2.198181	-0.497535	1.384952
C	-3.178503	0.381443	1.860193

C -1.157047 -0.874417 2.244513

C -3.127720 0.867494 3.168127

H -3.974670 0.705511 1.196682

C -1.104380 -0.391269 3.551379

H -0.375153 -1.541875 1.896179

C -2.091270 0.480706 4.018026

H -3.892497 1.557118 3.513153

H -0.287996 -0.694349 4.200830

H -2.047645 0.860519 5.035093

H 3.013522 0.688634 0.909438

C 3.940665 0.801728 -1.000557

C 4.850470 1.778792 -0.577679

C 3.819900 0.545610 -2.370612

C 5.627236 2.479455 -1.499076

H 4.946988 1.999686 0.482238

C 4.592411 1.248741 -3.296454

H 3.103939 -0.187760 -2.724266

C 5.499770 2.216499 -2.864248

H 6.324315 3.236413 -1.151432

H 4.479398 1.042659 -4.357057

H 6.097730 2.766411 -3.585124

H -0.715121 2.839608 -2.184483

H -1.872661 3.663310 -0.476983

C -1.887844 1.868171 -4.517663

H -1.836542 1.311637 -5.461468

H -2.943152 2.022881 -4.274816

H -1.430152 2.850317 -4.686040

^{1}**TS10**^{1c}-*S*

B3LYP/BSI SCF energy: -2923.321119a.u.

M06/BSII SCF energy in solution: -2922.017227a.u.

M06/BSII free energy in solution: -2921.031033a.u.

C -1.081243 -1.544419 -0.747742

N -1.274127 -0.351012 -0.318081

O -2.035663 -2.463603 -0.491618

C -3.073417 -1.836962 0.348088

C -2.623959 -0.303158 0.289176

C 1.389940 -1.500281 -1.050302

N 1.613174 -0.316256 -0.624557

O 2.438393 -2.363997 -1.068120

C 3.512112 -1.741200 -0.285070

C 3.048715 -0.217086 -0.310745

Ni 0.013004 1.202656 -0.421907

C 0.078522 -2.040410 -1.604482

C 0.104820 -3.582404 -1.653555

H -0.841693 -3.955436 -2.046394

H 0.263965 -4.022223 -0.665861

H 0.914100 -3.914313 -2.306174

C -0.119428 -1.476214 -3.043341

H 0.709467 -1.803131 -3.679617

H -0.175883 -0.384042 -3.037190

H -1.047128 -1.878365 -3.462418

C 4.835688 -2.055675 -0.976935

C 6.036547 -2.083138 -0.255905

C 4.875975 -2.295006 -2.357354

C 7.248800 -2.332149 -0.900467

H 6.029189 -1.922258 0.816733

C 6.086927 -2.552427 -2.999044

H 3.955051 -2.288804 -2.927794

C 7.279506 -2.569219 -2.274629

H 8.168408 -2.349483 -0.322455

H 6.095795 -2.740447 -4.068887

H 8.222272 -2.769592 -2.775376

C	3.441017	-2.354060	1.122765
C	3.805849	-1.644387	2.274186
C	3.056659	-3.696599	1.262648
C	3.770824	-2.252205	3.532278
H	4.130288	-0.610726	2.207844
C	3.016392	-4.301971	2.517517
H	2.786836	-4.264497	0.379421
C	3.370985	-3.581158	3.659883
H	4.055396	-1.679870	4.410479
H	2.712217	-5.341569	2.601555
H	3.339779	-4.052382	4.637890
C	-4.403389	-2.131632	-0.360026
C	-4.742128	-1.458032	-1.544887
C	-5.256189	-3.152419	0.085765
C	-5.911725	-1.769748	-2.237602
H	-4.099645	-0.687523	-1.958100
C	-6.426367	-3.465085	-0.607682
H	-5.009133	-3.711557	0.980062
C	-6.763278	-2.770519	-1.768705
H	-6.149836	-1.216973	-3.140651
H	-7.071491	-4.256536	-0.236591
H	-7.676581	-3.009917	-2.306090
C	-2.972339	-2.443121	1.747764
C	-1.930272	-3.307801	2.098626
C	-3.923974	-2.111392	2.726108
C	-1.833676	-3.822136	3.394764
H	-1.195219	-3.589980	1.355334
C	-3.830797	-2.627621	4.016396
H	-4.740516	-1.441962	2.477429
C	-2.781794	-3.485017	4.358240
H	-1.013107	-4.488999	3.643948
H	-4.577796	-2.356233	4.756745
H	-2.708313	-3.885695	5.365186
C	-0.896291	2.392648	-1.763092
N	-2.289761	2.621722	-1.544509
O	-0.502617	1.827719	-2.825745
C	-3.383247	2.282464	-2.328576
C	-4.670241	2.627431	-1.853012
C	-3.287066	1.635637	-3.580965
C	-5.810596	2.345058	-2.597394
H	-4.762640	3.140066	-0.897611
C	-4.442715	1.366505	-4.313767
H	-2.305703	1.381443	-3.954732
C	-5.711499	1.711644	-3.839439
H	-6.783523	2.629414	-2.204937
H	-4.342600	0.880447	-5.281400
H	-6.600507	1.502745	-4.426920
C	1.077723	3.974218	-1.697813
H	1.674339	3.243022	-2.248696
H	1.757402	4.496476	-1.013537
C	1.041754	2.570968	0.614145
H	0.959474	1.602700	1.165359
H	2.062828	2.661657	0.234356
C	0.747565	3.658589	1.651723
H	-0.294971	3.569625	1.984698
H	0.853079	4.656972	1.204925
C	1.669563	3.574230	2.878833
H	2.717394	3.634421	2.550350
H	1.553392	2.588583	3.352214
C	1.400797	4.669656	3.918969
H	1.514076	5.655568	3.445706
H	0.352616	4.609609	4.245589
C	2.317674	4.591538	5.146470
H	3.364902	4.650036	4.819257

H	2.203810	3.606897	5.620989
C	2.043490	5.690033	6.178721
H	2.183506	6.686013	5.743173
H	2.713047	5.607477	7.041376
H	1.013815	5.636146	6.550810
H	-3.265784	0.214910	-0.425798
C	-2.681485	0.494567	1.574328
C	-3.779094	1.333730	1.802715
C	-1.672539	0.422114	2.543936
C	-3.875111	2.081727	2.979396
H	-4.563377	1.400189	1.052338
C	-1.765251	1.169072	3.717616
H	-0.816428	-0.222914	2.377855
C	-2.866496	2.001050	3.939855
H	-4.733888	2.727323	3.140925
H	-0.976783	1.101114	4.461879
H	-2.935614	2.582422	4.854969
H	3.150221	0.208041	0.688868
C	3.787503	0.695027	-1.277737
C	4.964758	1.326702	-0.854770
C	3.333646	0.917138	-2.582939
C	5.686792	2.146192	-1.721373
H	5.322988	1.173146	0.160520
C	4.054801	1.739585	-3.451383
H	2.396553	0.480975	-2.909978
C	5.233936	2.352307	-3.026203
H	6.597965	2.626889	-1.376650
H	3.684212	1.909005	-4.458255
H	5.791439	2.994091	-3.702428
H	-2.513727	3.050272	-0.657807
C	-0.000148	3.230943	-0.903675
H	-0.603738	3.923413	-0.316535
C	0.474987	4.988916	-2.679875
H	-0.152244	4.486245	-3.420923
H	-0.138861	5.734466	-2.159841
H	1.265798	5.526896	-3.214429

^{1}TS10^{1c}-*R*

B3LYP/BSI SCF energy: -2923.32119a.u.

M06/BSII SCF energy in solution: -2922.016993a.u.

M06/BSII free energy in solution: -2921.028496a.u.

C	0.559248	-0.433225	-1.649936
N	1.111193	-0.042317	-0.560043
O	1.186362	-1.415539	-2.351495
C	2.217852	-1.972186	-1.475439
C	2.378584	-0.786299	-0.421516
C	-1.833477	-0.045324	-1.191516
N	-1.707650	0.142266	0.068754
O	-3.060829	-0.334740	-1.670960
C	-4.019947	-0.224319	-0.553401
C	-3.028245	-0.061205	0.693253
Ni	-0.021594	0.780908	1.072689
C	-0.739064	0.089594	-2.243988
C	-1.116419	-0.687472	-3.521747
H	-0.324830	-0.582823	-4.266654
H	-1.259587	-1.752343	-3.325303
H	-2.043074	-0.285379	-3.933280
C	-0.590025	1.599281	-2.579797
H	-1.517353	1.965300	-3.029607
H	-0.387185	2.185165	-1.684448
H	0.222022	1.742195	-3.299348
C	-4.837664	1.044990	-0.837196
C	-6.084817	0.969049	-1.475068

C	-4.302349	2.313979	-0.558223
C	-6.793921	2.125806	-1.800691
H	-6.509229	0.001740	-1.718374
C	-5.015017	3.468295	-0.887029
H	-3.326250	2.431812	-0.095907
C	-6.263820	3.381380	-1.503273
H	-7.761213	2.041431	-2.288267
H	-4.583343	4.437948	-0.656205
H	-6.816817	4.282707	-1.752978
C	-4.866987	-1.488962	-0.527057
C	-5.891504	-1.613869	0.424270
C	-4.657213	-2.538439	-1.427262
C	-6.676279	-2.763103	0.479534
H	-6.079391	-0.804560	1.122895
C	-5.447156	-3.690266	-1.373644
H	-3.880188	-2.450065	-2.176859
C	-6.456738	-3.809094	-0.420473
H	-7.461404	-2.840994	1.226144
H	-5.270634	-4.493173	-2.084095
H	-7.070093	-4.704593	-0.378790
C	3.439280	-2.285342	-2.337599
C	4.349789	-3.284597	-1.969385
C	3.685401	-1.553765	-3.508029
C	5.482446	-3.538375	-2.744068
H	4.172450	-3.881292	-1.081407
C	4.813238	-1.813509	-4.285695
H	2.985862	-0.785611	-3.814954
C	5.719117	-2.805001	-3.906375
H	6.174291	-4.318784	-2.440074
H	4.981848	-1.238296	-5.191638
H	6.597032	-3.006770	-4.513222
C	1.626581	-3.237681	-0.833725
C	0.690644	-3.999124	-1.549399
C	2.037600	-3.700478	0.423441
C	0.167459	-5.176328	-1.015462
H	0.375217	-3.663196	-2.530586
C	1.520899	-4.884366	0.956033
H	2.770972	-3.148570	1.003197
C	0.580575	-5.625150	0.240941
H	-0.560230	-5.746664	-1.585660
H	1.855042	-5.221981	1.932791
H	0.177699	-6.544979	0.654814
C	-0.112173	2.258462	2.509763
C	-0.288276	2.876392	1.121071
N	0.890099	3.476660	0.614811
O	-1.403058	3.176428	0.635369
C	1.051177	4.409043	-0.412416
C	2.367854	4.795086	-0.741767
C	-0.017362	5.005591	-1.112305
C	2.609710	5.735451	-1.738476
H	3.202210	4.351047	-0.204480
C	0.245652	5.944355	-2.109629
H	-1.028299	4.721705	-0.859137
C	1.549975	6.318818	-2.437299
H	3.635074	6.016999	-1.965113
H	-0.593112	6.392163	-2.636844
H	1.737710	7.052331	-3.215529
C	-1.400602	2.301859	3.338850
H	-2.230713	1.945954	2.726074
H	-1.314168	1.617315	4.191260
C	0.896699	0.747494	2.842748
H	0.745474	-0.262318	2.355192
H	0.461370	0.611478	3.837317
C	2.393231	1.049354	2.998068

H	2.864720	1.184751	2.017117
H	2.519382	1.997329	3.541636
C	3.151262	-0.044641	3.769289
H	2.649853	-0.219160	4.732035
H	3.083749	-0.994161	3.218677
C	4.626711	0.294858	4.018827
H	4.689245	1.228078	4.596809
H	5.119569	0.498968	3.058045
C	5.393901	-0.807730	4.760376
H	4.898464	-1.010971	5.719559
H	5.331775	-1.742008	4.184910
C	6.864943	-0.458923	5.009013
H	6.958947	0.457648	5.602540
H	7.382270	-1.258768	5.549198
H	7.398572	-0.295096	4.065644
C	-2.985595	-1.185676	1.712867
C	-3.608936	-1.016096	2.954685
C	-2.324642	-2.392992	1.451284
C	-3.586111	-2.031859	3.912394
H	-4.111388	-0.077834	3.177286
C	-2.296838	-3.408115	2.406527
H	-1.820776	-2.539617	0.501231
C	-2.929098	-3.232014	3.639970
H	-4.074074	-1.880777	4.871273
H	-1.773997	-4.334154	2.185846
H	-2.905297	-4.022929	4.384431
H	-3.296497	0.853930	1.222669
H	2.413857	-1.208275	0.583509
C	3.599420	0.102683	-0.596110
C	4.819006	-0.295084	-0.029653
C	3.553844	1.298872	-1.320311
C	5.970844	0.472295	-0.198264
H	4.870002	-1.217609	0.543327
C	4.706382	2.070767	-1.488545
H	2.612005	1.641967	-1.734063
C	5.917944	1.659314	-0.932721
H	6.907147	0.144888	0.244664
H	4.647336	2.997860	-2.050848
H	6.813176	2.260090	-1.065038
H	1.751777	3.132336	1.014223
H	0.648759	2.864199	3.014503
C	-1.725968	3.714393	3.846309
H	-0.907490	4.119073	4.454106
H	-1.896314	4.392017	3.005110
H	-2.628756	3.711323	4.467253

^{1}TS12^{1c}-*R*

B3LYP/BSI SCF energy: -2923.343703a.u.

M06/BSII SCF energy in solution: -2922.020693a.u.

M06/BSII free energy in solution: -2921.036404a.u.

C	-1.508546	-1.916708	0.693977
N	-1.587174	-0.929012	-0.115542
O	-2.581371	-2.157308	1.484617
C	-3.680865	-1.325441	0.966650
C	-2.867358	-0.245271	0.132976
H	-3.350363	-0.072777	-0.827309
C	-2.657035	1.107738	0.797414
C	-3.591324	2.129252	0.578717
C	-1.559581	1.366861	1.626822
C	-3.444728	3.374950	1.188455
H	-4.440976	1.949826	-0.074575
C	-1.406092	2.614897	2.234069
H	-0.807010	0.602027	1.784412

C	-2.350859	3.620759	2.021339
H	-4.181750	4.152684	1.009564
H	-0.541576	2.799135	2.865002
H	-2.235802	4.589420	2.499967
C	0.949263	-2.182932	0.505458
N	1.146314	-1.304398	-0.408750
O	2.017463	-2.549568	1.248991
C	3.060303	-1.538143	1.002607
C	2.591469	-0.972380	-0.402951
H	2.699843	0.110872	-0.424811
C	3.315239	-1.531179	-1.617660
C	4.379489	-0.798975	-2.160889
C	2.974445	-2.759864	-2.195392
C	5.097700	-1.293131	-3.250432
H	4.623378	0.173189	-1.742113
C	3.688737	-3.251445	-3.288957
H	2.137655	-3.329303	-1.804534
C	4.755355	-2.522035	-3.816967
H	5.917698	-0.711567	-3.662232
H	3.407984	-4.203440	-3.730839
H	5.309572	-2.904857	-4.669269
Ni	-0.289942	-0.677534	-1.724758
C	-0.357263	-2.900228	0.814583
C	-0.581258	-4.018968	-0.245698
H	-1.536212	-4.518619	-0.059853
H	-0.594435	-3.594713	-1.253142
H	0.219392	-4.761579	-0.175256
C	-0.319523	-3.518575	2.228082
H	0.487896	-4.250023	2.289777
H	-0.148508	-2.759253	2.995261
H	-1.266496	-4.017437	2.438280
C	2.937537	-0.517067	2.146408
C	2.654114	-0.999631	3.435288
C	3.155641	0.855828	1.976758
C	2.566100	-0.131670	4.520817
H	2.503129	-2.063484	3.582444
C	3.072541	1.725554	3.070511
H	3.394611	1.277222	1.004713
C	2.773358	1.238193	4.341196
H	2.341866	-0.526675	5.507919
H	3.242899	2.785931	2.915333
H	2.708040	1.917840	5.186174
C	4.416476	-2.233447	1.025193
C	5.586451	-1.476695	1.175921
C	4.525370	-3.621155	0.876977
C	6.836766	-2.093504	1.168043
H	5.521931	-0.401876	1.310366
C	5.777290	-4.238335	0.880213
H	3.628854	-4.219403	0.767249
C	6.937745	-3.477989	1.022370
H	7.732200	-1.489624	1.283718
H	5.842164	-5.317238	0.770396
H	7.911524	-3.959183	1.023378
C	-4.473511	-0.791779	2.155550
C	-5.778363	-0.314212	1.972830
C	-3.911861	-0.743424	3.437142
C	-6.500510	0.209405	3.044618
H	-6.240662	-0.364119	0.992264
C	-4.639681	-0.228681	4.510970
H	-2.907415	-1.117187	3.595355
C	-5.934827	0.252450	4.319727
H	-7.510562	0.574650	2.882416
H	-4.189823	-0.205873	5.499521
H	-6.500076	0.652480	5.156455

C	-4.542830	-2.247913	0.089273
C	-4.766752	-3.564388	0.523763
C	-5.164509	-1.821219	-1.089996
C	-5.570709	-4.432808	-0.211014
H	-4.303519	-3.904925	1.443493
C	-5.976555	-2.690849	-1.824437
H	-5.028648	-0.809723	-1.457436
C	-6.179293	-3.999223	-1.391368
H	-5.725824	-5.448660	0.141268
H	-6.442674	-2.339179	-2.740082
H	-6.805732	-4.675394	-1.965638
C	1.489756	2.468715	-2.548538
C	2.298889	2.878947	-1.421967
N	1.666730	3.777943	-0.564224
O	3.464865	2.486797	-1.229003
C	2.204196	4.500119	0.509864
C	1.307255	5.197185	1.340842
C	3.585130	4.592512	0.763965
C	1.776575	5.965556	2.401916
H	0.239026	5.123725	1.152454
C	4.037526	5.368855	1.831551
H	4.275760	4.055441	0.129625
C	3.147020	6.056995	2.658068
H	1.066539	6.495634	3.030737
H	5.106685	5.434700	2.014196
H	3.513488	6.656876	3.485462
C	2.029198	1.667380	-3.681915
H	2.991416	1.236942	-3.398018
H	1.325910	0.845881	-3.855243
C	-0.996629	-0.551045	-3.579971
H	-1.746472	-1.358790	-3.656180
H	-0.162850	-0.893112	-4.222154
C	-1.595940	0.713820	-4.220370
H	-0.829866	1.499237	-4.304468
H	-1.917571	0.517363	-5.259336
C	-2.796543	1.305807	-3.470772
H	-3.564617	0.524453	-3.353171
H	-2.479062	1.582119	-2.455293
C	-3.425449	2.522824	-4.161713
H	-3.746261	2.241120	-5.175081
H	-2.657560	3.299312	-4.296510
C	-4.617280	3.125159	-3.406013
H	-5.387512	2.351940	-3.271057
H	-4.295200	3.412433	-2.395230
C	-5.235437	4.338540	-4.108471
H	-5.597376	4.073953	-5.108656
H	-6.082701	4.744451	-3.545020
H	-4.500110	5.142625	-4.228394
C	2.184428	2.494337	-4.977085
H	2.531632	1.854130	-5.794514
H	1.232162	2.940618	-5.282748
H	2.910792	3.302731	-4.844342
H	0.684265	3.934500	-0.740079
H	0.465052	2.828433	-2.618698

^{1}TS12^{1c}-*S*

B3LYP/BSI SCF energy: -2923.334701a.u.

M06/BSII SCF energy in solution: -2922.02508a.u.

M06/BSII free energy in solution: -2921.045457a.u.

C	0.963633	-1.134339	-1.456604
N	1.256630	-0.622460	-0.318163
O	1.993058	-1.512936	-2.249512
C	3.193980	-1.525531	-1.379323

C	2.733185	-0.512496	-0.258212
H	3.069542	-0.859869	0.717667
C	3.206230	0.924751	-0.418862
C	4.428286	1.300694	0.156469
C	2.475104	1.883263	-1.127600
C	4.914922	2.598854	0.012490
H	5.001995	0.572539	0.722679
C	2.955692	3.187072	-1.267206
H	1.511424	1.628265	-1.553424
C	4.178743	3.546520	-0.701444
H	5.863438	2.871561	0.466349
H	2.362717	3.919870	-1.805260
H	4.550702	4.561785	-0.804956
C	-1.553009	-0.984937	-1.190802
N	-1.555081	-0.491723	-0.003580
O	-2.755042	-1.207234	-1.768730
C	-3.785304	-0.584717	-0.930352
C	-2.972656	-0.336151	0.414426
H	-3.114430	0.700940	0.719345
C	-3.322126	-1.212376	1.604025
C	-4.201201	-0.719034	2.575649
C	-2.802850	-2.503662	1.756315
C	-4.563052	-1.499742	3.674178
H	-4.603004	0.286775	2.477770
C	-3.160087	-3.284659	2.855213
H	-2.098760	-2.893406	1.028716
C	-4.041967	-2.786181	3.816068
H	-5.243048	-1.099435	4.420420
H	-2.742293	-4.281162	2.964311
H	-4.314902	-3.394099	4.673667
Ni	0.033108	-0.487198	1.338270
C	-0.393016	-1.428009	-2.060011
C	-0.509390	-2.967006	-2.266004
H	0.310775	-3.313720	-2.896527
H	-0.453133	-3.493273	-1.308852
H	-1.458921	-3.206532	-2.748855
C	-0.482600	-0.712051	-3.436741
H	-1.432015	-0.949285	-3.918914
H	-0.411207	0.373626	-3.323325
H	0.334825	-1.048997	-4.076581
C	-4.203171	0.716991	-1.632117
C	-4.259491	0.740798	-3.034491
C	-4.595322	1.863866	-0.930258
C	-4.671006	1.886200	-3.713035
H	-3.979225	-0.145428	-3.592927
C	-5.013837	3.010676	-1.610523
H	-4.585333	1.884130	0.154784
C	-5.048090	3.028310	-3.003112
H	-4.700113	1.884870	-4.798912
H	-5.302822	3.891047	-1.044736
H	-5.364649	3.922681	-3.531081
C	-4.958548	-1.558851	-0.828102
C	-6.223039	-1.102027	-0.433380
C	-4.792313	-2.921358	-1.104897
C	-7.292348	-1.988131	-0.308877
H	-6.380821	-0.047464	-0.233899
C	-5.865822	-3.805696	-0.990014
H	-3.824171	-3.291150	-1.420239
C	-7.119150	-3.344290	-0.588811
H	-8.264212	-1.613842	-0.000607
H	-5.718373	-4.857907	-1.216123
H	-7.954006	-4.033070	-0.499088
C	4.394827	-1.081249	-2.196174
C	5.687623	-1.346318	-1.721863

C	4.246390	-0.370377	-3.391889
C	6.806631	-0.905350	-2.426757
H	5.819468	-1.907166	-0.801518
C	5.367814	0.062776	-4.101425
H	3.252631	-0.158321	-3.767632
C	6.650759	-0.200701	-3.621876
H	7.800398	-1.118055	-2.043394
H	5.234745	0.609574	-5.030529
H	7.522173	0.138346	-4.174436
C	3.313651	-2.978626	-0.893631
C	3.770575	-3.948548	-1.802554
C	2.900129	-3.399397	0.377796
C	3.828956	-5.293771	-1.447167
H	4.087075	-3.641625	-2.794219
C	2.964006	-4.750059	0.735309
H	2.511495	-2.695380	1.104846
C	3.429223	-5.700145	-0.171322
H	4.190777	-6.024395	-2.165099
H	2.645333	-5.050648	1.729179
H	3.480582	-6.747930	0.110629
C	-1.204679	2.109495	2.760783
C	-0.787639	3.212346	1.915725
N	-0.939638	2.984302	0.542623
C	-0.709824	3.859941	-0.523703
C	-0.229515	5.173837	-0.365039
C	-0.991641	3.391893	-1.822760
C	-0.032167	5.975024	-1.489516
H	-0.015908	5.534324	0.630880
C	-0.783002	4.203944	-2.933911
H	-1.400999	2.392836	-1.950815
C	-0.297675	5.504856	-2.777817
H	0.340546	6.986506	-1.350107
H	-1.012512	3.819943	-3.924369
H	-0.137636	6.141461	-3.642880
C	-1.121154	2.233017	4.244650
H	-0.985579	1.239542	4.685532
H	-0.248822	2.842131	4.502106
C	0.656139	-1.078389	3.132118
H	1.041887	-2.104655	2.984148
H	-0.305533	-1.221247	3.656211
C	1.607949	-0.336230	4.084876
H	1.209966	0.658425	4.321586
H	1.670767	-0.866141	5.052876
C	3.040231	-0.159258	3.566982
H	3.437171	-1.139005	3.254074
H	3.020372	0.474149	2.670344
C	3.994627	0.452933	4.601425
H	4.010673	-0.182004	5.499538
H	3.597903	1.425509	4.926541
C	5.430339	0.642052	4.095385
H	5.823840	-0.325985	3.752838
H	5.417592	1.296343	3.213145
C	6.374341	1.229051	5.150149
H	6.434378	0.580194	6.031753
H	7.390392	1.353902	4.759796
H	6.025339	2.211291	5.488948
O	-0.377670	4.286492	2.374285
C	-2.378295	2.893575	4.856836
H	-3.281783	2.316572	4.629246
H	-2.286894	2.959233	5.946706
H	-2.505542	3.905189	4.461334
H	-1.773984	1.289044	2.338407
H	-1.107465	2.020260	0.284756

^{2}TS16^{1c}-*R*

B3LYP/BSI SCF energy: -2936.785026a.u.

M06/BSII SCF energy in solution: -2935.434977a.u.

M06/BSII free energy in solution: -2934.451481a.u.

C	0.515366	-1.849883	-0.881141
N	0.879470	-1.100688	0.094376
O	1.224123	-2.968811	-1.096265
C	2.136907	-3.165317	0.054284
C	2.012156	-1.760645	0.794562
C	-1.751265	-0.806554	-1.303124
N	-1.798692	-0.112753	-0.226772
O	-2.866834	-0.836453	-2.058248
C	-3.882542	0.025444	-1.444332
Ni	-0.167752	0.522369	0.943218
C	-0.622520	-1.653626	-1.871107
C	-1.216908	-3.042133	-2.238914
H	-0.435759	-3.675419	-2.658240
H	-1.626389	-3.548292	-1.359744
H	-2.009365	-2.917029	-2.977431
C	-0.051819	-0.988238	-3.157039
H	-0.864006	-0.801696	-3.864232
H	0.469077	-0.050320	-2.948993
H	0.657505	-1.676292	-3.625266
C	-4.130081	1.196233	-2.402329
C	-4.547959	2.451040	-1.940645
C	-4.012689	0.996146	-3.785194
C	-4.834853	3.483064	-2.837368
H	-4.658206	2.637805	-0.876882
C	-4.289009	2.029030	-4.679924
H	-3.702134	0.027168	-4.159506
C	-4.702853	3.277480	-4.210295
H	-5.157126	4.448005	-2.457215
H	-4.184141	1.855798	-5.747048
H	-4.921595	4.080512	-4.907808
C	-5.159086	-0.809017	-1.282242
C	-5.151244	-2.195813	-1.471262
C	-6.367107	-0.185969	-0.938918
C	-6.321644	-2.942003	-1.315703
H	-4.232435	-2.697225	-1.748238
C	-7.532103	-0.932298	-0.775096
H	-6.405075	0.888958	-0.802434
C	-7.515744	-2.315411	-0.964348
H	-6.294012	-4.016725	-1.471691
H	-8.455667	-0.429144	-0.504076
H	-8.425353	-2.896060	-0.842263
C	3.523804	-3.487012	-0.484415
C	4.551015	-3.797582	0.419443
C	3.806936	-3.487949	-1.853310
C	5.834274	-4.086863	-0.037326
H	4.344935	-3.810984	1.485121
C	5.094051	-3.783962	-2.311133
H	3.020981	-3.260708	-2.563651
C	6.111743	-4.081148	-1.406906
H	6.618437	-4.318282	0.677614
H	5.295656	-3.781091	-3.378594
H	7.112300	-4.308728	-1.762948
C	1.527258	-4.337812	0.840922
C	1.883838	-5.655287	0.510567
C	0.532437	-4.138580	1.811019
C	1.282030	-6.741202	1.144653
H	2.640452	-5.833671	-0.245314
C	-0.066565	-5.228755	2.446832
H	0.206300	-3.142570	2.095607

C	0.306188	-6.532125	2.120563
H	1.579686	-7.750946	0.876159
H	-0.824235	-5.047165	3.203586
H	-0.158133	-7.377267	2.620962
C	1.857330	2.441318	-1.898285
O	1.921772	1.735937	-2.916570
C	4.264376	3.195434	-1.865474
C	5.185392	3.955793	-1.121790
C	4.673063	2.620270	-3.081983
C	6.487109	4.135158	-1.578290
H	4.875317	4.401138	-0.179123
C	5.982501	2.811619	-3.524539
H	3.964766	2.037013	-3.652426
C	6.897540	3.562562	-2.784833
H	7.182163	4.725093	-0.987545
H	6.286948	2.362715	-4.466110
H	7.913415	3.701439	-3.141751
C	-0.658591	2.755398	-2.021462
H	-0.697450	1.875276	-2.669150
H	-1.524987	2.722581	-1.356493
C	-0.787592	2.303340	1.629769
H	-1.627482	2.012859	2.271754
H	-1.183885	2.936694	0.824345
C	0.246254	3.069897	2.459545
H	0.623458	2.418502	3.257548
H	1.123941	3.333154	1.845787
C	-0.300809	4.363745	3.093107
H	-0.679778	5.025174	2.299198
H	-1.168353	4.110969	3.718587
C	0.730310	5.123013	3.938171
H	1.604782	5.366299	3.315817
H	1.101520	4.462452	4.734574

C	0.185911	6.413245	4.564803
H	-0.183859	7.074218	3.768463
H	-0.687146	6.171229	5.186163
C	1.221255	7.162110	5.410300
H	2.092554	7.447939	4.809358
H	0.802407	8.076929	5.843259
H	1.582460	6.538702	6.236303
Br	-0.273106	-0.595941	3.201722
C	3.257198	-0.901281	0.876823
C	3.859238	-0.323235	-0.248577
C	3.853491	-0.722866	2.132647
C	5.056107	0.381351	-0.121811
H	3.400741	-0.423508	-1.227846
C	5.043801	-0.005531	2.262092
H	3.376255	-1.144366	3.013212
C	5.652600	0.539169	1.131613
H	5.521165	0.810463	-1.002363
H	5.490844	0.124935	3.243568
H	6.583494	1.091680	1.222923
H	1.681164	-1.924566	1.819181
C	-3.157640	0.458113	-0.086823
H	-3.047126	1.542909	-0.074948
C	-3.833480	0.049584	1.208129
C	-4.538898	1.010921	1.940630
C	-3.789138	-1.265040	1.684442
C	-5.203568	0.665835	3.117989
H	-4.558313	2.042671	1.597193
C	-4.450576	-1.612683	2.860592
H	-3.221676	-2.017100	1.146652
C	-5.162683	-0.650055	3.578841
H	-5.741234	1.425722	3.677611
H	-4.401492	-2.635293	3.222502

H	-5.672470	-0.922586	4.498314
N	2.967163	3.068138	-1.346564
H	2.797601	3.580940	-0.492733
C	-0.763924	4.014147	-2.915843
H	-0.729799	4.930330	-2.316673
H	0.059488	4.044226	-3.635444
H	-1.707213	4.004945	-3.470779
C	0.602158	2.746781	-1.217094
H	0.639311	3.364735	-0.326149

^{2}TS16^{1c}-*S*

B3LYP/BSI SCF energy: -2936.784093a.u.

M06/BSII SCF energy in solution: -2935.442474a.u.

M06/BSII free energy in solution: -2934.454547a.u.

C	2.400080	1.231799	0.151969
N	1.969938	0.038065	-0.045666
O	3.719260	1.456110	-0.027709
C	4.239558	0.265424	-0.745104
C	3.187982	-0.801169	-0.295348
C	0.154425	2.334817	0.534262
N	-0.585403	1.402134	0.040704
O	-0.468474	3.435690	0.993766
C	-1.914246	3.124052	1.049944
Ni	-0.047053	-0.537393	-0.504524
C	1.658729	2.455250	0.637817
C	2.092966	3.688345	-0.205861
H	3.167907	3.838897	-0.104872
H	1.870327	3.541369	-1.266447
H	1.571003	4.579036	0.146549
C	2.047061	2.690759	2.126679
H	1.569193	3.601573	2.490630
H	1.724665	1.856092	2.753655
H	3.130669	2.800465	2.209162
C	-2.203297	2.695798	2.494967
C	-2.642046	1.420274	2.862522
C	-1.993780	3.651604	3.506024
C	-2.874384	1.105899	4.206936
H	-2.792567	0.628720	2.138469
C	-2.222834	3.339289	4.842236
H	-1.657239	4.648002	3.235735
C	-2.669670	2.062014	5.197607
H	-3.187691	0.096644	4.451989
H	-2.057143	4.093032	5.606868
H	-2.850412	1.817399	6.240444
C	-2.692908	4.380552	0.683157
C	-2.092853	5.454858	0.017350
C	-4.056671	4.456389	0.999626
C	-2.841470	6.582903	-0.323617
H	-1.039120	5.412155	-0.230791
C	-4.805802	5.577833	0.647286
H	-4.531514	3.639395	1.534093
C	-4.199724	6.647781	-0.013934
H	-2.358413	7.411436	-0.833689
H	-5.861616	5.618210	0.898798
H	-4.780721	7.525650	-0.281071
C	5.669826	0.003847	-0.305337
C	6.366435	-1.062721	-0.891778
C	6.319124	0.795856	0.646388
C	7.679592	-1.340334	-0.519773
H	5.881102	-1.673718	-1.647481
C	7.639796	0.521309	1.011948
H	5.794407	1.629050	1.099077
C	8.322630	-0.547723	0.434514

H	8.202906	-2.173702	-0.979321
H	8.131800	1.147612	1.750762
H	9.348172	-0.761475	0.720997
C	4.170894	0.620864	-2.239728
C	5.081219	1.569712	-2.735322
C	3.210164	0.093606	-3.113799
C	5.036773	1.978009	-4.065646
H	5.834310	1.983931	-2.072142
C	3.171325	0.501199	-4.451642
H	2.480942	-0.636762	-2.780333
C	4.080365	1.441366	-4.931998
H	5.753439	2.709611	-4.428041
H	2.423346	0.073265	-5.112618
H	4.049045	1.752037	-5.972441
C	-1.539382	-2.411178	2.058949
N	-1.695723	-3.529770	1.256578
C	-2.819365	-4.352941	1.095277
C	-4.021984	-4.177334	1.803665
C	-2.710007	-5.422169	0.185435
C	-5.080133	-5.061097	1.590238
H	-4.105451	-3.356671	2.501247
C	-3.777996	-6.290707	-0.017252
H	-1.781475	-5.564032	-0.362082
C	-4.973742	-6.117694	0.684802
H	-6.003636	-4.914658	2.144340
H	-3.671762	-7.107808	-0.725410
H	-5.807048	-6.796006	0.527823
C	0.214854	-0.907337	3.134254
H	1.134594	-0.397825	2.832151
H	-0.567799	-0.157558	3.270676
C	-1.906766	-0.893195	-1.015020
H	-2.136798	0.032918	-1.547774
H	-2.433502	-0.885113	-0.049697
C	-2.410026	-2.049312	-1.874245
H	-1.899214	-2.044672	-2.844515
H	-2.182134	-3.015842	-1.419434
C	-3.932260	-1.963829	-2.106183
H	-4.447134	-1.993935	-1.135164
H	-4.181976	-0.992390	-2.558207
C	-4.472838	-3.091643	-2.995352
H	-4.217420	-4.059393	-2.542514
H	-3.961860	-3.064142	-3.968947
C	-5.989122	-3.025520	-3.218644
H	-6.497487	-3.057568	-2.245299
H	-6.248931	-2.055454	-3.665559
C	-6.520611	-4.154726	-4.107715
H	-6.306684	-5.135880	-3.668482
H	-7.604803	-4.082446	-4.247766
H	-6.054881	-4.130479	-5.099918
Br	0.715572	-2.673596	-1.465393
C	3.555991	-1.624526	0.931005
C	3.798107	-1.043132	2.183658
C	3.683511	-3.013398	0.802151
C	4.161421	-1.828956	3.276955
H	3.717568	0.031641	2.309700
C	4.045931	-3.802844	1.895379
H	3.478840	-3.479007	-0.156631
C	4.286150	-3.213138	3.136334
H	4.349617	-1.359765	4.238414
H	4.135274	-4.878645	1.775060
H	4.567748	-3.825161	3.988401
H	2.965728	-1.495252	-1.099631
C	-1.970639	1.964675	-0.007524
H	-2.679662	1.204699	0.307171

C	-2.352047	2.406845	-1.416703
C	-3.702108	2.366507	-1.792877
C	-1.412453	2.867263	-2.345025
C	-4.104409	2.785928	-3.059460
H	-4.443615	1.994311	-1.091243
C	-1.812205	3.283950	-3.616566
H	-0.358539	2.882773	-2.090792
C	-3.158668	3.247567	-3.977503
H	-5.154827	2.742794	-3.331898
H	-1.066450	3.631619	-4.325823
H	-3.468574	3.567447	-4.968011
O	-2.458215	-1.927364	2.742215
C	-0.183425	-1.872859	2.066718
H	0.597166	-2.422247	1.549850
C	0.454291	-1.608762	4.492175
H	1.247484	-2.358809	4.415791
H	0.748899	-0.877290	5.253384
H	-0.461335	-2.102263	4.828971
H	-0.922485	-3.716861	0.626168
